# Photocatalytic Fluoro Sulfoximidations of Styrenes

**DOI:** 10.1002/anie.202005844

**Published:** 2020-06-08

**Authors:** Chenyang Wang, Yongliang Tu, Ding Ma, Carsten Bolm

**Affiliations:** ^1^ Institute of Organic Chemistry RWTH Aachen University Landoltweg 1 52074 Aachen Germany

**Keywords:** fluorination, hypervalent compounds, photochemistry, radicals, sulfur

## Abstract

Reactions of difluoroiodotoluene with NH‐sulfoximines provide new hypervalent iodine(III) reagents, which photocatalytically transfer a fluoro and a sulfoximidoyl group onto styrenes with high regioselectivity. The substrate scope is broad with respect to both sulfoximines and olefins. Following an operationally simple protocol, a large library of fluorine‐containing N‐functionalized sulfoximines can be accessed. Results from mechanistic investigations revealed the importance of radical intermediates.

## Graphical abstract

**Double transfer**: New hypervalent iodine(III) reagents are formed by reactions of difluoroiodotoluene with NH‐sulfoximines. When applied in situ, they photocatalytically react with styrenes by transferring a fluoro and a sulfoximidoyl group onto the olefin with high regioselectivity. The operationally simple process provides a variety of fluorine‐containing N‐functionalized sulfoximines, which are difficult to prepare otherwise. Mechanistic investigations reveal the importance of radical intermediates.

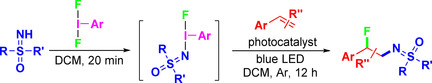

Hypervalent iodine compounds are tremendously important in modern organic chemistry, and recent developments have elevated them from lab curiosities to indispensable synthetic tools.^1^ With the intention to advance preparative high‐valent sulfur chemistry, we introduced the sulfoximidoyl‐containing iodine(III) reagents **1** 2 and **2** 3 and demonstrated their potential in sulfoximidations of alkynes,^2^ thiols,^4^ olefins,^5^ and benzylic substrates (Figure 1).^6^ Whereas in the first two transformations **1** and **2** reacted like electrophilic reagents (with a formal positive charge at nitrogen), the latter two reactions proceeded via nitrogen‐centered radicals formed by photocatalysis. Intending to progress this science we wondered about the accessibility and reactivity of the analogous fluoro‐containing iodine(III) reagents **3**. The introduction of such a molecule appeared attractive because its activation could eventually generate intermediates prone to giving products with both moieties, a sulfoximidoyl and fluoro group, being incorporated.^7^ Considering that both moieties are highly important in medicinal and crop protection chemistry,^8^, ^9^ the realization of this idea appeared attractive. The advance in this project is presented here.

**Figure 1:**
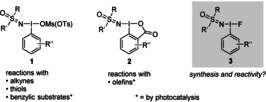
Reported sulfoximidoyl‐containing iodine(III) reagents **1** and **2** and targeted analogous compounds **3**.

As starting materials for the preparation of fluoro‐containing iodine(III) reagents (**3**), difluoro iodoarenes (**4**) appeared most promising.^10^ The fluorination chemistry of **4** is rich, and various ligand exchange reactions at the iodo core are known.^11^ Furthermore, the accessibility of **4** has recently been significantly improved, leading to efficient fluorination reactions with even catalytic amounts of such these interesting reagents.^12^

In the initial studies, difluoro iodotoluene (**4 a**) and sulfoximine **5 a** were applied as substrates (Scheme 1, top). Combining these two compounds in dichloromethane for 20 minutes led to a new fluorine‐containing species as revealed by ^1^H and ^19^F NMR spectroscopy (see the Supporting Information). Evaporation of the solvent afforded an oil, which proved sensitive to standard conditions of chromatography. ESI‐MS analysis showed a signal at *m*/*z* 357.97513 corresponding to a composition of [**3 a**‐F^−^]. Attempts to isolate analytically pure **3 a** failed.

**Scheme 1:**
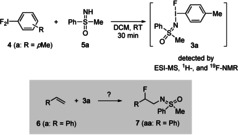
Preparation of the monofluoro sulfoximidoyl iodoarene **3 a** and its hypothesized addition to an olefin (**6**) to give a difunctionalized product (**7**).

Focusing on potential applications of **3 a**, an in situ approach was considered, thereby avoiding the need to isolate the apparently sensitive reagent. Functionalizing alkenes^13^ was set into focus with the goal of adding both the sulfoximidoyl and the fluoro group to the double bond to give products such as **7** (Scheme 1, bottom). Such transformations would then be analogous to aminofluorinations developed by the groups of Liu,14a Zhang,14b Pérez,14c Xu,14d and Xu,14e but contrasting them by neither requiring a metal catalyst nor relying on an external fluoride source such as Selectfluor as demonstrated by the groups of Studer15a and Lectka.15b

The first test reactions along these lines were performed with styrene (**6 a**, R=Ph) as the olefinic component. The reagent **3 a** was prepared in situ by mixing difluoroiodotoluene (**4 a**, 1.5 equiv) and the sulfoximine **5 a** (1.8 equiv). An extensive screening of the reaction conditions (for details see Table S2 in the Supporting Information) revealed that the highest yield of **7 aa** was achieved by following a stepwise process. First, **3 a** was prepared from **4 a** and **5 a** in a sealed tube with DCM as solvent under argon at ambient temperature, and then, after 20 minutes of stirring, **6 a** and Ru(bpy)_3_(PF_6_)_2_ (1 mol %) were sequentially added. Irradiation with a blue LED (24 W) for 12 hours followed by standard aqueous work‐up and chromatography afforded **7 aa** in 83 % yield (Scheme 2).^16^ Applying Ir(dtbpy)ppy)PF_6_, Rose Bengal, Eosin yellowish, Rhodamine B, or Ru(bpy)_3_) instead of Ru(bpy)_3_(PF_6_)_2_ as the photocatalyst proved less efficient.^17^ Upon addition of CsF, AgF, or CuF_2_ (2.0 equiv) the amount of formed **7 aa** was lowered. Without LED irradiation, the yield of **7 aa** was significantly reduced (up to 25 %) in a range of solvents (DCM, DCE, MeCN, THF, and toluene).^17^ Neither changing the addition mode nor altering the substrate ratio was beneficial.

**Scheme 2:**
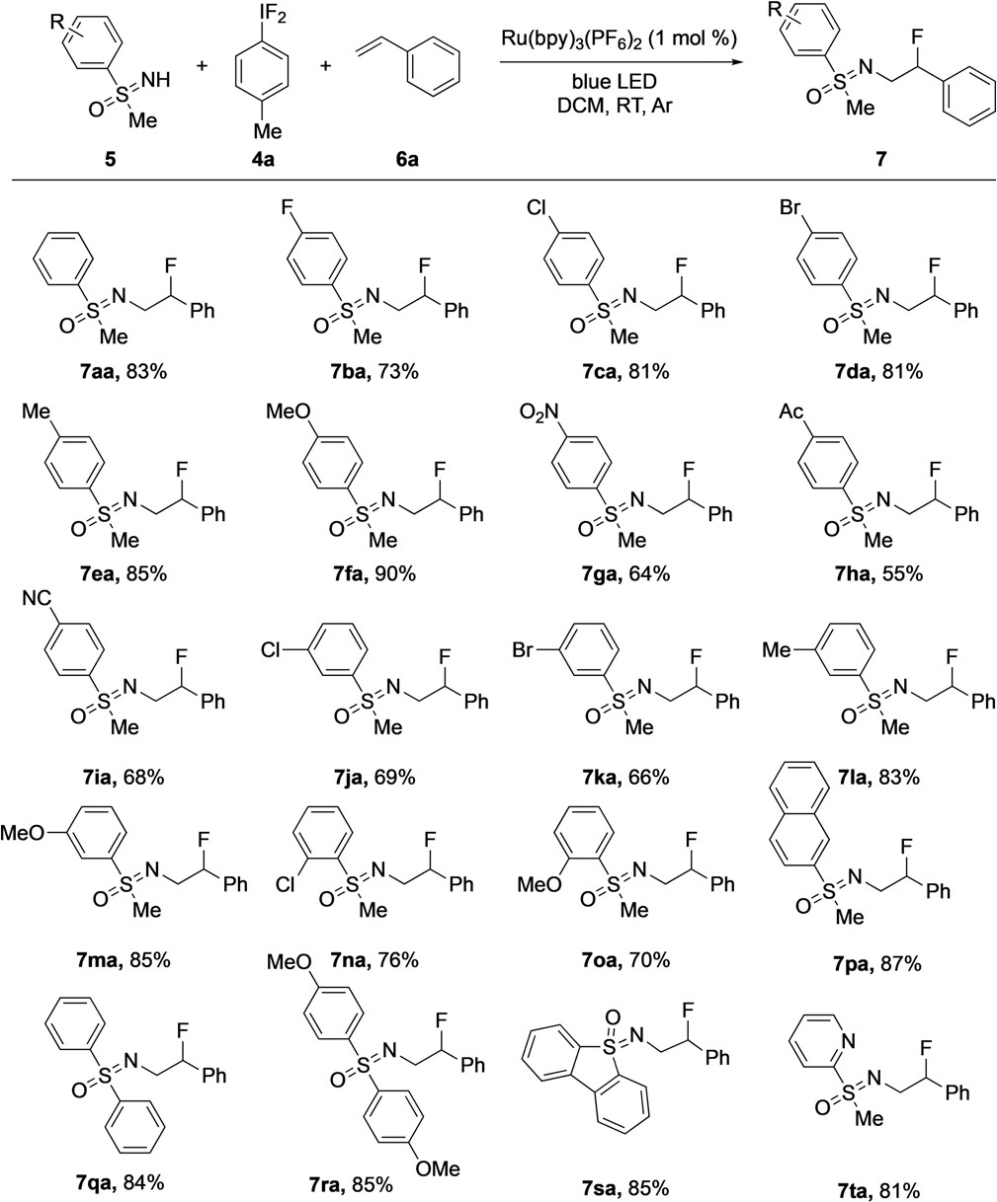
Substrate scope with respect to **5** in additions of in situ formed **3 a** to styrene (**6 a**).

Consequently, the substrate scope was evaluated under the aforementioned optimal reaction conditions. First, various sulfoximines (**5**) were used with **6 a** as the olefinic acceptor [and difluoro iodotoluene (**4 a**) as iodine(III) source]. Scheme 2 shows the results. The substrate scope with respect to the sulfoximine component proved quite general, and a wide range of functional groups, including halo, alkyl, alkoxy, acetoxy, and nitro substituents were tolerated. In the series of *S*‐aryl *S*‐methyl sulfoximines (**5 a**–**p**), substrates with both electron‐donating and electron‐withdrawing substituents on the arene reacted well, affording the corresponding products in yields ranging from 55 to 90 %. A more detailed analysis of the results in the series of *para*‐substituted products (**7 ea**–**ia**) suggested that sulfoximines with electron‐rich arenes (**7 ea** and **7 fa**) gave slightly better results than those with electron‐poor aryl groups (**7 ga**–**ia**). Comparing the yields for products stemming from the chloro‐containing sulfoximines **5 c**, **5 j**, and **5 n** and the methoxy‐substituted **5 f**, **5 m**, and **5 o** showed that in both cases the *para*‐substituted derivatives gave the highest yields of the corresponding products. Nevertheless, although not on the same level, the results for *meta*‐ and *ortho*‐substituted products were good too with yields ranging from 69 % (for **7 ja**) to 85 % (for **7 ma**). The *S*‐2‐naphthyl‐containing product **7 pa** was obtained in 87 % yield. Also *S*,*S*‐diaryl sulfoximines reacted well as exemplified by the results for the products **7 qa** and **7 ra**, which were obtained in 84 and 85 % yield, respectively. Finally, the high yields in the formation of **7 sa** (85 %) and **7 ta** (81 %) were noteworthy because the former involved the use of the dibenzothiophene sulfoximine **5 s**, which in former studies had revealed a rather unusual nitrogen‐transfer behavior,^18^ and the latter stemmed from the sulfoximine **5 t**, representing the only substrate with an *S*‐heteroaryl moiety. All additions occurred with high regioselectivity leading to products with the fluoro substituent in the benzylic position. In each case, the diastereomeric ratio was about 1:1.

Next, the olefinic component was varied. As precursors for the in situ formed iodine(III) reagent, a combination of **4 a** and **5 f** was used (Scheme 3). The applicability of 1‐styrenes with monosubstituted aryl groups (**6 b**–**r**) was studied first. Also in this series the substrate scope was broad. The yields varied, but generally, they were high. The best results were observed with *para*‐substituted styrenes as exemplified by the products **7 ff** (91 %) and **7 fh** (93 %) bearing a *para*‐*tert*‐butyl and a *para*‐phenyl group, respectively. Substrates with *meta*‐ and *ortho*‐substituents gave products (**7 fk**–**ft**) in somewhat diminished yields with 56 % being the lowest as observed in the formation of **7 fk** having a *meta*‐fluoro substituent. Representing substrates with more than one substituent on the arene, 1‐naphthyl‐ and mesityl styrene (**6 s** and **6 t**) gave the corresponding products (**7 fs** and **7 ft**) in 86 % and 50 % yield, respectively. The latter result was particularly interesting because the dr was 4:1, thereby contrasting all other reactions with dr ratios of about 1:1. Using the 1,1‐disubstituted styrenes **6 u** and **6 v** afforded **7 fu** (83 %) and **7 fv** (85 %), respectively. Finally, to our surprise and delight, also dihydronaphthaline **6 w** reacted well leading to the corresponding addition product **7 fw** in 73 % yield. This result was remarkable because it showed that 1,2‐disubstituted styrene derivatives could also react selectively.

**Scheme 3:**
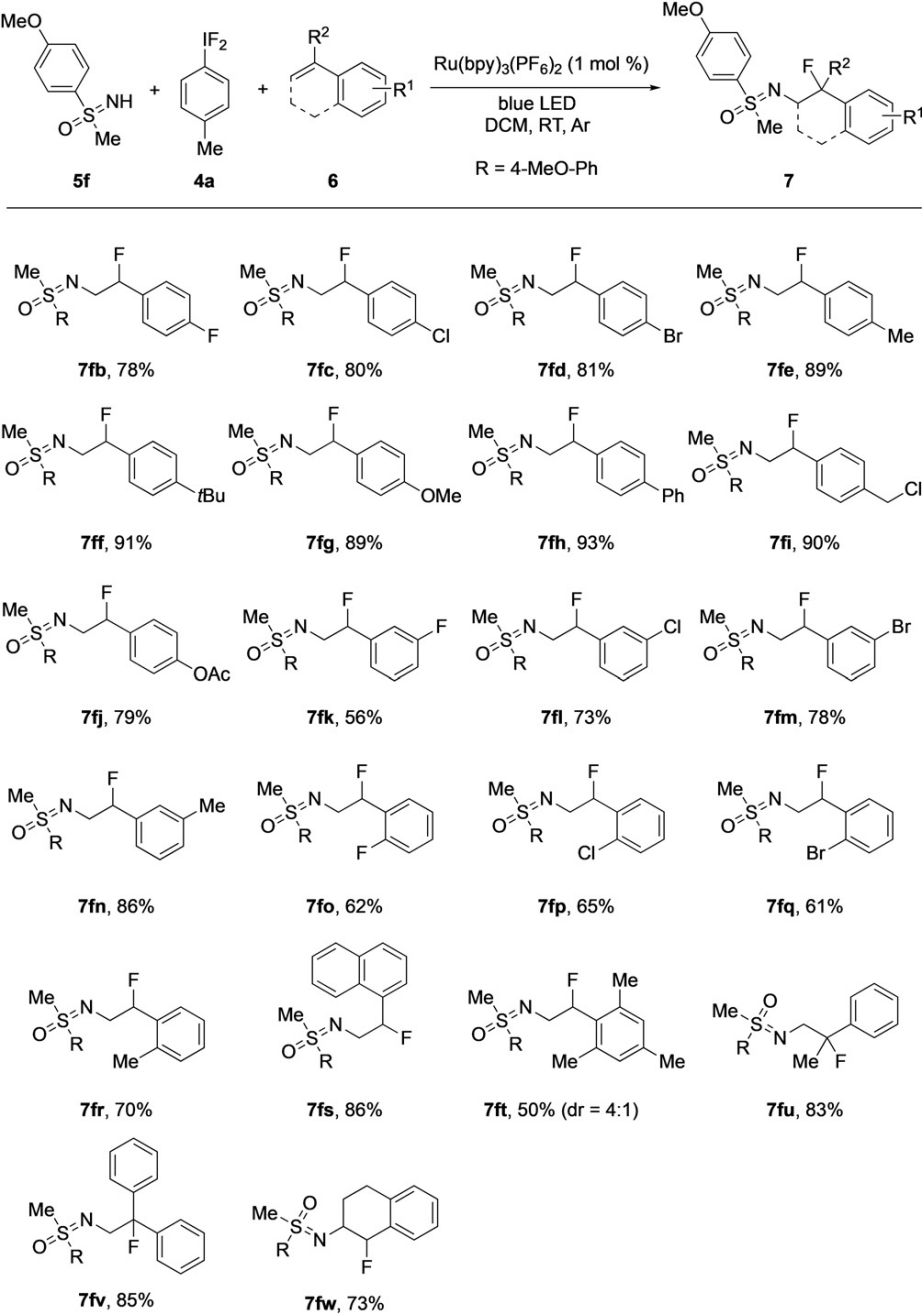
Substrate scope with respect to **6** by addition of the in situ formed iodine(III) reagent starting from **4 a** and **5 f**.

To elucidate the reaction pathway, various process modifications were studied (Scheme 4, top). As noted before, **3 a** could be identified by mass spectrometry and NMR spectroscopy. The stepwise protocol involving an addition of **6 a** to a solution of in situ formed **3 a** followed by irradiation of the resulting mixture with a blue LED in the presence of the photocatalyst gave **7 aa** in 83 % yield. If all reagents were mixed without the separate preformation of **3 a**, the yield of **7 aa** was reduced to 68 %, suggesting that under these reaction conditions parts of the starting materials reacted differently (specific by‐products formed in trace amounts have not been identified). This observation was in line with the result from an experiment with altered mixing order. Thus, an initial stirring of the iodine reagent **4 a** in the presence of **6 a** for 20 minutes followed by the addition of **5 a** and the photocatalyst with subsequent blue LED irradiation did not lead to any detectable amounts of **7 aa**. Apparently, other (unidentified) reaction pathways dominated. Following the original protocol but generating **3 a** in the presence of base (3 equiv of K_2_CO_3_) reduced the yield of **7 aa** to 55 %, indicating a decisive role of the intermediately formed HF. No product was observed when the reaction (after the in situ formation of **3 a**) was performed in the presence of TEMPO (2 equiv), suggesting an involvement of radicals as relevant intermediates.

**Scheme 4:**
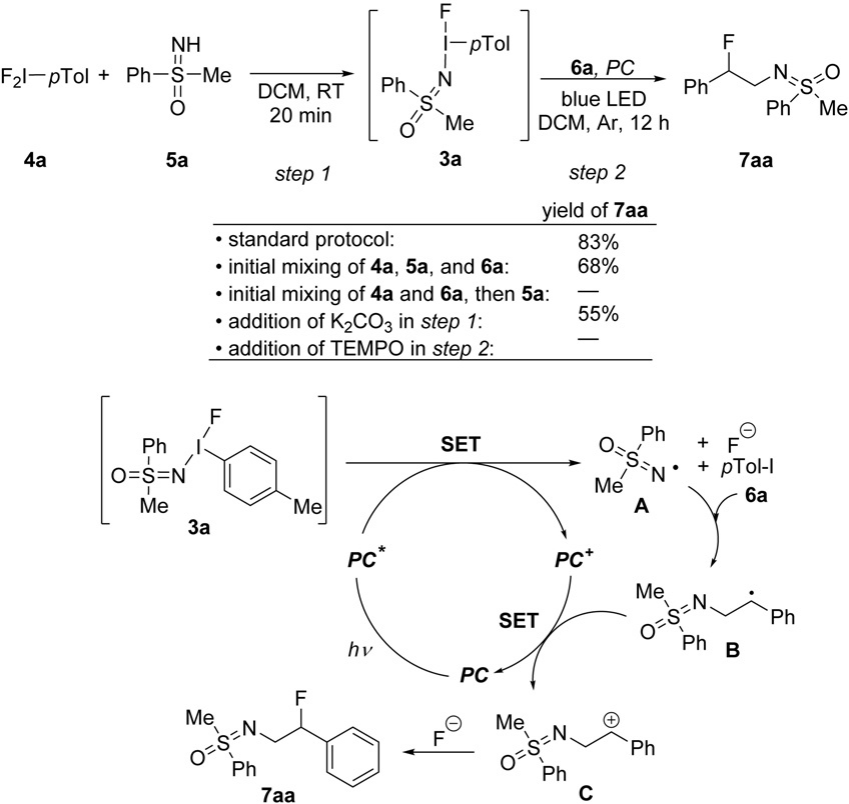
Standard protocol and variations thereof (top); suggested mechanistic pathway (bottom).

Taking all observations into account, the following mechanistic scenario is suggested: After the initial formation of **3 a** (Scheme 4, step 1), the N−I bond of **3 a** is cleaved by single‐electron transfer (SET) from the excited state of Ru(bpy)_3_(PF_6_)_2_ (PC*), which was generated from the ground state of the photocatalyst by visible light. As a consequence, the N‐centered sulfoximidoyl radical **A**, a fluoride ion, *p*‐tolyl iodide, and the oxidized photocatalyst (*PC* ^*+*^) are formed. Subsequently, **A** adds to the double bond of **6 a** leading to the radical **B**. The benzylic stabilization is critical for the entire process and the basis for its high regioselectivity.^19^ Oxidation of **B** by SET from *PC* ^*+*^ provides the benzylic cation **C** and closes the catalytic cyclic by regenerating the ground‐state photocatalyst *PC*. Finally, **C** reacts with fluoride to give **7 aa**.^20^

In summary, we developed a new in situ formed hypervalent iodine(III) reagent, which photocatalytically adds two of its iodine‐bound substituents to styrenes, providing fluorine‐containing N‐functionalized sulfoximines in a single operational step. The fluoro sulfoximinations show a pronounced functional‐group tolerance and occur with high regioselectivity. Mechanistic studies suggest the intermediacy of radicals.

## Conflict of interest

The authors declare no conflict of interest.

## Supporting information

As a service to our authors and readers, this journal provides supporting information supplied by the authors. Such materials are peer reviewed and may be re‐organized for online delivery, but are not copy‐edited or typeset. Technical support issues arising from supporting information (other than missing files) should be addressed to the authors.

Supplementary
