# Supplementary material for: Photocatalytic Fluoro Sulfoximidations of Styrenes
Source: Angew Chem Int Ed Engl. 2020 Jun 8;59(33):14134–7. doi: 10.1002/anie.202005844 (PMC7496861; doi:10.1002/anie.202005844)
Supplement: Supplementary file 1 — Supplementary [file ANIE-59-14134-s001.pdf]

Angewandte Chemie
Eine Zeitschrift der Gesellschaft Deutscher Chemiker

Supporting Information

Photocatalytic Fluoro Sulfoximidations of Styrenes

*Chenyang Wang, Yongliang Tu, Ding Ma, and Carsten Bolm**

anie_202005844_sm_miscellaneous_information.pdf

Supporting Information

Table of Contents

1. General Information	S1
2. Preparation of the hypervalent iodine reagents	S2
3. Fluoro sulfoximidations of styrenes	S5
4. Reference	S5
5. Characterizing data	S6
6. NMR Spectra	S22

1. General Information

Unless otherwise noted, the materials were purchased from commercial suppliers and used without further purification. All the solvents were treated according to general methods. The reactions were monitored by thin layer chromatography (TLC) with aluminium sheets silica gel 60 F_{254} from Merck, and flash column chromatography purifications were performed using silica gel 60 (63-200 µm) from Merck. ^{1}H and ^{13}C NMR spectra were recorded with an Agilent VNMRS 600, Agilent VNMRS 400 or Varian Mercury 300 in deuterated solvents. Chemical shifts (δ) are reported in parts per million (ppm) and spin-spin coupling constants (J) are given in Hz, while multiplicities are abbreviated by s (singlet), d (doublet), t (triplet), q (quartet), br (broad), m (multiplet). The IR spectra were recorded with a PerkinElmer Spectrum 100 spectrometer with an attached UATR device Diamond KRS-5. All IR data were collected by attenuated total reflectance (ATR) and wavenumbers ν are given in cm^{-1}. Mass spectra were recorded with a Finnigan SSQ Finnigan 7000 spectrometer (EI, 70 eV). High resolution mass spectra (HRMS) were recorded on a Thermo Scientific LTQ Orbitrap XL spectrometer. Melting points (mp) were determined on a Büchi B-540 melting point apparatus. Determination and separation of stereoisomers were performed by analytical high-performance liquid chromatography (HPLC) on an Agilent 1200-series with a Chiralpak AD-H column (250 mm · 4.6 mm) from Chiral Technologies Inc. as chiral stationary phase (CSP) and by preparative supercritical fluid chromatography (SFC) on a Thar SFC Prep 80 from Waters with a Chiralpak IA column (250 mm · 19 mm). *N*H-sulfoximines[S1] were prepared in accordance with previously published synthetic strategies.

2. Preparation of the hypervalent iodine reagents[S2]

2.1 Preparation of *p*TolIF$_2$ (4a)

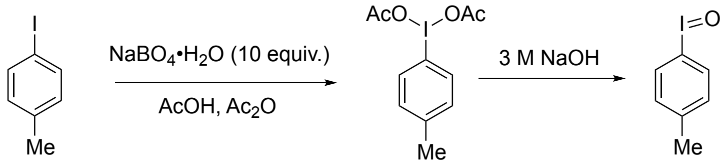

In a 250 mL round-bottom flask equipped with a magnetic stir bar, 4-iodotoluene (4.36 g, 20 mmol) and acetic anhydride (50 mL) were dissolved in AcOH (120 mL) and heated to 45 ^{o}C. Then, $NaBO_3{\cdot}4H_2O$ (10 equiv., 30.7 g, 200 mmol) was added over a period of 30 min, and the mixture was stirred overnight at this temperature. When the reaction was completed, the mixture was cooled to room temperature and concentrated to about half volume. After water (100 mL) was added, the mixture was extracted with CH_2Cl_2 (3×50 mL). The combined organic layers were washed with brine, and the solvent was then removed under vacuum. Subsequently, the crude product was moved to a 100 mL round-bottom flask, and 3M NaOH (50 mL) was added dropwise in 30 min with vigorous stirring. The mixture was stirred for 2 h, diluted with water (30 mL) and vigorously stirred for an additional hour. The product was filtered off using a Buchner funnel and washed with water (3×15 mL) and $CHCl_3$ (2×15 mL). After drying in vacuo, 4-iodosyltoluene was obtained as a yellow solid (3.4 g, 72%).

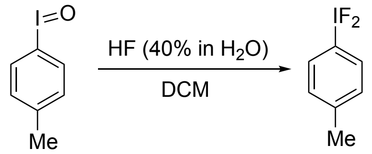

In a 30 mL bottle equipped with a magnetic stir bar, 4-iodosyltoluene (2.3 g, 10 mmol) was mixed with CH_2Cl_2 (20 mL). An aqueous solution of HF (40%, 7.0 mL) was slowly added in 30 min under vigorous stirring. The mixture was stirred for further 30 min until a clear solution was formed. The organic layer was separated, and the aqueous layer was extracted with CH_2Cl_2 (3×3 mL). The solvent of the combined organic layer was removed. 4-(Difluoroiodo)toluene (**4a**, *p*TolIF$_2$) was obtained as a light yellow solid (2.3 g, 90%) and purified by recrystallization from hexane/chloroform if necessary. *Noted: pTolIF$_2$ is quite sensitive to moisture and glass thus all aforementioned operations were performed in plastic vessels under argon. A storage in fluoropolymer containers has been recommended.* ^{1}H NMR (300 MHz, $CDCl_3$) δ 7.83 (dd, J = 8.5, 1.7 Hz, 2H), 7.42 – 7.35 (m, 2H), 2.46 (s, 3H). ^{19}F NMR (282 MHz, $CDCl_3$) δ –176.72.

2.2 Syntheses of sulfoximidoyl-containing hypervalent iodine(III) reagents

In a 10 mL plastic tube equipped with a magnetic stir bar, *p*TolIF$_2$ (51.2 mg, 0.2 mmol) and the *N*H-sulfoximine (0.24 mmol) were mixed with CH_2Cl_2 (2 mL) under vigorous stirring for 20 min. After the reaction was complete, the solvent was removed under vacuum, and the residue was dried in vacuo to afford the respective crude iodine(III) reagent.

Table S1. Preparation of **3a** from **4a** and **5a**; variations of the reagent amounts and analysis by ^{1}H and ^{19}F NMR spectroscopy as presented in Figs. 1 and 2, respectively.

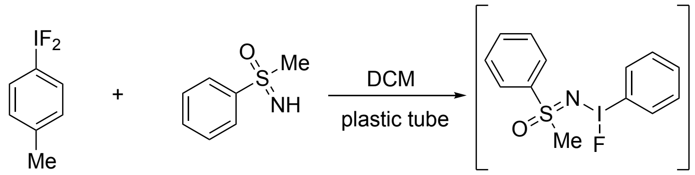

4a **5a** **3a**

Entry	*p*TolIF$_2$ [mmol]	Sulfoximine **5a** [mmol]
A	0.2	0.2
B	0.2	0.24
C	0.24	0.2

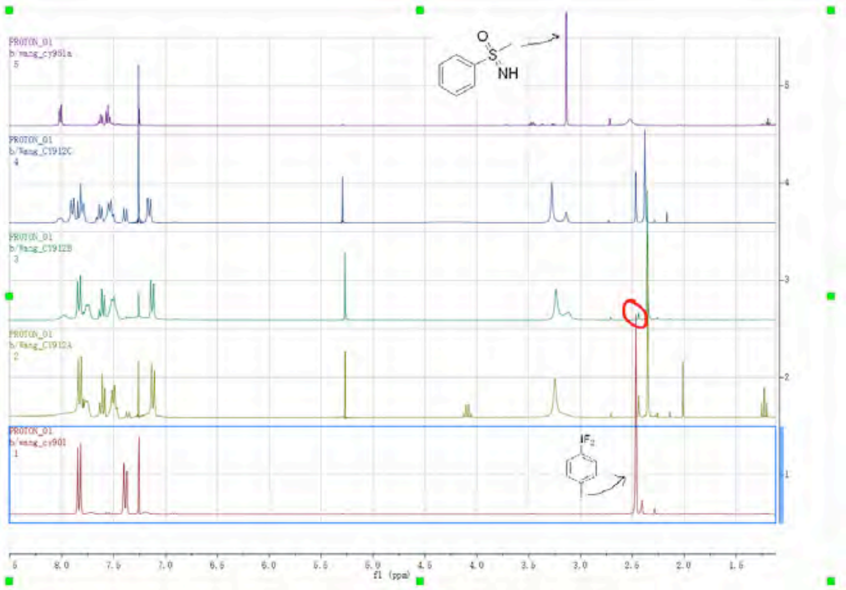

Figure S1. ^{1}H NMR (300 MHz, $CDCl_3$) spectra of substrates and reaction mixtures

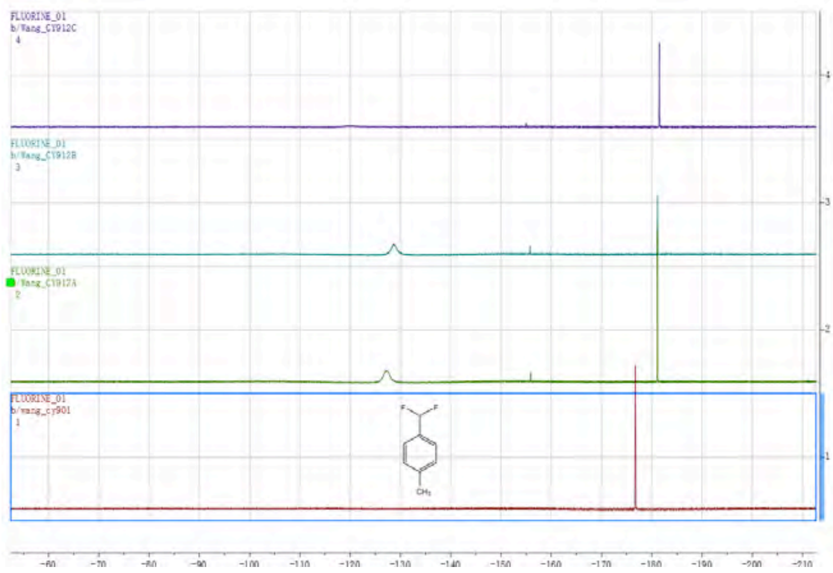

Figure S2. ^{19}F NMR (282 MHz, $CDCl_3$) spectra of substrates and reaction mixtures.

3. Fluoro sulfoximidations of styrenes

3.1. Reaction optimization for the fluoro sulfoximidations of styrenes [as studied for the synthesis of 7aa starting from *p*TolIF$_2$ (4a), sulfoximine 5a, and styrene (6a)].

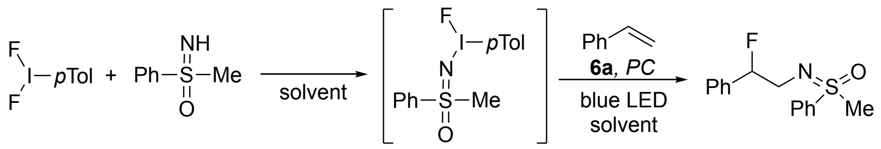

4a **5a** **3a** **7aa**

In a 5.0 mL sealable reaction tube under an argon atmosphere, sulfoximine **5a** was dissolved in the given solvent (1.0 mL). Then, freshly prepared 4-(difluoroiodo)toluene (**4a**) was added, and the reaction mixture was stirred for about 20 min to get clear solution. Subsequently, a mixture of styrene (**6a**) and the photocatalyst (*PC*, 1% mol) in the given solvent (1 mL) was added, and the mixture was stirred with blue LED irradiation (24 W) at room temperature for 12 h. Product **7aa** was then purified by flash column chromatography (ethyl acetate/ *n*-pentane = 1/2 to 1.5/1). The diastereomeric ratio of **7aa** was determined by ^{19}F NMR spectroscopy of the crude product.
*Note: In all reactions freshly prepared p-TolIF$_2$ (**4a**) were used.*

Table S2. Reaction optimization of the synthesis of **7aa**.[a]

Entry	Photocatalyst (*PC*)	Additive	Solvent	Yield[b]
1	--	--	CH_3CN	trace[c]
2	--	--	THF	trace[c]
3	--	--	Toluene	trace[c]
4	--	--	DCE	7[c]
5	--	--	DCM	13[c]
6	--	--	DCM	25
7	$Ir^{III}(dtbpy)(ppy)PF_4$	--	DCM	55
8	Rose Bengal	--	DCM	53
9	Eosin yellowish	--	DCM	51
10	Rhodamine B	--	DCM	36
11	$Ru(bpy)_3$	--	DCM	42
12	$Ru(bpy)_3(PF_6)_2$	--	DCM	83
13	$Ru(bpy)_3(PF_6)_2$	CsF	DCM	45
14	$Ru(bpy)_3(PF_6)_2$	AgF	DCM	65
15	$Ru(bpy)_3(PF_6)_2$	CuF_2	DCM	56
16	$Ru(bpy)_3(PF_6)_2$	--	DCM	68[d]
17	$Ru(bpy)_3(PF_6)_2$	--	DCM	79[e]
18	$Ru(bpy)_3(PF_6)_2$	--	DCM	51[f]
19	$Ru(bpy)_3(PF_6)_2$	--	DCM	84[g]

[a] Reaction conditions: **4a** (0.30 mmol), **5a** (0.36 mmol), **6a** (0.20 mmol), catalyst (1 mol %), additive (0.4 mmol), solvent (2 mL), under Ar, 12 h, sealed tube. DCE = dichloroethane, dtbpy = 4,4'-di-*tert*-butyl-2,2'-dipyridyl, ppy = phenylpyridine, bpy = 2,2'-bipyridine. [b] After column

chromatography. [c] Under air. [d] Immediate mixing of all starting materials [**4a** (0.36 mmol), **5a** (0.3 mmol), **6a** (0.2 mmol)]. [e] Use of **4a** (0.4 mmol), **5a** (0.48 mmol), and **6a** (0.2 mmol). [f] 6 h. [g] 24 h.

3.2. General procedure for the fluoro sulfoximidations of styrenes

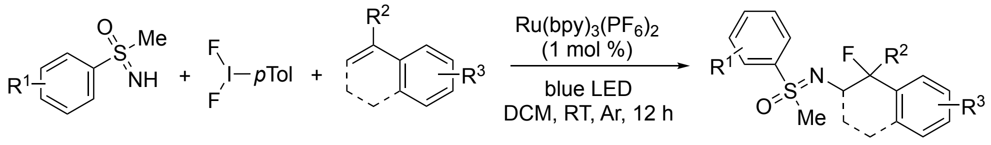

In a 5.0 mL sealable reaction tube under an argon atmosphere, sulfoximines **5** (0.36 mmol, 1.8 equiv.) was dissolved in DCM (1.0 mL). Then, freshly prepared 4-(difluoroiodo)toluene (**4a**, 0.3 mmol, 1.5 equiv.) was added, and the reaction mixture was stirred for about 20 min to get clear solution. Subsequently, styrenes **6** (0.2 mmol) and $Ru(bpy)_3(PF_6)_2$ (1.7 mg, 2 μmol, 1 mol %) in DCM (1 mL) was added, and the mixture was stirred with blue LED irradiation (24 W) at room temperature for 12 h. The products were then purified by flash column chromatography (ethyl acetate/ *n*-pentane = 1/2 to 1.5/1). The diastereomeric ratios were determined by ^{19}F NMR spectroscopy of the crude product mixture. *Note: In all reactions freshly prepared p-TolIF$_2$ were used.*

4. Reference

[S1] a) A. Pandey, C. Bolm, *Synthesis* **2010**, 2922-2925; b) A. Tota, M. Zenzola, S. J. Chawner, S. S. John-Campbell, C. Carlucci, G. Romanazzi, L. Degennaro, J. A. Bull, R. Luisi, *Chem. Commun.* **2017**, *53*, 348-351.

[S2] a) B. Xing, C. Ni, J. Hu, *Angew. Chem. Int. Ed.* **2018**, *57*, 9896-9900; b) D. M. Lemal, J. Tao, G. K. Murphy, *Encyclopedia of Reagents for Organic Synthesis* **2003**, 1-3; c) M. A. Arrica, T. Wirth, *Eur. J. Org. Chem.* **2005**, 395-403.

5. Characterizing Data

[(2-Fluoro-2-phenylethyl)imino](methyl)(phenyl)-λ^6-sulfanone (7aa)

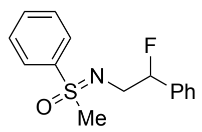

Following the general procedure afforded the product as a yellow viscous oil (46 mg, 83% yield). dr = 1 : 1.3. ^{1}H NMR (600 MHz, $CDCl_3$, mixture of diastereomers) δ 7.90 – 7.87 (m, 2.6H), 7.79 (dt, J = 8.3, 1.1 Hz, 2H), 7. 62 – 7.57 (m, 2H), 7.53 (dt, J = 20.3, 7.7 Hz, 4.6H), 7.35 – 7.28 (m, 11.5H), 5.55 (tdd, J = 47.4, 8.1, 3.8 Hz, 2.6H), 3.46 (ddd, J = 16.0, 13.4, 7.6 Hz, 1.3H), 3.35 – 3.25 (m, 1.3H), 3.18 (ddd, J = 18.2, 13.7, 8.5 Hz, 2H), 3.12 (s, 3.9H), 3.10 (s, 3H). ^{13}C {^{1}H} (151 MHz, $CDCl_3$, mixture of diastereomers) δ 139.0 (d, J_{FC} = 28.4 Hz), 138.5 (d, J_{FC} = 20.0 Hz), 133.0, 133.0, 129.5, 129.4, 128.8, 128.6, 128.3, 128.3, 126.0 (d, J_{FC} = 7.0 Hz), 125.6 (d, J_{FC} = 7.2 Hz), 95.4 (d, J_{FC} = 174.1 Hz), 94.5 (d, J_{FC} = 174.1 Hz), 49.8 (d, J_{FC} = 24.4 Hz), 49.6 (d, J_{FC} = 27.2 Hz), 45.3, 45.0. ^{19}F NMR (564 MHz, $CDCl_3$, mixture of diastereomers) δ –179.25 (ddd, J = 47.2, 26.0, 15.9 Hz), –181.26 (ddd, J = 48.4, 32.8, 18.2 Hz). MS (EI): m/z (%) = 169 (10), 168 (100), 141 (60), 125 (16), 109 (32), 97 (6), 83 (9), 77 (11), 51 (8). IR (ATR): v = 3062, 2926, 2842, 2167, 1446, 1230, 1143, 1085, 1031, 978, 870, 745, 694 cm^{-1}. HRMS m/z: calcd for $[C_{15}H_{16}FNOS+Na]^+$: 300.0829, found: 300.0826.

[(2-Fluoro-2-phenylethyl)imino](4-fluorophenyl)(methyl)-λ^6-sulfanone (7ba)

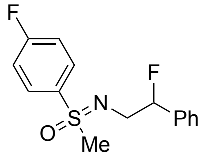

Following the general procedure afforded the product as a yellow viscous oil (43 mg, 73% yield). dr = 1 : 1. ^{1}H NMR (600 MHz, $CDCl_3$, mixture of diastereomers) ^{1}H NMR (600 MHz, $CDCl_3$, mixture of diastereomers) δ 7.89 – 7.85 (m, 2H), 7.78 – 7.73 (m, 2H), 7.37 – 7.27 (m, 10H), 7.23 – 7.13 (m, 4H), 5.64 – 5.44 (m, 2H), 3.51 – 3.42 (m, 1H), 3.35 – 3.25 (m, 1H), 3.18 – 3.12 (m, 2H), 3.11 (s, 3H), 3.08 (s, 3H). ^{13}C {^{1}H} (151 MHz, $CDCl_3$, mixture of diastereomers) δ 171.1, 166.3 (d, J_{FC} = 11.1 Hz), 164.6 (d, J_{FC} = 11.8 Hz), 138.5 (dd, J_{FC} = 35.8, 19.4 Hz), 135.0 (d, J_{FC} = 3.0 Hz), 134.8 (d, J_{FC} = 3.0 Hz), 131.6 (d, J_{FC} = 9.3 Hz), 131.3 (d, J_{FC} = 9.3 Hz), 128.4, 128.4, 128.4, 128.3, 128.3, 126.0 (d, J_{FC} = 7.0 Hz), 125.6 (d, J_{FC} = 7.0 Hz), 116.7 (dd, J_{FC} = 22.5, 8.5 Hz), 95.5 (d, J_{FC} = 174.8 Hz), 94.3 (d, J_{FC} = 174.8 Hz), 49.8 (d, J_{FC} = 23.8 Hz), 49.5 (d, J_{FC} = 27.2 Hz), 45.5, 45.2. ^{19}F NMR (564 MHz, $CDCl_3$, mixture of diastereomers) δ –105.17 (tdd, J = 13.1, 8.6, 5.1 Hz), –179.28 (ddd, J = 47.6, 24.8, 15.9 Hz), –181.41 (ddd, J = 48.5, 32.9, 18.6 Hz). MS (EI): m/z (%) = 186 (71), 159 (67), 143 (30), 115 (7), 109 (100), 107 (8), 95 (7), 83 (24), 77 (8). IR (ATR): v = 3066, 3034, 2929, 2843, 2164, 1588, 1490, 1453, 1404, 1316, 1227, 1141, 1085, 1031, 980, 870, 839, 761, 699 cm^{-1}. HRMS m/z: calcd for $[C_{15}H_{15}F_2NOS+Na]^+$: 318.0735, found: 318.0733.

(4-Chlorophenyl)[(2-fluoro-2-phenylethyl)imino](methyl)-λ^6-sulfanone (7ca)

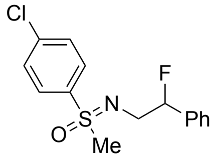

Following the general procedure afforded the product as a yellow viscous oil (50 mg, 81% yield). dr = 1 : 1.1. ^{1}H NMR (600 MHz, $CDCl_3$, mixture of diastereomers) δ 7.81 – 7.77 (m, 2.2H), 7.69 – 7.66 (m, 2H), 7.51 – 7.48 (m, 2.2H), 7.47 – 7.44 (m, 2H), 7.37 – 7.27 (m, 11H), 5.63 – 5.43 (m, 2.2H), 3.46 (ddd, J = 15.9, 13.3, 7.3 Hz, 1H), 3.30 (ddd, J = 32.7, 13.9, 2.8 Hz, 1.2H), 3.18 – 3.11 (m, 2H), 3.10 (s, 3.7H), 3.07 (s, 3H). ^{13}C {^{1}H} (151 MHz, $CDCl_3$, mixture of diastereomers) δ 139.7, 139.6, 138.5 (d, J_{FC} = 20.0 Hz), 138.3 (d, J_{FC} = 20.0 Hz), 137.6, 137.4, 130.3, 130.0, 129.7, 129.6, 128.4, 128.4, 128.3, 128.3, 128.2, 126.0 (d, J_{FC} = 6.8 Hz), 125.6 (d, J_{FC} = 6.8 Hz), 95.4 (d, J_{FC} = 175.7

Hz), 94.5 (d, J_{FC} = 175.7 Hz), 49.8 (d, J_{FC} = 23.3 Hz), 49.5 (d, J_{FC} = 23.3 Hz), 45.3, 45.0. ^{19}F NMR (564 MHz, $CDCl_3$, mixture of diastereomers) δ –179.18 (ddd, J = 47.5, 24.7, 15.9 Hz), –181.31 (ddd, J = 48.3, 32.8, 18.7 Hz). MS (EI): m/z (%) = 204 (39), 202 (100), 177 (21), 175 (55), 160 (12), 108 (38), 83 (11), 77 (5), 75 (7). IR (ATR): ν = 3030, 2926, 2325, 1575, 1472, 1393, 1315, 1230, 1144, 1082, 979, 871, 829, 771, 699 cm^{-1}. HRMS m/z: calcd for $[C_{15}H_{15}ClFNOS+Na]^+$: 334.0439, found: 334.0439.

(4-Bromophenyl)[(2-fluoro-2-phenylethyl)imino](methyl)-λ^6-sulfanone (7da)

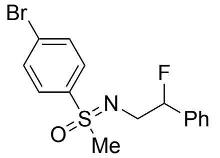

Following the general procedure afforded the product as a colorless oil (58 mg, 81% yield). dr = 1 : 1. ^{1}H NMR (600 MHz, $CDCl_3$, mixture of diastereomers) δ 7.72 – 7.70 (m, 2H), 7.67 – 7.65 (m, 2H), 7.63 – 7.58 (m, 4H), 7.36 – 7.26 (m, 10H), 5.64 – 5.42 (m, 2H), 3.49 – 3.41 (m, 1H), 3.34 – 3.25 (m, 1H), 3.17 – 3.10 (m, 2H), 3.10 (s, 3H), 3.06 (s, 3H). ^{13}C {^{1}H} (151 MHz, $CDCl_3$, mixture of diastereomers) δ 138.5 (d, J_{FC} = 19.4 Hz), 138.3 (d, J_{FC} = 19.4 Hz), 138.2, 138.0, 132.8, 132.7, 130.5, 130.2, 128.4, 128.4, 128.4, 128.3, 128.3, 128.2, 125.8 (d, J_{FC} = 57.2 Hz), 125.7 (d, J_{FC} = 57.2 Hz), 95.8 (d, J_{FC} = 177.2 Hz), 94.5 (d, J_{FC} = 177.2 Hz), 49.8 (d, J_{FC} = 23.6 Hz), 49.5 (d, J_{FC} = 23.6 Hz), 45.3, 45.1. ^{19}F NMR (565 MHz, $CDCl_3$, mixture of diastereomers) δ –179.15 (ddd, J = 47.3, 24.7, 15.9 Hz), –181.29 (ddd, J = 48.6, 32.6, 18.6 Hz). MS (EI): m/z (%) = 356 (7), 248 (72), 246 (100), 221 (45), 219 (45), 204 (16), 202 (11), 140 (5), 109 (57), 96 (5), 83 (14), 77 (6), 63 (8), 50 (7). IR (ATR): ν = 3029, 2926, 2842, 2165, 1570, 1462, 1386, 1230, 1143, 1065, 1033, 978, 871, 825, 768, 699 cm^{-1}. HRMS m/z: calcd for $[C_{15}H_{15}BrFNOS+Na]^+$: 377.9933, found: 377.9934.

[(2-Fluoro-2-phenylethyl)imino](methyl)(*p*-tolyl)-λ^6-sulfanone (7ea)

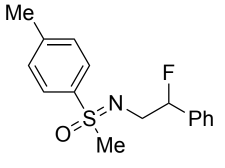

Following the general procedure afforded the product as a yellow viscous oil (49 mg, 85% yield). dr = 1 : 1. ^{1}H NMR (600 MHz, $CDCl_3$, mixture of diastereomers) δ 7.76 – 7.73 (m, 2H), 7.68 – 7.64 (m, 2H), 7.36 – 7.26 (m, 14H), 5.64 – 5.43 (m, 2H), 3.44 (ddd, J = 15.9, 13.4, 7.6 Hz, 1H), 3.28 (ddd, J = 33.1, 13.8, 3.0 Hz, 1H), 3.20 – 3.14 (m, 1H), 3.13 – 3.08 (m, 4H), 3.07 (s, 3H), 2.42 (s, 3H), 2.42 (s, 3H). ^{13}C {^{1}H} (151 MHz, $CDCl_3$, mixture of diastereomers) δ 143.9, 143.8, 138.7 (d, J_{FC} = 19.7 Hz), 138.5 (d, J_{FC} = 19.7 Hz), 135.9, 135.6, 130.1, 130.0, 128.8, 128.5, 128.3, 128.2, 128.2, 128.2, 128.2, 125.9 (d, J_{FC} = 6.7 Hz), 125.6 (d, J_{FC} = 6.6 Hz), 95.5 (d, J_{FC} = 176.2 Hz), 94.8 (d, J_{FC} = 176.2 Hz), 49.9 (d, J_{FC} = 23.4 Hz), 49.6 (d, J_{FC} = 23.4 Hz), 45.4, 45.1, 21.4. ^{19}F NMR (565 MHz, $CDCl_3$, mixture of diastereomers) δ –179.16 (ddd, J = 47.1, 26.3, 15.8 Hz), –181.18 (ddd, J = 48.1, 33.1, 18.3 Hz). MS (EI): m/z (%) = 183 (10), 182 (100), 155 (59), 139 (23), 109 (35), 91 (10), 83 (9), 77 (5), 65 (6). IR (ATR): ν = 3057, 2925, 2841, 2159, 1588, 1499, 1453, 1348, 1228, 1139, 1069, 1028, 983, 866, 820, 756, 699 cm^{-1}. HRMS m/z: calcd for $[C_{16}H_{18}FNOS+Na]^+$: 314.0985, found: 314.0985.

[(2-Fluoro-2-phenylethyl)imino](4-methoxyphenyl)(methyl)-λ^6-sulfanone (7fa)

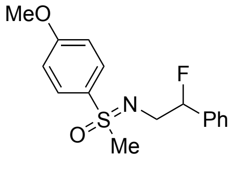

Following the general procedure afforded the product as a colorless oil (55 mg, 90% yield). dr = 1 : 1. ^{1}H NMR (600 MHz, $CDCl_3$, mixture of diastereomers) δ 7.81 – 7.77 (m, 2H), 7.72 – 7.67 (m, 2H), 7.35 – 7.27 (m, 10H), 7.02 – 6.94 (m, 4H), 5.54 (tdd, J = 48.1, 8.1, 3.8 Hz, 2H), 3.86 (d, J =

1.1 Hz, 3H), 3.86 (d, J = 1.1 Hz, 3H), 3.44 (ddd, J = 15.9, 13.4, 7.6 Hz, 1H), 3.28 (ddd, J = 33.3, 13.9, 2.9 Hz, 1H), 3.20 – 3.15 (m, 1H), 3.14 – 3.08 (m, 4H), 3.07 (s, 3H). ^{13}C {^{1}H} (151 MHz, $CDCl_3$, mixture of diastereomers) δ 163.2, 163.2, 138.7 (d, J_{FC} = 19.5 Hz), 138.5 (d, J_{FC} = 19.5 Hz), 130.9, 130.7, 130.1, 129.9, 128.3, 128.2, 128.2, 126.0 (d, J_{FC} = 6.7 Hz), 125.6 (d, J_{FC} = 6.7 Hz), 114.6, 114.6, 95.5 (d, J_{FC} = 174.5 Hz), 94.7 (d, J_{FC} = 174.5 Hz), 55.6, 49.9 (d, J_{FC} = 24.0 Hz), 49.6 (d, J_{FC} = 27.0 Hz), 45.6, 45.3. ^{19}F NMR (564 MHz, $CDCl_3$, mixture of diastereomers) δ –179.16 (dddd, J = 46.3, 26.1, 15.8, 3.7 Hz), –181.21 (ddd, J = 50.1, 33.3, 18.3 Hz). MS (EI): m/z (%) = 308 (6), 199 (9), 198 (100), 171 (47), 155 (36), 154 (12), 109 (27), 83 (6), 77 (5). IR (ATR): ν = 3029, 2929, 2840, 2171, 1590, 1494,1454, 1410, 1310, 1254, 1139, 1088, 1023, 978, 872, 835, 802, 760, 700. HRMS m/z: calcd for $[C_{16}H_{18}FNO_2S+Na]^+$: 330.0934, found: 330.0933.

[(2-Fluoro-2-phenylethyl)imino](methyl)(4-nitrophenyl)-λ^{6}-sulfanone (7ga)

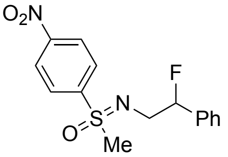

Following the general procedure afforded the product as a yellow viscous oil (41 mg, 64% yield). dr = 1 : 1. ^{1}H NMR (600 MHz, $CDCl_3$, mixture of diastereomers) δ 8.38 – 8.35 (m, 2H), 8.33 – 8.30 (m, 2H), 8.06 (d, J = 8.8 Hz, 2H), 7.94 – 7.91 (m, 2H), 7.38 – 7.26 (m, 10H), 5.64 – 5.44 (m, 2H), 3.50 (ddd, J = 16.3, 13.3, 7.0 Hz, 1H), 3.35 (ddd, J = 32.5, 13.9, 2.8 Hz, 1H), 3.18 – 3.09 (m, 8H). ^{13}C {^{1}H} (151 MHz, $CDCl_3$, mixture of diastereomers) δ 150.5, 150.4, 145.6, 145.5, 138.3 (d, J_{FC} = 19.7 Hz), 138.0 (d, J_{FC} = 19.7 Hz), 130.2, 129.9, 128.6, 128.5, 128.4, 128.4, 126.0 (d, J_{FC} = 6.7 Hz), 125.5 (d, J_{FC} = 7.1 Hz), 124.6, 124.5, 95.0 (d, J_{FC} = 175.0 Hz), 94.2 (d, J_{FC} = 173.7 Hz), 49.7 (d, J_{FC} = 23.9 Hz), 49.4 (d, J_{FC} = 28.3 Hz), 45.1, 44.8. ^{19}F NMR (564 MHz, $CDCl_3$, mixture of diastereomers) δ –179.18 – –179.38 (m), –181.30 (ddd, J = 48.0, 32.5, 19.0 Hz). MS (EI): m/z (%) = 213 (42), 186 (34), 140 (11), 109 (100), 107 (8), 83 (18), 77 (3), 63 (5). IR (ATR): ν = 3101, 3031, 2927, 2848, 2163, 1604, 1527, 1453, 1347, 1314, 1260, 1233, 1144, 1083, 1030, 980, 855, 768, 740, 699 cm^{-1}. HRMS m/z: calcd for $[C_{15}H_{15}FN_2O_3S+Na]^+$: 345.0680, found: 345.0683.

(4-Acetylphenyl)[(2-fluoro-2-phenylethyl)imino](methyl)-λ^{6}-sulfanone (7ha)

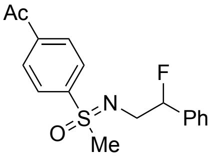

Following the general procedure afforded the product as a yellow viscous oil (35 mg, 55% yield). dr = 1 : 1. ^{1}H NMR (600 MHz, $CDCl_3$, mixture of diastereomers) δ 8.10 – 8.07 (m, 2H), 8.06 – 8.03 (m, 2H), 7.98 – 7.95 (m, 2H), 7.88 – 7.85 (m, 2H), 7.37 – 7.27 (m, 10H), 5.63 – 5.44 (m, 2H), 3.50 – 3.43 (m, 1H), 3.36 – 3.27 (m, 1H), 3.19 – 3.08 (m, 8H), 2.65 (d, J = 1.2 Hz, 3H), 2.65 (d, J = 1.1 Hz, 3H). ^{13}C {^{1}H} (151 MHz, $CDCl_3$, mixture of diastereomers) δ 196.9, 196.9, 143.4, 143.3, 140.4, 140.3, 138.4 (d, J_{FC} = 19.8 Hz), 138.3 (d, J_{FC} = 19.7 Hz), 129.2, 129.2, 128.9, 128.5, 128.4, 128.3, 128.3, 126.0 (d, J_{FC} = 6.7 Hz), 125.6 (d, J_{FC} = 6.7 Hz), 95.3 (d, J_{FC} = 174.4 Hz), 94.5 (d, J_{FC} = 174.1 Hz), 49.8 (d, J_{FC} = 23.3 Hz), 49.6 (d, J_{FC} = 23.3 Hz), 44.9, 27.0. ^{19}F NMR (564 MHz, $CDCl_3$, mixture of diastereomers) δ –179.23 (dddd, J = 47.5, 25.1, 16.4, 3.4 Hz), –181.22 (ddd, J = 48.3, 32.8, 18.5 Hz). MS (EI): m/z (%) = 320 (20), 300 (7), 210 (100), 183 (54), 166 (20), 151 (13), 109 (43), 90 (6), 77 (6). IR (ATR): ν = 3364, 3031, 2926, 2844, 2172, 1688, 1593, 1450, 1396, 1359, 1256, 1144, 1089, 1026, 959, 837, 780, 754, 700 cm^{-1}. HRMS m/z: calcd for $[C_{17}H_{18}FNO_2S+Na]^+$: 342.0934, found: 342.0933.

4-[*N*-(2-Fluoro-2-phenylethyl)-*S*-methylsulfonimidoyl]benzonitrile (7ia)

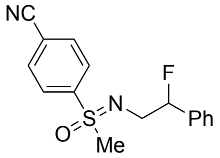

Following the general procedure afforded the product as a yellow viscous oil (41 mg, 68% yield). dr = 1 : 1. ^{1}H NMR (600 MHz, $CDCl_3$, mixture of diastereomers) δ 7.99 – 7.95 (m, 2H), 7.86 – 7.75 (m, 6H), 7.37 – 7.24 (m, 10H), 5.61 – 5.43 (m, 2H), 3.47 (ddd, J = 16.2, 13.3, 7.0 Hz, 1H), 3.32 (ddd, J = 32.4, 13.9, 2.8 Hz, 1H), 3.15 – 3.06 (m, 8H). ^{13}C {^{1}H} (151 MHz, $CDCl_3$, mixture of diastereomers) δ 144.0, 143.9, 138.4 (d, J_{FC} = 19.6 Hz), 138.1 (d, J_{FC} = 19.7 Hz), 133.2, 133.1, 129.5, 129.3, 128.5, 128.5, 128.5, 128.4, 128.4, 126.0 (d, J_{FC} = 6.7 Hz), 125.6 (d, J_{FC} = 6.9 Hz), 117.1 (d, J_{FC} = 81.9 Hz), 117.0 (d, J_{FC} = 87.3 Hz), 95.1 (d, J_{FC} = 175.0 Hz), 94.3 (d, J_{FC} = 174.2 Hz), 49.7 (d, J_{FC} = 23.6 Hz), 49.4 (d, J_{FC} = 28.0 Hz), 45.1, 44.8. ^{19}F NMR (564 MHz, $CDCl_3$, mixture of diastereomers) δ –179.23 (dddd, J = 45.7, 21.8, 15.9, 5.3 Hz), –181.26 (dddd, J = 50.8, 32.3, 19.0, 4.0 Hz). MS (EI): *m/z* (%) = 303 (6), 195 (6), 193 (100), 166 (65), 150 (13), 121 (8), 109 (40), 83 (11), 77 (3), 63 (5). IR (ATR): ν = 3033, 2927, 2845, 2233, 2160, 1491, 1453, 1395, 1316, 1261, 1232, 1143, 1081, 1029, 980, 840, 789, 756, 700 cm^{-1}. HRMS *m/z*: calcd for $[C_{16}H_{15}FN_2OS+Na]^+$: 325.0781, found: 325.0782.

(3-Chlorophenyl)[(2-fluoro-2-phenylethyl)imino](methyl)-λ^{6}-sulfanone (7ja)

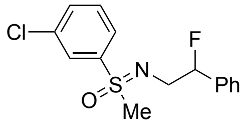

Following the general procedure afforded the product as a yellow viscous oil (43 mg, 69% yield). dr = 1 : 1. ^{1}H NMR (600 MHz, $CDCl_3$, mixture of diastereomers) ^{1}H NMR (600 MHz, $CDCl_3$, mixture of diastereomers) δ 7.88 (t, J = 1.9 Hz, 1H), 7.80 (t, J = 1.9 Hz, 1H), 7.74 (dt, J = 7.7, 1.3 Hz, 1H), 7.64 (dt, J = 7.9, 1.3 Hz, 1H), 7.56 (tdd, J = 8.2, 2.1, 1.1 Hz, 2H), 7.46 (dt, J = 23.1, 7.9 Hz, 2H), 7.37-7.28 (m, 10H), 5.63 – 5.44 (m, 2H), 3.47 (ddd, J = 16.2, 13.3, 7.3 Hz, 1H), 3.32 (ddd, J = 32.0, 13.8, 3.0 Hz, 1H), 3.23 – 3.12 (m, 2H), 3.11 (s, 3H), 3.08 (s, 3H). ^{13}C {^{1}H} (151 MHz, $CDCl_3$, mixture of diastereomers) δ 141.3, 141.1, 138.5 (d, J_{FC} = 19.5 Hz), 138.3 (d, J_{FC} = 19.8 Hz), 135.7, 135.6, 133.1, 133.1, 130.7, 130.6, 128.9, 128.6, 128.4, 128.4, 128.3, 128.3, 126.7, 126.5, 126.0 (d, J_{FC} = 6.0 Hz), 125.6 (d, J_{FC} = 6.9 Hz), 95.3 (d, J_{FC} = 175.8 Hz), 94.5 (d, J_{FC} = 174.2 Hz), 49.6 (d, J_{FC} = 41.2 Hz), 49.5 (d, J_{FC} = 44.4 Hz), 45.2, 45.0. ^{19}F NMR (565 MHz, $CDCl_3$, mixture of diastereomers) δ –179.00 (ddd, J = 47.3, 24.9, 16.1 Hz), –181.06 (ddd, J = 49.2, 32.0, 18.3 Hz). MS (EI): *m/z* (%) = 177 (24), 175 (64), 159 (17), 131 (8), 111 (10), 109 (52), 83 (14), 77 (5), 75 (13), 63 (9). IR (ATR): ν = 3065, 2926, 2843, 2326, 1575, 1456, 1408, 1315, 1332, 1144, 1074, 980, 872, 785, 750, 699, 679 cm^{-1}. HRMS *m/z*: calcd for $[C_{15}H_{15}ClFNOS+Na]^+$: 334.0439, found: 334.0442.

(3-Bromophenyl)[(2-fluoro-2-phenylethyl)imino](methyl)-λ^{6}-sulfanone (7ka)

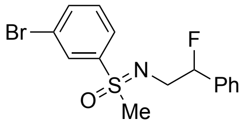

Following the general procedure afforded the product as a yellow viscous oil (47 mg, 66% yield). *dr* = 1 : 1.2. ^{1}H NMR (600 MHz, $CDCl_3$, mixture of diastereomers) δ 8.04 (t, J = 1.9 Hz, 1.2H), 7.97 (t, J = 1.9 Hz, 1H), 7.79 (dt, J = 7.9, 1.3 Hz, 1.2H), 7.74 – 7.66 (m, 3.2H), 7.43 – 7.37 (m, 2.2H), 7.36 – 7.29 (m, 11.2H), 5.63 – 5.44 (m, 2.2H), 3.47 (ddd, J = 16.2, 13.3, 7.3 Hz, 1H), 3.32 (ddd, J = 32.1, 13.8, 3.0 Hz, 1.2H), 3.22 – 3.13 (m, 2.2H), 3.11 (s, 3.6H), 3.08 (s, 3H). ^{13}C {^{1}H} (151 MHz, $CDCl_3$, mixture of diastereomers) δ 141.4, 141.3, 138.5 (d, J_{FC} = 19.7 Hz), 138.3 (d, J_{FC} = 19.5 Hz), 136.1, 136.0, 131.8, 131.5, 130.9, 130.9, 128.4, 128.4, 128.3, 128.3, 127.2, 127.0, 126.0 (d, J_{FC} = 6.6 Hz), 125.6 (d, J_{FC} = 7.1 Hz), 123.5, 123.4, 95.3 (d, J_{FC} = 175.4 Hz), 94.4 (d, J_{FC} =

174.4 Hz), 49.7 (d, J_{FC} = 24.3 Hz), 49.4 (d, J_{FC} = 27.7 Hz), 45.3, 45.0. ^{19}F NMR (565 MHz, $CDCl_3$, mixture of diastereomers) δ –179.02 (ddd, J = 47.3, 25.0, 16.1 Hz), –181.04 (ddd, J = 49.3, 32.1, 18.7 Hz). MS (EI): *m/z* (%) = 358 (6), 356 (8), 248 (64), 246 (100), 221 (50), 219 (55), 205 (16), 202 (12), 156 (8), 139 (9), 109 (84), 83 (22), 77 (11), 63 (13), 50 (12). IR (ATR): ν = 3064, 2925, 2329, 1727, 1568, 1455, 1405, 1232, 1143, 1096, 1070, 979, 872, 775, 699, 678 cm^{-1}. HRMS *m/z*: calcd for $[C_{15}H_{15}BrFNOS+Na]^+$: 377.9934, found: 377.9939.

[(2-Fluoro-2-phenylethyl)imino](methyl)(*m*-tolyl)-λ^6-sulfanone (7la)

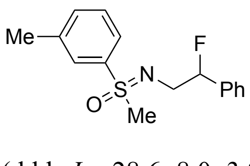

Following the general procedure afforded the product as a yellow viscous oil (48 mg, 83% yield). dr = 1 : 1. ^{1}H NMR (400 MHz, $CDCl_3$, mixture of diastereomers) δ 7.71 – 7.65 (m, 2H), 7.64 – 7.55 (m, 2H), 7.45 – 7.36 (m, 4H), 7.35 – 7.27 (m, 10H), 5.61 (ddd, J = 29.3, 8.0, 3.9 Hz, 1H), 5.49 (ddd, J = 28.6, 8.0, 3.9 Hz, 1H), 3.46 (ddd, J = 15.8, 13.2, 7.4 Hz, 1H), 3.37 – 3.09 (m, 7H), 3.08 (s, 3H), 2.42 (s, 3H), 2.40 (s, 3H). ^{13}C {^{1}H} (101 MHz, $CDCl_3$, mixture of diastereomers) δ 139.7, 139.7, 138.8 (d, J_{FC} = 16.8 Hz), 138.4 (d, J_{FC} = 18.9 Hz), 133.8, 133.8, 129.3, 129.3, 129.2, 128.9, 128.3, 128.3, 126.0, 125.9, 125.8, 125.7, 125.6, 125.6, 95.6 (d, J_{FC} = 174.5 Hz), 94.6 (d, J_{FC} = 174.5 Hz), 49.8 (d, J_{FC} = 24.2 Hz), 49.6 (d, J_{FC} = 26.9 Hz), 45.3, 45.0, 21.4. ^{19}F NMR (376 MHz, $CDCl_3$, mixture of diastereomers) δ –179.13 (ddd, J = 47.2, 25.6, 15.7 Hz), –181.14 (ddd, J = 50.0, 32.3, 18.4 Hz). MS (EI): *m/z* (%) = 182 (47), 155 (57), 139 (21), 109 (100), 91 (15), 83 (23), 77 (5), 63 (8). IR (ATR): ν = 3400, 3030, 2925, 2842, 2327, 1598, 1474, 1453, 1410, 1313, 1229, 1141, 1087, 1034, 979, 871, 759, 695 cm^{-1}. HRMS *m/z*: calcd for $[C_{16}H_{18}FNOS+Na]^+$: 314.0985, found: 314.0983.

[(2-Fluoro-2-phenylethyl)imino](3-methoxyphenyl)(methyl)-λ^6-sulfanone (7ma)

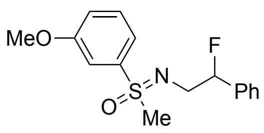

Following the general procedure afforded the product as a yellow viscous oil (52 mg, 85% yield). dr = 1 : 1. ^{1}H NMR (400 MHz, $CDCl_3$, mixture of diastereomers) δ 7.47 – 7.37 (m, 5H), 7.36 – 7.28 (m, 11H), 7.16 – 7.08 (m, 2H), 5.61 (ddd, J = 34.5, 8.1, 3.8 Hz, 1H), 5.49 (ddd, J = 33.8, 8.1, 3.9 Hz, 1H), 3.86 (s, 3H), 3.84 (s, 3H), 3.46 (ddd, J = 16.5, 13.4, 7.6 Hz, 1H), 3.38 – 3.27 (m, 1H), 3.26 – 3.11 (m, 5H), 3.10 (s, 3H). ^{13}C {^{1}H} (101 MHz, $CDCl_3$, mixture of diastereomers) δ 160.3, 160.3, 140.3 (d, J_{FC} = 30.6 Hz), 138.7, 138.5, 138.3, 130.5, 130.4, 128.3, 128.3, 128.2, 126.0 (d, J_{FC} = 6.8 Hz), 125.7 (d, J_{FC} = 6.9 Hz), 120.7 (d, J_{FC} = 28.9 Hz), 119.7 (d, J_{FC} = 28.9 Hz), 113.1, 113.0, 95.5 (d, J_{FC} = 174.8 Hz), 94.7 (d, J_{FC} = 173.9 Hz), 55.6, 49.9 (d, J_{FC} = 23.7 Hz), 49.6 (d, J_{FC} = 26.8 Hz), 45.3, 45.1. ^{19}F NMR (376 MHz, $CDCl_3$, mixture of diastereomers) δ –179.18 (ddd, J = 47.8, 26.4, 16.5 Hz), –180.93 (ddd, J = 50.1, 33.0, 18.2 Hz). MS (EI): *m/z* (%) = 308 (20), 198 (100), 171 (48), 155 (18), 124 (7), 109 (36), 107 (14), 77 (11), 59 (7). IR (ATR): ν = 3012, 2931, 2840, 2323, 1593, 1477, 1427, 1317, 1237, 1141, 1033, 978, 867, 760, 696 cm^{-1}. HRMS *m/z*: calcd for $[C_{16}H_{18}FNO_2S+Na]^+$: 330.0934, found: 330.0934.

(2-Chlorophenyl)[(2-fluoro-2-phenylethyl)imino](methyl)-λ^6-sulfanone (7na)

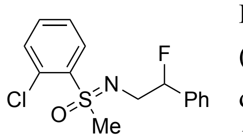

Following the general procedure afforded the product as a yellow viscous oil (53 mg, 76% yield). dr = 1 : 1. ^{1}H NMR (400 MHz, $CDCl_3$, mixture of diastereomers) δ 8.20 – 8.11 (m, 2H), 7.57 – 7.41 (m, 6H), 7.35 – 7.26 (m, 10H), 5.58 (ddd, J = 12.2, 8.2, 3.7 Hz, 1H), 5.46 (ddd, J = 12.1, 7.8, 4.1 Hz,

1H), 3.40 – 3.31 (m, 7H), 3.28 – 3.17 (m, 2H), 3.07 (ddd, J = 30.3, 13.6, 3.8 Hz, 1H). $^{13}C\{^1H\}$ (101 MHz, $CDCl_3$, mixture of diastereomers) δ 138.5 (d, J_{FC} = 4.1 Hz), 138.3 (d, J_{FC} = 4.1 Hz), 136.6, 136.1, 134.1, 134.1, 133.2, 132.8, 132.5, 132.3, 132.0, 131.9, 128.3, 128.3, 128.2, 127.6, 127.5, 125.8 (d, J_{FC} = 6.8 Hz), 125.7 (d, J_{FC} = 6.8 Hz), 95.2 (d, J_{FC} = 175.0 Hz), 94.7 (d, J_{FC} = 175.1 Hz), 50.3 (d, J_{FC} = 25.9 Hz), 49.9 (d, J_{FC} = 25.0 Hz), 43.2, 43.1. ^{19}F NMR (376 MHz, $CDCl_3$, mixture of diastereomers) δ –178.71 (ddd, J = 47.1, 30.2, 16.6 Hz), –179.83 (ddd, J = 48.7, 30.3, 18.9 Hz). MS (EI): m/z (%) = 312 (16), 204 (30), 202 (100), 177 (27), 175 (72), 159 (16), 131 (9), 109 (57), 83 (16), 77 (5), 75 (11). IR (ATR): v = 3064, 2928, 2348, 1573, 1448, 1313, 1239, 1145, 1037, 977, 871, 756, 699 cm^{-1}. HRMS m/z: calcd for $[C_{15}H_{15}ClFNOS+Na]^+$: 334.0439, found: 334.0441.

[(2-Fluoro-2-phenylethyl)imino](2-methoxyphenyl)(methyl)-λ⁶-sulfanone (7oa)

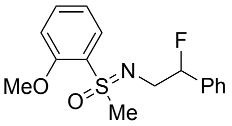

Following the general procedure afforded the product as a yellow viscous oil (43 mg, 70% yield). dr = 1 : 1. ^1H NMR (400 MHz, $CDCl_3$, mixture of diastereomers) δ 7.82 – 7.78 (m, 2H), 7.72 – 7.68 (m, 2H), 7.35 – 7.28 (m, 10H), 7.02 – 6.94 (m, 4H), 5.61 (ddd, J = 33.2, 8.1, 3.9 Hz, 1H), 5.49 (ddd, J = 32.4, 8.0, 3.9 Hz, 1H), 3.87 (d, J = 2.5 Hz, 6H), 3.45 (ddd, J = 15.9, 13.3, 7.5 Hz, 1H), 3.35 – 3.06 (m, 9H). $^{13}C\{^1H\}$ (101 MHz, $CDCl_3$, mixture of diastereomers) δ 163.2, 138.6, 138.4, 131.0, 130.7, 130.2, 130.0, 128.3, 126.0 (d, J_{FC} = 6.9 Hz), 125.7 (d, J_{FC} = 6.8 Hz), 114.7, 114.6, 95.4 (d, J_{FC} = 175.0 Hz), 94.7 (d, J_{FC} = 174.0 Hz), 55.6, 49.9 (d, J_{FC} = 23.5 Hz), 49.6 (d, J_{FC} = 25.4 Hz), 45.7, 45.4. ^{19}F NMR (376 MHz, $CDCl_3$, mixture of diastereomers) δ –179.19 (ddd, J = 47.5, 25.8, 15.7 Hz), –181.19 (ddd, J = 50.2, 33.0, 18.5 Hz). MS (EI): m/z (%) = 198 (100), 171 (60), 155 (56), 154 (20), 123 (6), 109 (36), 92 (9), 77 (9), 63 (6). IR (ATR): v = 3027, 2929, 2841, 2166, 1590, 1494, 1455, 1309, 1254, 1138, 1088, 1023, 978, 835, 802, 760, 700. HRMS m/z: calcd for $[C_{16}H_{18}FNO_2S+Na]^+$: 330.0934, found: 330.0934.

[(2-Fluoro-2-phenylethyl)imino](methyl)(naphthalen-2-yl)-λ⁶-sulfanone (7pa)

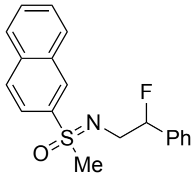

Following the general procedure afforded the product as a yellow viscous oil (57 mg, 87% yield). dr = 1 : 1. ^1H NMR (600 MHz, $CDCl_3$, mixture of diastereomers) δ 8.48 (d, J = 1.8 Hz, 1H), 8.41 (d, J = 1.9 Hz, 1H), 8.01 – 7.90 (m, 6H), 7.83 (dd, J = 8.6, 1.9 Hz, 1H), 7.71 (dd, J = 8.7, 1.9 Hz, 1H), 7.68 – 7.59 (m, 4H), 7.36 – 7.26 (m, 9H), 5.68 – 5.49 (m, 2H), 3.50 (ddd, J = 15.9, 13.4, 7.5 Hz, 1H), 3.34 (ddd, J = 32.8, 13.8, 2.9 Hz, 1H), 3.26 – 3.12 (m, 8H). $^{13}C\{^1H\}$ (151 MHz, $CDCl_3$, mixture of diastereomers) δ 138.6 (d, J_{FC} = 19.7 Hz), 138.4 (d, J_{FC} = 19.8 Hz), 135.9, 135.8, 135.1, 135.0, 132.6, 132.5, 130.7, 130.5, 129.8, 129.7, 129.3, 129.0, 128.4, 128.4, 128.3, 128.3, 127.9, 127.6, 127.5, 126.0 (d, J_{FC} = 6.8 Hz), 125.7 (d, J_{FC} = 7.0 Hz), 123.5, 123.2, 95.3 (d, J_{FC} = 174.8 Hz), 94.7 (d, J_{FC} = 174.9 Hz), 50.0 (d, J_{FC} = 23.7 Hz), 49.8 (d, J_{FC} = 27.1 Hz), 45.3, 45.1. ^{19}F NMR (564 MHz, $CDCl_3$, mixture of diastereomers) δ –178.93 – –179.15 (m), –181.02 (dddd, J = 47.5, 32.3, 18.6, 5.5 Hz). MS (EI): m/z (%) = 218 (100), 191 (36), 175 (28), 174 (10), 147 (8), 127 (14), 109 (24), 83 (5), 77 (3). IR (ATR): v = 3031, 2925, 2842, 2325, 2079, 1595, 1493, 1451, 1405, 1314, 1228, 1142, 1086, 1033, 979, 872, 815, 759, 699 cm^{-1}. HRMS m/z: calcd for $[C_{19}H_{18}FNOS+Na]^+$: 350.0985, found: 350.0984.

[(2-Fluoro-2-phenylethyl)imino]diphenyl-λ^6-sulfanone (7qa)

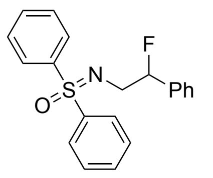

Following the general procedure afforded the product as a yellow viscous oil (57 mg, 84% yield). ^{1}H NMR (600 MHz, $CDCl_3$) δ 7.94 – 7.86 (m, 4H), 7.52 – 7.48 (m, 2H), 7.47 – 7.42 (m, 4H), 7.39 – 7.31 (m, 4H), 5.69 (ddd, J = 47.6, 7.5, 4.3 Hz, 1H), 3.52 – 3.36 (m, 2H). ^{13}C {^{1}H} (151 MHz, $CDCl_3$) δ 140.3, 140.1, 138.8 (d, J_{FC} = 19.7 Hz), 133.1, 132.5, 132.5, 131.0, 129.4, 129.2, 129.1, 128.7, 128.5, 128.5, 128.2, 127.6, 125.9 (d, J_{FC} = 7.0 Hz), 124.7, 123.5, 122.4, 95.1 (d, J_{FC} = 175.3 Hz), 49.7 (d, J_{FC} = 26.0 Hz). ^{19}F NMR (564 MHz, $CDCl_3$) δ –180.68 (ddd, J = 47.7, 26.7, 17.2 Hz). MS (EI): *m/z* (%) = 340 (6), 230 (100), 203 (60), 186 (16), 154 (9), 125 (9), 109 (37), 77 (10), 51 (8). IR (ATR): v = 3063, 2900, 2844, 2165, 1475, 1446, 1261, 1145, 1089, 1025, 909, 873, 829, 755, 726, 690 cm^{-1}. HRMS *m/z*: calcd for $[C_{20}H_{18}FNOS+Na]^+$: 362.0985, found: 362.0986.

[(2-Fluoro-2-phenylethyl)imino]bis(4-methoxyphenyl)-λ^6-sulfanone (7ra)

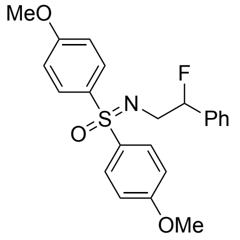

Following the general procedure afforded the product as a yellow viscous oil (68 mg, 85% yield). ^{1}H NMR (600 MHz, $CDCl_3$) δ 7.83 – 7.74 (m, 4H), 7.40 – 7.28 (m, 5H), 6.92 – 6.86 (m, 4H), 5.67 (ddd, J = 47.6, 7.5, 4.2 Hz, 1H), 3.81 – 3.80 (m, 6H), 3.49 – 3.31 (m, 2H). ^{13}C {^{1}H} (151 MHz, $CDCl_3$) δ 162.7, 162.7, 139.0 (d, J_{FC} = 19.5 Hz), 132.3, 132.0, 130.6, 130.4, 128.3, 128.2, 126.0 (d, J_{FC} = 7.3 Hz), 114.4, 114.3, 95.1 (d, J_{FC} = 175.7 Hz), 55.6, 49.8 (d, J_{FC} = 26.2 Hz). ^{19}F NMR (564 MHz, $CDCl_3$) δ –180.63 (ddd, J = 47.7, 27.1, 17.1 Hz). MS (EI): *m/z* (%) = 291 (13), 290 (100), 263 (47), 246 (14), 214 (17), 199 (11), 155 (8), 123 (5), 109 (20), 77 (2). IR (ATR): v = 2932, 2842, 2164, 1586, 1492, 1455, 1309, 1250, 1140, 1094, 1018, 911, 835, 803, 758, 682. HRMS *m/z*: calcd for $[C_{22}H_{22}FNO_3S+Na]^+$: 422.1197, found: 422.1193.

5-[(2-Fluoro-2-phenylethyl)imino]-3,5-dihydro-2*H*-5λ^4-dibenzo[*b*,*d*]thiophene 5-oxide (7sa)

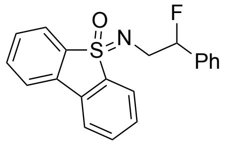

Following the general procedure afforded the product as a yellow viscous oil (57 mg, 85% yield). ^{1}H NMR (600 MHz, $CDCl_3$) δ 7.80 – 7.72 (m, 4H), 7.59 (qd, J = 7.6, 1.2 Hz, 2H), 7.48 (dtd, J = 8.6, 7.6, 1.1 Hz, 2H), 7.38 – 7.31 (m, 5H), 5.59 (ddd, J = 47.9, 8.0, 3.7 Hz, 1H), 3.72 (ddd, J = 17.1, 13.5, 8.1 Hz, 1H), 3.57 (ddd, J = 29.8, 13.5, 3.7 Hz, 1H). ^{13}C {^{1}H} (151 MHz, $CDCl_3$) δ 138.9 (d, J_{FC} = 81.2 Hz), 138.3 (d, J_{FC} = 19.3 Hz), 133.2, 133.0, 132.2, 131.9, 130.2, 130.1, 128.5, 128.4, 125.8 (d, J_{FC} = 6.8 Hz), 122.7, 122.5, 121.6, 121.6, 94.9 (d, J_{FC} = 175.8 Hz), 50.2 (d, J_{FC} = 26.2 Hz). ^{19}F NMR (564 MHz, $CDCl_3$) δ –179.50 (ddd, J = 47.5, 29.8, 17.1 Hz). MS (EI): *m/z* (%) = 229 (11), 228 (72), 201 (56), 200 (12), 184 (35), 172 (23), 171 (44), 139 (9), 109 (100), 83 (18), 77 (6). IR (ATR): v = 3062, 2904, 2844, 1694, 1479, 1446, 1232, 1149, 1066, 1030, 908, 871, 828, 754, 702 cm^{-1}. HRMS *m/z*: calcd for $[C_{20}H_{18}FNOS+Na]^+$: 362.0985, found: 362.0983.

[(2-Fluoro-2-phenylethyl)imino](methyl)(pyridin-2-yl)-λ^6-sulfanone (7ta)

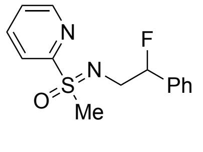

Following the general procedure afforded the product as a yellow viscous oil (45 mg, 81% yield). dr = 1 : 1. ^{1}H NMR (600 MHz, $CDCl_3$, mixture of diastereomers) δ 8.76 – 8.70 (m, 2H), 8.08 (ddt, J = 14.9, 7.8, 1.1 Hz, 2H), 7.91 (td, J = 7.7, 1.7 Hz, 2H), 7.48 (ddt, J = 7.6, 4.7, 1.0 Hz, 2H), 7.33 – 7.26 (m, 10H), 5.56 – 5.44 (m, 2H), 3.52 – 3.43 (m, 1H), 3.38 – 3.28 (m, 3H), 3.27 (s, 3H), 3.26 (s, 3H),

3.19 – 3.10 (m, 2H). ^{13}C {^{1}H} (151 MHz, $CDCl_3$, mixture of diastereomers) δ 157.9, 157.6, 150.4, 150.3, 138.4 (d, J_{FC} = 6.9 Hz), 138.3 (d, J_{FC} = 6.9 Hz), 137.9, 137.8, 128.4, 128.3, 128.3, 126.6, 126.6, 125.7 (d, J_{FC} = 6.8 Hz), 125.6 (d, J_{FC} = 6.8 Hz), 123.2, 122.9, 122.9, 95.1 (d, J_{FC} = 174.2 Hz), 94.7 (d, J_{FC} = 173.7 Hz), 49.7 (d, J_{FC} = 25.7 Hz), 49.6 (d, J_{FC} = 25.0 Hz), 41.4, 41.4. ^{19}F NMR (564 MHz, $CDCl_3$, mixture of diastereomers) δ –179.14 (ddd, J = 47.7, 30.4, 17.1 Hz), –179.76 (ddd, J = 48.7, 30.6, 18.5 Hz). MS (EI): *m/z* (%) = 279 (10), 169 (100), 142 (14), 124 (33), 109 (36), 83 (10), 77(2), 51 (12). IR (ATR): *v* = 3036, 2927, 2843, 2197, 1574, 1495, 1451, 1424, 1309, 1230, 1149, 1119, 1036, 982, 870, 758, 700 cm^{-1}. HRMS *m/z*: calcd for $[C_{14}H_{15}FN_2OS+Na]^+$: 301.0781, found: 301.0780.

[(2-Fluoro-2-(4-fluorophenyl)ethyl]imino)(4-methoxyphenyl)(methyl)-λ^6-sulfanone (7fb)

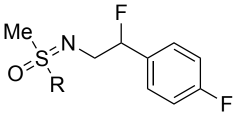

R = 4-MeO-Ph

Following the general procedure afforded the product as a yellow viscous oil (50 mg, 78% yield). dr = 1 : 1.4. ^{1}H NMR (600 MHz, $CDCl_3$, mixture of diastereomers) δ 7.81 – 7.78 (m, 2.8H), 7.72 – 7.68 (m, 2H), 7.34 – 7.30 (m, 2H), 7.30 – 7.27 (m, 2.8H), 7.05 – 6.96 (m, 10.8H), 5.61 – 5.42 (m, 2.6H), 3.87 (s, 4.2H), 3.87 (s, 3H), 3.43 (ddd, J = 15.6, 13.3, 7.1 Hz, 1H), 3.29 – 3.11 (m, 3.8H), 3.10 (s, 4.2H).3.07 (s, 3H). ^{13}C {^{1}H} (151 MHz, $CDCl_3$, mixture of diastereomers) δ 163.5, 163.5, 163.4, 163.3, 163.3, 161.8, 161.8, 134.6 (dd, J_{FC} = 20.6, 3.4 Hz), 134.4 (dd, J_{FC} = 20.6, 3.4 Hz), 130.9, 130.7, 130.1, 129.9, 128.0, 127.9, 127.9, 127.6, 127.6, 127.5, 115.2 (d, J_{FC} = 21.2 Hz), 115.1 (d, J_{FC} = 21.2 Hz), 114.7, 114.6, 94.8 (d, J_{FC} = 175.3 Hz), 94.0 (d, J_{FC} = 174.3 Hz), 55.6, 49.7 (d, J_{FC} = 24.5 Hz), 49.4 (d, J_{FC} = 27.8 Hz), 45.7, 45.4. ^{19}F NMR (564 MHz, $CDCl_3$, mixture of diastereomers) δ –113.68 (ddd, J = 12.1, 8.0, 4.1 Hz), –113.77 (dh, J = 9.9, 5.2 Hz), –176.91 (dddd, J = 47.3, 23.4, 15.5, 2.7 Hz), –178.89 (ddd, J = 48.7, 31.7, 18.2 Hz). MS (EI): *m/z* (%) = 199 (10), 198 (100), 171 (53), 155 (47), 154 (15), 127 (28), 108 (5), 77 (6). IR (ATR): *v* = 3031, 2832, 2842, 2167, 1593, 1498, 1462, 1411, 1353, 1309, 1254, 1224, 1139, 1088, 1022, 979, 873, 833, 804, 764, 659. HRMS *m/z*: calcd for $[C_{16}H_{17}F_2NO_2S+Na]^+$: 348.0840, found: 348.0833.

{[2-(4-Chlorophenyl)-2-fluoroethyl]imino}(4-methoxyphenyl)(methyl)-λ^6-sulfanone (7fc)

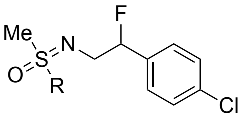

R = 4-MeO-Ph

Following the general procedure afforded the product as a yellow viscous oil (55 mg, 80% yield). dr = 1 : 1.^{1}H NMR (600 MHz, $CDCl_3$, mixture of diastereomers) δ 7.79 – 7.76 (m, 2H), 7.70 – 7.66 (m, 2H), 7.33 – 7.23 (m, 8H), 7.02 – 6.95 (m, 4H), 5.60 – 5.41 (m, 2H), 3.87 (s, 3H), 3.87 (s, 3H), 3.42 (ddd, J = 15.6, 13.2, 6.8 Hz, 1H), 3.26 (ddd, J = 31.3, 13.8, 3.2 Hz, 1H), 3.18 – 3.06 (m, 5H), 3.06 (s, 3H). ^{13}C {^{1}H} (151 MHz, $CDCl_3$, mixture of diastereomers) δ 163.3, 163.3, 137.3 (d, J_{FC} = 20.0 Hz), 137.1 (d, J_{FC} = 20.0 Hz), 134.1, 130.9, 130.6, 130.1, 129.9, 129.8, 128.7, 128.4, 128.4, 127.5 (d, J_{FC} = 6.7 Hz), 127.1 (d, J_{FC} = 6.7 Hz), 114.7, 114.6, 114.4, 94.8 (d, J_{FC} = 175.4 Hz), 93.8 (d, J_{FC} = 174.5 Hz), 55.6, 49.6 (d, J_{FC} = 24.3 Hz) 49.4 (d, J_{FC} = 27.8 Hz), 45.7, 45.4. ^{19}F NMR (564 MHz, $CDCl_3$, mixture of diastereomers) δ –178.91 (ddd, J = 47.0, 22.5, 15.8 Hz), –181.14 (ddd, J = 49.0, 31.4, 18.8 Hz). MS (EI): *m/z* (%) = 199 (10), 198 (100), 171 (45), 155 (43), 143 (16), 139 (5), 108 (6), 77 (5). IR (ATR): *v* = 3011, 2930, 2841, 1904, 1591, 1492, 1463, 1409, 1310, 1254, 1139, 1088, 1018, 979, 872, 829, 804, 767, 732 cm^{-1}. HRMS *m/z*: calcd for $[C_{16}H_{17}ClFNO_2S+H]^+$: 342.0725, found: 342.0723.

{[2-(4-Bromophenyl)-2-fluoroethyl]imino}(4-methoxyphenyl)(methyl)-λ^6-sulfanone (7fd)

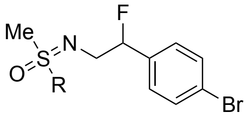

R = 4-MeO-Ph

Following the general procedure afforded the product as a yellow viscous oil (63 mg, 81% yield). dr = 1 : 1.4.^{1}H NMR (600 MHz, $CDCl_3$, mixture of diastereomers) *δ* 7.78 – 7.74 (m, 2H), 7.68 – 7.64 (m, 3H), 7.48 – 7.45 (m, 3H), 7.45 – 7.42 (m, 2H), 7.21 (d, *J* = 8.3 Hz, 3H), 7.17 (d, *J* = 8.4 Hz, 2H), 7.01 – 6.97 (m, 2H), 6.97 – 6.94 (m, 3H), 5.58 – 5.38 (m, 2.68H), 3.86 (s, 3.16H), 3.86 (s, 3.97H), 3.41 (ddd, *J* = 15.7, 13.2, 6.9 Hz, 1.75H), 3.30 – 3.21 (m, 1H), 3.16 – 3.06 (m, 6.24H), 3.05 (s, 4.43H). ^{13}C {^{1}H} (151 MHz, $CDCl_3$, mixture of diastereomers) *δ* 163.3, 163.2, 137.8 (d, J_{FC} = 19.8 Hz), 137.6 (d, J_{FC} = 20.1 Hz), 131.3, 131.3, 130.8, 130.6, 130.3, 130.0, 129.8, 127.8 (d, J_{FC} = 7.0 Hz), 127.4 (d, J_{FC} = 7.0 Hz), 122.3 (d, J_{FC} = 2.5 Hz), 122.2 (d, J_{FC} = 2.5 Hz), 114.7, 114.6, 94.7 (d, J_{FC} = 175.0 Hz), 93.6 (d, J_{FC} = 175.0 Hz), 55.6, 49.6 (d, J_{FC} = 20.1 Hz), 49.3 (d, J_{FC} = 27.8 Hz), 45.6, 45.4. ^{19}F NMR (564 MHz, $CDCl_3$, mixture of diastereomers) *δ* –179.30 (ddd, *J* = 47.1, 22.4, 15.6 Hz), –181.62 (ddd, *J* = 49.0, 31.0, 18.9 Hz). MS (EI): *m/z* (%) = 199 (11), 198 (100), 171 (35), 154 (33), 108 (8), 77 (3), 63 (2). IR (ATR): *v* = 3012, 2930, 2841, 2189, 1590, 1491, 1407, 1310, 1254, 1139, 1087, 1012, 979, 872, 828, 767, 730, 671 cm^{-1}. HRMS *m/z*: calcd for $[C_{16}H_{17}BrFNO_2S+H]^+$: 386.0220, found: 386.0220.

{[2-Fluoro-2-(*p*-tolyl)ethyl]imino}(4-methoxyphenyl)(methyl)-λ^6-sulfanone (7fe)

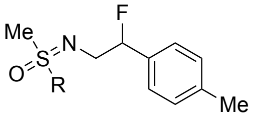

R = 4-MeO-Ph

Following the general procedure afforded the product as a yellow viscous oil (57 mg, 89% yield). dr = 1 : 1. ^{1}H NMR (600 MHz, $CDCl_3$, mixture of diastereomers) *δ* 7.82 – 7.79 (m, 2H), 7.73 – 7.69 (m, 2H), 7.24 – 7.11 (m, 8H), 7.02 – 6.98 (m, 2H), 6.98 – 6.94 (m, 2H), 5.50 (m, 2H), 3.87 (s, 3H), 3.86 (s, 3H), 3.44 (ddd, *J* = 15.6, 13.4, 7.6 Hz, 1H), 3.30 – 3.08 (m, 7H), 3.07 (s, 3H), 2.34 (s, 3H), 2.31 (s, 3H). ^{13}C {^{1}H} (151 MHz, $CDCl_3$, mixture of diastereomers) *δ* 163.2, 163.2, 138.1, 135.7 (d, J_{FC} = 19.7 Hz), 135.5 (d, J_{FC} = 19.7 Hz), 130.9, 130.7, 130.3, 130.0, 128.9, 126.0 (d, J_{FC} = 6.6 Hz), 125.7 (d, J_{FC} = 6.6 Hz), 114.6, 114.6, 95.6 (d, J_{FC} = 173.4 Hz), 94.7 (d, J_{FC} = 173.4 Hz), 55.6, 49.8 (d, J_{FC} = 24.0 Hz), 49.6 (d, J_{FC} = 27.1 Hz), 45.7, 45.3, 21.2, 21.1. ^{19}F NMR (564 MHz, $CDCl_3$, mixture of diastereomers) *δ* –177.48 (ddd, *J* = 47.7, 26.3, 15.8 Hz), –179.47 (ddd, *J* = 49.7, 33.1, 17.9 Hz). MS (EI): *m/z* (%) = 322 (4), 200 (10), 198 (100), 171 (48), 156 (9), 155 (37), 123 (22), 92 (3), 77 (9). IR (ATR): *v* = 3019, 2921, 2844, 2167, 1590, 1494, 1445, 1414, 1308, 1253, 1133, 1022, 957, 909, 872, 808, 766, 706, 661 cm^{-1}. HRMS *m/z*: calcd for $[C_{17}H_{20}FNO_2S+H]^+$: 322.1272, found: 322.1266.

{[2-(4-(*tert*-Butyl)phenyl]-2-fluoroethyl}imino)(4-methoxyphenyl)(methyl)-λ^6-sulfanone (7ff)

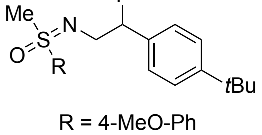

R = 4-MeO-Ph

Following the general procedure afforded the product as a yellow viscous oil (66 mg, 91% yield). dr = 1 : 1. ^{1}H NMR (600 MHz, $CDCl_3$, mixture of diastereomers) *δ* 7.82 – 7.78 (m, 2H), 7.72 – 7.69 (m, 2H), 7.38 – 7.22 (m, 8H), 7.02 – 6.98 (m, 2H), 6.98 – 6.94 (m, 2H), 5.63 – 5.43 (m, 2H), 3.87 (s, 3H), 3.86 (s, 3H), 3.45 (ddd, *J* = 15.4, 13.4, 7.7 Hz, 1H), 3.32 – 3.09 (m, 7H), 3.08 (s, 3H), 1.31 (s, 9H), 1.28 (s, 9H). ^{13}C {^{1}H} (151 MHz, $CDCl_3$, mixture of diastereomers) *δ* 163.2, 163.2, 151.3, 151.3, 135.7 (d, J_{FC} = 19.9 Hz), 135.5 (d, J_{FC} = 19.9 Hz), 131.0, 130.7, 130.2, 130.0, 129.8, 125.8 (d, J_{FC} = 6.8 Hz), 125.4 (d, J_{FC} = 6.8 Hz), 125.2, 114.6, 114.6, 114.3, 95.5 (d, J_{FC} = 174.1 Hz), 94.5 (d, J_{FC} = 173.0 Hz), 55.6, 49.8 (d, J_{FC} = 23.5 Hz), 49.6 (d, J_{FC} = 27.0 Hz), 45.7, 45.4, 34.5, 34.5, 31.3, 31.2. ^{19}F NMR (564 MHz, $CDCl_3$, mixture of diastereomers) *δ* –177.71 (ddd, *J* =

47.7, 26.9, 15.6 Hz), –180.01 (ddd, J = 48.4, 33.8, 18.1 Hz). MS (EI): m/z (%) = 200 (5), 198 (100), 171 (35), 155 (28), 150 (7), 135 (11), 108 (4), 77 (4). IR (ATR): ν = 2959, 2843, 2165, 1591, 1494, 1463, 1409, 1363, 1310, 1255, 1140, 1088, 1022, 979, 873, 803, 767, 734, 699 cm^{-1}. HRMS m/z: calcd for $[C_{20}H_{26}FNO_2S+H]^+$: 364.1741, found: 364.1736.

{[2-Fluoro-2-(4-methoxyphenyl)ethyl]imino}(4-methoxyphenyl)(methyl)-λ^6-sulfanone (7fg)

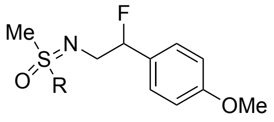

R = 4-MeO-Ph

Following the general procedure afforded the product as a yellow viscous oil (60 mg, 89% yield). dr = 1 : 1.2. ^{1}H NMR (600 MHz, $CDCl_3$, mixture of diastereomers) δ 7.82 – 7.80 (m, 2H), 7.73 – 7.70 (m, 2.4H), 7.27 –7.22 (m, 6.0H), 7.02 – 6.99 (m, 2H), 6.98 – 6.95 (m, 2.4H), 6.85 (m, 4.4H), 5.48 (m, 2.2H), 3.87 (s, 3H), 3.86 (s, 3.6H), 3.79 (s, 3.6H), 3.77 (s, 3.0H), 3.44 (ddd, J = 15.1, 13.3, 7.5 Hz, 1H), 3.28 – 3.08 (m, 7.4H), 3.07 (s, 3.6H). ^{13}C {^{1}H} (151 MHz, $CDCl_3$, mixture of diastereomers) δ 163.3, 163.2, 159.6, 159.6, 131.0, 130.9, 130.7, 130.7, 130.5, 130.2, 130.0, 127.6 (d, J_{FC} = 6.2 Hz), 127.3 (d, J_{FC} = 6.2 Hz), 114.7, 114.6, 113.6, 95.4 (d, J_{FC} = 173.7 Hz), 94.4 (d, J_{FC} = 173.7 Hz), 55.6, 55.2, 55.2, 49.7 (d, J_{FC} = 24.8 Hz), 49.4 (d, J_{FC} = 28.0 Hz), 45.7, 45.4, 14.2. ^{19}F NMR (564 MHz, $CDCl_3$, mixture of diastereomers) δ –174.48 (ddd, J = 47.5, 25.2, 15.2 Hz), –176.42 (ddd, J = 49.3, 32.3, 17.9 Hz). MS (EI): m/z (%) = 317 (15), 199 (11), 198 (100), 171 (40), 155 (32), 139 (24), 96 (5), 77 (3). IR (ATR): ν = 3737, 3020, 2959, 2923, 2842, 2016, 1581, 1495, 1460, 1308, 1251, 1174, 1135, 1089, 1018, 865, 804, 745, 663 cm^{-1}. HRMS m/z: calcd for $[C_{17}H_{20}FNO_3S+Na]^+$: 360.1040, found: 360.1039.

{[2-([1,1'-Biphenyl]-4-yl)-2-fluoroethyl]imino}(4-methoxyphenyl)(methyl)-λ^6-sulfanone (7fh)

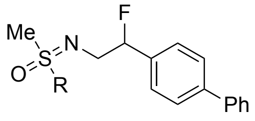

R = 4-MeO-Ph

Following the general procedure afforded the product as a yellow viscous oil (72 mg, 93% yield). dr = 1 : 1.2. ^{1}H NMR (600 MHz, $CDCl_3$, mixture of diastereomers) δ 7.83 – 7.80 (m, 2.0H), 7.73 – 7.70 (m, 2.4H), 7.59 – 7.54 (m, 8.8H), 7.46 – 7.32 (m, 11H), 7.02 – 6.99 (m, 2H), 6.98 – 6.94 (m, 2.4H), 5.60 (ddd, J = 97.8, 7.9, 3.9 Hz, 2.2H), 3.87 (s, 3H), 3.85 (s, 3.6H), 3.49 (ddd, J = 15.5, 13.3, 7.3 Hz, 1H), 3.38 – 3.29 (m, 1H), 3.26 – 3.14 (m, 2.4H), 3.12 (s, 3H), 3.09 (s, 3.6H). ^{13}C {^{1}H} (151 MHz, $CDCl_3$, mixture of diastereomers) δ 163.3, 163.2, 141.2, 140.7, 140.6, 137.7 (d, J_{FC} = 19.7 Hz), 137.5 (d, J_{FC} = 19.7 Hz), 131.0, 130.7, 130.1, 129.9, 128.8, 128.7, 127.4, 127.1, 127.0, 127.0, 126.5 (d, J_{FC} = 6.8 Hz), 126.2 (d, J_{FC} = 6.8 Hz), 114.7, 114.6, 95.3 (d, J_{FC} = 174.5 Hz), 94.5 (d, J_{FC} = 174.5 Hz), 55.6, 55.6, 49.8 (d, J_{FC} = 23.5 Hz), 49.6 (d, J_{FC} = 27.3 Hz), 45.7, 45.4. ^{19}F NMR (564 MHz, $CDCl_3$, mixture of diastereomers) δ –178.47 (ddd, J = 47.3, 24.8, 15.5 Hz), –180.82 (ddd, J = 48.5, 32.6, 18.4 Hz). MS (EI): m/z (%) = 198 (100), 185 (18), 171 (28), 165 (5), 155 (23), 139 (3), 77 (3). IR (ATR): ν = 3565, 3027, 2963, 2927, 2839, 2195, 1592, 1491, 1457, 1408, 1310, 1257, 1138, 1090, 1017, 980, 957, 908, 871, 831, 802, 764, 732, 694 cm^{-1}. HRMS m/z: calcd for $[C_{22}H_{22}FNO_2S+Na]^+$: 406.1247, found: 406.1248.

{[2-(4-(Chloromethyl)phenyl)-2-fluoroethyl]imino}(4-methoxyphenyl)(methyl)-λ^6-sulfanone (7fi)

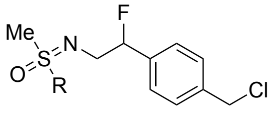

R = 4-MeO-Ph

Following the general procedure afforded the product as a yellow viscous oil (64 mg, 90% yield). dr = 1 : 1.2. ^{1}H NMR (600 MHz, $CDCl_3$, mixture of diastereomers) δ 7.77 – 7.73 (m, 2.4H), 7.65 – 7.62 (m, 2H), 7.38 – 7.28 (m, 8.8H), 7.01 – 6.98 (m, 2.4H), 6.97 – 6.94 (m, 2H), 5.65

– 5.43 (m, 2.4H), 4.58 (s, 2H), 4.55 (s, 2.4H), 3.86 (s, 3.6H), 3.86 (s, 3H), 3.44 (ddd, *J* = 15.4, 13.2, 7.1 Hz, 1H), 3.28 (ddd, *J* = 31.9, 13.8, 3.0 Hz, 1.2H), 3.18 – 3.07 (m, 5.8H), 3.06 (s, 3.0H). ^{13}C {^{1}H} (151 MHz, $CDCl_3$, mixture of diastereomers) *δ* 163.3, 163.2, 139.1 (d, J_{FC} = 19.6 Hz), 138.8 (d, J_{FC} = 19.6 Hz), 137.5, 130.9, 130.7, 129.9, 129.8, 128.5, 126.4 (d, J_{FC} = 6.7 Hz), 126.1 (d, J_{FC} = 7.4 Hz), 114.7, 114.6, 95.1 (d, J_{FC} = 175.2 Hz), 94.2 (d, J_{FC} = 175.2 Hz), 55.6, 49.8 (d, J_{FC} = 23.7 Hz), 49.6 (d, J_{FC} = 27.6 Hz), 45.9, 45.8, 45.6, 45.4. ^{19}F NMR (564 MHz, $CDCl_3$, mixture of diastereomers) *δ* –179.62 (ddd, *J* = 47.2, 23.3, 15.3 Hz), –181.98 (ddd, *J* = 49.4, 31.9, 19.1 Hz). MS (EI): *m/z* (%) = 356 (1), 199 (11), 198 (100), 171 (30), 155 (27), 122 (7), 77 (2). IR (ATR): *ν* = 3031, 2930, 2841, 1590, 1494, 1444, 1310, 1255, 1138, 1088, 1021, 978, 835, 803, 764, 675 cm^{-1}. HRMS *m/z*: calcd for $[C_{17}H_{19}ClFNO_2S+Na]^+$: 378.0701, found: 378.0701.

4-(1-Fluoro-2-{[(4-methoxyphenyl)(methyl)(oxo)-λ^6-sulfaneylidene)amino]ethyl}phenyl acetate (7fj)

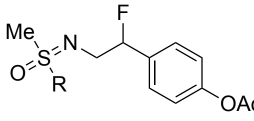

R = 4-MeO-Ph

Following the general procedure afforded the product as a yellow viscous oil (57 mg, 79% yield). dr = 1 : 1. ^{1}H NMR (600 MHz, $CDCl_3$, mixture of diastereomers) *δ* 7.74 – 7.71 (m, 2H), 7.61 – 7.57 (m, 2H), 7.33 (d, *J* = 8.3 Hz, 2H), 7.31 – 7.28 (m, 2H), 7.06 – 7.01 (m, 4H), 7.00 – 6.94 (m, 4H), 5.61 – 5.42 (m, 2H), 3.84 (s, 3H), 3.83 (s, 3H), 3.42 (ddd, *J* = 14.9, 13.1, 7.1 Hz, 1H), 3.31 – 3.22 (m, 1H), 3.16 – 3.01 (m, 2H), 3.07 (s, 3H), 3.03 (s, 3H), 2.27 (s, 3H), 2.25 (s, 3H). ^{13}C {^{1}H} (151 MHz, $CDCl_3$, mixture of diastereomers) *δ* 169.3, 169.3, 169.0, 163.2, 163.2, 150.5, 150.4, 136.4 (d, J_{FC} = 20.4 Hz), 136.1 (d, J_{FC} = 20.0 Hz), 130.8, 130.6, 129.7, 129.7, 129.3, 127.2 (d, J_{FC} = 6.7 Hz), 126.8 (d, J_{FC} = 6.7 Hz), 121.4, 121.3, 114.9, 114.8, 114.6, 114.6, 94.9 (d, J_{FC} = 174.7 Hz), 94.1 (d, J_{FC} = 174.2 Hz), 55.6, 49.8 (d, J_{FC} = 23.6 Hz), 49.5 (d, J_{FC} = 28.3 Hz), 45.5, 45.4, 45.3, 21.0, 21.0, 21.0. ^{19}F NMR (564 MHz, $CDCl_3$, mixture of diastereomers) *δ* –178.35 (ddd, *J* = 47.0, 22.9, 14.8 Hz), –180.82 (dddd, *J* = 51.4, 31.9, 19.4, 3.5 Hz). MS (EI): *m/z* (%) = 366 (1), 199 (9), 198 (100), 171 (26), 155 (33), 135 (11), 77 (8). IR (ATR): *ν* = 3067, 3012, 2930, 2740, 2173, 1590, 1494, 1468,1439, 1411, 1309, 1254, 1225, 1140, 1088, 1021, 979, 875, 834, 803, 758, 679 cm^{-1}. HRMS *m/z*: calcd for $[C_{18}H_{20}FNO_4S+H]^+$: 366.1170, found: 366.1170.

{[2-Fluoro-2-(3-fluorophenyl)ethyl]imino}(4-methoxyphenyl)(methyl)-λ^6-sulfanone (7fk)

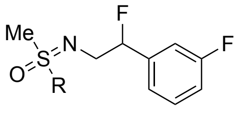

R = 4-MeO-Ph

Following the general procedure afforded the product as a yellow viscous oil (36 mg, 56% yield). dr = 1 : 1. ^{1}H NMR (600 MHz, $CDCl_3$, mixture of diastereomers) *δ* 7.80 – 7.77 (m, 2H), 7.72 – 7.68 (m, 2H), 7.34 – 7.26 (m, 2H), 7.13 – 6.95 (m, 10H), 5.62 – 5.43 (m, 2H), 3.88 (s, 3H), 3.87 (s, 3H), 3.42 (ddd, *J* = 16.3, 13.3, 7.1 Hz, 1H), 3.28 (ddd, *J* = 32.0, 13.8, 3.1 Hz, 1H), 3.18 – 3.09 (m, 5H), 3.07 (s, 3H). ^{13}C {^{1}H} (151 MHz, $CDCl_3$, mixture of diastereomers) *δ* 163.5, 163.4, 163.3, 161.9, 141.3 (dd, J_{FC} = 20.0, 7.2 Hz), 141.1 (dd, J_{FC} = 20.0, 7.2 Hz), 130.9, 130.7, 130.1, 129.9, 129.9, 129.8, 129.8, 121.6 (dd, J_{FC} = 7.3, 3.2 Hz), 121.2 (dd, J_{FC} = 7.3, 3.2 Hz), 115.2, 115.0, 114.7, 114.7, 114.4, 113.1 (dd, J_{FC} = 22.3, 7.6 Hz), 112.7 (dd, J_{FC} = 22.6, 7.9 Hz), 94.6 (d, J_{FC} = 176.8 Hz), 93.8 (d, J_{FC} = 175.8 Hz), 55.6, 49.7 (d, J_{FC} = 23.6 Hz), 49.4 (d, J_{FC} = 27.1 Hz), 45.7, 45.4. ^{19}F NMR (564 MHz, $CDCl_3$, mixture of diastereomers) *δ* –112.83 (td, *J* = 9.2, 5.8 Hz), –112.96 (td, *J* = 9.2, 5.7 Hz), –179.85 (ddd, *J* = 47.3, 23.7, 16.3 Hz), –182.09 (ddd, *J* = 49.4, 32.1, 18.8 Hz). MS (EI): *m/z* (%) = 199 (10), 198 (100), 171 (53), 155 (30), 154 (10), 127 (18), 77 (4). IR (ATR): *ν* = 3013, 2962, 2842, 2166, 1590, 1492, 1449, 1411, 1309, 1256, 1139, 1087, 1018, 868, 833, 794,

694 cm^{-1}. HRMS *m/z*: calcd for $[C_{16}H_{17}F_2NO_2S+H]^+$: 326.1021 found: 326.1016.

{[2-(3-Chlorophenyl)-2-fluoroethyl]imino}(4-methoxyphenyl)(methyl)-λ^6-sulfanone (7fl)

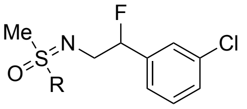

R = 4-MeO-Ph

Following the general procedure afforded the product as a yellow viscous oil (50 mg, 73% yield). dr = 1 : 1. ^{1}H NMR (600 MHz, $CDCl_3$, mixture of diastereomers) δ 7.79 – 7.75 (m, 2H), 7.69 – 7.64 (m, 2H), 7.34 – 7.16 (m, 8H), 7.02 – 6.99 (m, 2H), 6.99 – 6.96 (m, 2H), 5.61 – 5.40 (m, 2H), 3.88 (s, 3H), 3.87 (s, 3H), 3.42 (ddd, J = 15.9, 13.3, 7.0 Hz, 1H), 3.28 (ddd, J = 31.7, 13.8, 3.1 Hz, 1H), 3.17 – 3.07 (m, 5H), 3.06 (s, 3H). ^{13}C {^{1}H} (151 MHz, $CDCl_3$, mixture of diastereomers) δ 163.3, 163.3, 140.8 (d, J_{FC} = 19.7 Hz), 140.5 (d, J_{FC} = 19.7Hz), 134.2, 134.2, 130.9, 130.7, 129.9, 129.8, 129.6, 129.5, 128.4, 128.4, 126.2 (d, J_{FC} = 7.7 Hz), 125.8 (d, J_{FC} = 7.7 Hz), 124.3 (d, J_{FC} = 6.8 Hz), 123.8 (d, J_{FC} = 6.8 Hz), 114.7, 114.7, 94.6 (d, J_{FC} = 176.8 Hz), 93.8 (d, J_{FC} = 175.5 Hz), 55.6, 49.6 (d, J_{FC} = 24.3 Hz), 49.3 (d, J_{FC} = 27.5 Hz), 45.6, 45.4. ^{19}F NMR (564 MHz, $CDCl_3$, mixture of diastereomers) δ –179.94 (ddd, J = 47.2, 22.9, 15.8 Hz), –182.52 (ddd, J = 48.3, 31.6, 19.1 Hz). MS (EI): *m/z* (%) = 198 (100), 171 (34), 155 (31), 143 (9), 77 (2). IR (ATR): ν = 3012, 2931, 2841, 2164, 1590, 1493, 1436, 1309, 1254, 1139, 1088, 1022, 979, 885, 836, 797, 694 cm^{-1}. HRMS *m/z*: calcd for $[C_{16}H_{17}ClFNO_2S+Na]^+$: 364.0545, found: 364.0545.

{[2-(3-Bromophenyl)-2-fluoroethyl]imino}(4-methoxyphenyl)(methyl)-λ^6-sulfanone (7fm)

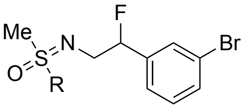

R = 4-MeO-Ph

Following the general procedure afforded the product as a yellow viscous oil (60 mg, 78% yield). dr = 1 : 1. ^{1}H NMR (600 MHz, $CDCl_3$, mixture of diastereomers) δ 7.78 – 7.74 (m, 2H), 7.68 – 7.64 (m, 2H), 7.51 – 7.39 (m, 4H), 7.29 – 7.17 (m, 4H), 7.02 – 6.96 (m, 4H), 5.59 – 5.39 (m, 2H), 3.88 (s, 3H), 3.87 (s, 3H), 3.42 (ddd, J = 15.8, 13.2, 7.0 Hz, 1H), 3.33 – 3.23 (m, 1H), 3.17 – 3.07 (m, 5H), 3.06 (s, 3H). ^{13}C {^{1}H} (151 MHz, $CDCl_3$, mixture of diastereomers) δ 163.4, 163.3, 141.1 (d, J_{FC} = 20.2 Hz), 140.8 (d, J_{FC} = 19.7 Hz), 131.3, 131.3, 130.9, 130.7, 129.9, 129.9, 129.8, 129.8, 129.1 (d, J_{FC} = 7.2 Hz), 128.7 (d, J_{FC} = 7.2 Hz), 124.8 (d, J_{FC} = 7.1 Hz), 124.3 (d, J_{FC} = 7.1 Hz), 122.4, 114.7, 114.7, 94.6 (d, J_{FC} = 176.6 Hz), 93.7 (d, J_{FC} = 176.6 Hz), 55.6, 49.7 (d, J_{FC} = 24.1 Hz), 49.3 (d, J_{FC} = 27.4 Hz), 45.6, 45.4. ^{19}F NMR (564 MHz, $CDCl_3$, mixture of diastereomers) δ –179.90 (ddd, J = 47.0, 22.7, 15.8 Hz), –182.54 (ddd, J = 49.2, 31.5, 19.2 Hz). MS (EI): *m/z* (%) = 199 (11), 198 (100), 171 (33), 123 (3), 108 (7), 77 (3). IR (ATR): ν = 3067, 3012, 2929, 2841, 2195,1590, 1493, 141, 1310, 1254, 1139, 1089, 1022, 979, 883, 835, 800, 693 cm^{-1}. HRMS *m/z*: calcd for $[C_{16}H_{17}BrFNO_2S+H]^+$: 386.0220, found: 386.0221.

{[2-Fluoro-2-(*m*-tolyl)ethyl]imino}(4-methoxyphenyl)(methyl)-λ^6-sulfanone (7fn)

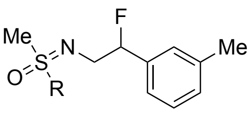

R = 4-MeO-Ph

Following the general procedure afforded the product as a yellow viscous oil (55 mg, 86% yield). dr = 1 : 1. ^{1}H NMR (600 MHz, $CDCl_3$, mixture of diastereomers) δ 7.82 – 7.79 (m, 2H), 7.72 – 7.69 (m, 2H), 7.21 (dt, J = 14.2, 7.6 Hz, 2H), 7.15 – 7.08 (m, 6H), 7.02 – 6.99 (m, 2H), 6.98 – 6.95 (m, 2H), 5.61 – 5.41 (m, 2H), 3.87 (s, 3H), 3.86 (s, 3H), 3.44 (ddd, J = 15.7, 13.4, 7.7 Hz, 1H), 3.27 (ddd, J = 33.8, 13.8, 2.8 Hz, 1H), 3.21 – 3.08 (m, 6H), 3.07 (s, 3H), 2.33 (s, 3H), 2.32 (s, 3H). ^{13}C {^{1}H} (151 MHz, $CDCl_3$, mixture of diastereomers) δ 163.3, 163.2, 138.6 (d, J_{FC} = 19.5 Hz), 138.5 (d, J_{FC} = 19.5 Hz), 137.9, 131.0, 130.7, 130.3, 130.0, 129.1, 129.0, 128.2, 128.1, 126.6 (d, J_{FC} = 6.7 Hz), 126.2 (d, J_{FC} = 6.7 Hz), 123.2 (d, J_{FC} = 6.7 Hz), 122.7 (d, J_{FC} = 6.7 Hz), 114.6,

114.6, 95.6 (d, J_{FC} = 174.8 Hz), 94.6 (d, J_{FC} = 174.8 Hz), 55.6, 49.8 (d, J_{FC} = 23.8 Hz), 49.6 (d, J_{FC} = 26.8 Hz), 45.7, 45.3, 21.4, 21.3. ^{19}F NMR (564 MHz, $CDCl_3$, mixture of diastereomers) δ –178.53 (ddd, J = 47.7, 26.7, 15.8 Hz), –180.95 (ddd, J = 48.4, 33.8, 18.3 Hz). MS (EI): *m/z* (%) = 322 (8), 198 (100), 171 (46), 155 (35), 154 (13), 123 (23), 77 (10). IR (ATR): ν = 3014, 2926, 2841, 2165, 1591, 1493, 1460, 1411, 1309, 1253, 1139, 1088, 1023, 979, 888, 835, 797, 702 cm^{-1}. HRMS *m/z*: calcd for $[C_{17}H_{20}FNO_2S+H]^+$: 322.1272, found: 322.1268.

{[2-Fluoro-2-(2-fluorophenyl)ethyl]imino}(4-methoxyphenyl)(methyl)-λ^6-sulfanone (7fo)

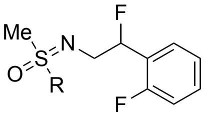

R = 4-MeO-Ph

Following the general procedure afforded the product as a yellow viscous oil (40 mg, 62% yield). dr = 1 : 1. ^{1}H NMR (600 MHz, $CDCl_3$, mixture of diastereomers) δ 7.80 – 7.76 (m, 4H), 7.43 (m, 2H), 7.28 (m, 2H), 7.13 (m, 2H), 7.03 – 6.98 (m, 6H), 5.88 (ddd, J = 29.1, 7.8, 3.5 Hz, 1H), 5.80 (ddd, J = 28.8, 7.8, 3.5 Hz, 1H), 3.88 (s, H), 3.87 (s, H), 3.46 – 3.30 (m, 2H), 3.28 – 3.17 (m, 2H), 3.10 (s, 3H), 3.08 (s, 3H). ^{13}C {^{1}H} (151 MHz, $CDCl_3$, mixture of diastereomers) δ 163.3, 160.2 (dd, J_{FC} = 8.7, 5.5 Hz), 158.6 (dd, J_{FC} = 8.7, 5.5 Hz), 130.9, 130.7, 130.3, 130.0, 129.9, 129.8, 127.6 (dd, J_{FC} = 7.8, 3.7 Hz), 127.5 (dd, J_{FC} = 9.1, 3.9 Hz), 126.0, 125.9, 125.9, 125.9, 125.8, 125.8, 125.8, 125.7, 124.1, 124.1, 124.1, 124.0, 115.3 (d, J_{FC} = 8.7 Hz), 115.1 (d, J_{FC} = 8.7 Hz), 114.7, 89.9 (dd, J_{FC} = 174.0, 2.0 Hz), 89.4 (dd, J_{FC} = 175.2, 2.0 Hz), 55.6, 48.6 (d, J_{FC} = 5.8 Hz), 48.5 (d, J_{FC} = 4.3 Hz), 45.7, 45.4. ^{19}F NMR (564 MHz, $CDCl_3$, mixture of diastereomers) δ –118.30 (dt, J = 11.5, 6.3 Hz), –118.56 (dt, J = 11.4, 6.2 Hz), –185.93 (ddd, J = 42.4, 28.7, 18.5 Hz), –186.50 – –186.73 (m). MS (EI): *m/z* (%) = 199 (10), 198 (100), 171 (65), 155 (42), 154 (13), 127 (34), 107 (5), 77 (5). IR (ATR): ν = 3013, 2932, 2842, 2166, 1590, 1492, 1456, 1410, 1309, 1255, 1181, 1140, 1088, 1023, 979, 877, 834, 802, 761, 671 cm^{-1}. HRMS *m/z*: calcd for $[C_{16}H_{17}F_2NO_2S+H]^+$: 326.1021, found: 326.1016.

{[2-(2-Chlorophenyl)-2-fluoroethyl]imino}(4-methoxyphenyl)(methyl)-λ^6-sulfanone (7fp)

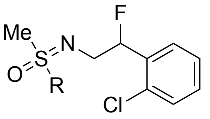

R = 4-MeO-Ph

Following the general procedure afforded the product as a yellow viscous oil (44 mg, 65% yield). dr = 1 : 1. ^{1}H NMR (600 MHz, $CDCl_3$, mixture of diastereomers) δ 7.84 – 7.81 (m, 2H), 7.77 – 7.74 (m, 2H), 7.50 (dd, J = 7.7, 1.7 Hz, 1H), 7.47 (dd, J = 7.6, 1.8 Hz, 1H), 7.31 – 7.19 (m, 7H), 7.02 – 6.97 (m, 4H), 5.99 – 5.82 (m, 2H), 3.87 (m, 6H), 3.45 – 3.19 (m, 3H), 3.15 – 3.06 (m, 7H). ^{13}C {^{1}H} (151 MHz, $CDCl_3$, mixture of diastereomers) δ 163.3, 163.2, 136.3 (d, J_{FC} = 2.2 Hz), 136.2 (d, J_{FC} = 2.2 Hz), 131.3 (d, J_{FC} = 6.1 Hz), 131.2 (d, J_{FC} = 6.1 Hz), 130.9, 130.8, 130.3, 129.9, 129.3, 129.2, 129.2, 127.2 (d, J_{FC} = 19.8 Hz), 126.8 (d, J_{FC} = 20.1 Hz), 114.6, 114.6, 92.3 (d, J_{FC} = 175.7 Hz), 92.0 (d, J_{FC} = 175.7 Hz), 55.6, 55.6, 48.4 (d, J_{FC} = 10.9 Hz), 48.3 (d, J_{FC} = 10.9 Hz), 45.7, 45.4. ^{19}F NMR (564 MHz, $CDCl_3$, mixture of diastereomers) δ –186.82 (ddd, J = 47.6, 32.7, 20.6 Hz), –187.18 (ddd, J = 47.6, 33.0, 21.2 Hz). MS (EI): *m/z* (%) = 199 (11), 198 (100), 171 (43), 154 (36), 154 (12), 142 (13), 108 (5), 77 (3). IR (ATR): ν = 3011, 2930, 2841, 2166, 1730, 1591, 1493, 1441, 1410, 1309, 1255, 1227, 1139, 1087, 1024, 979, 875, 834, 802, 759, 706, 663 cm^{-1}. HRMS *m/z*: calcd for $[C_{16}H_{17}ClFNO_2S+Na]^+$: 364.0545, found: 364.0544.

{[2-(2-Bromophenyl)-2-fluoroethyl]imino}(4-methoxyphenyl)(methyl)-λ^6-sulfanone (7fq)

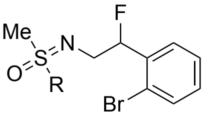

R = 4-MeO-Ph

Following the general procedure afforded the product as a yellow viscous oil (47 mg, 61% yield). dr = 1 : 1.4. ^{1}H NMR (600 MHz, $CDCl_3$, mixture of diastereomers) δ 7.85 – 7.82 (m, 2.8H), 7.76 – 7.74 (m, 2.0H), 7.50 – 7.44 (m, 4.8H), 7.30 (m, 2.4H), 7.14 (tdd, J = 7.8, 3.9, 1.7 Hz, 2.4H), 7.01 – 6.97 (m, 4.8H), 5.84 (m, 2.4H), 3.86 (s, 3H), 3.86 (s, 4.2H), 3.40 (ddd, J = 33.0, 14.0, 2.5 Hz, 1H), 3.29 – 3.22 (m, 2.8H), 3.11 (s, 3H), 3.11 (m, 4.2H), 3.11 – 3.04 (m, 1H). ^{13}C {^{1}H} (151 MHz, $CDCl_3$, mixture of diastereomers) δ 163.3, 163.2, 137.9, 137.8, 132.5, 132.4, 130.9, 130.8, 130.3, 129.8, 129.6, 129.5, 127.5, 127.4, 127.3, 121.0 (d, J_{FC} = 6.1 Hz), 120.9 (d, J_{FC} = 6.1 Hz),, 114.6, 114.6, 94.2 (d, J_{FC} = 176.6 Hz), 93.8 (d, J_{FC} = 176.6 Hz), 55.6, 55.6, 48.5 (d, J_{FC} = 23.7 Hz), 48.4 (d, J_{FC} = 23.7 Hz), 45.6, 45.3. ^{19}F NMR (564 MHz, $CDCl_3$, mixture of diastereomers) δ –185.44 (ddd, J = 47.3, 30.8, 22.3 Hz), –185.82 (ddd, J = 47.6, 33.0, 21.3 Hz). MS (EI): *m/z* (%) = 387 (3), 199 (8), 198 (100), 171 (33), 123 (4), 108 (7), 77 (9). IR (ATR): ν = 3013, 2932, 2842, 2164, 1756, 1639, 1591, 1497, 1462, 1369, 1310, 1255, 1194, 1139, 1089, 1017, 979, 911, 836, 804, 766, 731 cm^{-1}. HRMS *m/z*: calcd for $[C_{16}H_{17}BrFNO_2S+H]^+$: 386.0220, found: 386.0220.

{[2-Fluoro-2-(*o*-tolyl)ethyl]imino}(4-methoxyphenyl)(methyl)-λ^6-sulfanone (7fr)

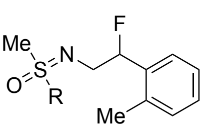

R = 4-MeO-Ph

Following the general procedure afforded the product as a yellow viscous oil (43 mg, 70% yield). dr = 1 : 1. ^{1}H NMR (600 MHz, $CDCl_3$, mixture of diastereomers) δ 7.82 – 7.79 (m, 2H), 7.76 – 7.72 (m, 2H), 7.39 – 7.35 (m, 1H), 7.35 – 7.32 (m, 1H), 7.21 – 7.15 (m, 4H), 7.13 – 7.08 (m, 2H), 7.02 – 6.99 (m, 2H), 6.99 – 6.96 (m, 2H), 5.76 (dddd, J = 47.5, 44.9, 8.4, 3.2 Hz, 2H), 3.87 (d, J = 0.8 Hz, 3H), 3.86 (d, J = 0.8 Hz, 3H), 3.44 – 3.37 (m, 1H), 3.27 – 3.19 (m, 1H), 3.10 (dd, J = 18.4, 0.8 Hz, 9H), 2.28 (s, 3H), 2.27 (s, 3H). ^{13}C {^{1}H} (151 MHz, $CDCl_3$, mixture of diastereomers) δ 163.3, 163.2, 136.9 (d, J_{FC} = 27.0 Hz), 136.7 (d, J_{FC} = 27.0 Hz), 134.6 (d, J_{FC} = 34.8 Hz), 134.5 (d, J_{FC} = 34.8 Hz), 131.0, 130.7, 130.3, 130.2, 130.2, 129.9, 128.1, 128.1, 126.0, 125.9, 125.6, 125.6, 125.3, 125.3, 114.7, 114.6, 93.3 (d, J_{FC} = 174.2 Hz), 92.5 (d, J_{FC} = 174.2 Hz), 91.6, 55.6, 49.1, 48.9, 48.9, 45.7, 45.3, 19.0, 18.9. ^{19}F NMR (564 MHz, $CDCl_3$, mixture of diastereomers) δ –181.28 – –181.50 (m), –183.40 (dddd, J = 48.0, 34.6, 19.2, 3.9 Hz). MS (EI): *m/z* (%) = 198 (100), 171 (46), 155 (39), 154 (13), 123 (18), 103 (6), 77 (11). IR (ATR): ν = 3019, 2930, 2841, 2171, 1590, 1493, 1460, 1410, 1309, 1254, 1139, 1087, 1025, 978, 872, 835, 803, 760, 727, 672 cm^{-1}. HRMS *m/z*: calcd for $[C_{17}H_{20}FNO_2+H]^+$: 322.1272, found: 322.1266.

{[2-Fluoro-2-(naphthalen-1-yl)ethyl]imino}(4-methoxyphenyl)(methyl)-λ^6-sulfanone (7fs)

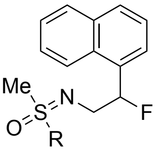

R = 4-MeO-Ph

Following the general procedure afforded the product as a yellow viscous oil (61 mg, 86% yield). dr = 1 : 1.3. ^{1}H NMR (600 MHz, $CDCl_3$, mixture of diastereomers) δ 7.85 – 7.79 (m, 9.1H), 7.78 – 7.75 (m, 2.6H), 7.64 – 7.61 (m, 2.6H), 7.50 – 7.40 (m, 7H), 6.97 – 6.93 (m, 2H), 6.87 – 6.83 (m, 2.6H), 5.81 – 5.60 (m, 2.3H), 3.85 (s, 3H), 3.82 (s, 3.9H), 3.55 (ddd, J = 15.3, 13.3, 7.2 Hz, 1.3H), 3.39 (ddd, J = 32.3, 13.8, 3.0 Hz, 1H), 3.30 – 3.18 (m, 2.3H), 3.11 (s, 3H), 3.06 (s, 3.9H). ^{13}C {^{1}H} (151 MHz, $CDCl_3$, mixture of diastereomers) δ 163.2, 163.1, 136.2 (d, J_{FC} = 19.2 Hz), 136.0 (d, J_{FC} = 19.2 Hz), 133.2, 133.2, 133.0, 132.9, 130.9, 130.6, 130.1, 129.9, 128.1, 128.0, 128.0, 127.6, 126.2, 126.2, 126.2, 126.1, 125.4 (d, J_{FC} = 7.0 Hz), 124.8 (d, J_{FC} = 7.0 Hz), 123.8 (d,

J_{FC} = 5.5 Hz), 123.4 (d, J_{FC} = 5.5 Hz), 114.6, 114.5, 95.7 (d, J_{FC} = 175.8 Hz), 94.8 (d, J_{FC} = 174.4 Hz), 55.6, 55.6, 49.8 (d, J_{FC} = 23.7 Hz), 49.6 (d, J_{FC} = 28.0 Hz), 45.7, 45.4. ^{19}F NMR (564 MHz, $CDCl_3$, mixture of diastereomers) *δ* –178.25 (ddd, *J* = 47.4, 23.9, 15.3 Hz), –181.08 (ddd, *J* = 48.3, 32.3, 19.0 Hz). MS (EI): *m/z* (%) = 200 (6), 198 (100), 171 (38), 159 (23), 155 (31), 154 (11), 133 (9), 77 (3). IR (ATR): *ν* = 3017, 2926, 2849, 2194, 1590, 1493, 1462, 1411, 1308, 1253, 1177, 1129, 1086, 1017, 972, 864, 827, 752, 711, 658 cm^{-1}. HRMS *m/z*: calcd for $[C_{20}H_{20}FNO_2S+Na]^+$: 380.1091, found: 380.1092.

[(2-Fluoro-2-mesitylethyl)imino](4-methoxyphenyl)(methyl)-λ^6-sulfanone (7ft)

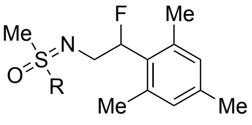

R = 4-MeO-Ph

Following the general procedure afforded the product as a yellow viscous oil (28+7 mg, 50% yield). dr = 1 : 4. ^{1}H NMR (600 MHz, $CDCl_3$, major of diastereomers) *δ* 7.73 – 7.68 (m, 2H), 6.98 – 6.93 (m, 2H), 6.78 (s, 2H), 5.86 (ddd, *J* = 47.4, 8.7, 4.5 Hz, 1H), 3.87 (s, 3H), 3.61 (td, *J* = 13.3, 8.7 Hz, 1H), 3.11 (s, 3H), 3.06 (ddd, *J* = 27.7, 13.7, 4.5 Hz, 1H), 2.27 (d, *J* = 2.4 Hz, 6H), 2.23 (s, 3H). ^{13}C {^{1}H} (151 MHz, $CDCl_3$, major of diastereomers) *δ* 163.2, 137.5, 136.2 (d, J_{FC} = 3.2 Hz), 131.3 (d, J_{CF} = 17.2 Hz), 130.7, 130.3, 129.8, 114.6, 93.1 (d, J_{CF} = 175.3 Hz), 55.6, 47.0 (d, J_{CF} = 26.4 Hz) 45.4, 20.8, 20.4, 20.3. ^{19}F NMR (564 MHz, $CDCl_3$, major of diastereomers) *δ* –184.10 (ddd, *J* = 44.1, 27.9, 13.3 Hz). ^{1}H NMR (600 MHz, $CDCl_3$, minor of diastereomers) *δ* 7.89 – 7.82 (m, 2H), 7.04 – 6.96 (m, 2H), 6.74 (s, 2H), 6.01 – 5.84 (m, 1H), 3.87 (s, 3H), 3.14 – 2.98 (m, 4H), 2.03 (s, 9H). MS (EI): *m/z* (%) = 199 (11), 198 (100), 171 (23), 155 (19), 151 (7), 77 (2). IR (ATR): *ν* = 3010, 2922, 2847, 2164, 1591, 1492, 1456, 1411, 1309, 1252, 1139, 1086, 1023, 980, 835, 802, 766 cm^{-1}. HRMS *m/z*: calcd for $[C_{19}H_{24}FNO_2S+Na]^+$: 372.1404, found: 372.1405.

[(2-Fluoro-2-phenylpropyl)imino](4-methoxyphenyl)(methyl)-λ^6-sulfanone (7fu)

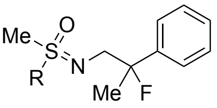

R = 4-MeO-Ph

Following the general procedure afforded the product as a yellow viscous oil (53 mg, 83% yield). dr = 1 : 1.2. ^{1}H NMR (600 MHz, $CDCl_3$, mixture of diastereomers) *δ* 7.71 – 7.69 (m, 2H), 7.65 – 7.62 (m, 2.2H), 7.41 – 7.23 (m, 11H), 6.97 – 6.92 (m, 4.4H), 3.86 (s, 3.6H), 3.85 (s, 3H), 3.37 (dd, *J* = 16.2, 13.1 Hz, 1.2H), 3.24 (dd, *J* = 27.8, 13.4 Hz, 1H), 3.14 – 3.05 (m, 5.3H), 3.02 (s, 3.6H), 1.79 – 1.74 (m, 6.6H). ^{13}C {^{1}H} (151 MHz, $CDCl_3$, mixture of diastereomers) *δ* 163.1, 163.1, 143.7 (d, J_{FC} = 21.4 Hz), 143.6 (d, J_{FC} = 21.4 Hz), 130.8, 130.7, 130.5, 130.4, 128.0, 127.9, 127.3, 124.9 (d, J_{FC} = 9.2 Hz), 124.5 (d, J_{FC} = 9.2 Hz), 114.5, 114.5, 97.7 (d, J_{FC} = 175.6 Hz), 97.5 (d, J_{FC} = 174.3 Hz), 55.6, 53.5, 53.4, 53.3, 53.2, 45.6, 45.3, 24.4 (d, J_{FC} = 24.3 Hz), 24.0 (d, J_{FC} = 24.3 Hz). ^{19}F NMR (564 MHz, $CDCl_3$, mixture of diastereomers) *δ* –149.70 (pd, *J* = 22.5, 16.3 Hz), –151.76 – -152.04 (m). MS (EI): *m/z* (%) = 322 (2), 199 (11), 198 (100), 171 (40), 154 (35), 123 (13), 103 (9), 77 (8). IR (ATR): *ν* = 2983, 2933, 2841, 2166, 1581, 1494, 1448, 1410, 1308, 1252, 1181, 1140, 1088, 1023, 980, 836, 802, 767, 767, 699 cm^{-1}. HRMS *m/z*: calcd for $[C_{17}H_{20}FNO_2S+Na]^+$: 344.1091, found: 344.1090.

[(2-Fluoro-2,2-diphenylethyl)imino](4-methoxyphenyl)(methyl)-λ^6-sulfanone (7fv)

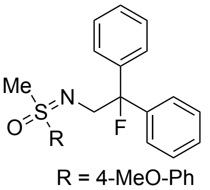

R = 4-MeO-Ph

Following the general procedure afforded the product as a yellow viscous oil (65 mg, 85% yield). ^{1}H NMR (600 MHz, $CDCl_3$) *δ* 7.64 – 7.61 (m, 2H), 7.46 (dd, *J* = 7.5, 1.8 Hz, 2H), 7.39 – 7.23 (m, 8H), 6.95 – 6.91 (m, 2H), 3.95 – 3.87 (m, 1H), 3.85 (s, 3H), 3.50 (dd, *J* = 20.1, 13.6 Hz, 1H), 2.98 (s, 3H). ^{13}C

{^{1}H} (151 MHz, $CDCl_3$) δ 163.0, 142.6 (d, J_{FC} = 23.1 Hz), 141.9 (d, J_{FC} = 23.1 Hz) 130.7, 127.9, 127.6, 127.5, 126.6 (d, J_{FC} = 7.2 Hz), 126.1 (d, J_{FC} = 7.2 Hz), 114.4, 99.2 (d, J_{FC} = 178.7 Hz), 55.6, 51.4 (d, J_{FC} = 24.5 Hz), 45.4. ^{19}F NMR (564 MHz, $CDCl_3$) δ –149.63 (t, J = 21.9 Hz). MS (EI): *m/z* (%) = 363 (2), 198 (100), 185 (8), 183 (7), 171 (33), 165 (13), 155 (27), 77 (2). IR (ATR): v = 3062, 3028, 2904, 2843, 1591, 1493, 1448, 1308, 1251, 1142, 1090, 1023, 984, 835, 761, 728, 699 cm^{-1}. HRMS *m/z*: calcd for $[C_{22}H_{22}FNO_2S+Na]^+$: 406.1247, found: 406.1247.

[(1-Fluoro-1,2,3,4-tetrahydronaphthalen-2-yl)imino](4-methoxyphenyl)(methyl)-λ^6-sulfanon (7fw)

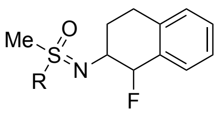

R = 4-MeO-Ph

Following the general procedure afforded the product as a yellow viscous oil (49 mg, 73% yield). dr = 1 : 2.2. ^{1}H NMR (600 MHz, $CDCl_3$, mixture of diastereomers) δ 7.94 – 7.91 (m, 4.4H), 7.91 – 7.89 (m, 2H), 7.43 (m, 3.4H), 7.24 – 7.21 (m, 2H), 7.21 – 7.17 (m, 4.4H), 7.11 – 7.10 (m, 1H), 7.07 (dd, J = 5.3, 3.6 Hz, 2.4H), 7.04 – 6.99 (m, 6.8H), 5.40 (m, 3.4H), 3.88 (s, 3H), 3.86 (s, 6.9H), 3.64 – 3.59 (m, 1H), 3.46 (dddd, J = 13.9, 10.6, 7.4, 3.4 Hz, 2.2H), 3.11 (s, 6.9H), 3.08 (s, 3H), 3.01 – 2.94 (m, 1H), 2.90 – 2.84 (m, 2.2H), 2.82 – 2.75 (m, 3.2H), 2.14 (dqd, J = 14.8, 4.9, 3.3 Hz, 2.2H), 1.94 (dtd, J = 13.4, 10.2, 5.4 Hz, 2.2H), 1.87 – 1.81 (m, 1H). ^{13}C {^{1}H} (151 MHz, $CDCl_3$, mixture of diastereomers) δ 163.3, 163.2, 137.2 (d, J_{FC} = 4.0 Hz), 137.1 (d, J_{FC} = 4.0 Hz), 134.1 (d, J_{FC} = 17.8 Hz), 133.6 (d, J_{FC} = 17.8 Hz), 131.3, 130.5, 130.2, 129.2, 129.2, 128.4, 128.3, 128.2, 128.1, 128.1, 128.0, 126.1, 126.0, 114.5, 114.5, 94.3 (d, J_{FC} = 175.7 Hz), 93.4 (d, J_{FC} = 173.6 Hz), 55.6 (d, J_{FC} = 19.0 Hz), 55.3 (d, J_{FC} = 18.6 Hz), 54.0, 53.9, 46.3, 45.1, 30.4 (d, J_{FC} = 5.6 Hz), 28.6 (d, J_{FC} = 3.3 Hz), 27.3, 26.6. ^{19}F NMR (564 MHz, $CDCl_3$, mixture of diastereomers) δ –162.82 (dd, J = 51.5, 13.4 Hz), –169.75 (dd, J = 52.3, 14.0 Hz). MS (EI): *m/z* (%) = 333 (28), 270 (7), 250 (33), 234 (5), 190 (6), 171 (20), 155 (100), 143 (7), 135 (9), 122 (8), 115 (10), 77 (4). IR (ATR): v = 3465, 3017, 2927, 2842, 2246, 1590, 1493, 1457, 1310, 1250, 1130, 1090, 1024, 984, 909, 836, 804, 770, 730 cm^{-1}. HRMS *m/z*: calcd for $[C_{18}H_{20}FNO_2S+Na]^+$: 356.1091, found: 356.1090.

6. NMR Spectra

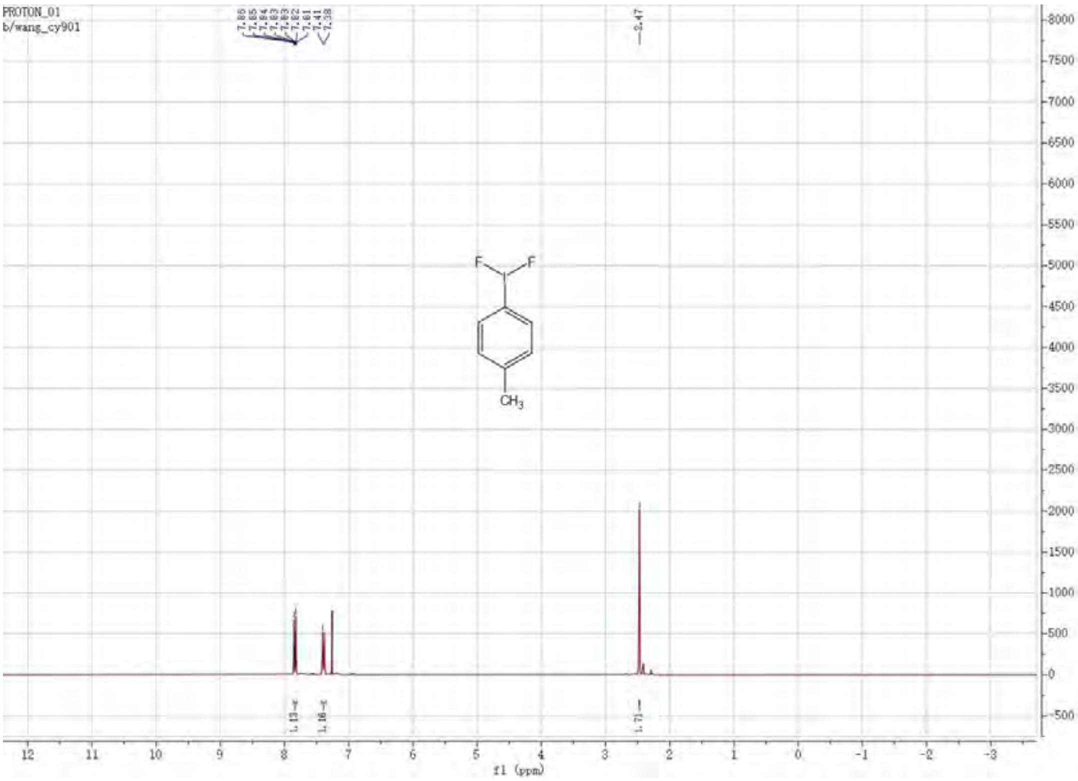

^{1}H NMR spectrum of compound **4a** (300 MHz, $CDCl_3$)

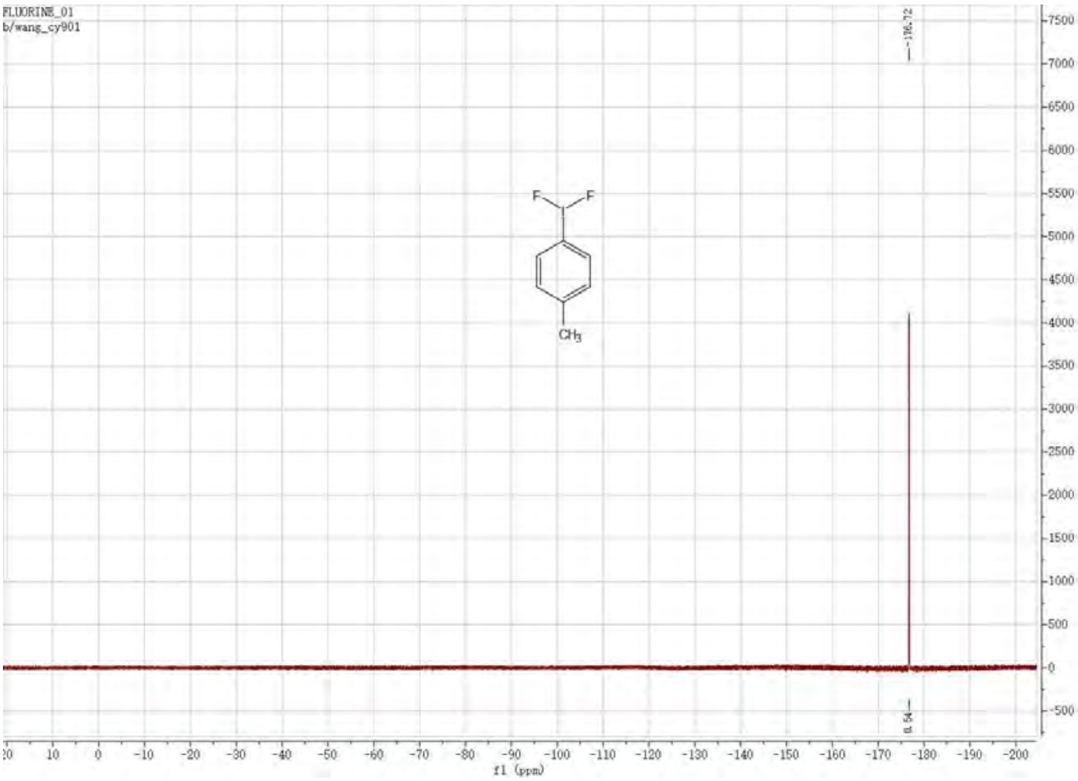

^{19}F NMR spectrum of compound **4a** (282 MHz, $CDCl_3$)

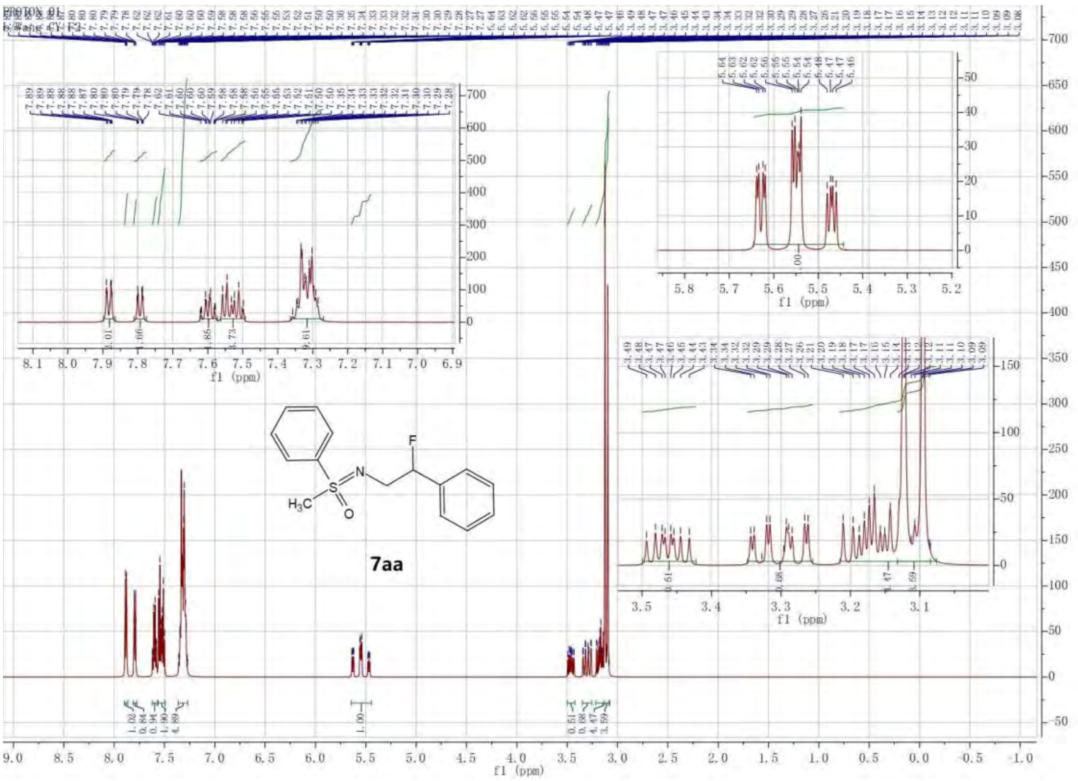

^{1}H NMR spectrum of compound **7aa** (600 MHz, $CDCl_3$)

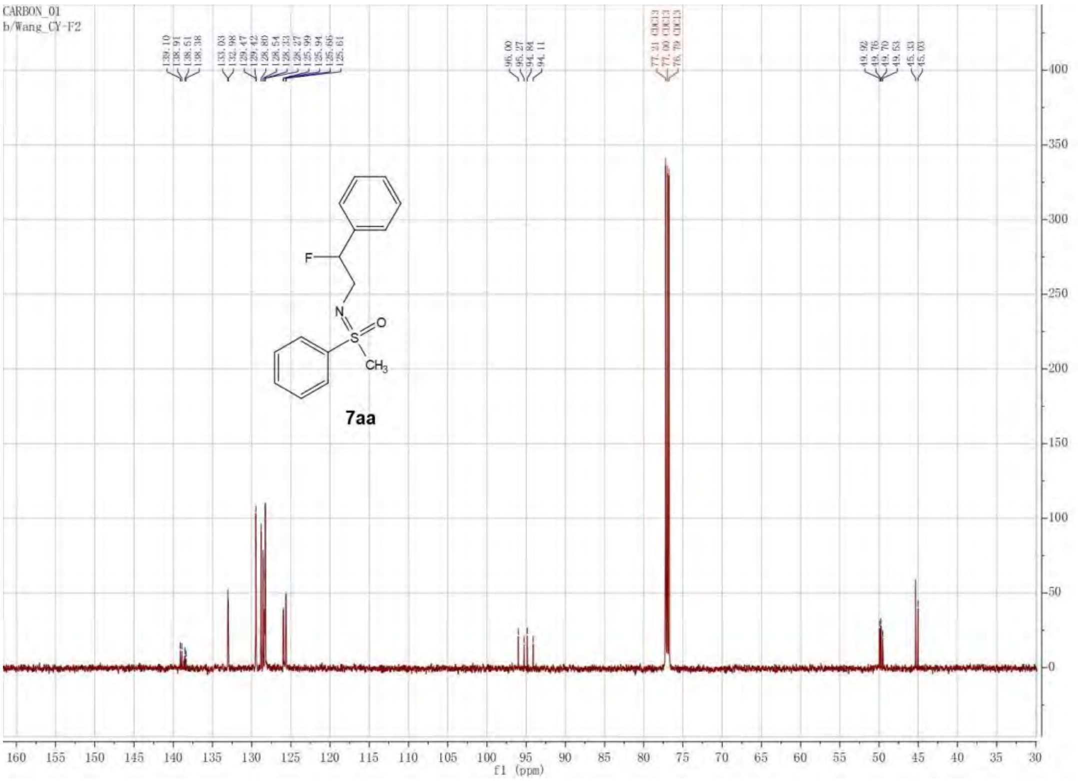

^{13}C NMR spectrum of compound **7aa** (151 MHz, CDCl3)

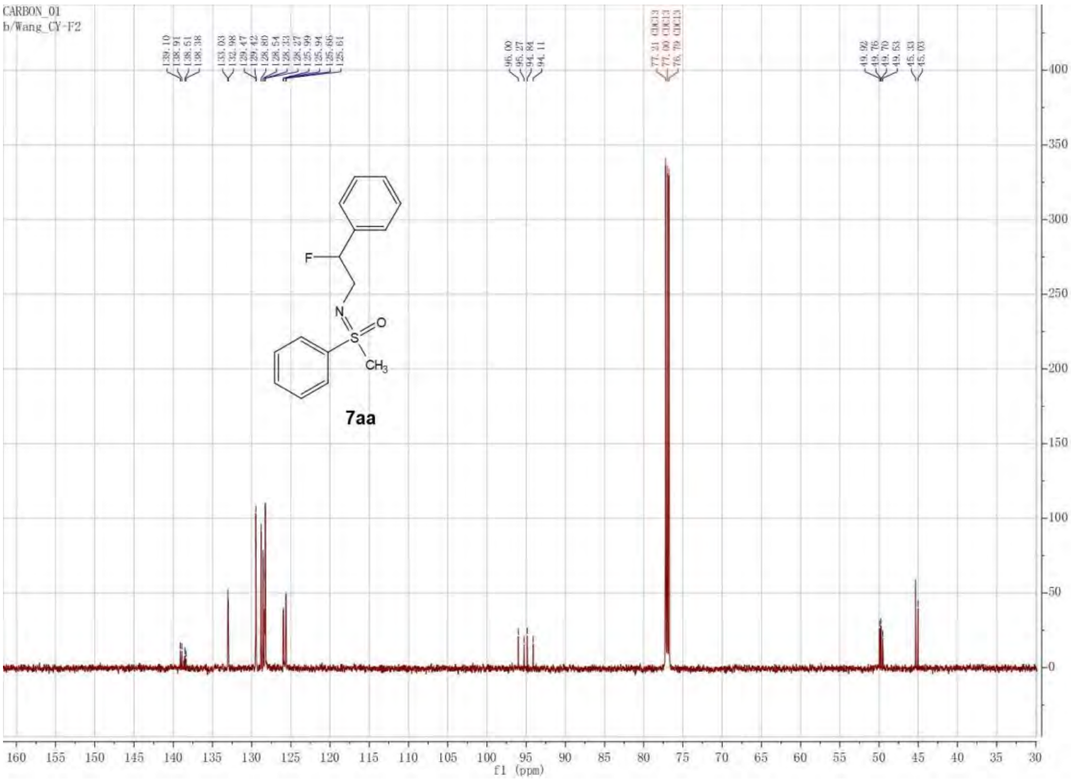

^{19}F NMR spectrum of compound **7aa** (564 MHz, $CDCl_3$)

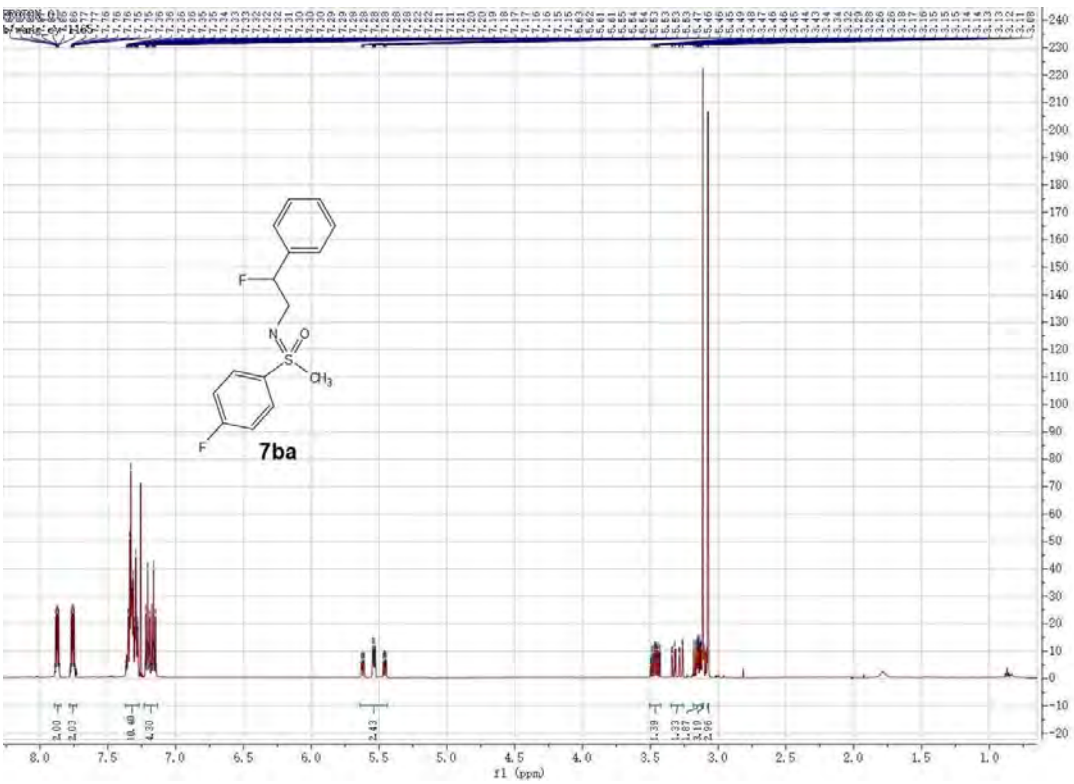

^{1}H NMR spectrum of compound **7ba** (600 MHz, $CDCl_3$)

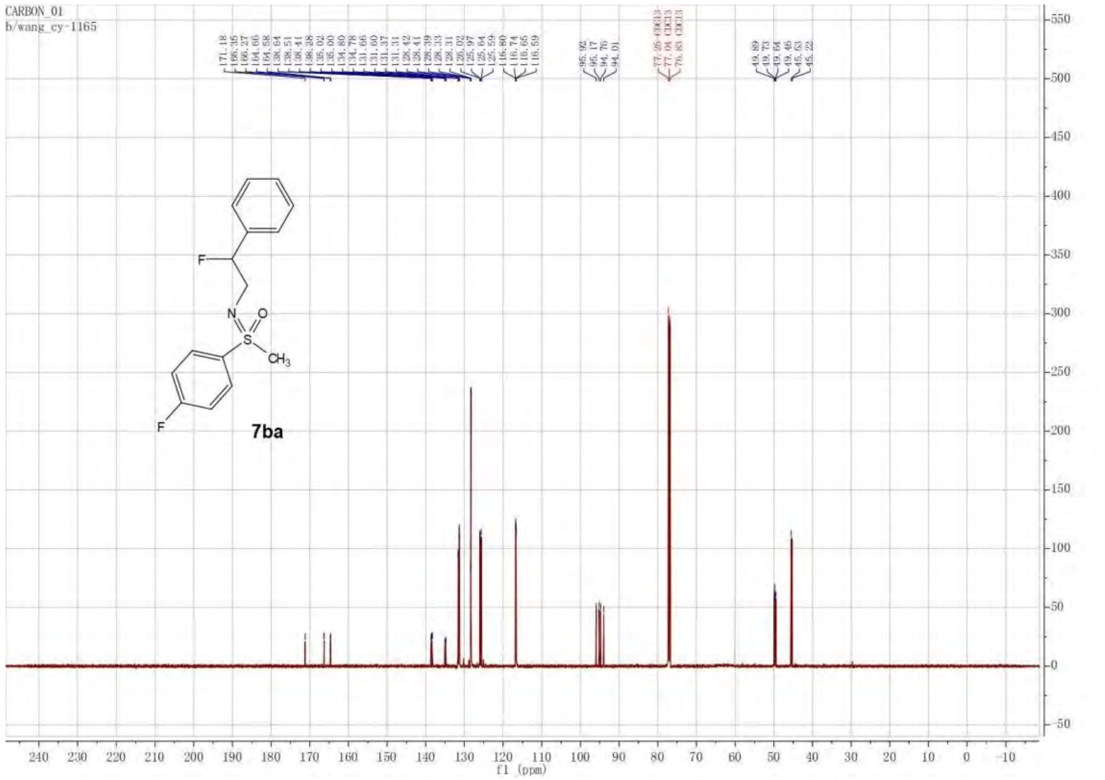

^{13}C NMR spectrum of compound **7ba** (151 MHz, $CDCl_3$)

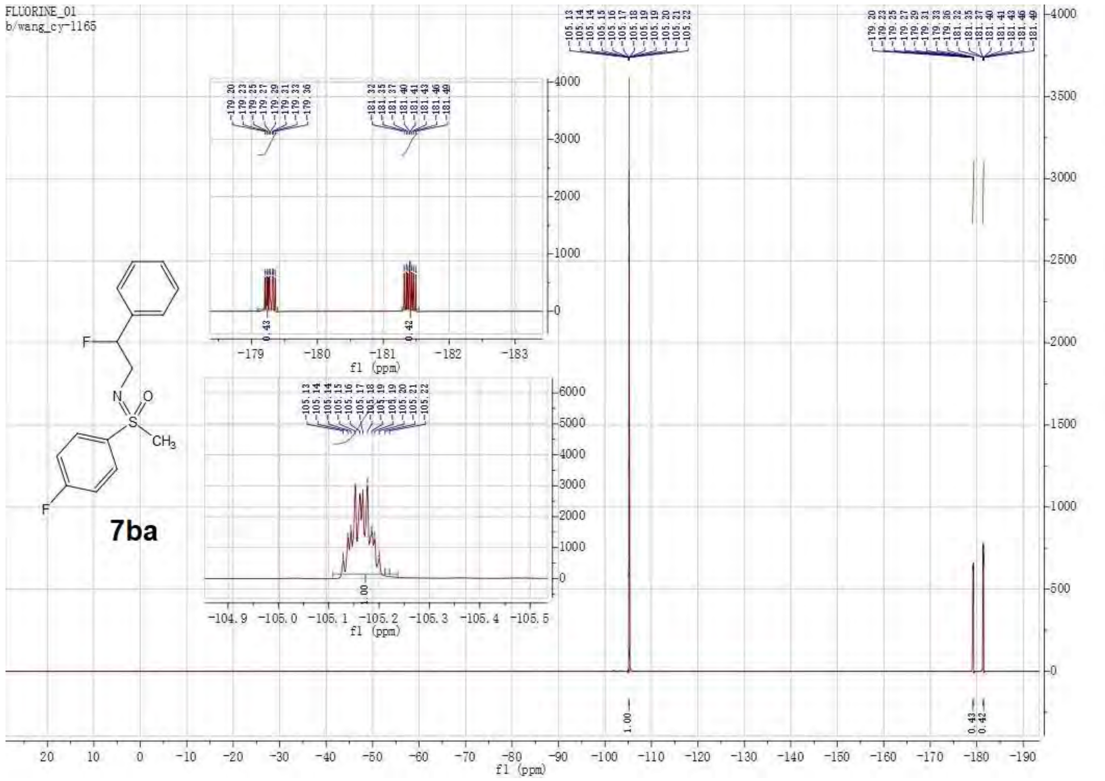

^{19}F NMR spectrum of compound **7ba** (564 MHz, $CDCl_3$)

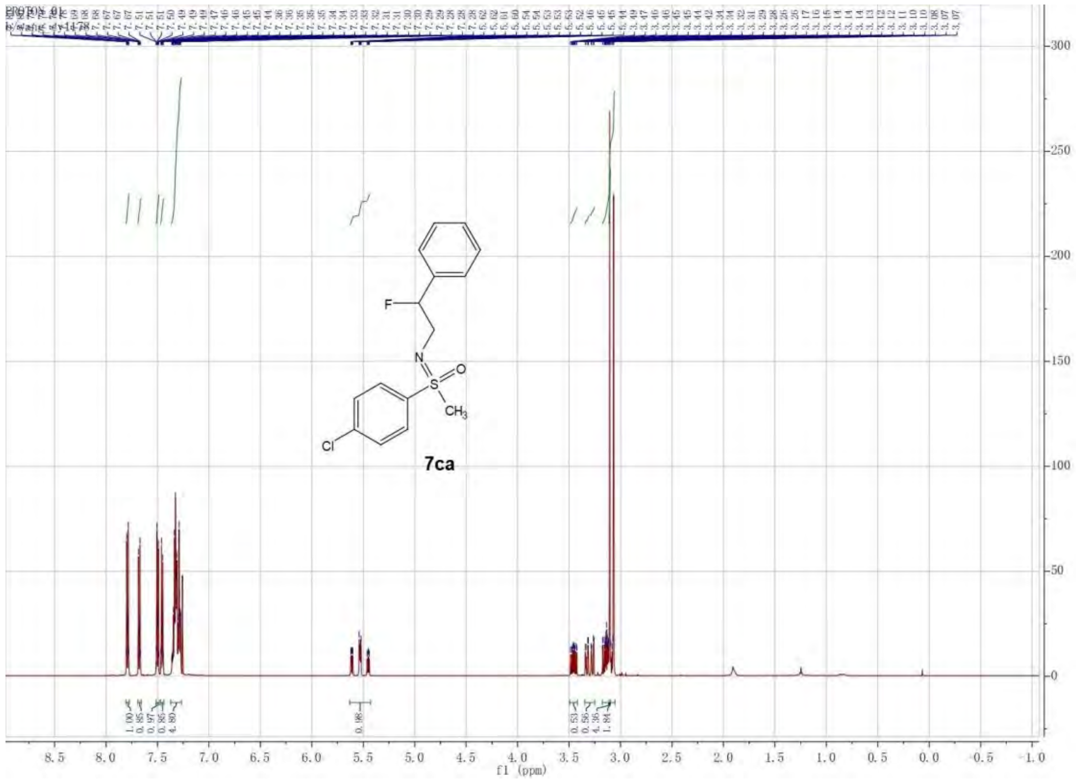

^{1}H NMR spectrum of compound **7ca** (600 MHz, $CDCl_3$)

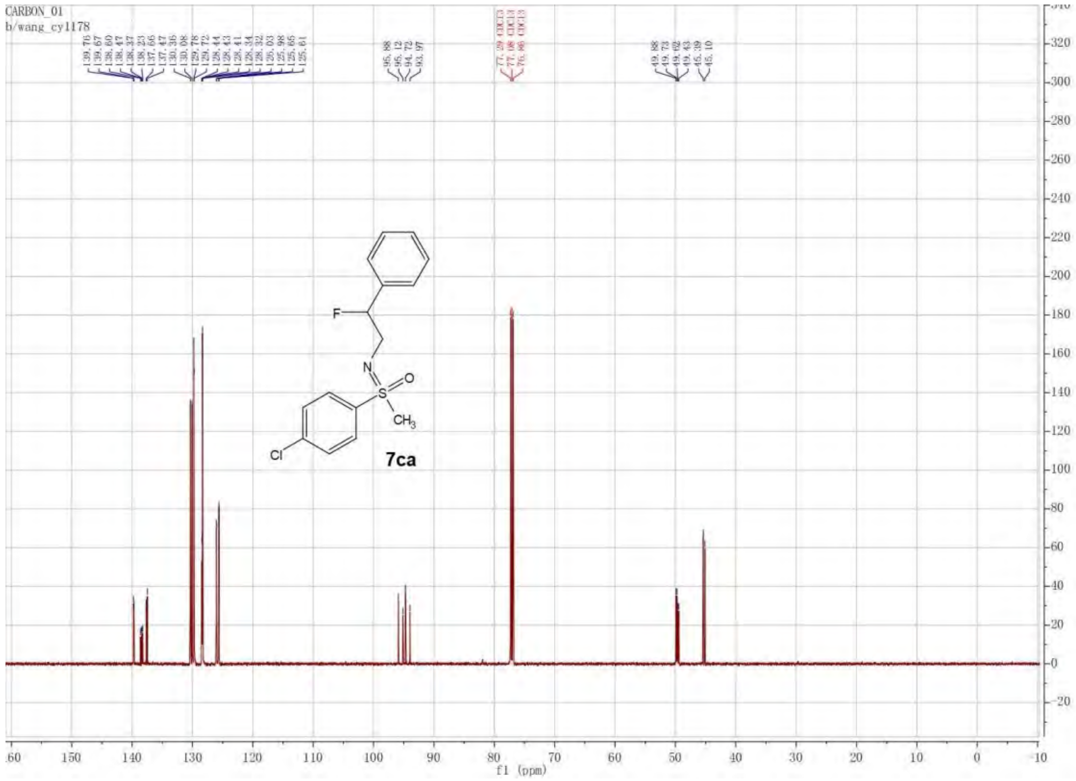

^{13}C NMR spectrum of compound **7ca** (151 MHz, $CDCl_3$)

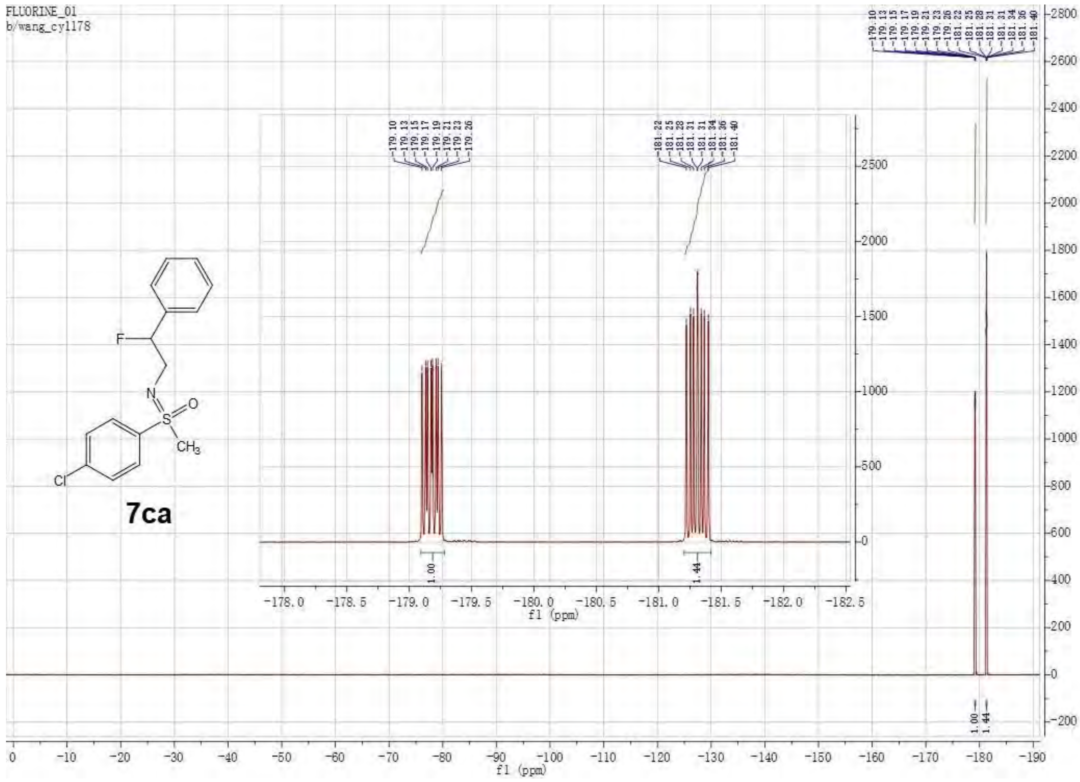

7ca

^{19}F NMR spectrum of compound **7ca** (564 MHz, $CDCl_3$)

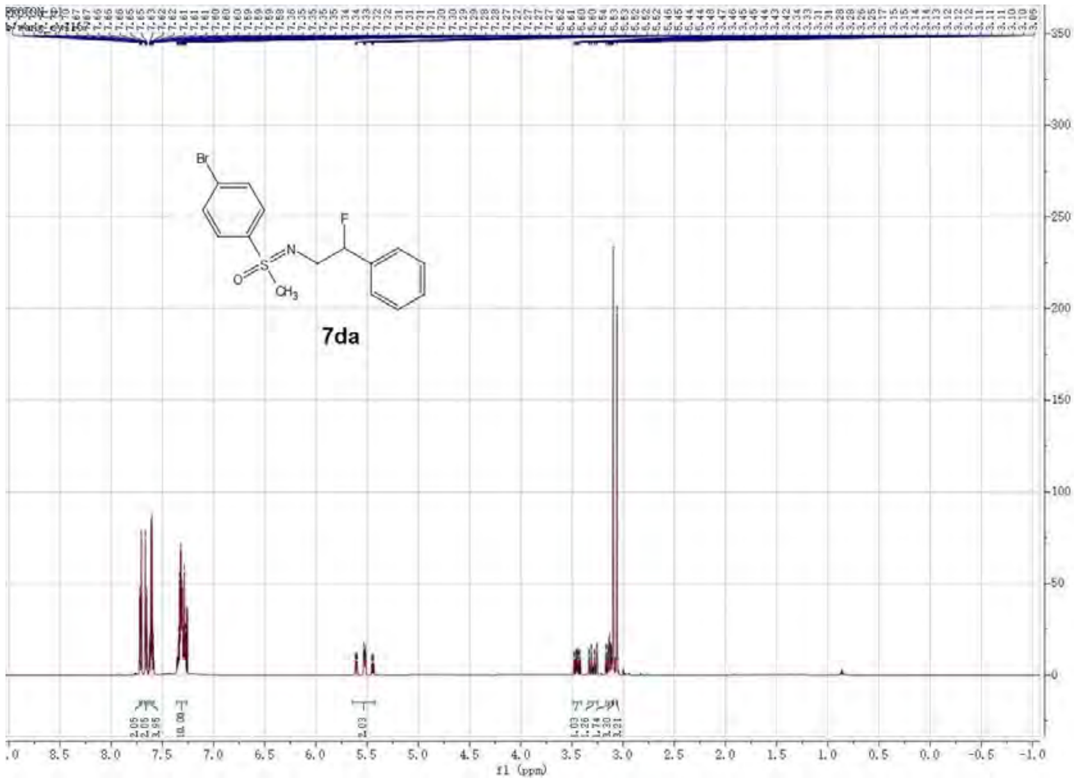

7da

^{1}H NMR spectrum of compound **7da** (600 MHz, $CDCl_3$)

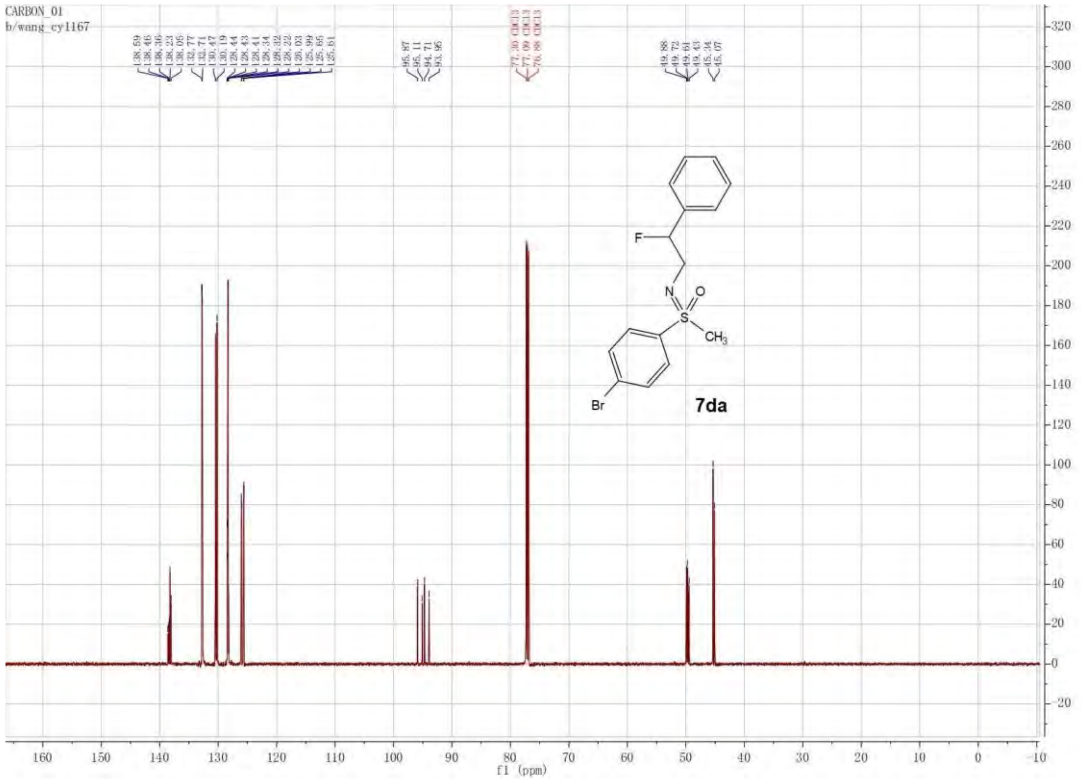

^{13}C NMR spectrum of compound **7da** (151 MHz, $CDCl_3$)

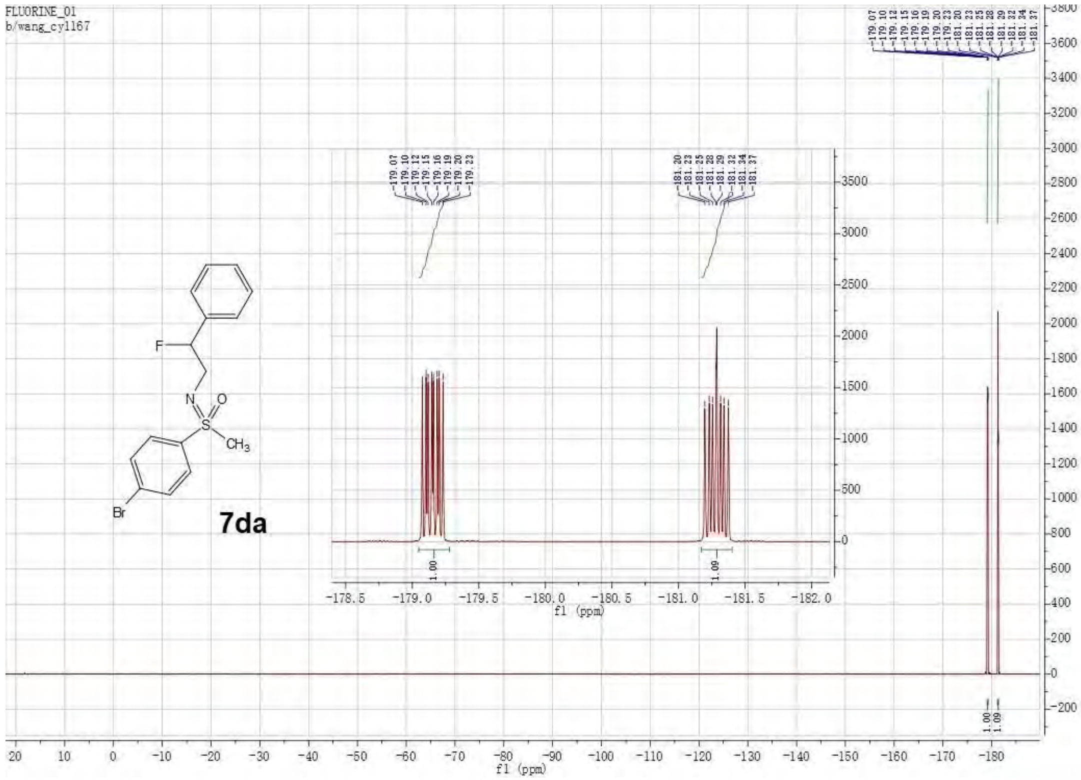

^{19}F NMR spectrum of compound **7da** (564 MHz, $CDCl_3$)

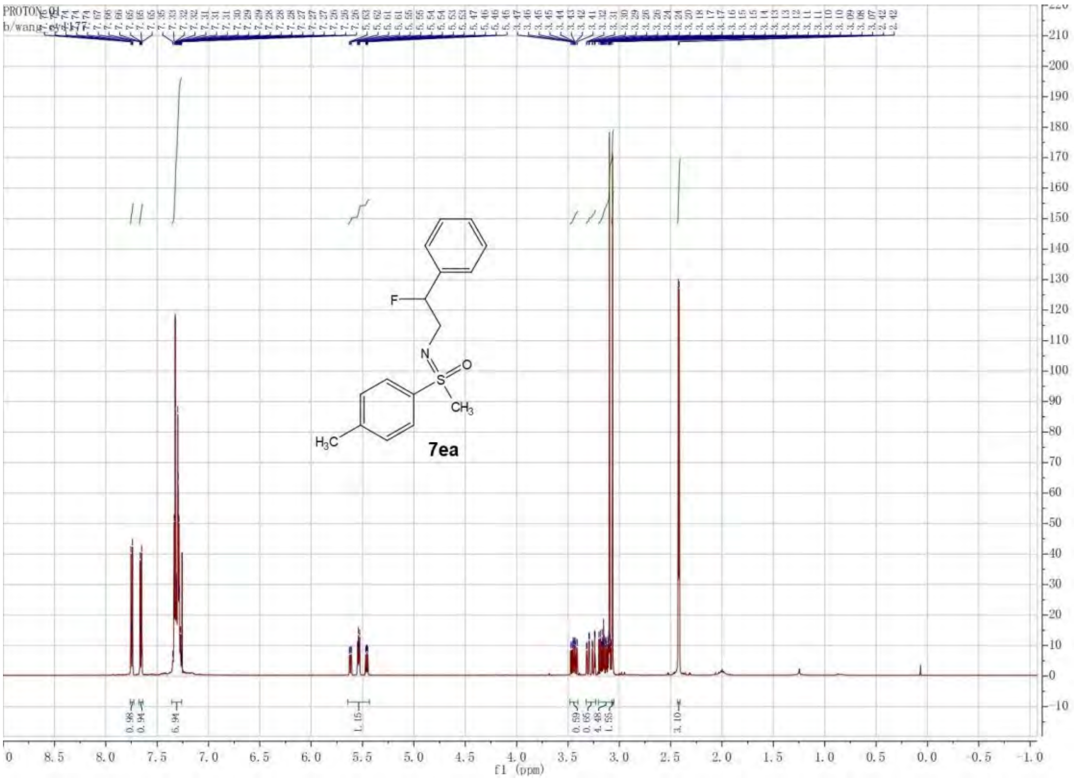

^{1}H NMR spectrum of compound **7ea** (600 MHz, $CDCl_3$)

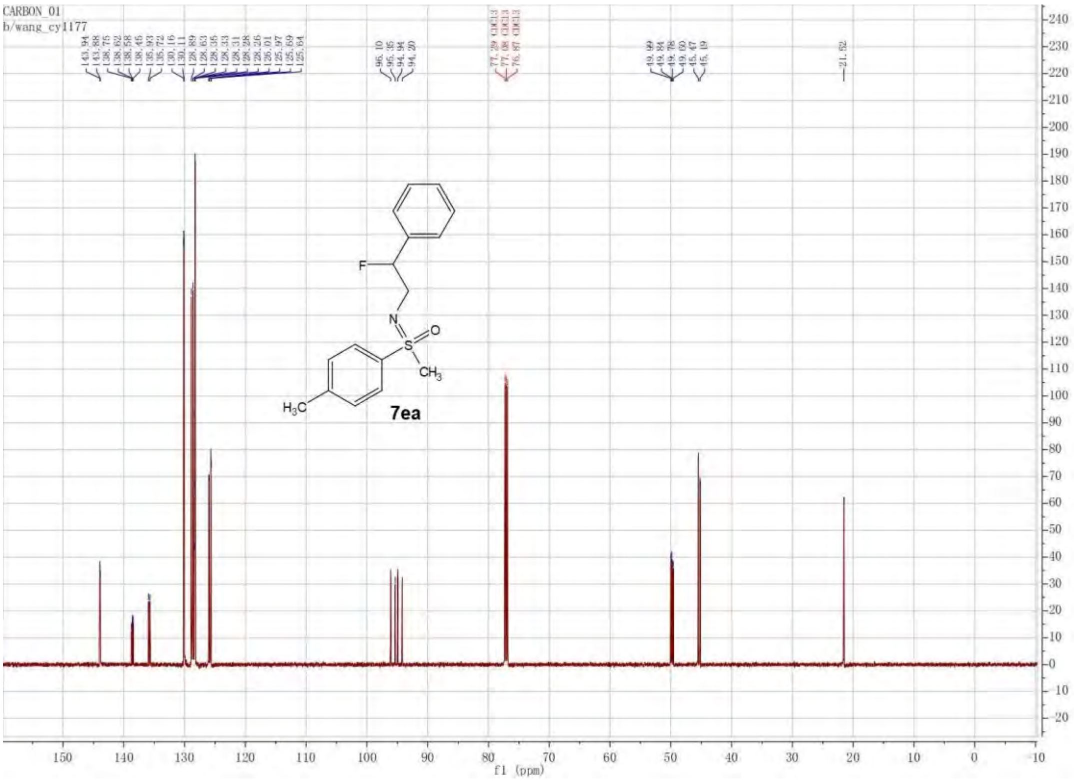

^{13}C NMR spectrum of compound **7ea** (151 MHz, $CDCl_3$)

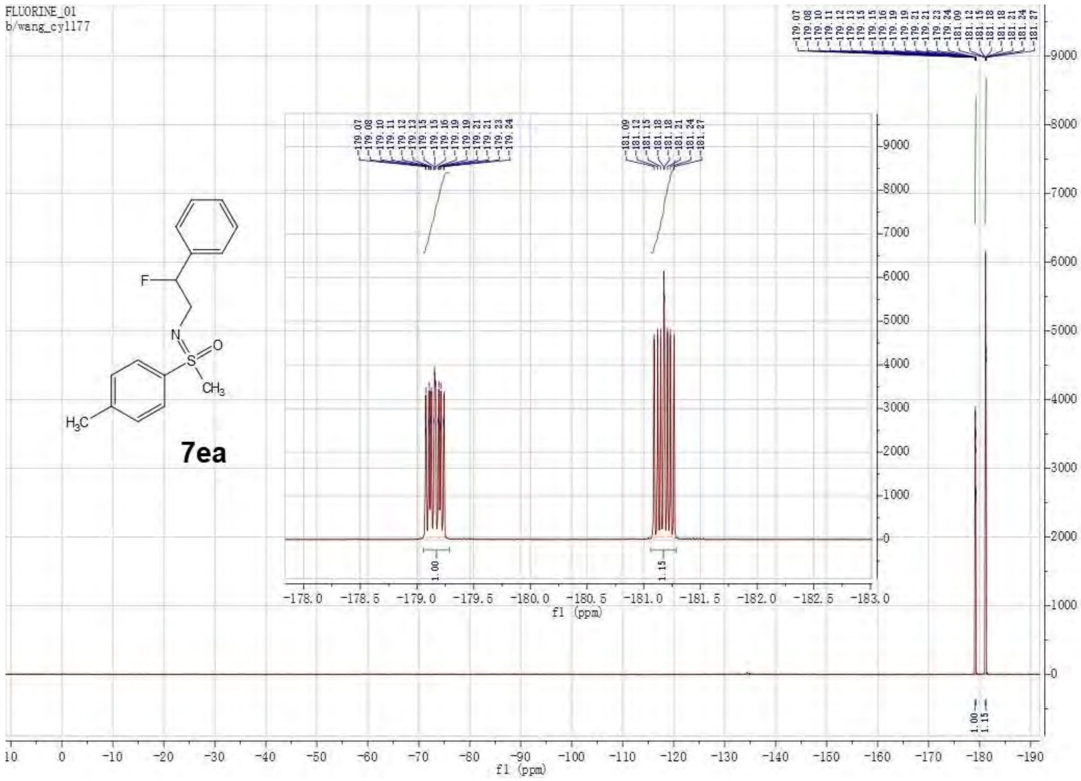

^{19}F NMR spectrum of compound **7ea** (564 MHz, $CDCl_3$)

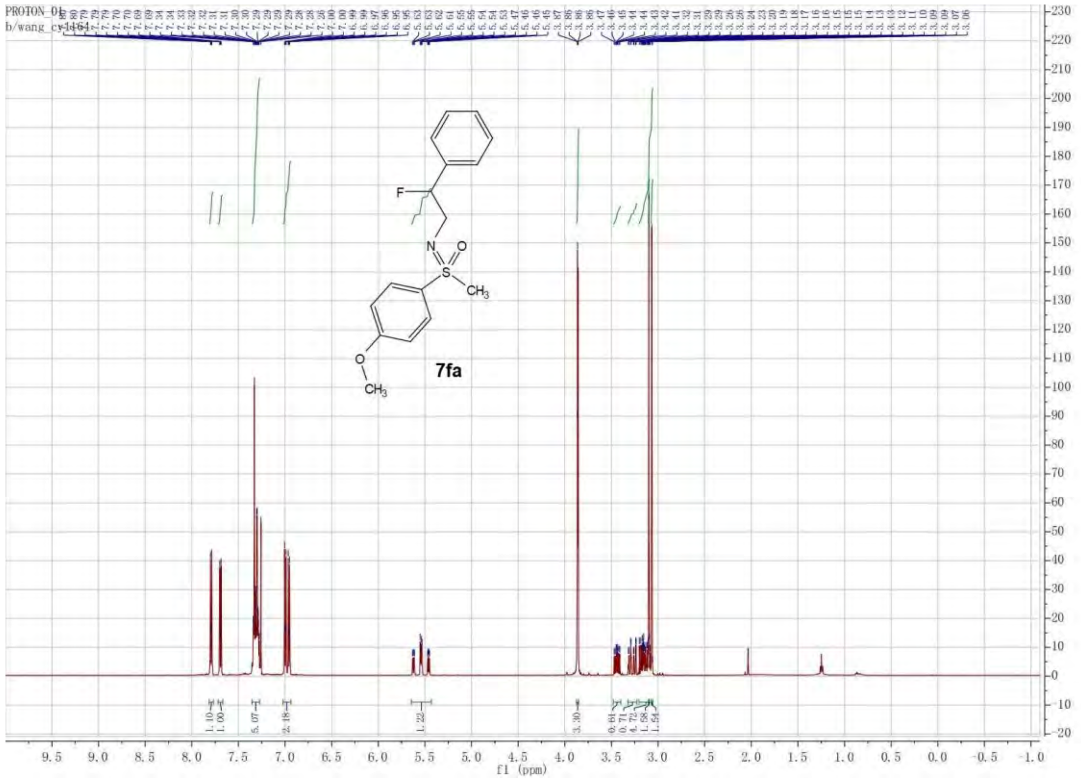

^{1}H NMR spectrum of compound **7fa** (600 MHz, $CDCl_3$)

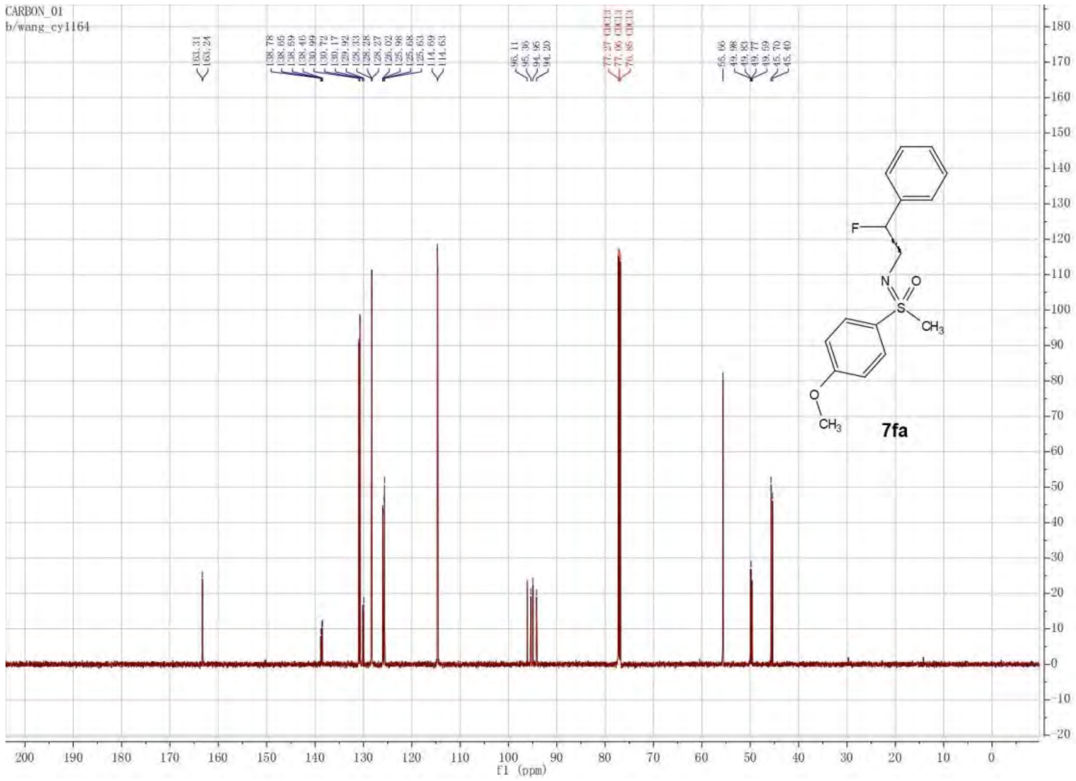

^{13}C NMR spectrum of compound **7fa** (151 MHz, $CDCl_3$)

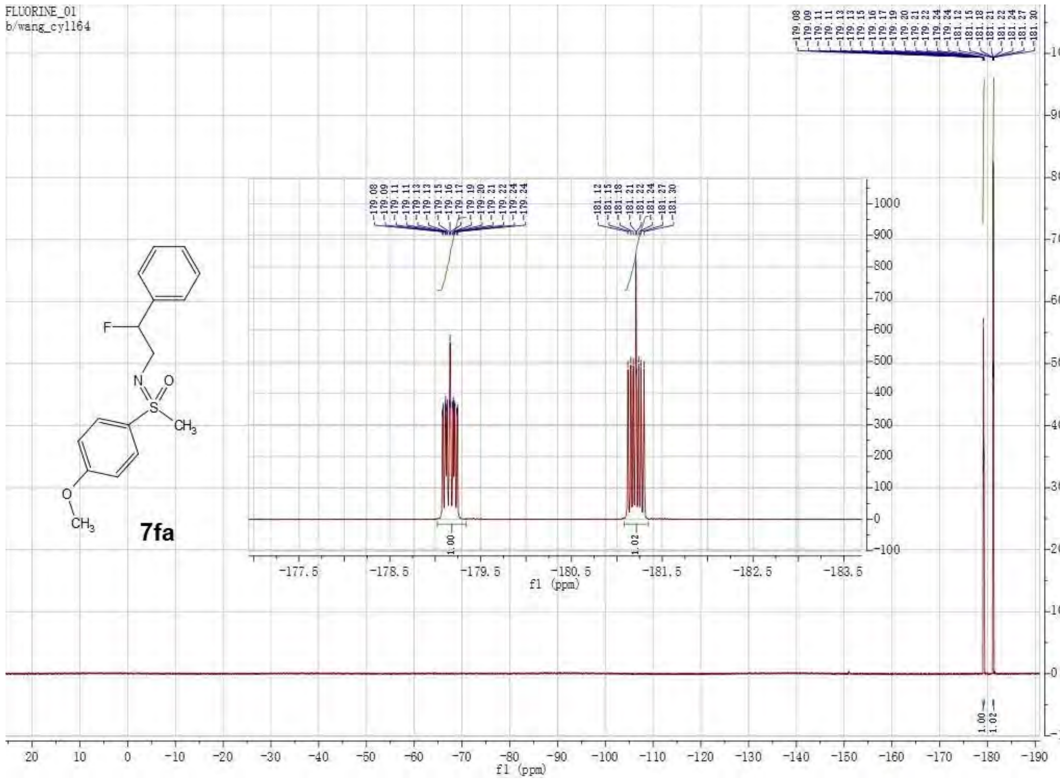

^{19}F NMR spectrum of compound **7fa** (564 MHz, $CDCl_3$)

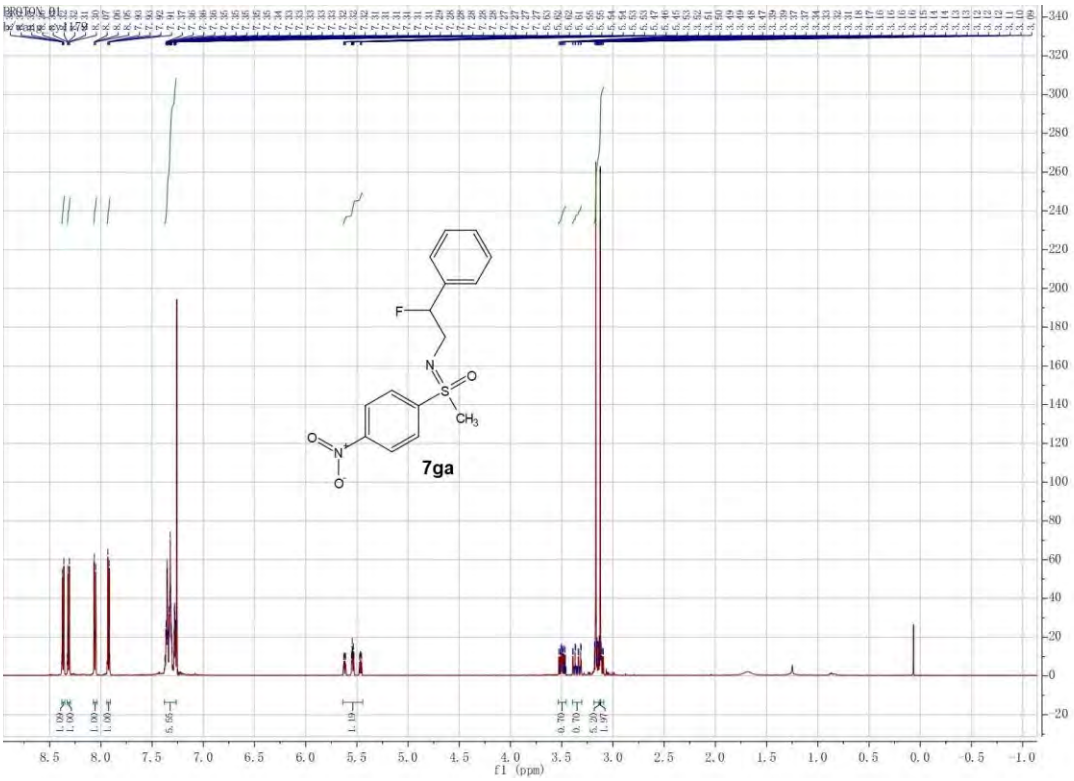

^{1}H NMR spectrum of compound **7ga** (600 MHz, $CDCl_3$)

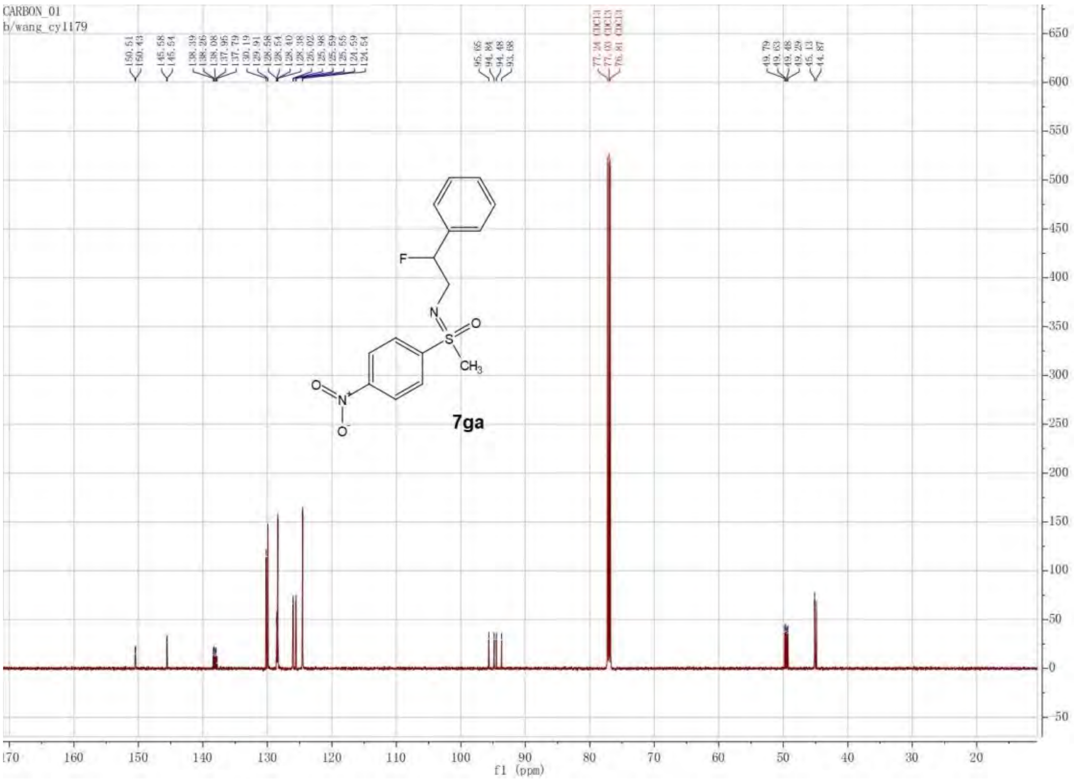

^{13}C NMR spectrum of compound **7ga** (151 MHz, $CDCl_3$)

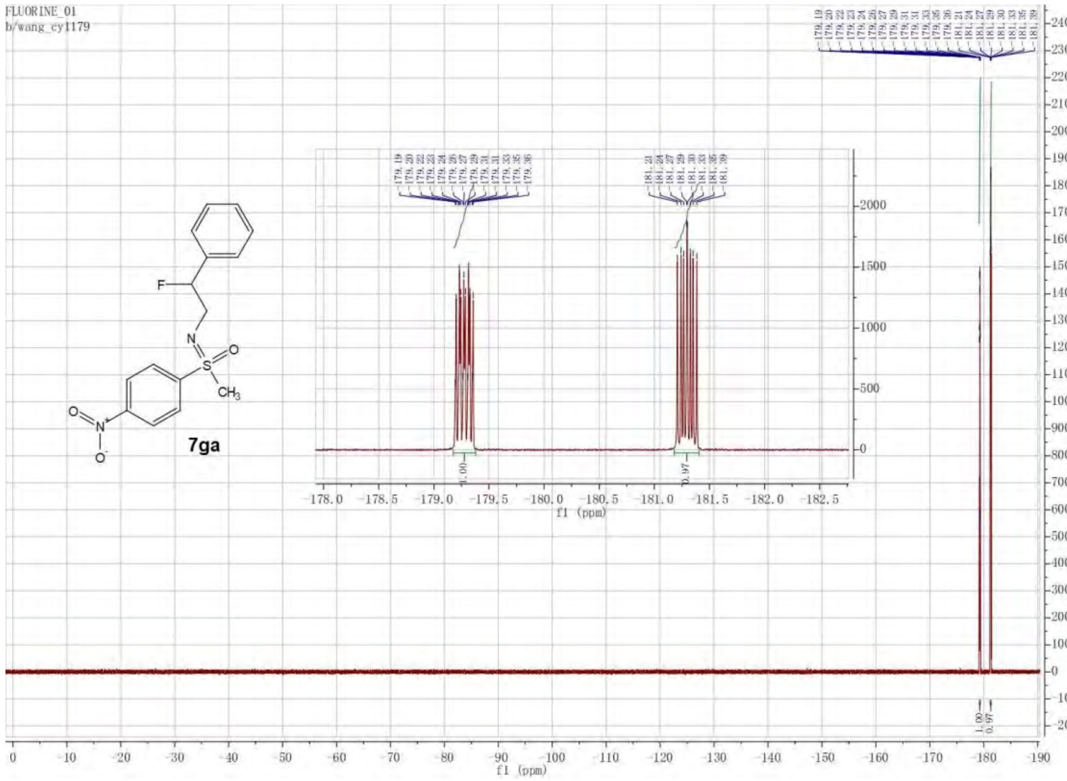

^{19}F NMR spectrum of compound **7ga** (564 MHz, $CDCl_3$)

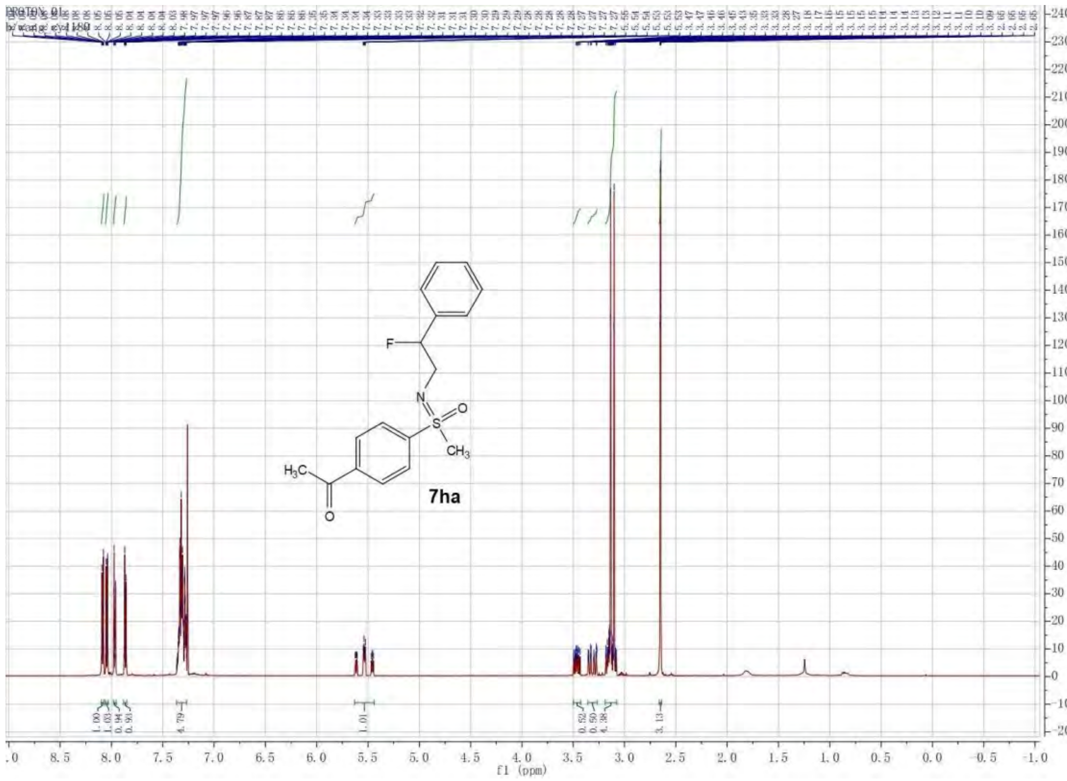

^{1}H NMR spectrum of compound **7ha** (600 MHz, $CDCl_3$)

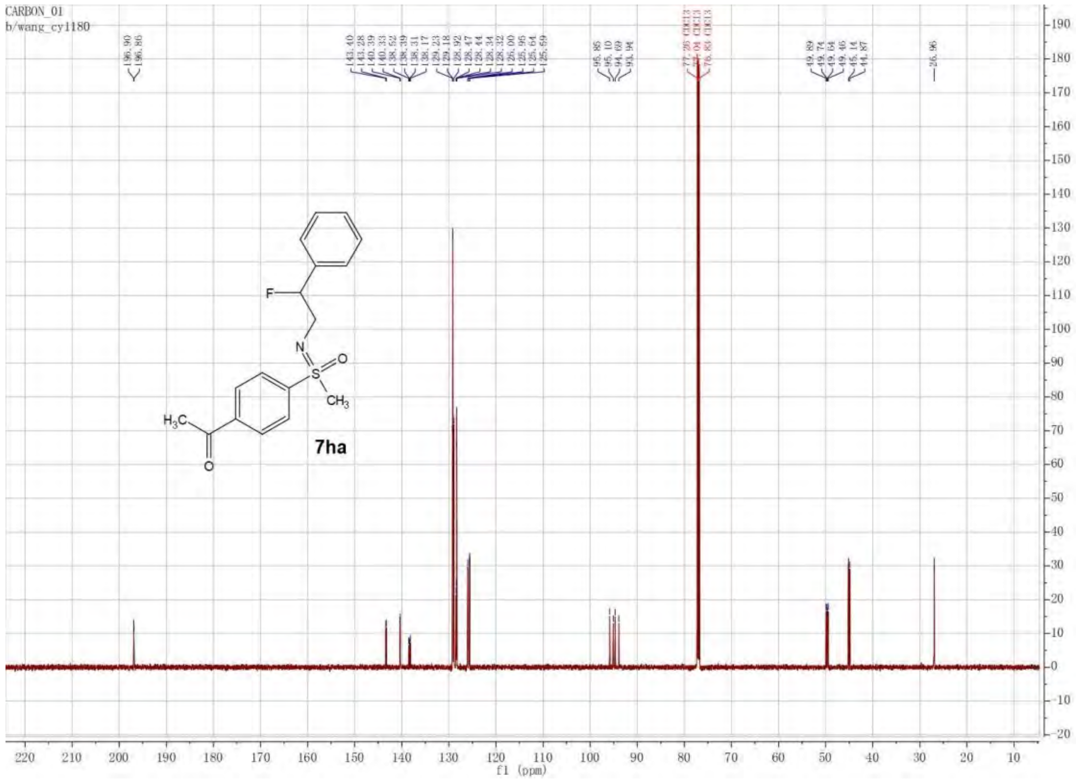

^{13}C NMR spectrum of compound **7ha** (151 MHz, $CDCl_3$)

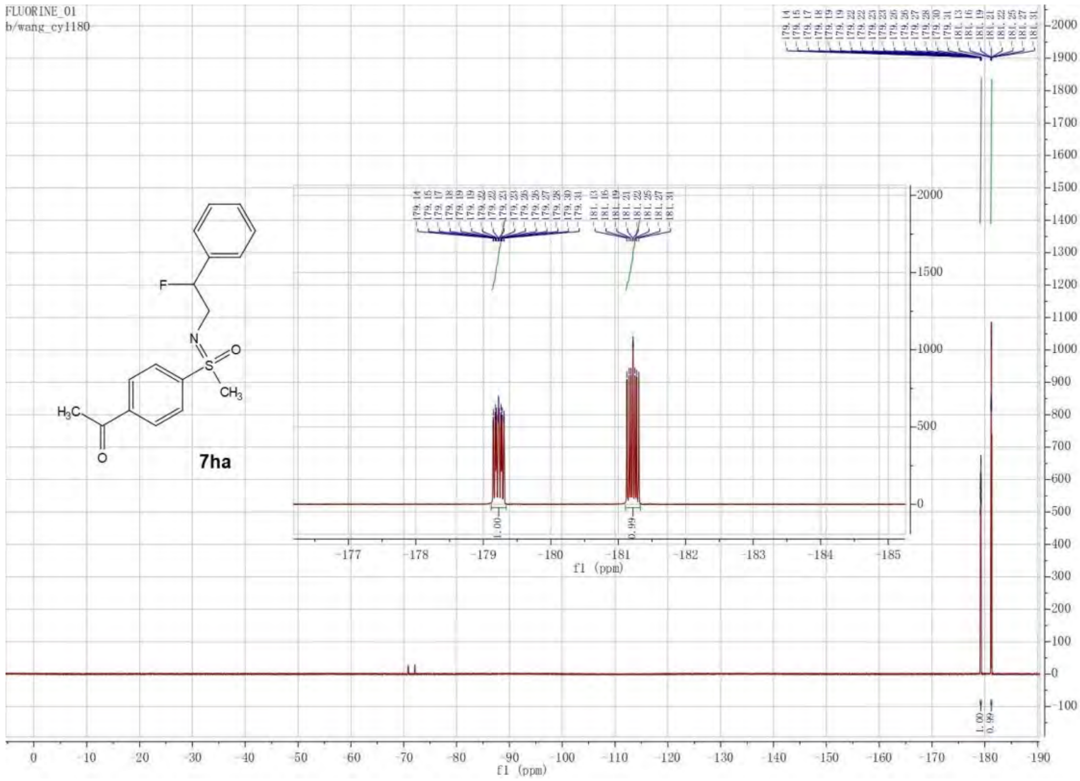

^{19}F NMR spectrum of compound **7ha** (564 MHz, $CDCl_3$)

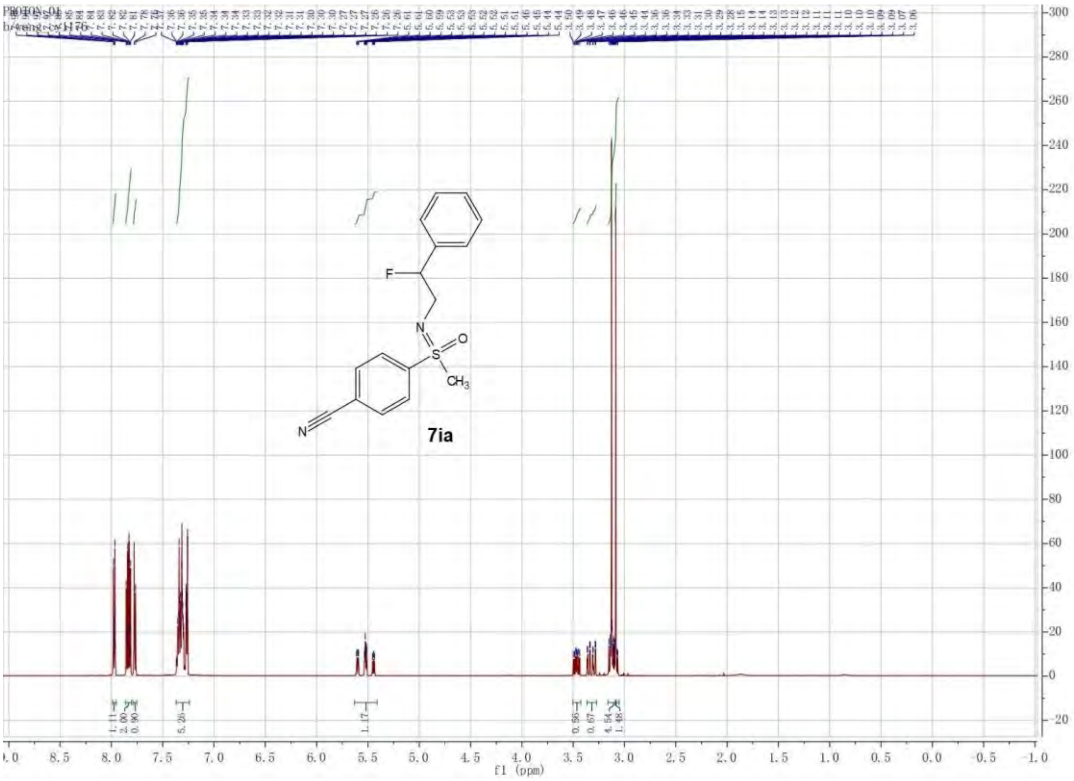

^{1}H NMR spectrum of compound **7ia** (600 MHz, $CDCl_3$)

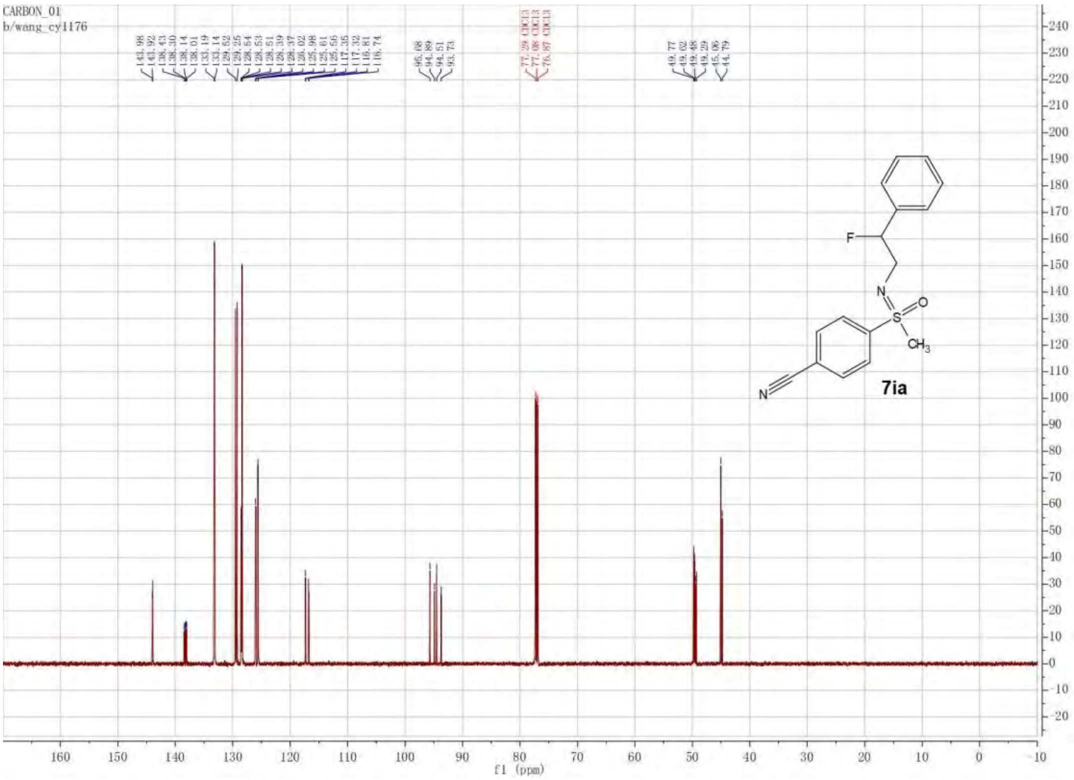

^{13}C NMR spectrum of compound **7ia** (151 MHz, $CDCl_3$)

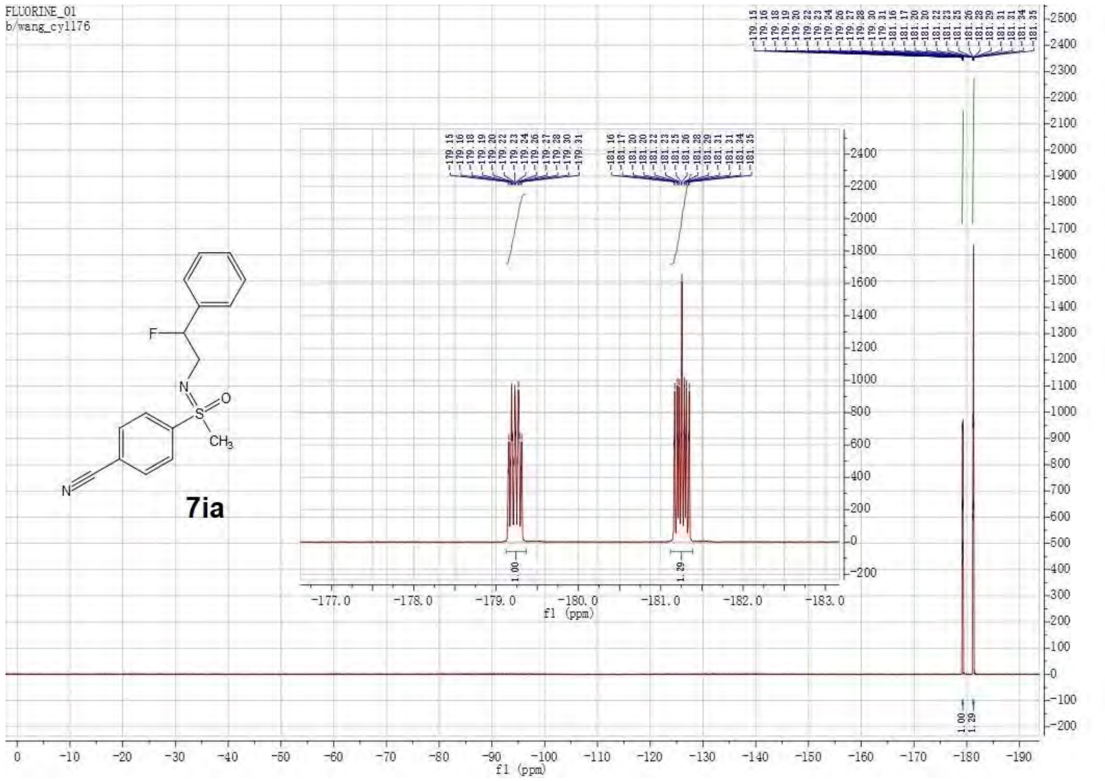

^{19}F NMR spectrum of compound **7ia** (564 MHz, $CDCl_3$)

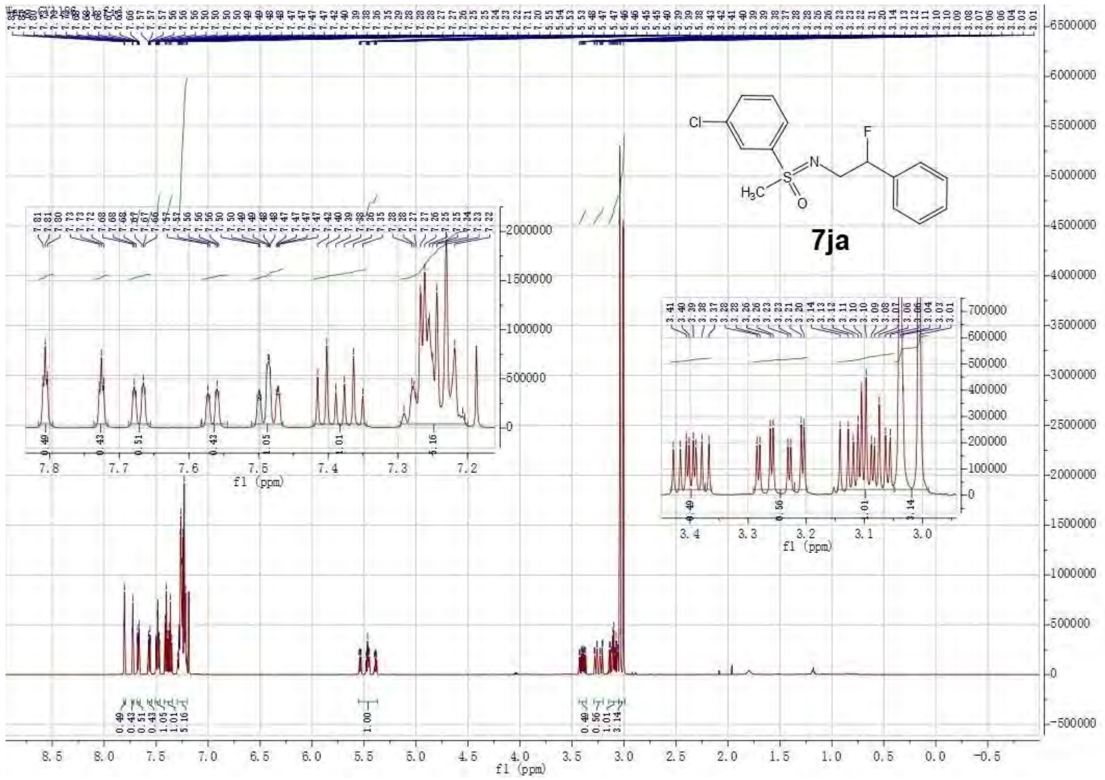

^{1}H NMR spectrum of compound **7ja** (600 MHz, $CDCl_3$)

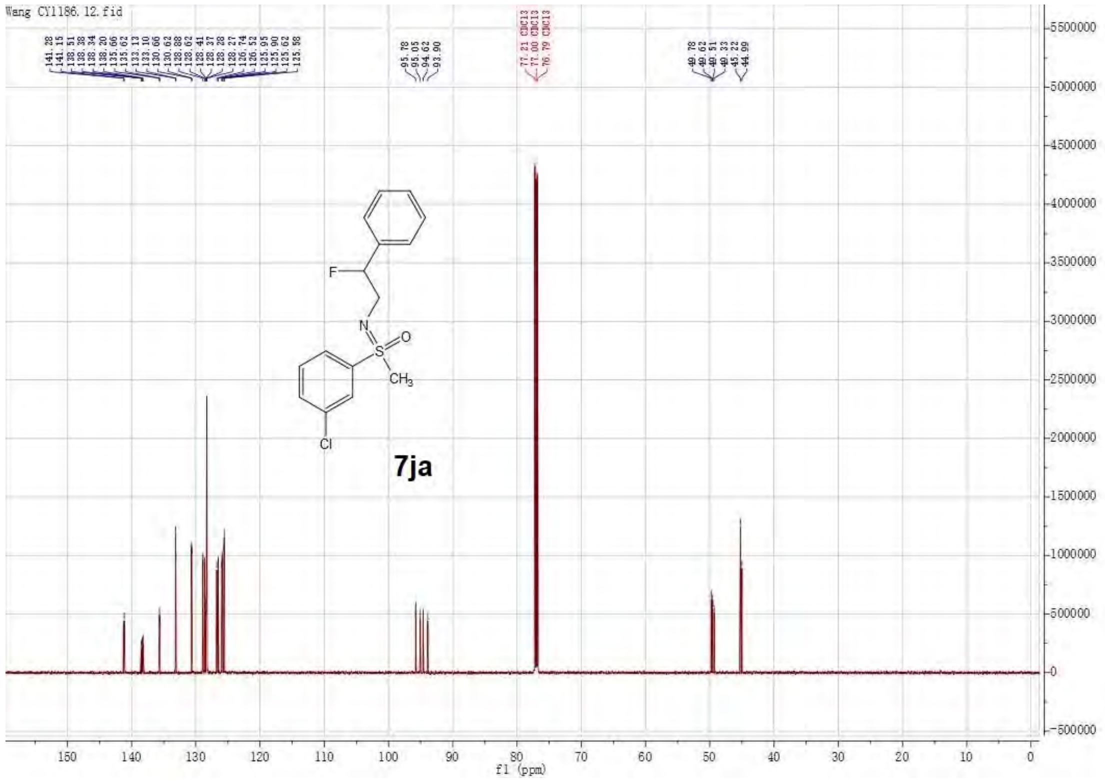

^{13}C NMR spectrum of compound **7ja** (151 MHz, $CDCl_3$)

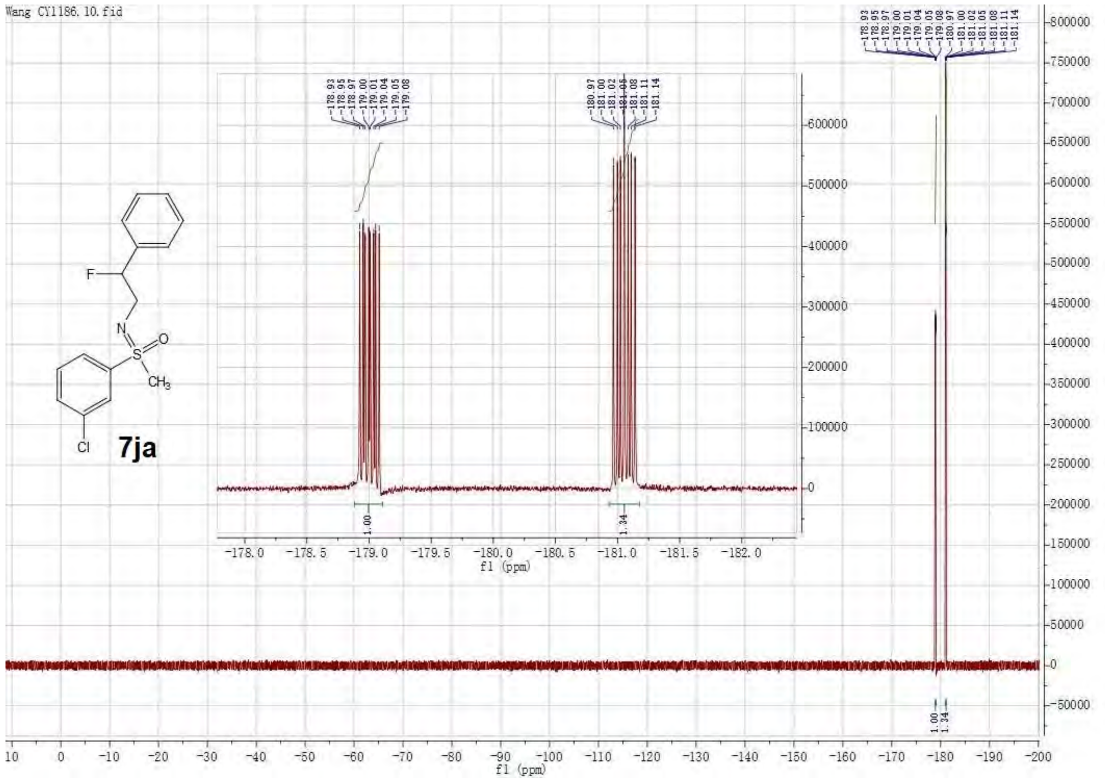

^{19}F NMR spectrum of compound **7ja** (564 MHz, $CDCl_3$)

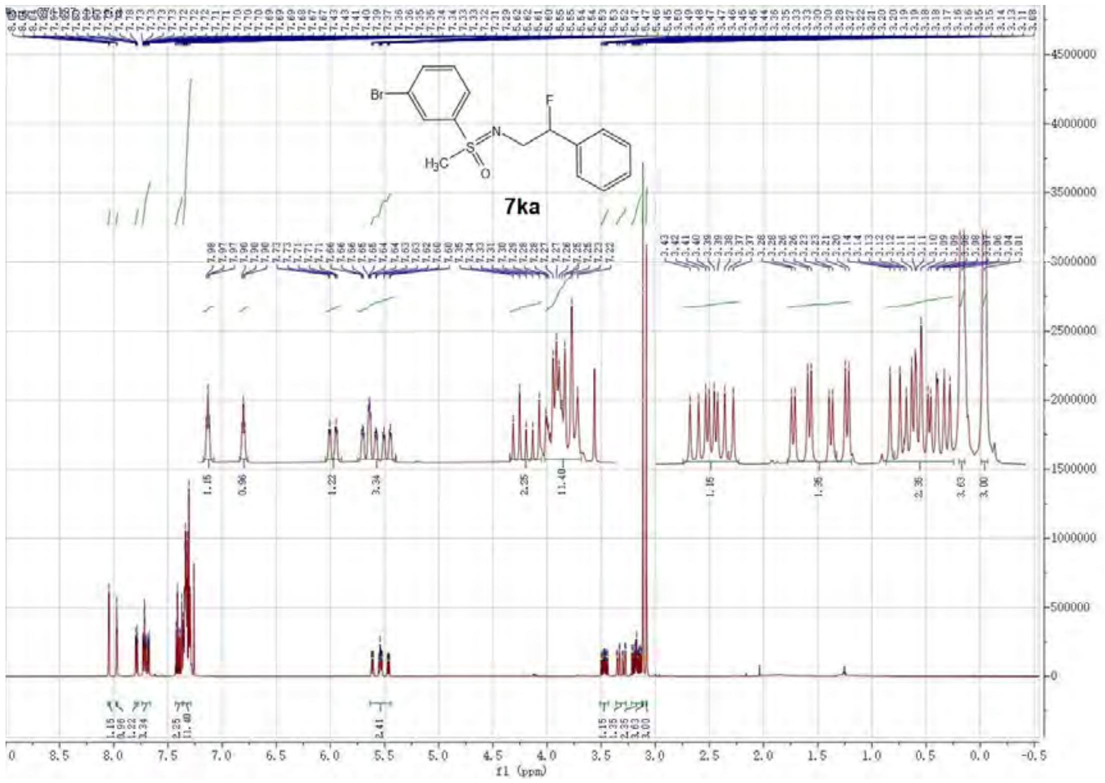

^{1}H NMR spectrum of compound **7ka** (600 MHz, $CDCl_3$)

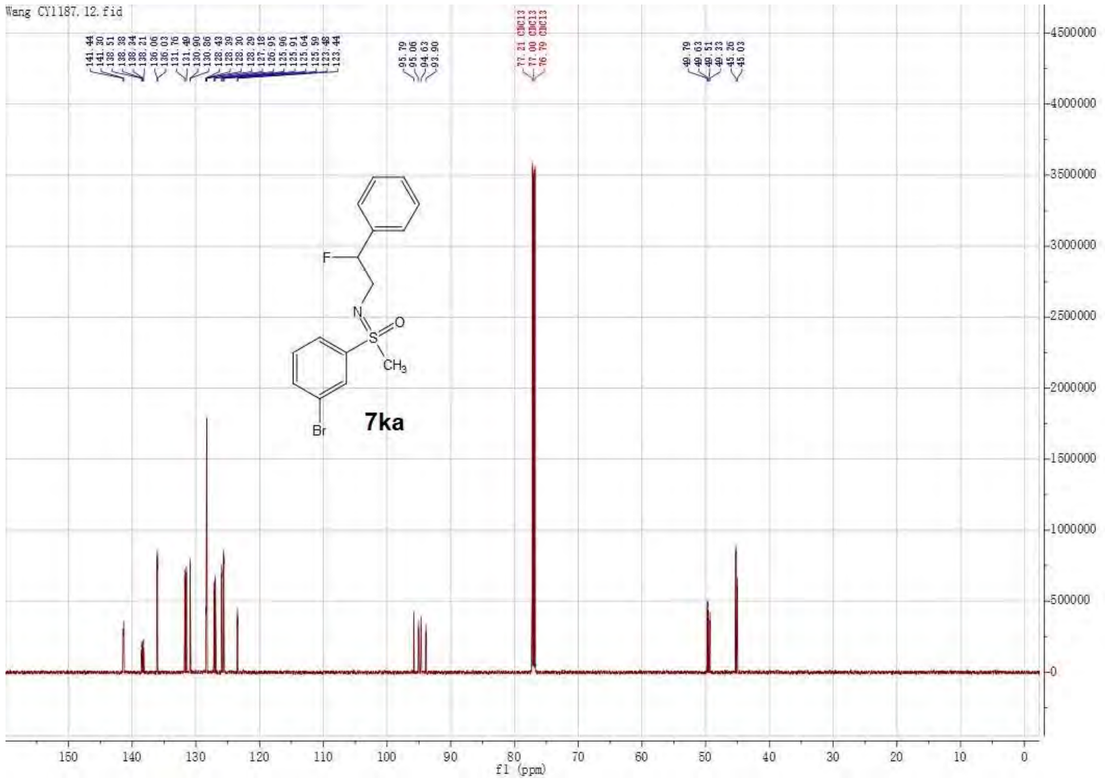

^{13}C NMR spectrum of compound **7ka** (151 MHz, $CDCl_3$)

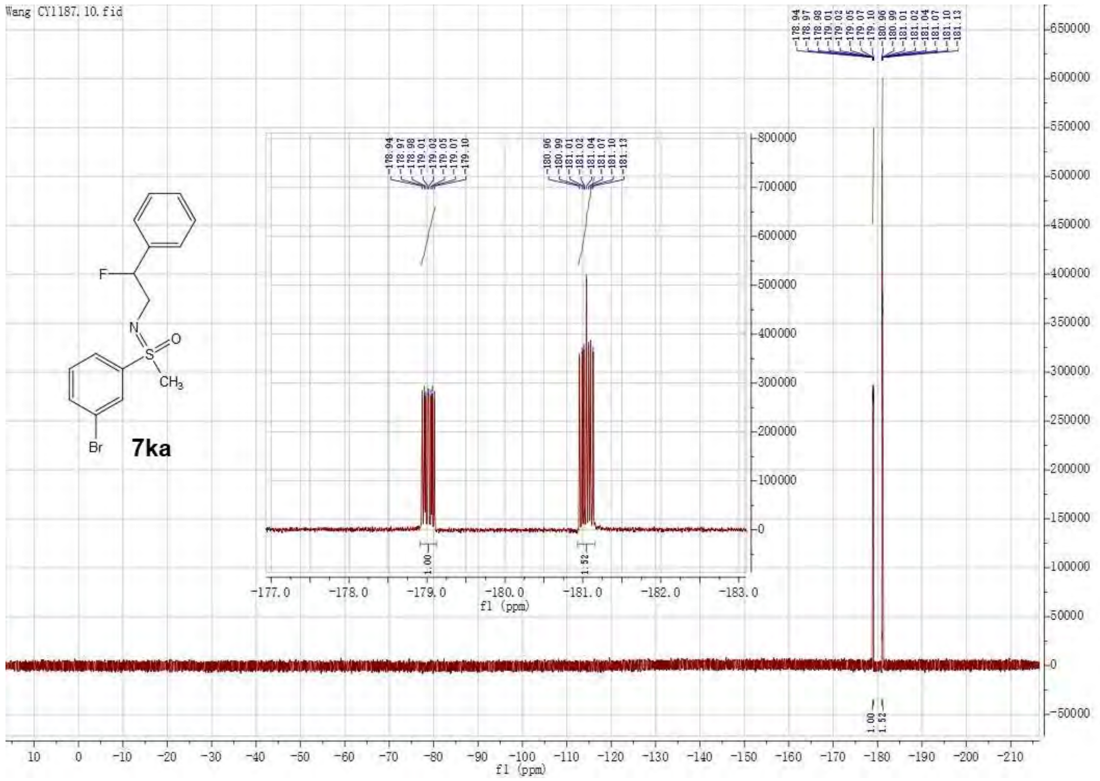

^{19}F NMR spectrum of compound **7ka** (564 MHz, $CDCl_3$)

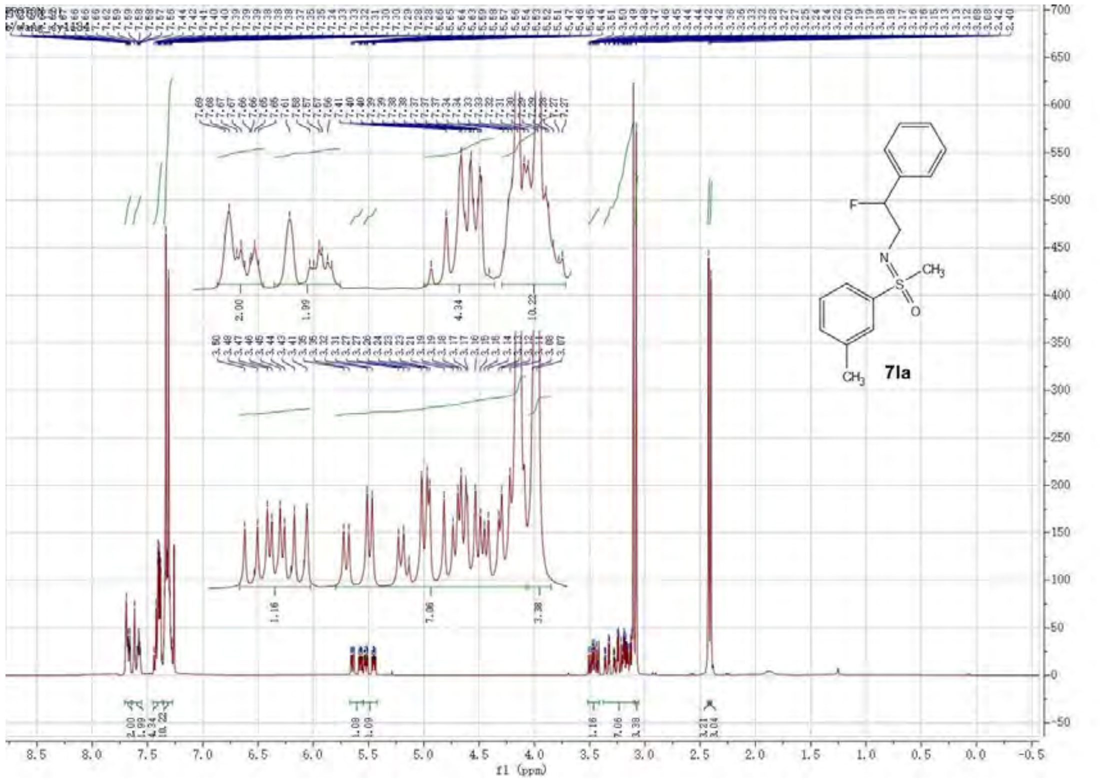

^{1}H NMR spectrum of compound **7la** (400 MHz, $CDCl_3$)

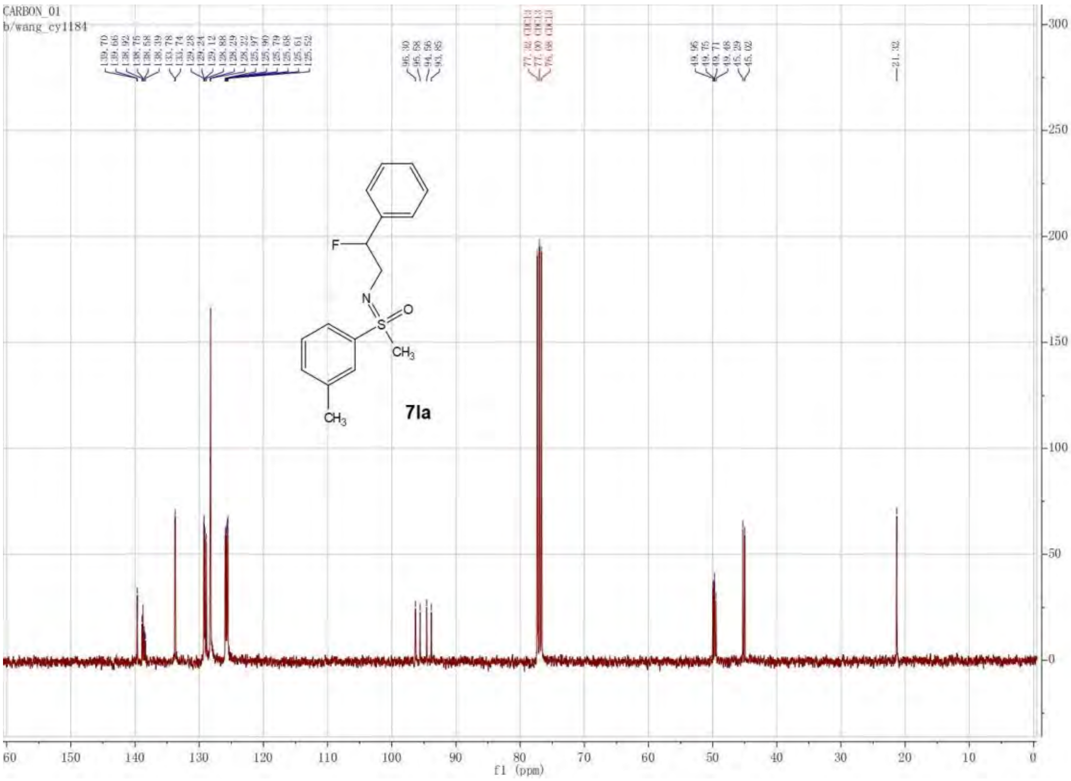

^{13}C NMR spectrum of compound **7la** (101 MHz, $CDCl_3$)

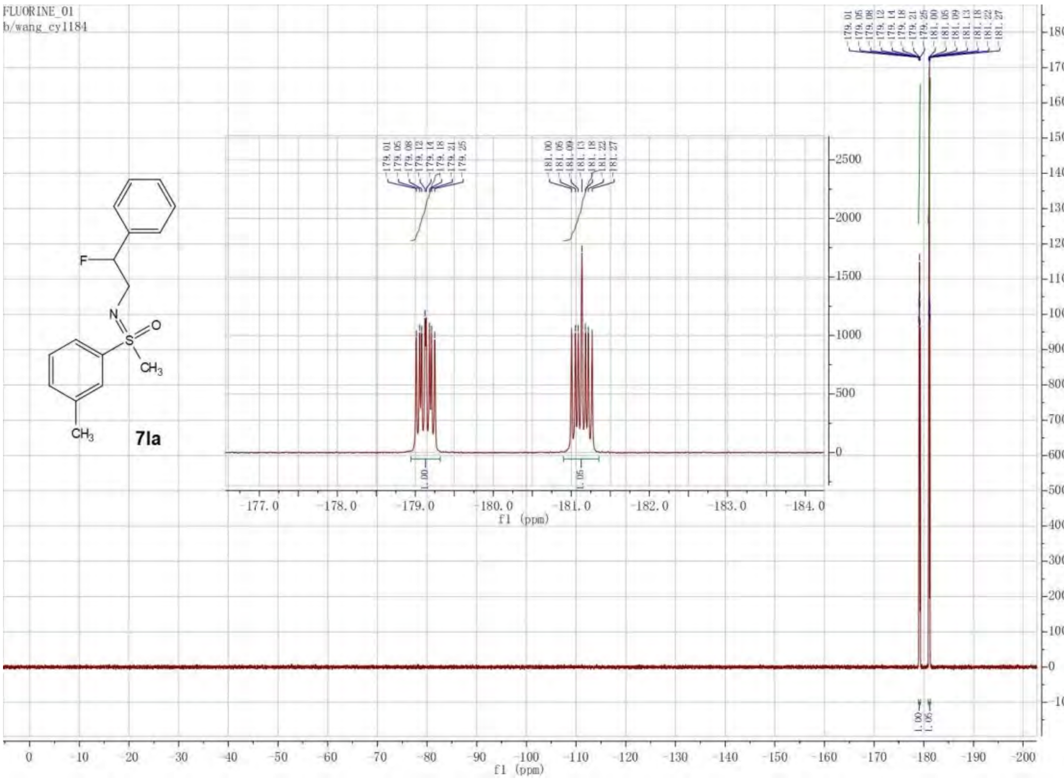

^{19}F NMR spectrum of compound **7la** (376 MHz, $CDCl_3$)

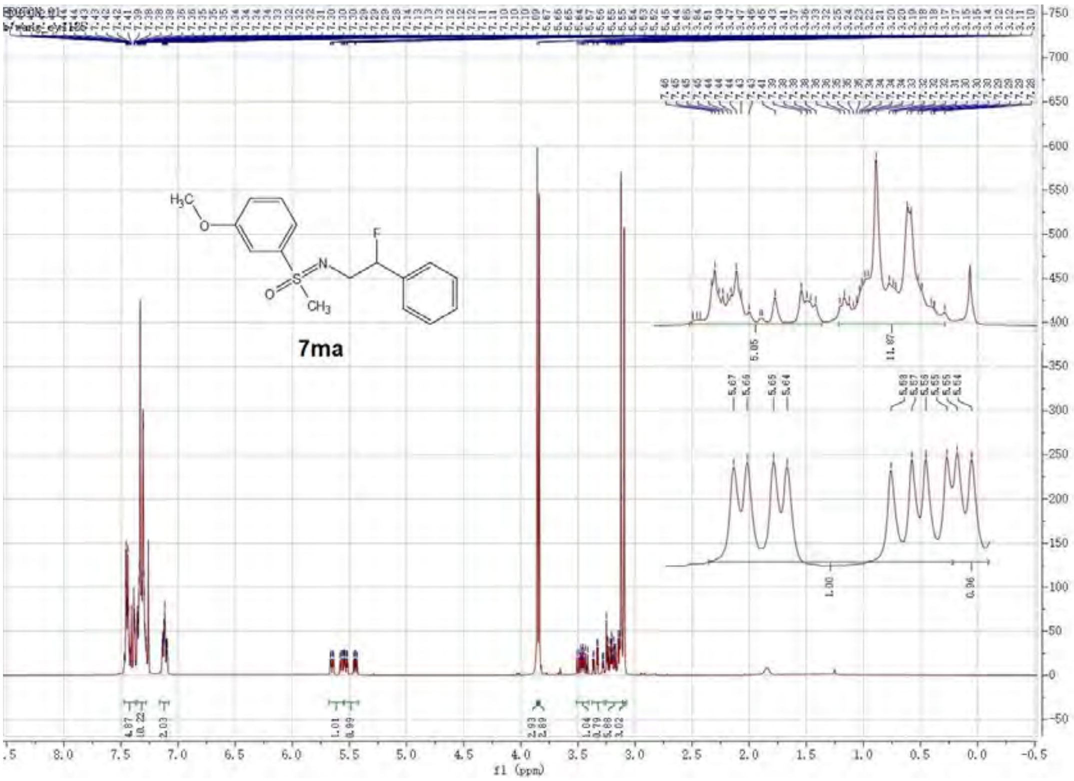

^{1}H NMR spectrum of compound **7ma** (600 MHz, $CDCl_3$)

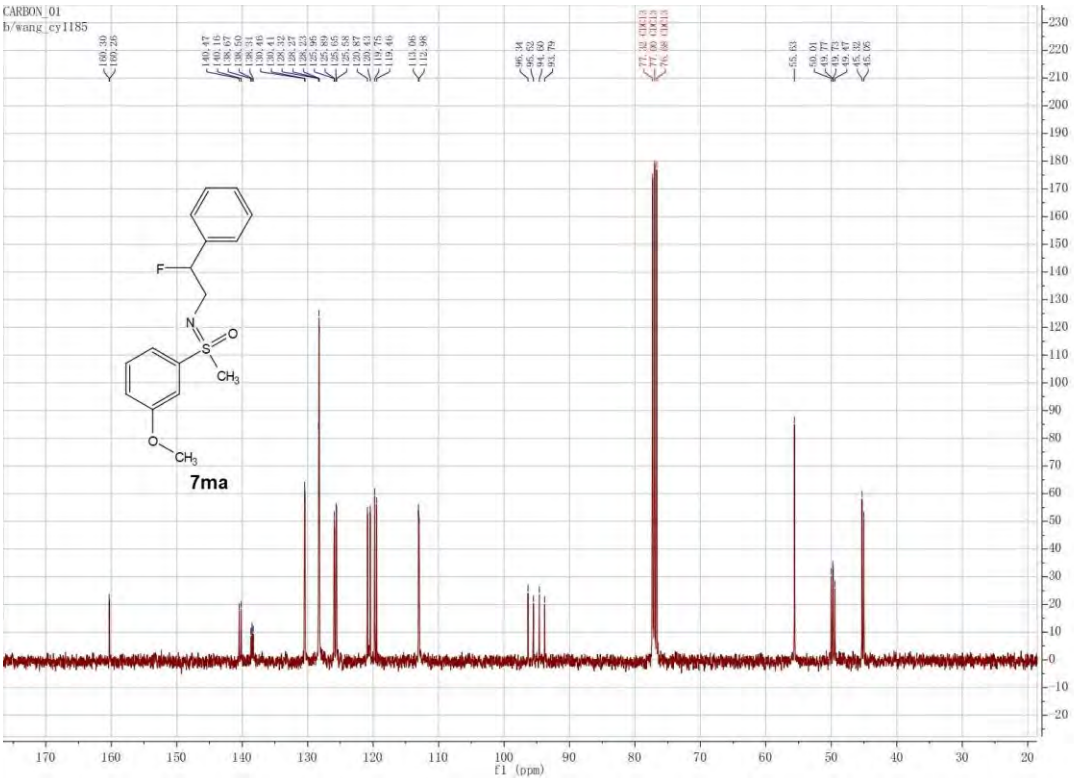

^{13}C NMR spectrum of compound **7ma** (151 MHz, $CDCl_3$)

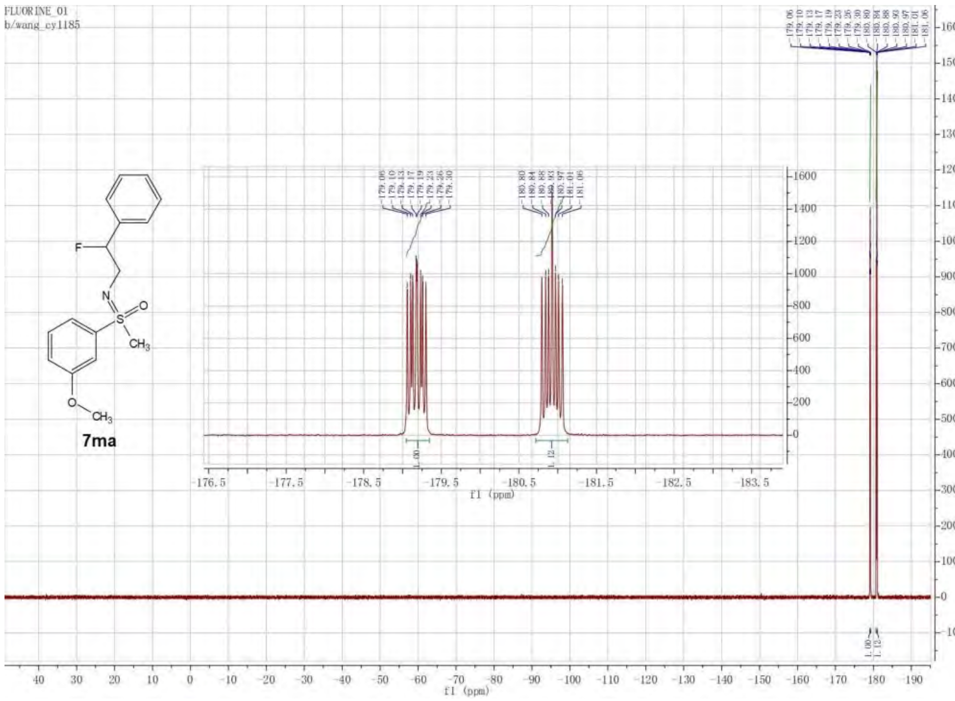

^{19}F NMR spectrum of compound **7ma** (564 MHz, $CDCl_3$)

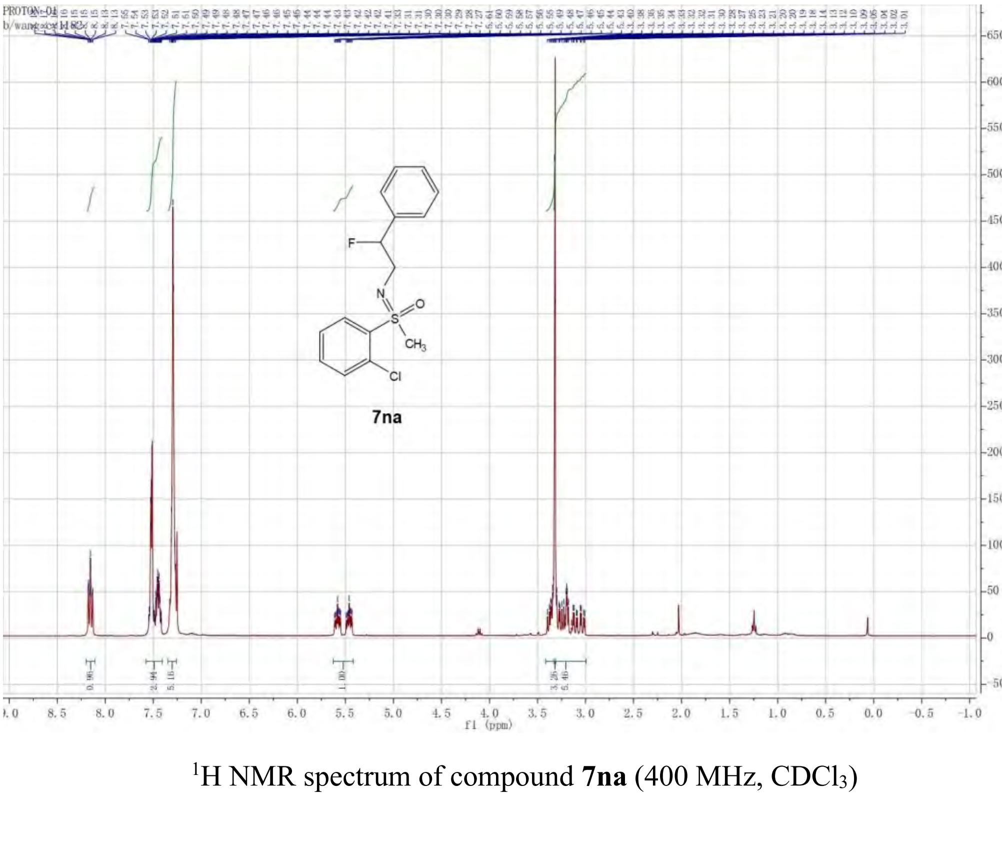

^{1}H NMR spectrum of compound **7na** (400 MHz, $CDCl_3$)

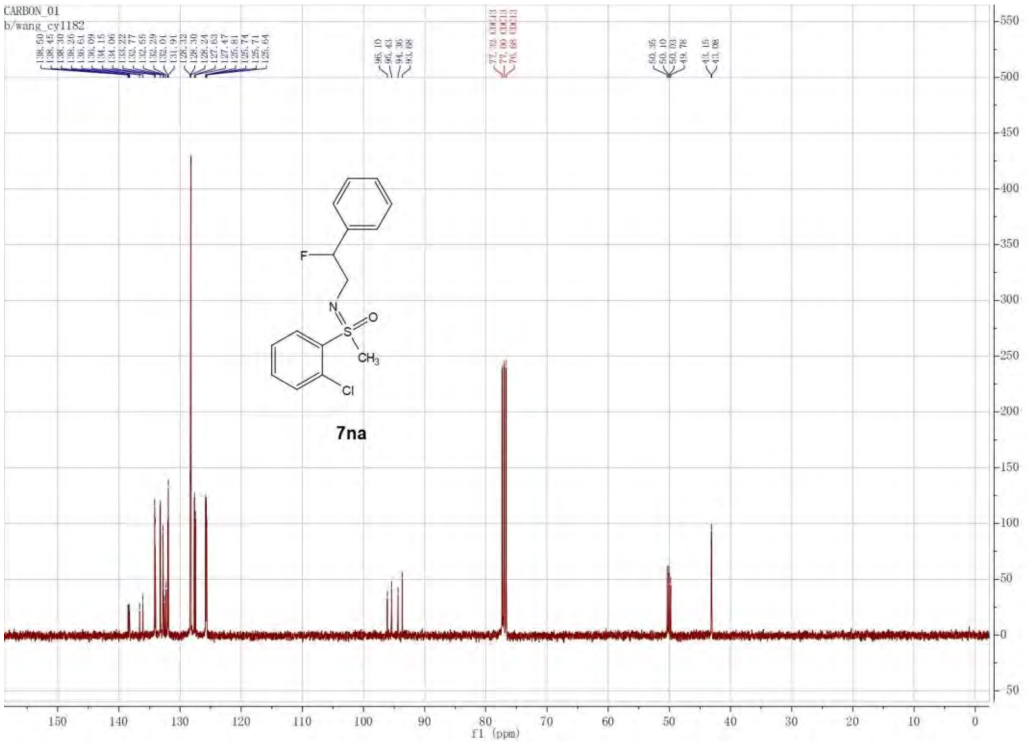

^{13}C NMR spectrum of compound **7na** (101 MHz, $CDCl_3$)

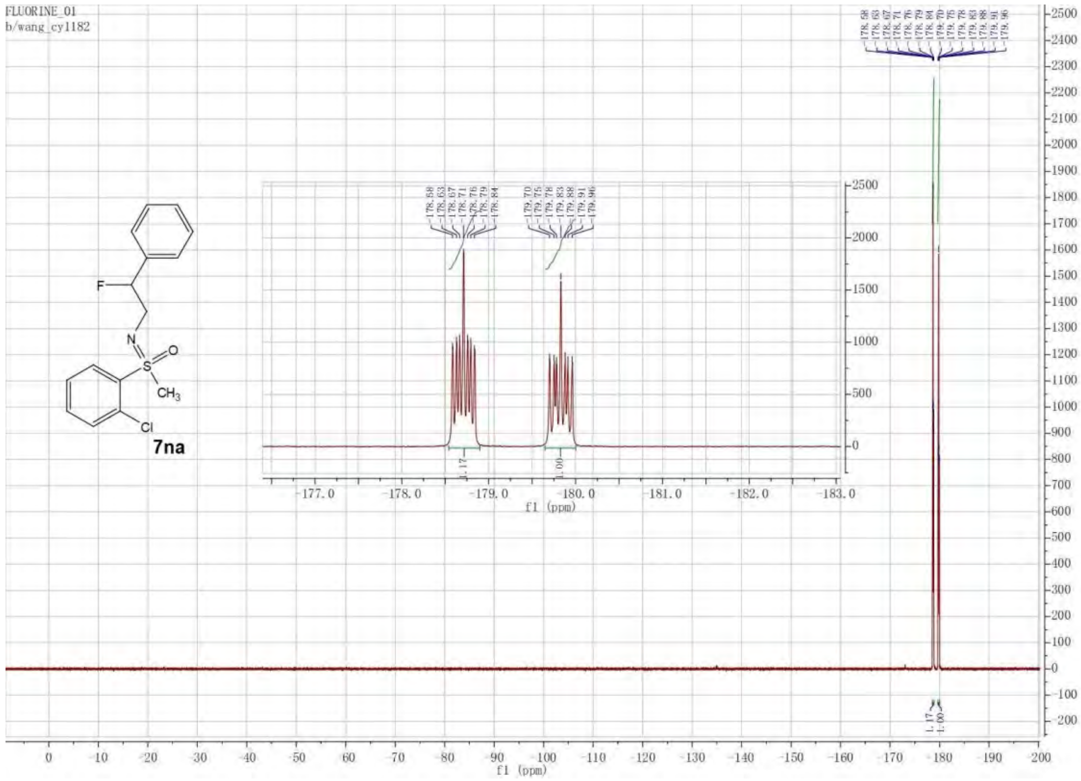

^{19}F NMR spectrum of compound **7na** (376 MHz, $CDCl_3$)

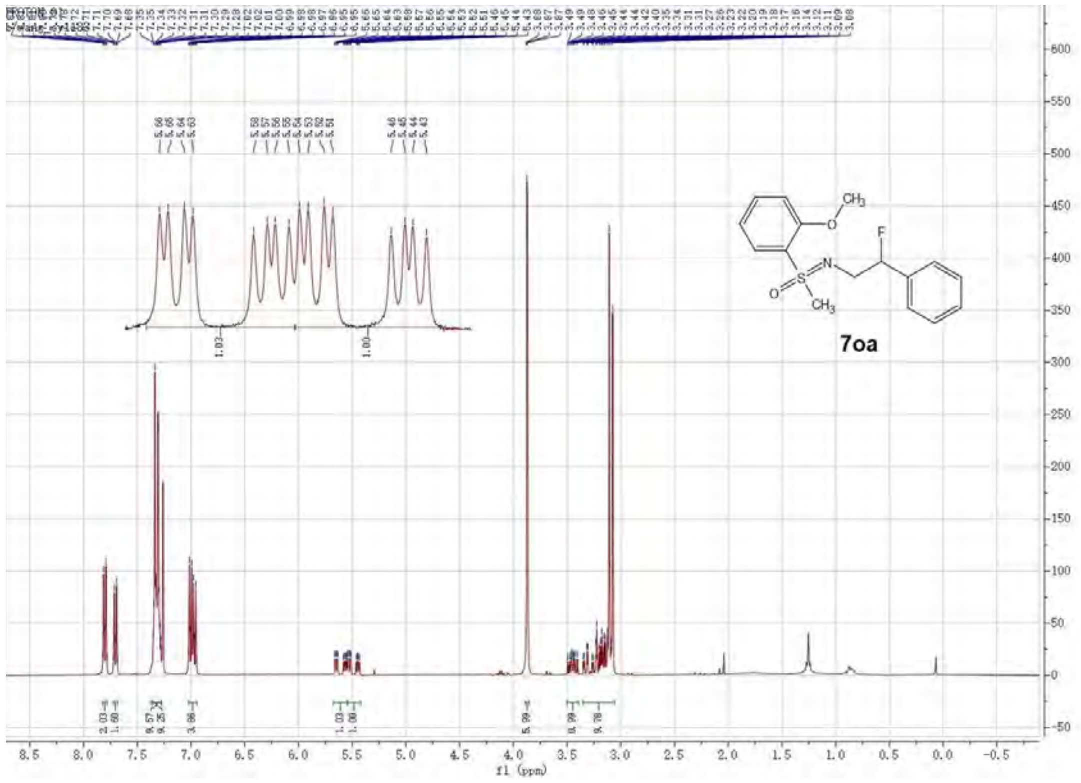

^{1}H NMR spectrum of compound **7oa** (400 MHz, $CDCl_3$)

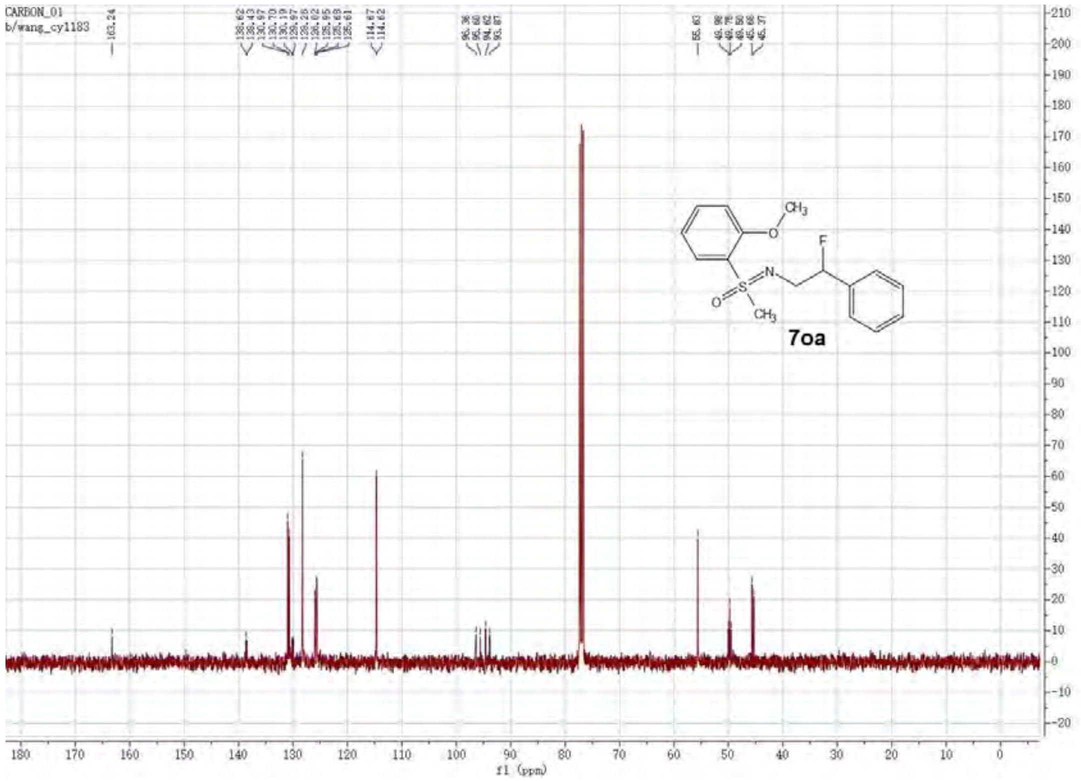

^{13}C NMR spectrum of compound **7oa** (101 MHz, $CDCl_3$)

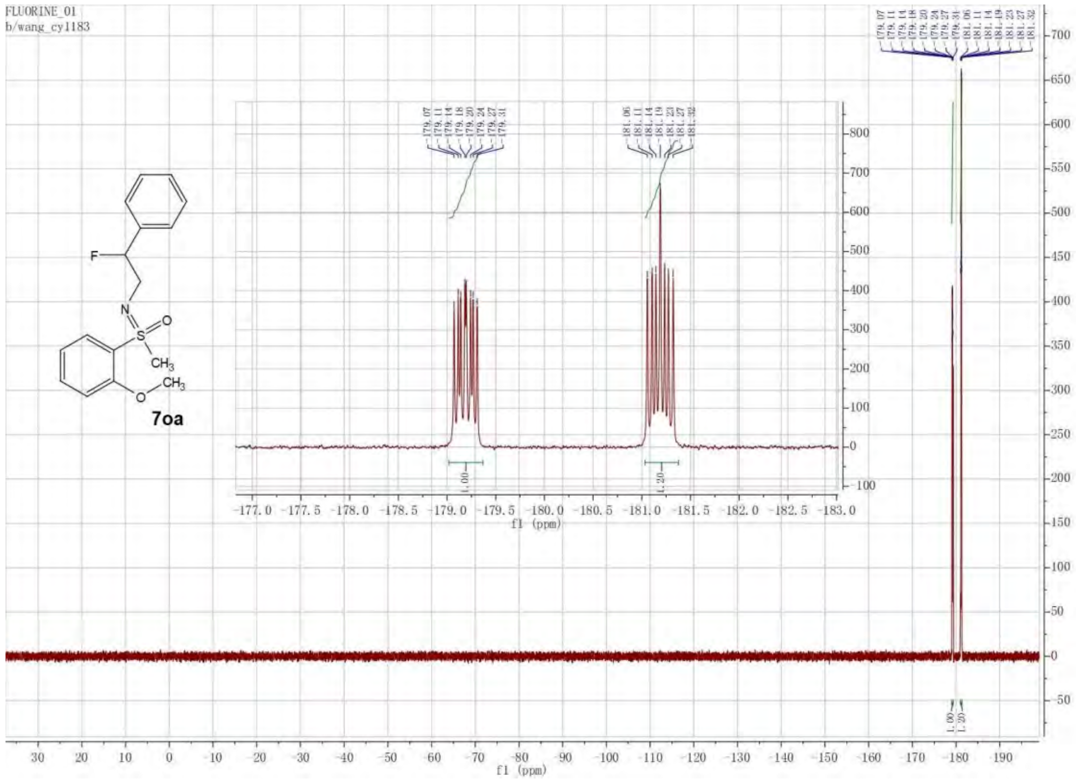

^{19}F NMR spectrum of compound **7oa** (376 MHz, $CDCl_3$)

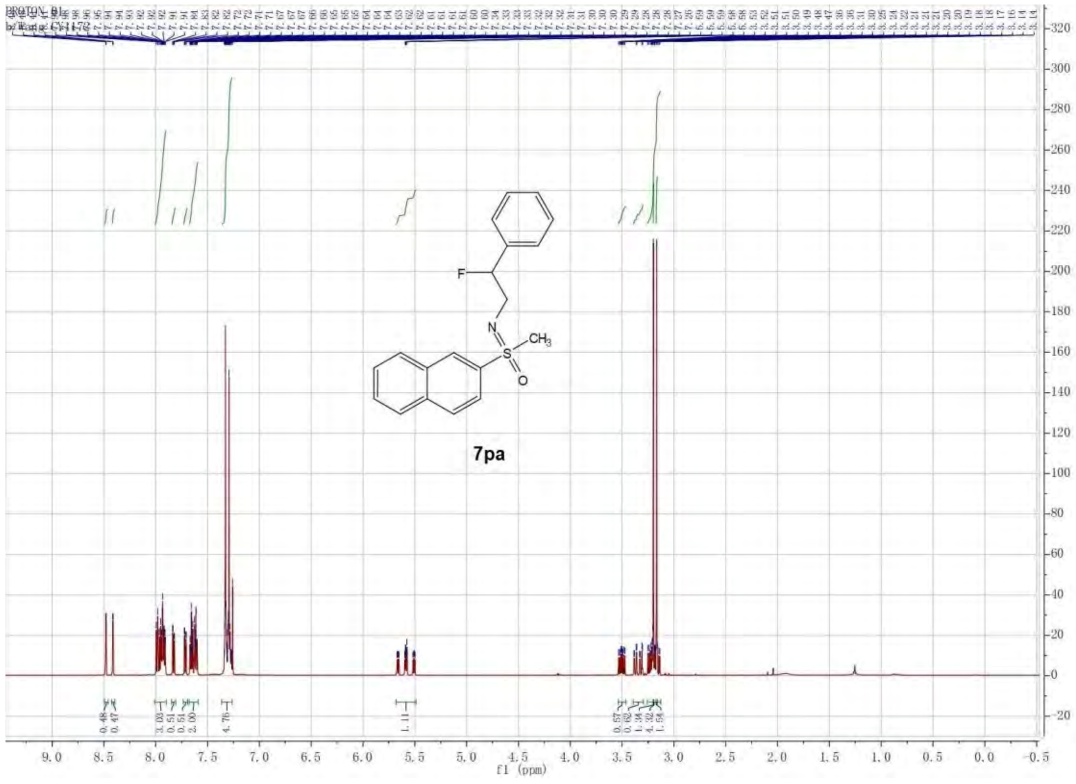

^{1}H NMR spectrum of compound **7pa** (600 MHz, $CDCl_3$)

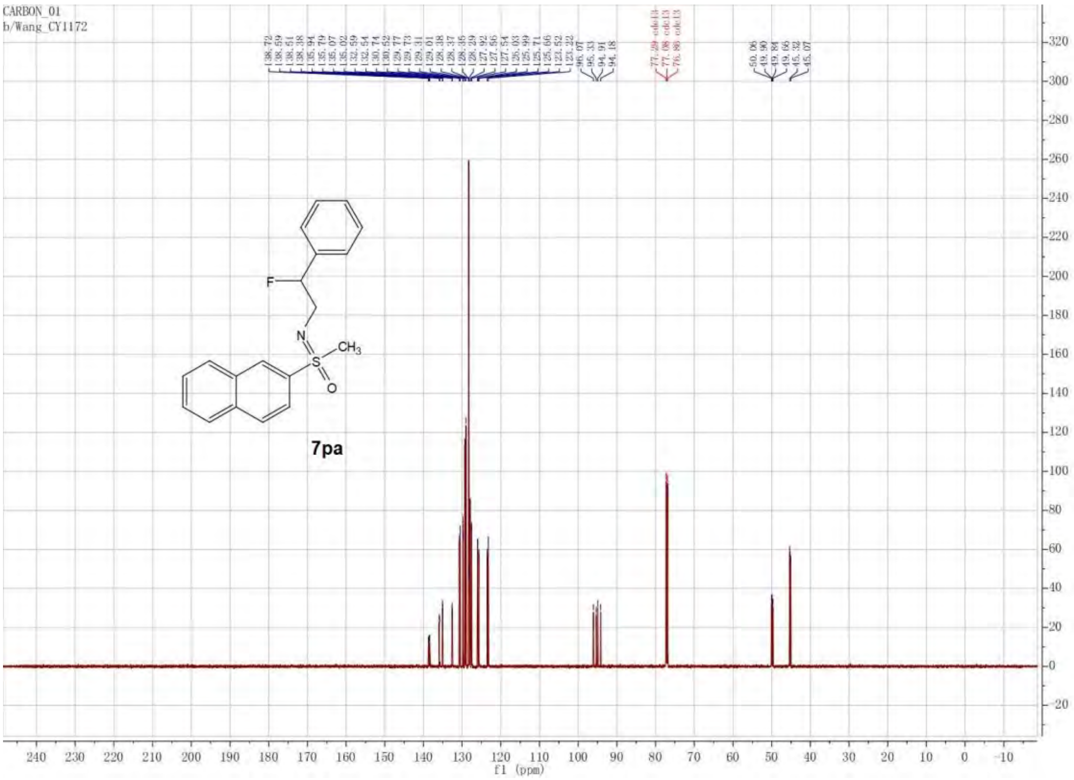

^{13}C NMR spectrum of compound **7pa** (151 MHz, $CDCl_3$)

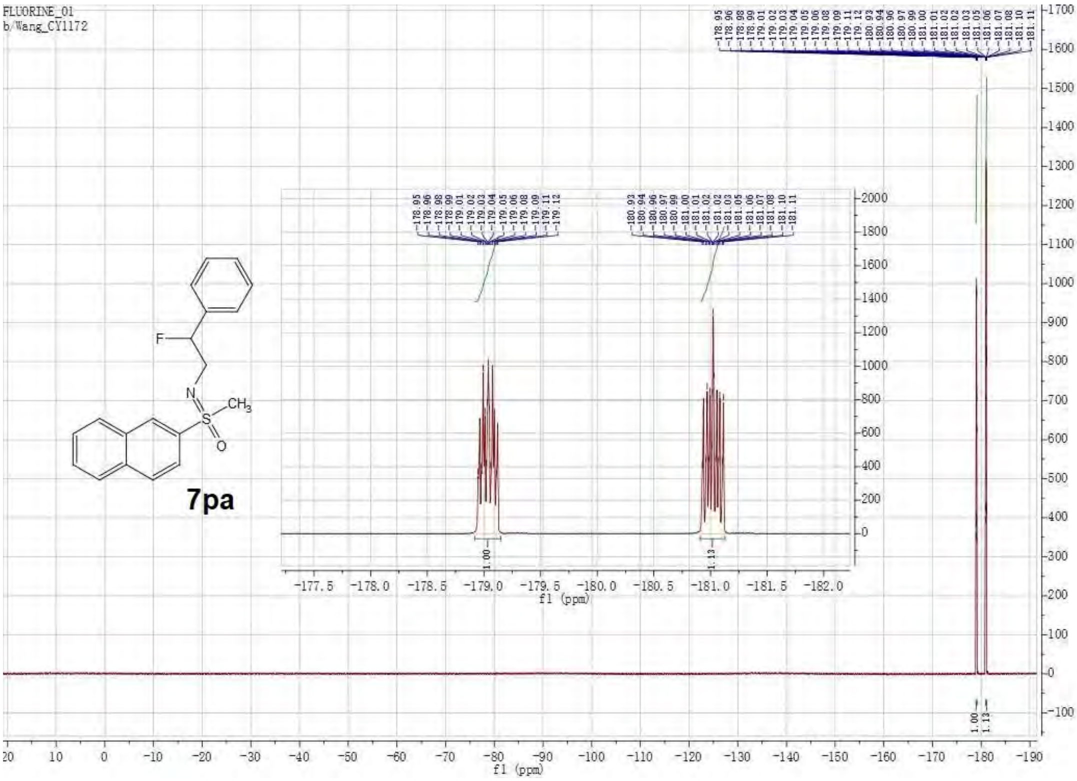

^{19}F NMR spectrum of compound **7pa** (564 MHz, $CDCl_3$)

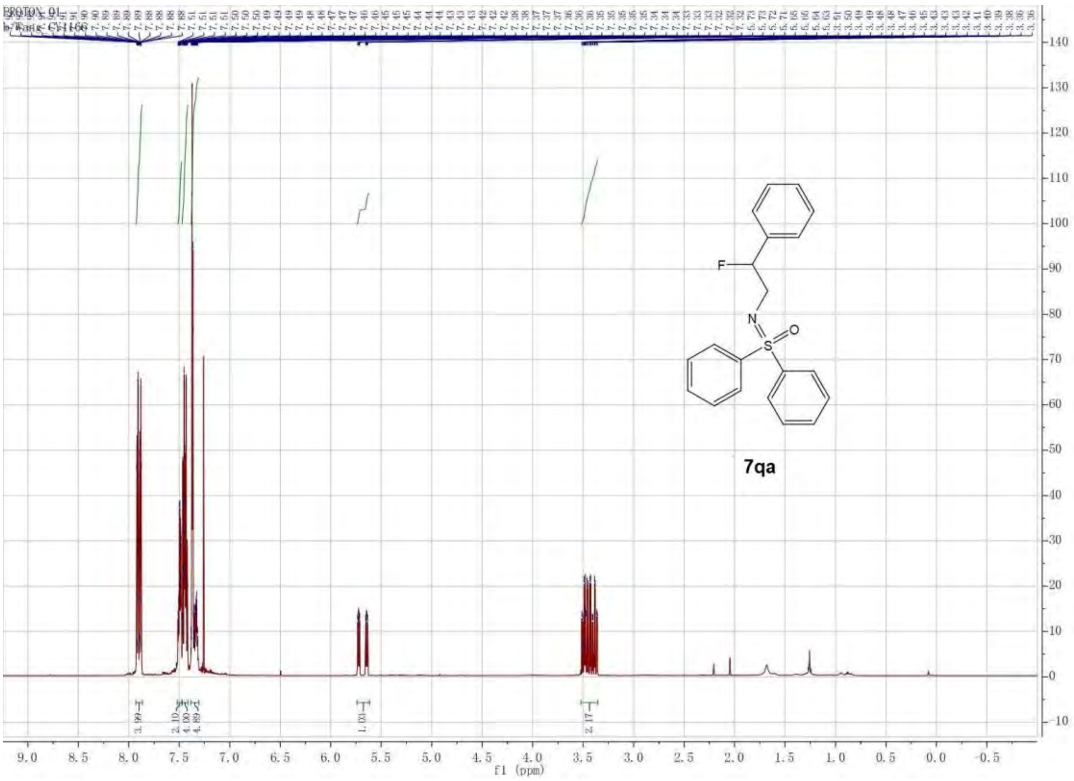

^{1}H NMR spectrum of compound **7qa** (600 MHz, $CDCl_3$)

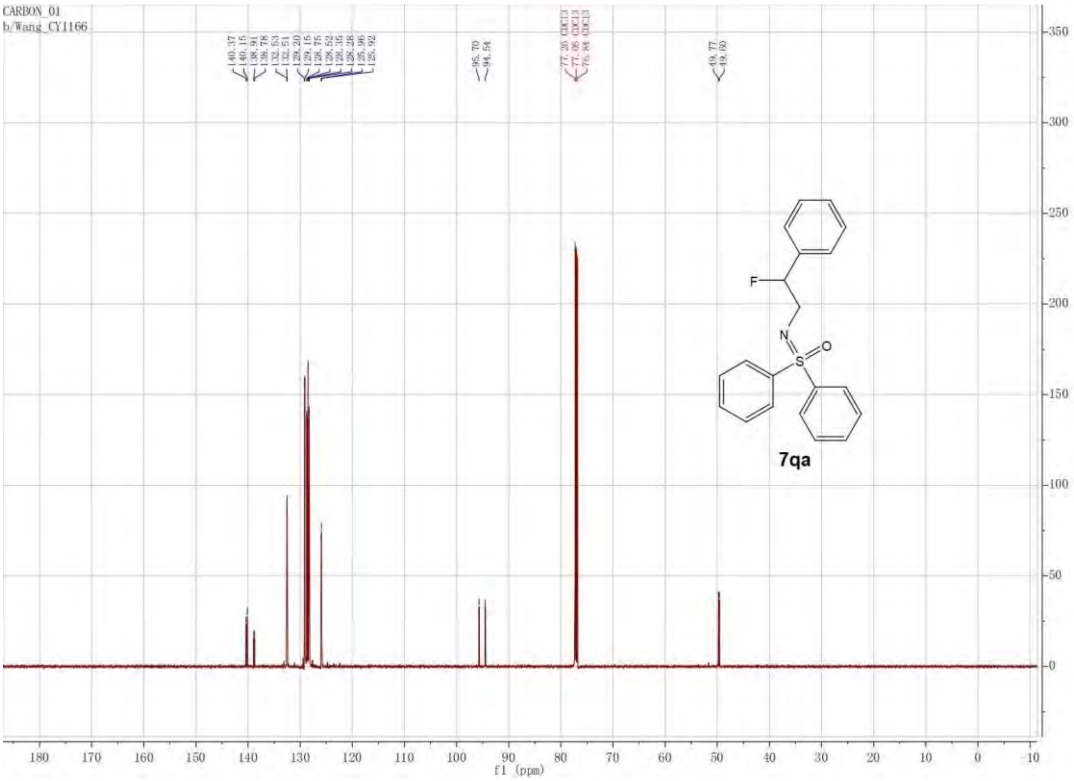

^{13}C NMR spectrum of compound **7qa** (151 MHz, $CDCl_3$)

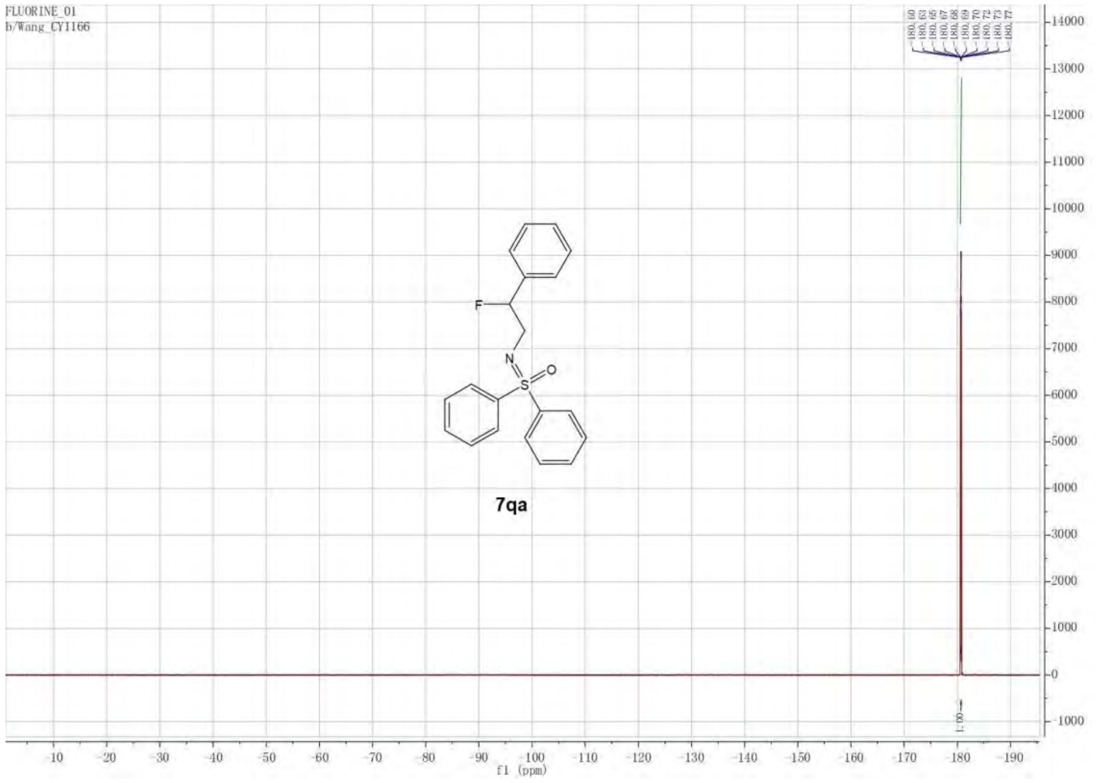

^{19}F NMR spectrum of compound **7qa** (564 MHz, $CDCl_3$)

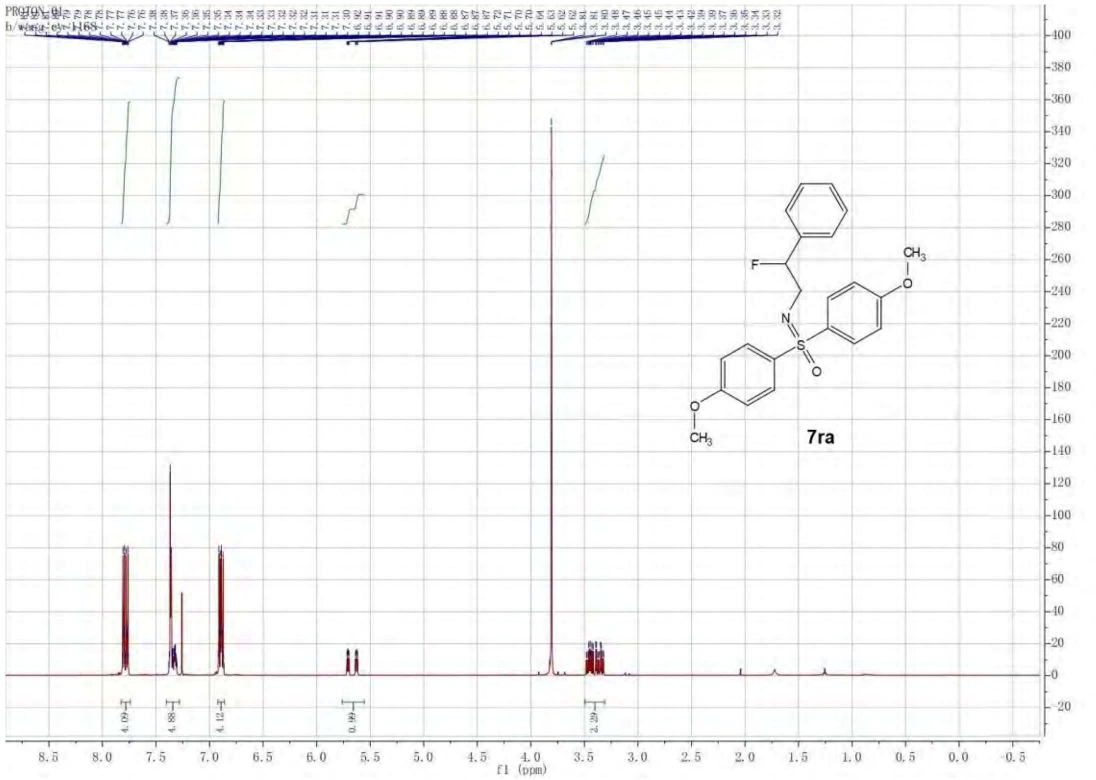

^{1}H NMR spectrum of compound **7ra** (600 MHz, $CDCl_3$)

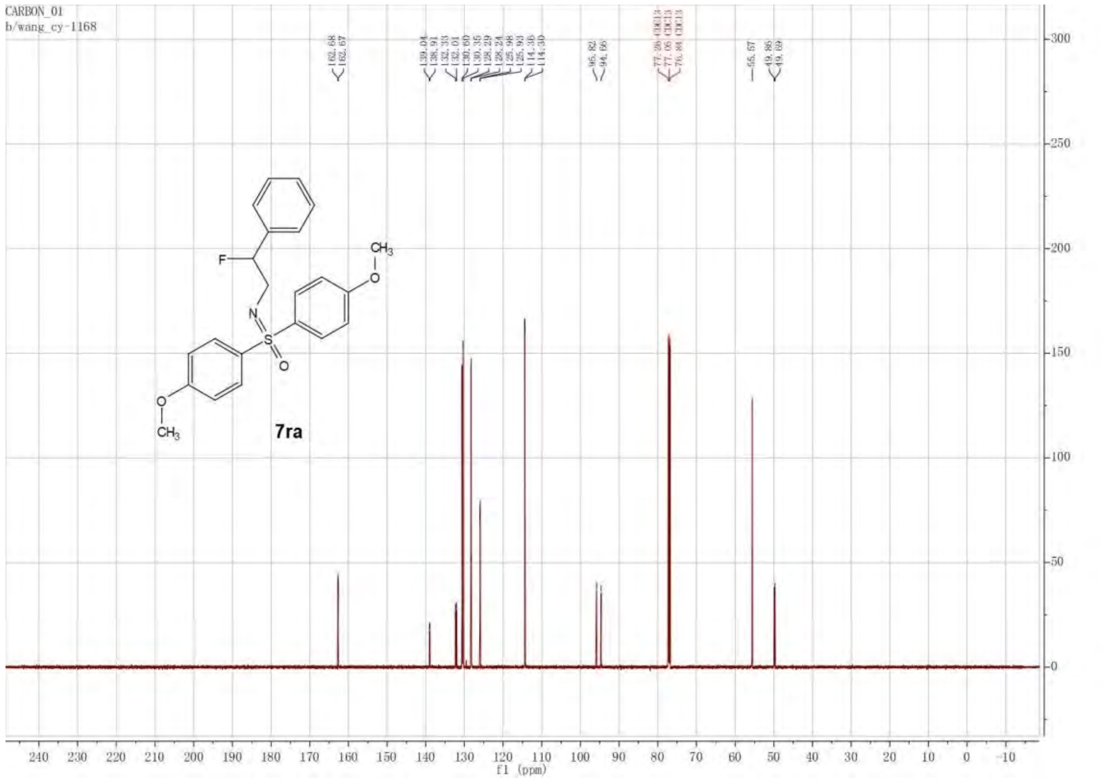

^{13}C NMR spectrum of compound **7ra** (151 MHz, $CDCl_3$)

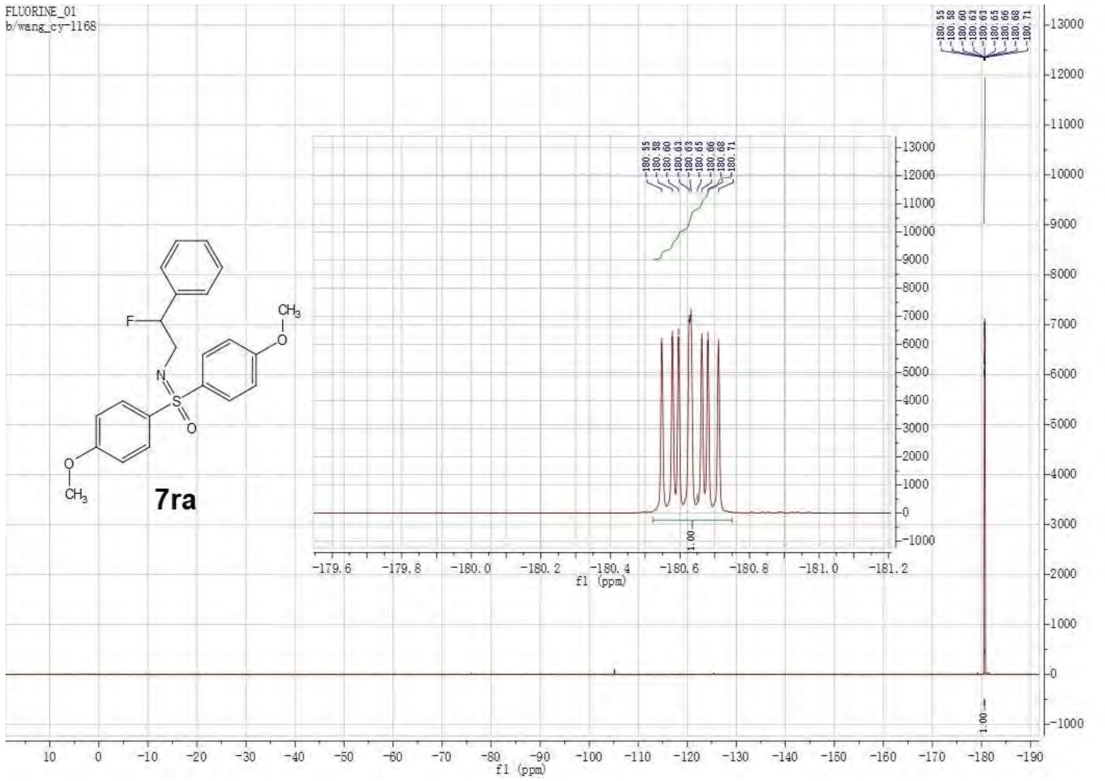

^{19}F NMR spectrum of compound **7ra** (564 MHz, $CDCl_3$)

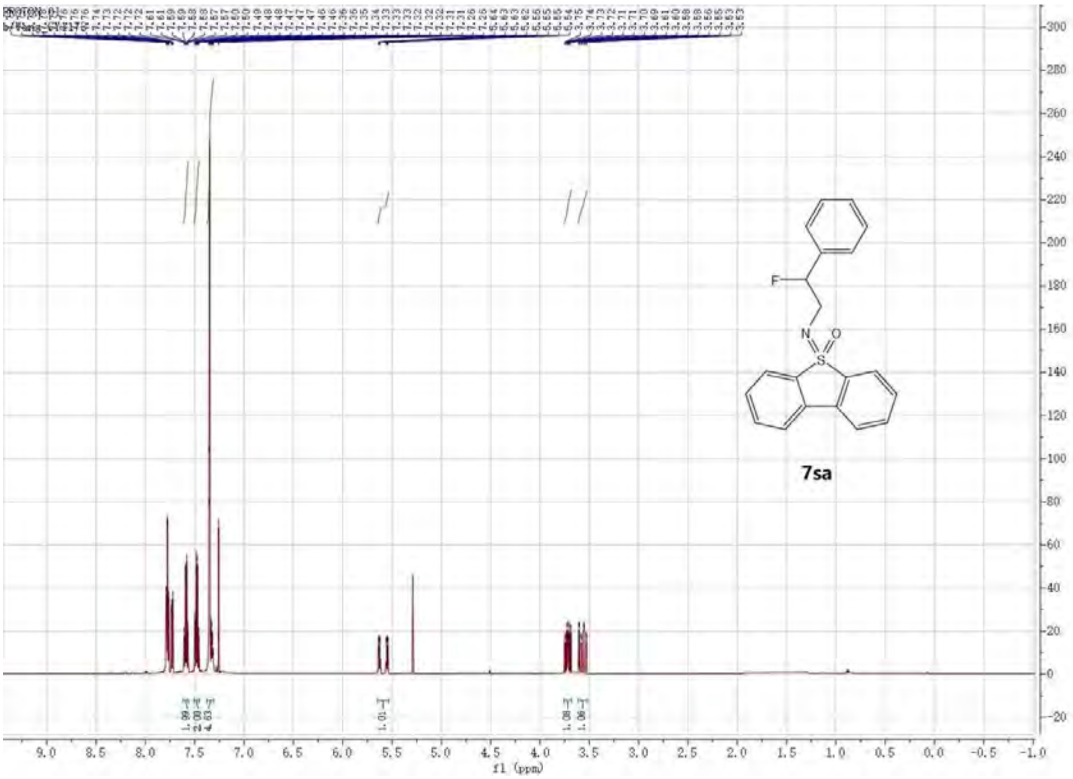

^{1}H NMR spectrum of compound **7sa** (600 MHz, $CDCl_3$)

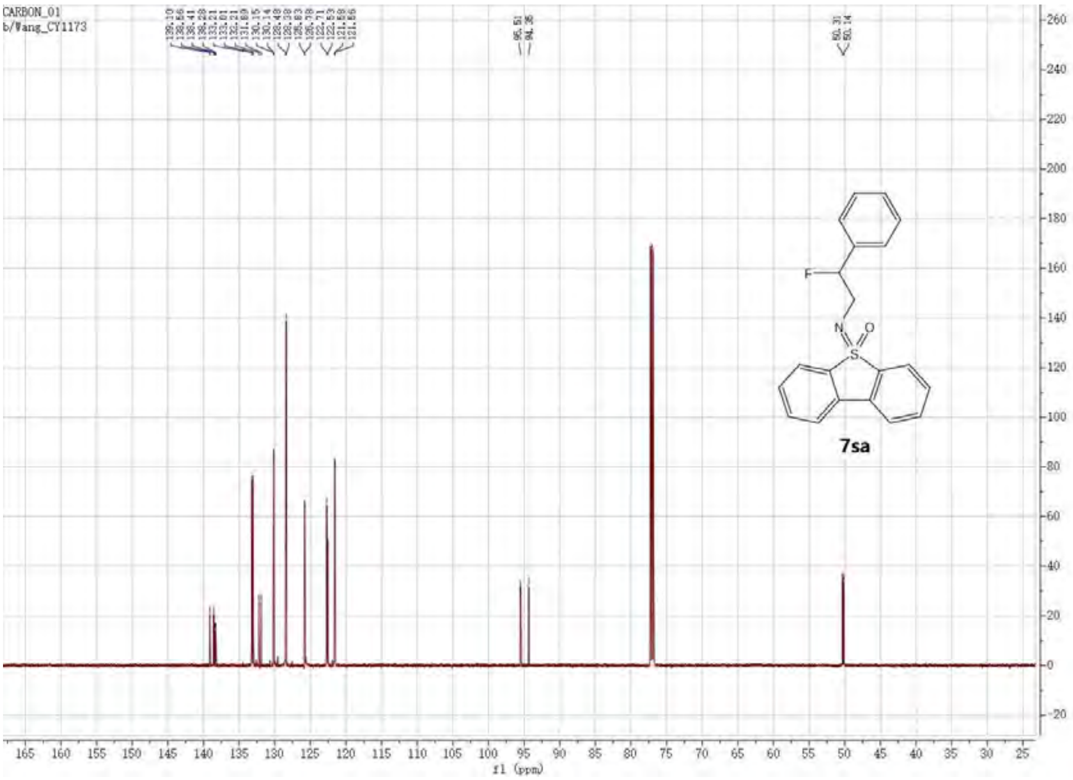

^{13}C NMR spectrum of compound **7sa** (151 MHz, $CDCl_3$)

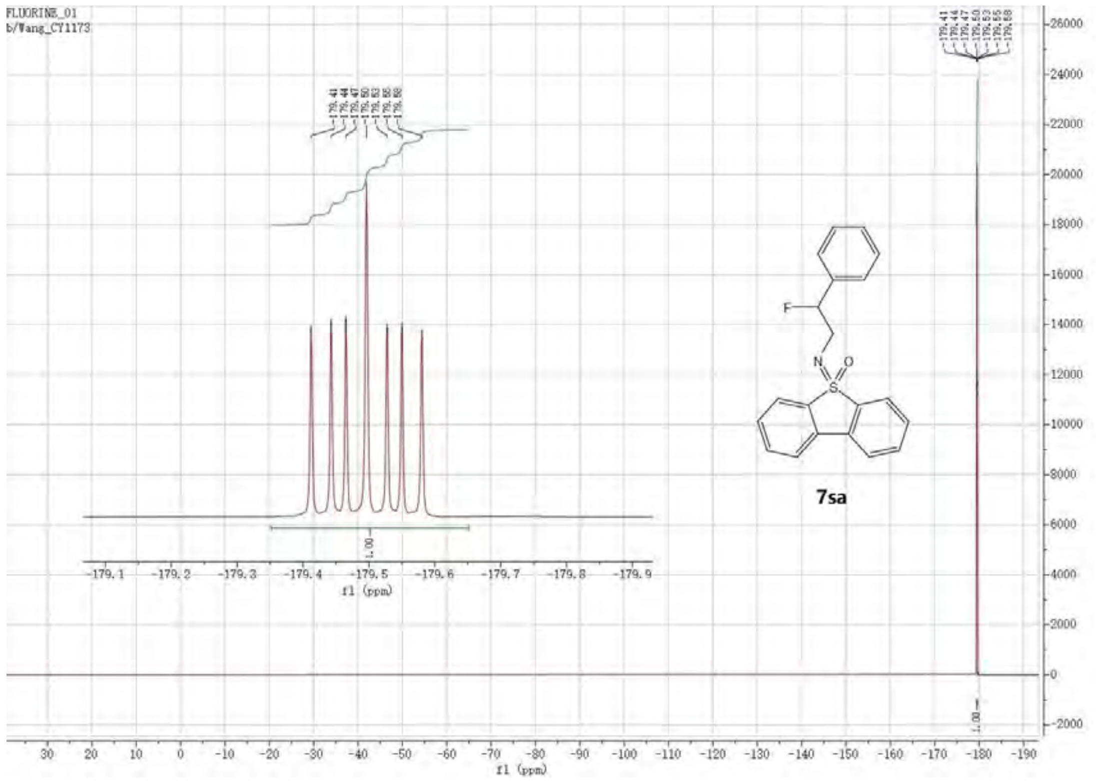

^{19}F NMR spectrum of compound **7sa** (564 MHz, $CDCl_3$)

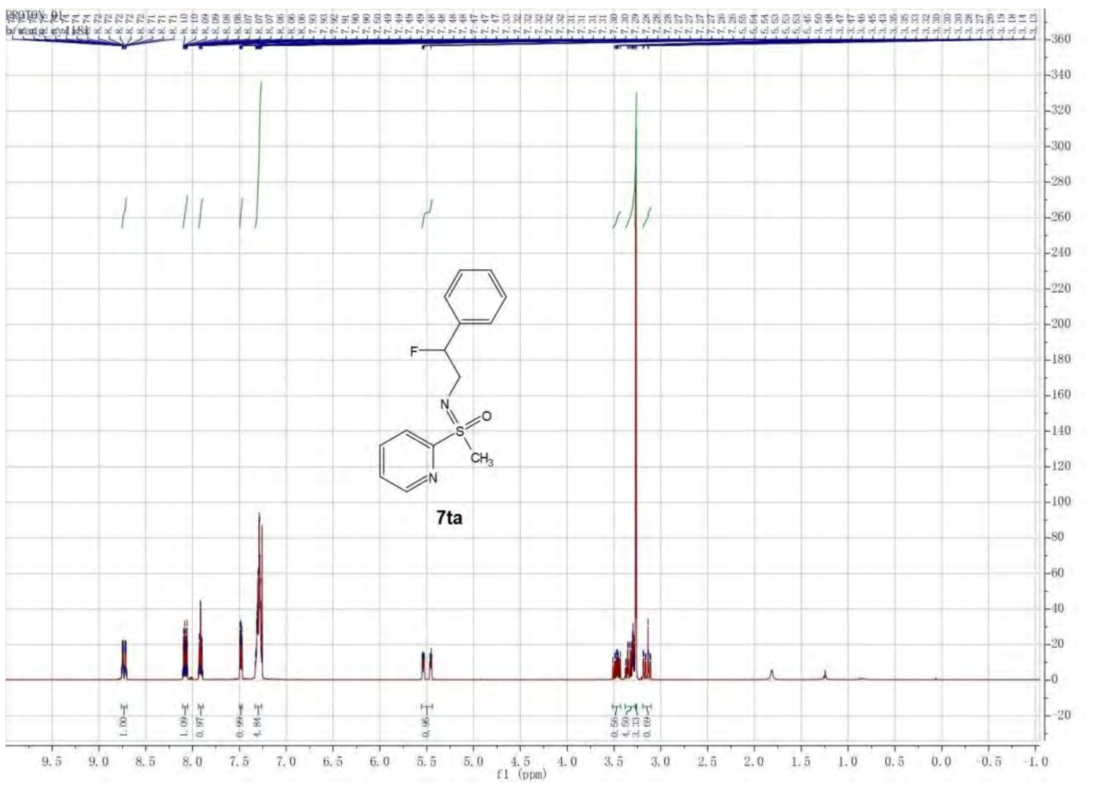

^{1}H NMR spectrum of compound **7ta** (600 MHz, $CDCl_3$)

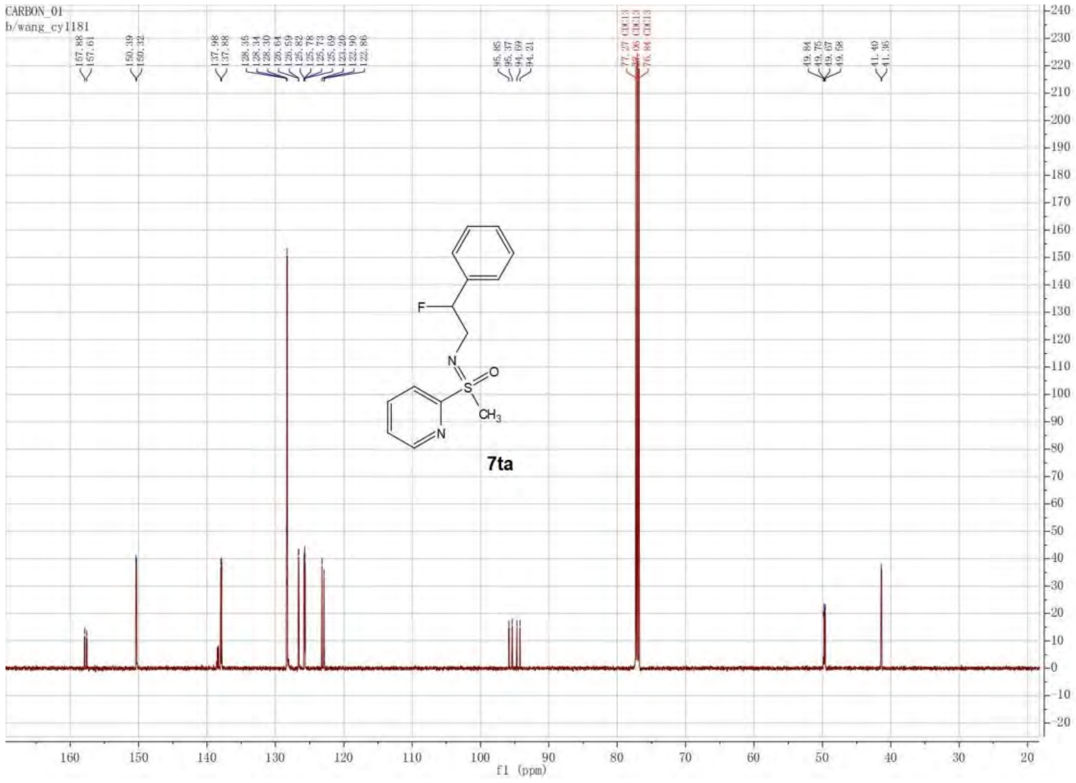

^{13}C NMR spectrum of compound **7ta** (151 MHz, $CDCl_3$)

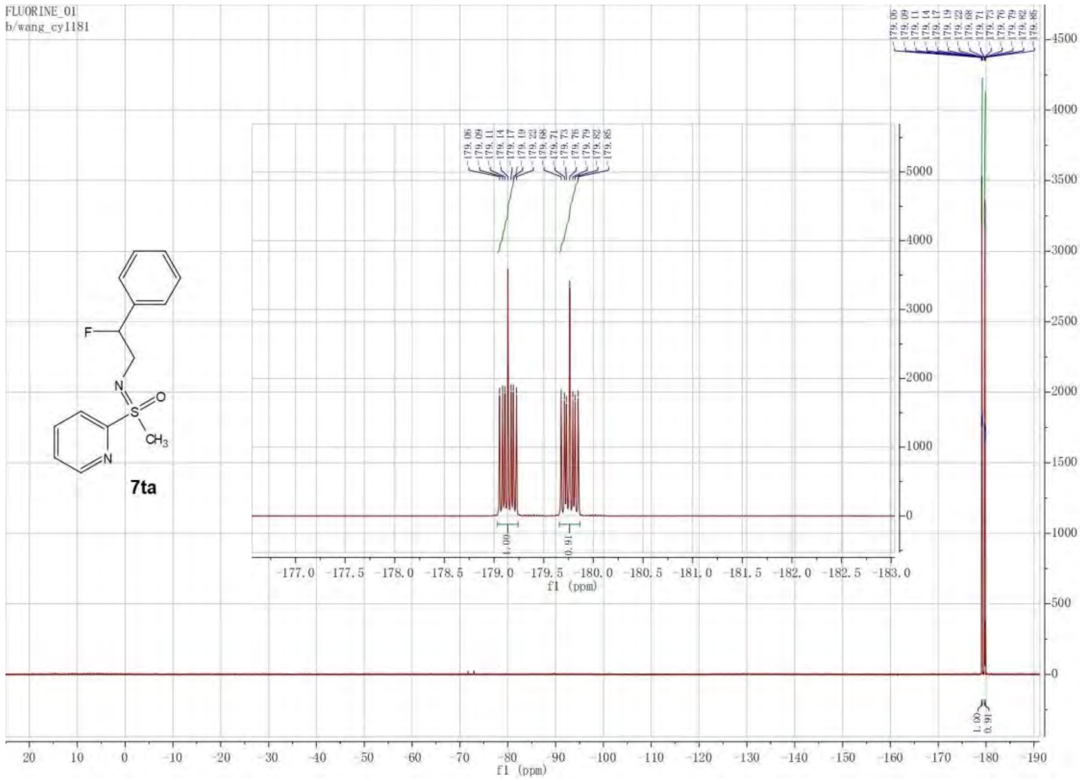

^{19}F NMR spectrum of compound **7ta** (564 MHz, $CDCl_3$)

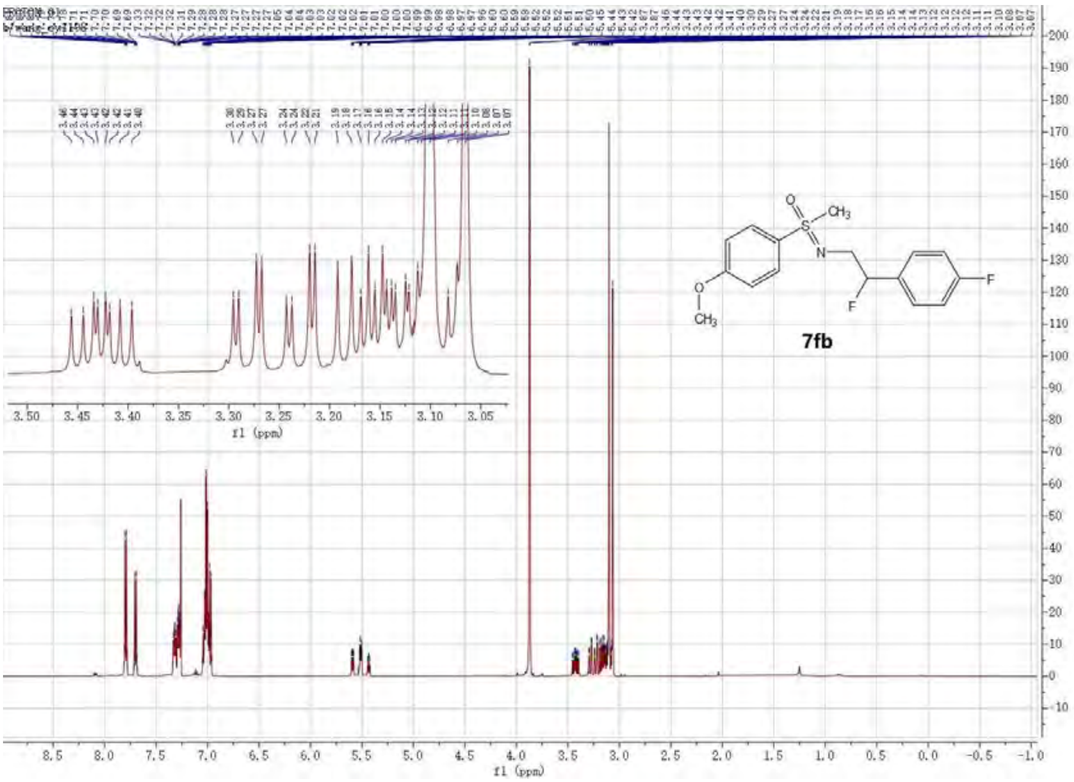

^{1}H NMR spectrum of compound **7fb** (600 MHz, $CDCl_3$)

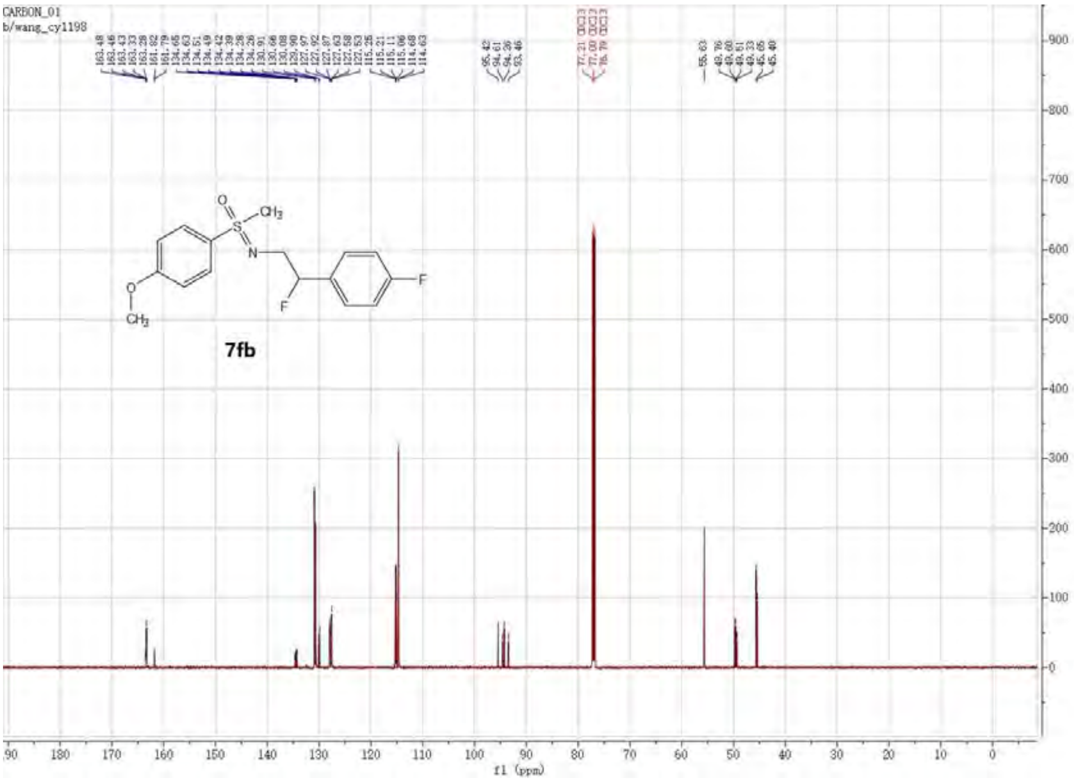

^{13}C NMR spectrum of compound **7fb** (151 MHz, $CDCl_3$)

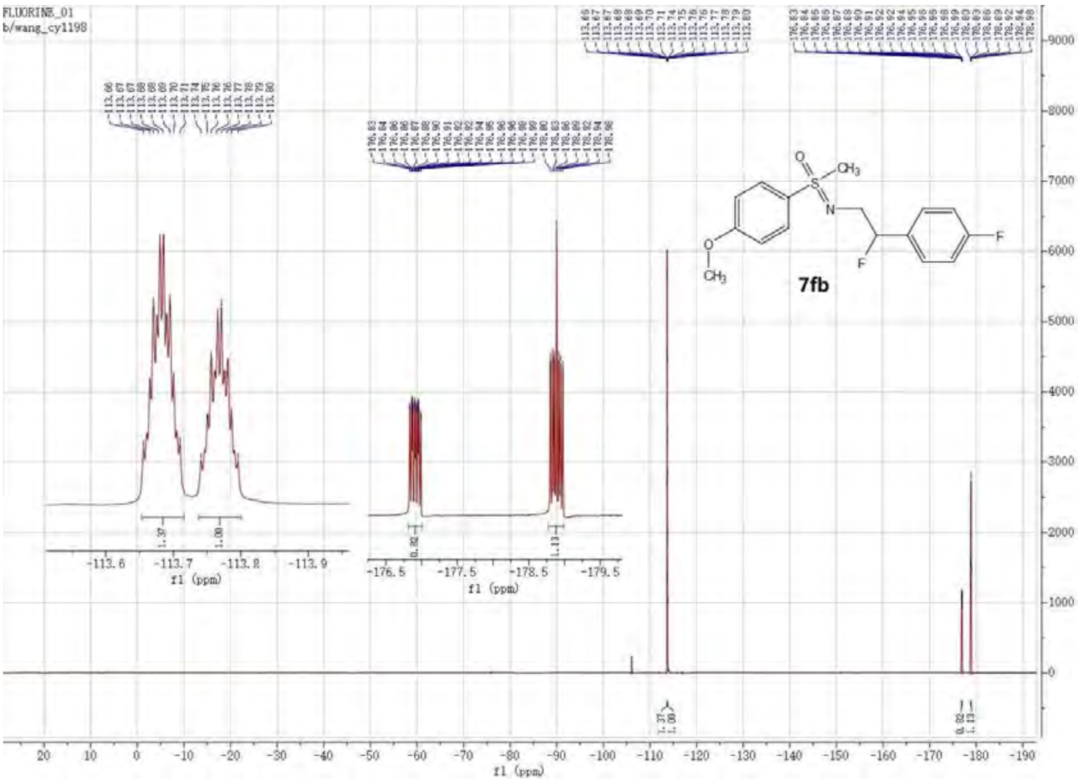

^{19}F NMR spectrum of compound **7fb** (564 MHz, CDCl$_3$)

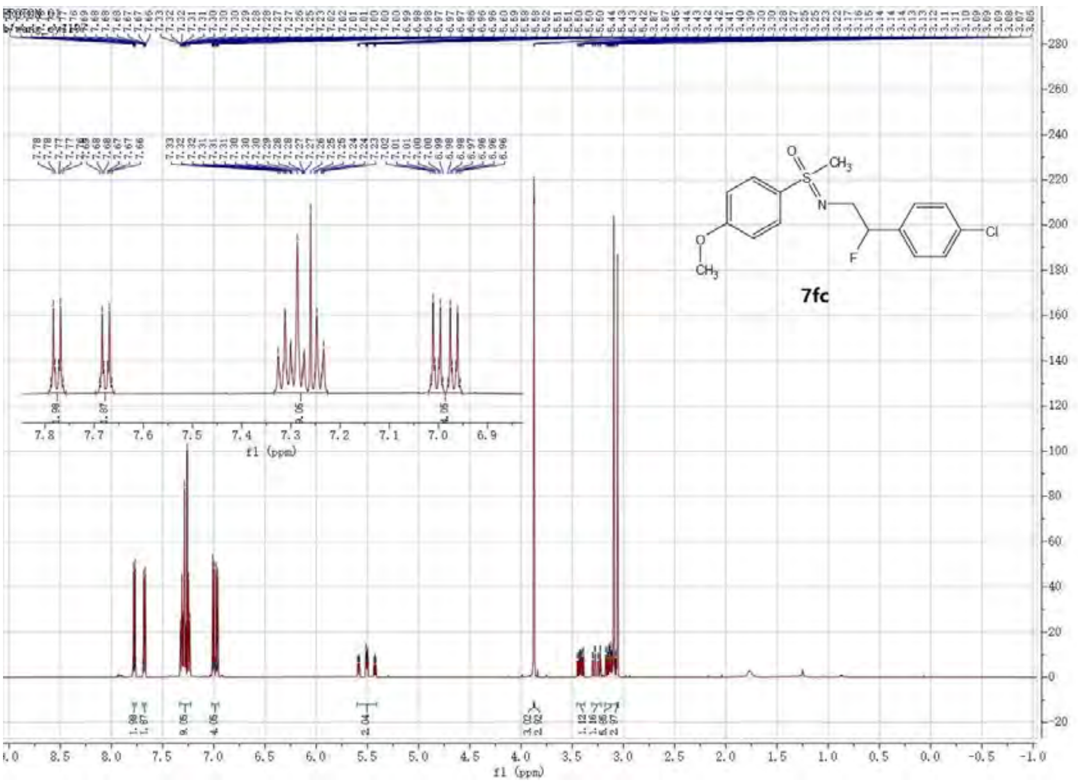

^{1}H NMR spectrum of compound **7fc** (600 MHz, CDCl$_3$)

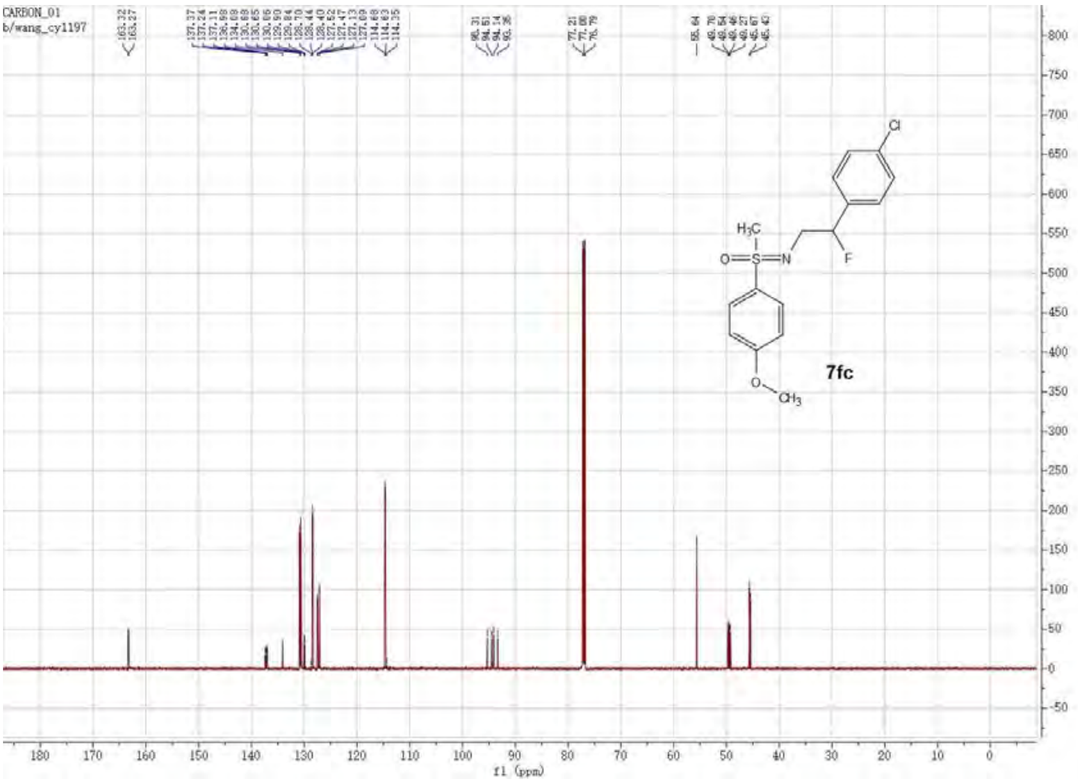

^{13}C NMR spectrum of compound **7fc** (151 MHz, $CDCl_3$)

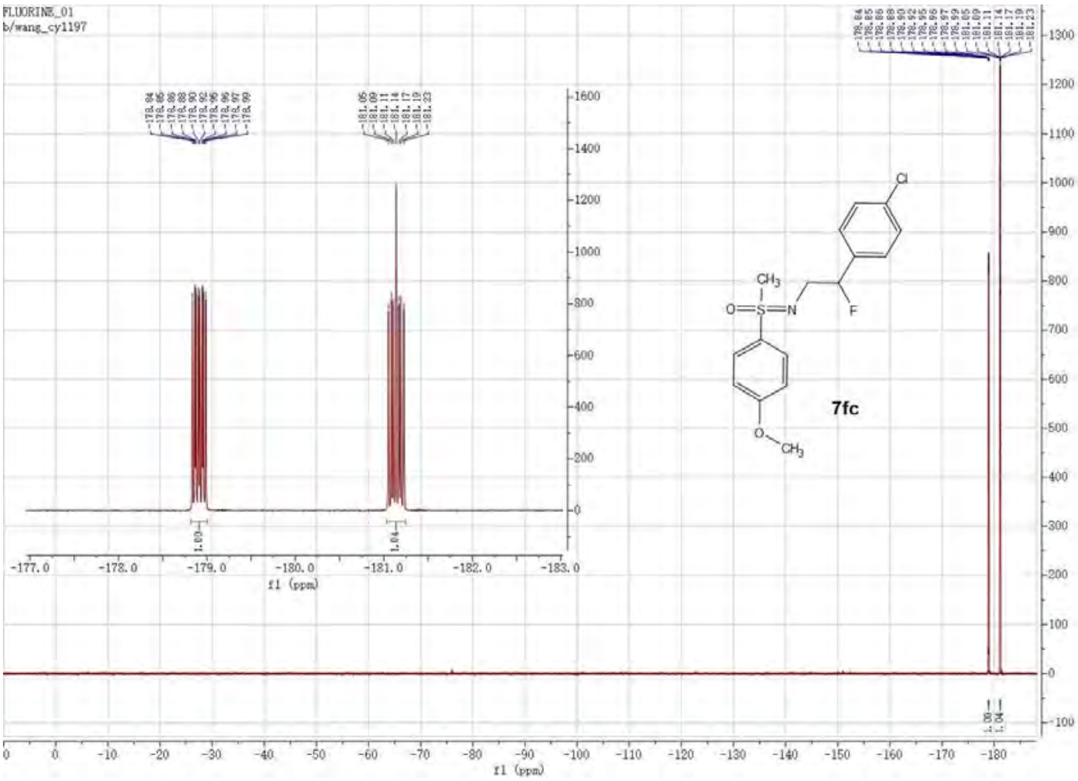

^{19}F NMR spectrum of compound **7fc** (564 MHz, $CDCl_3$)

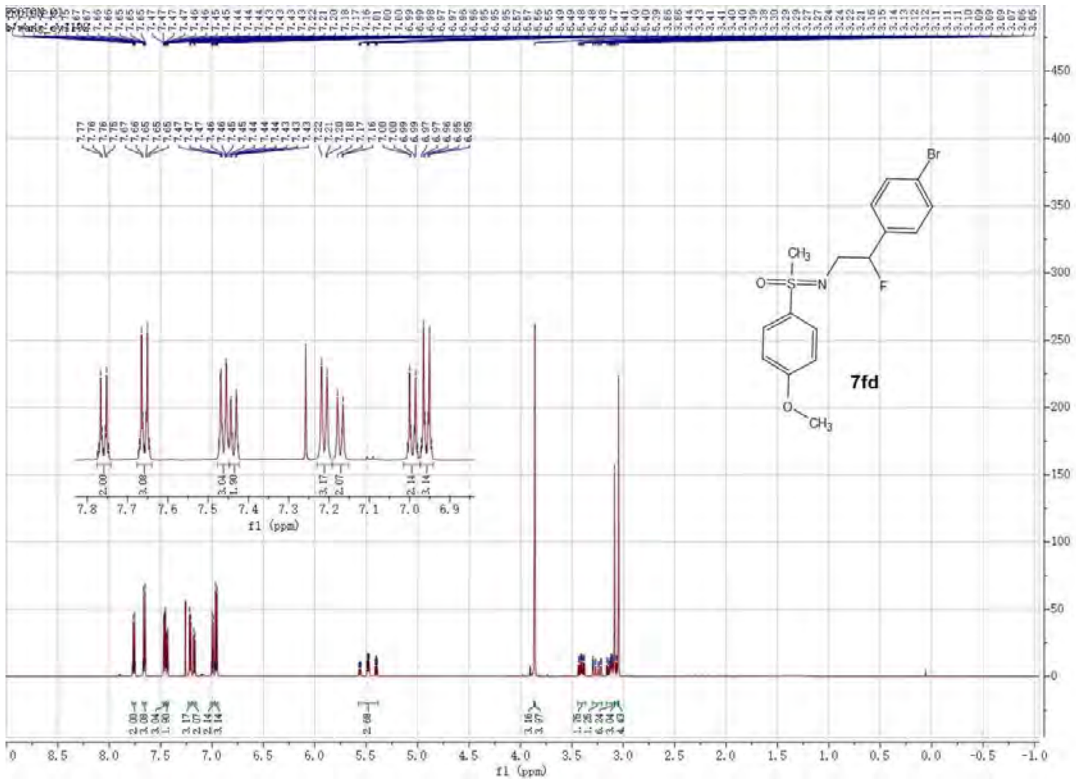

^{1}H NMR spectrum of compound **7fd** (600 MHz, $CDCl_3$)

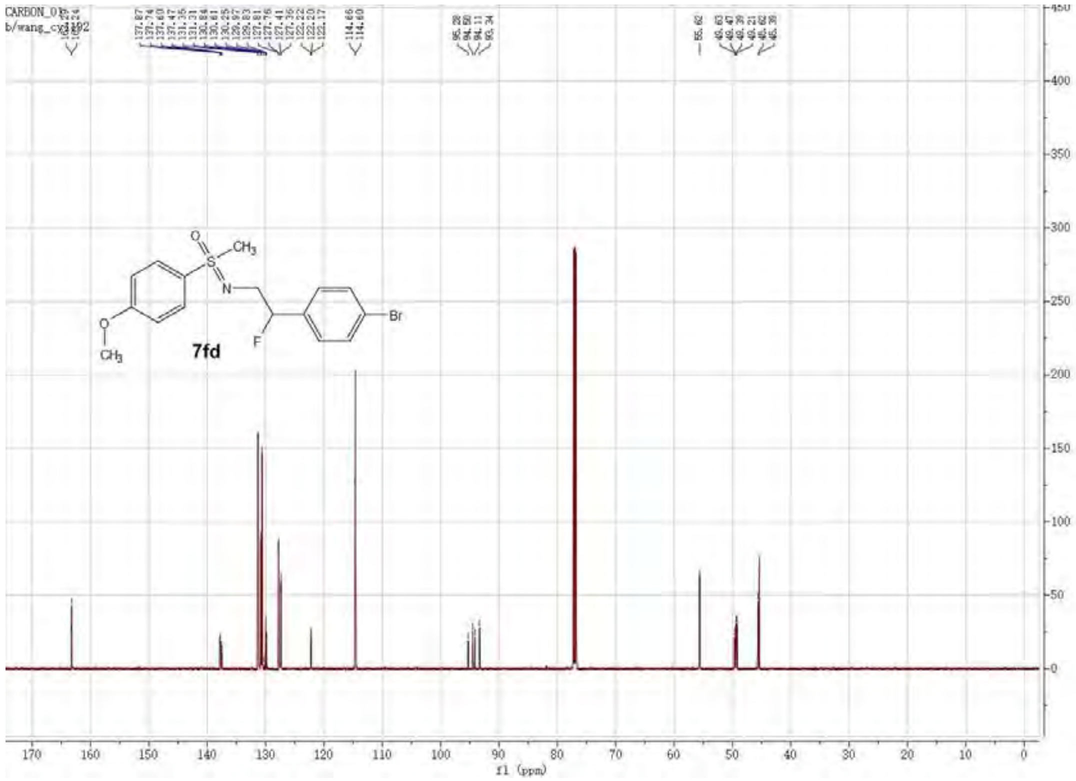

^{13}C NMR spectrum of compound **7fd** (151 MHz, $CDCl_3$)

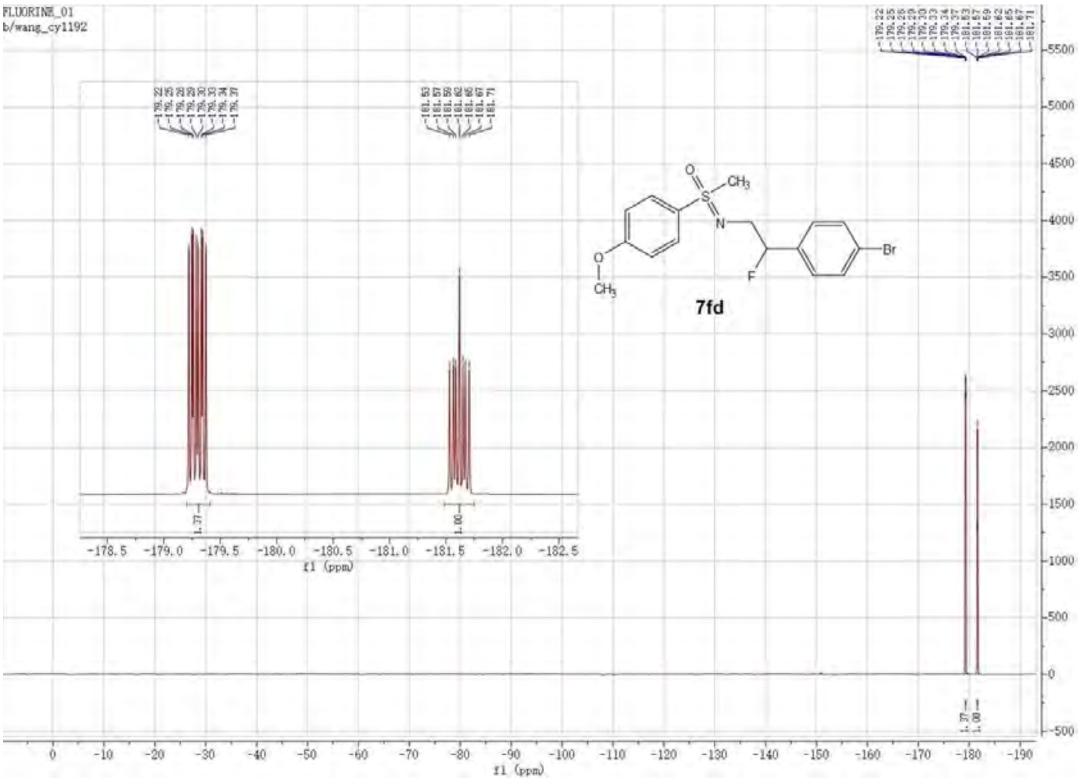

^{19}F NMR spectrum of compound **7fd** (564 MHz, $CDCl_3$)

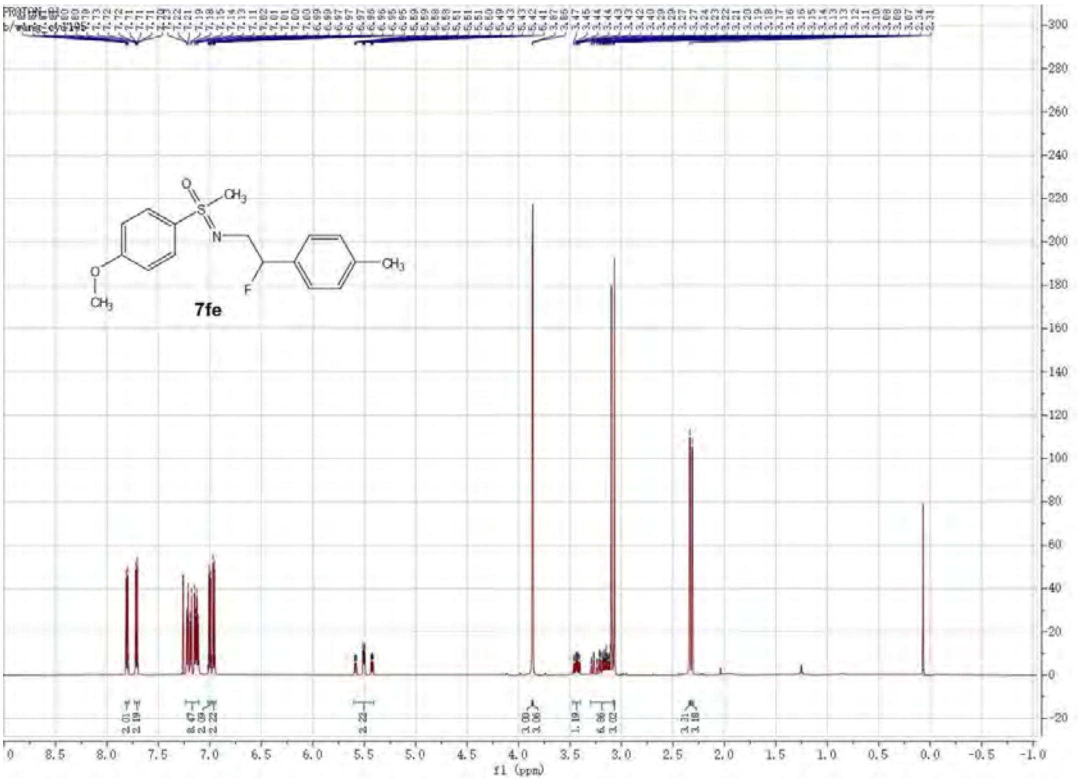

^{1}H NMR spectrum of compound **7fe** (600 MHz, $CDCl_3$)

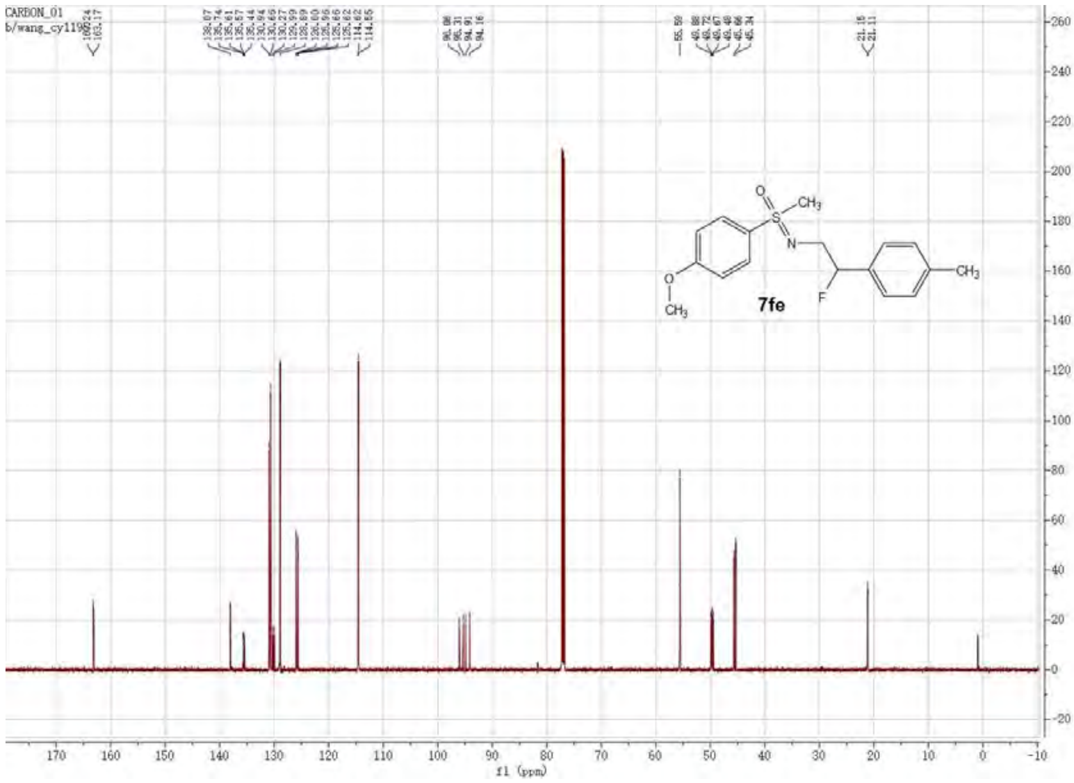

^{13}C NMR spectrum of compound **7fe** (151 MHz, $CDCl_3$)

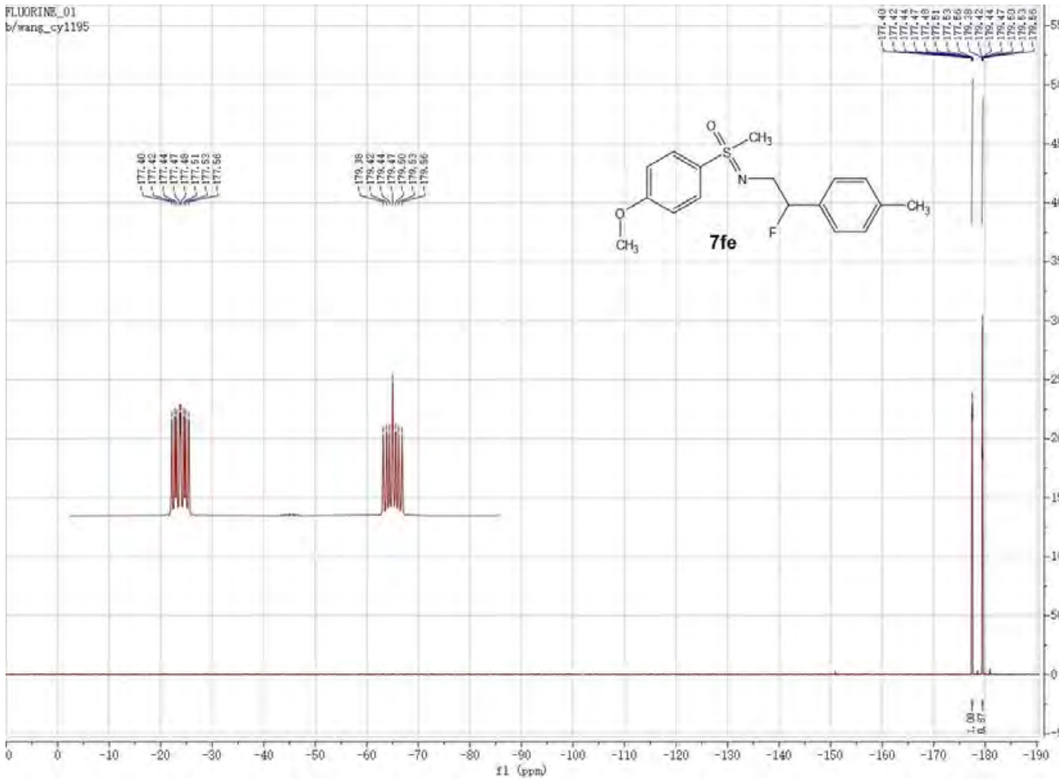

^{19}F NMR spectrum of compound **7fe** (564 MHz, $CDCl_3$)

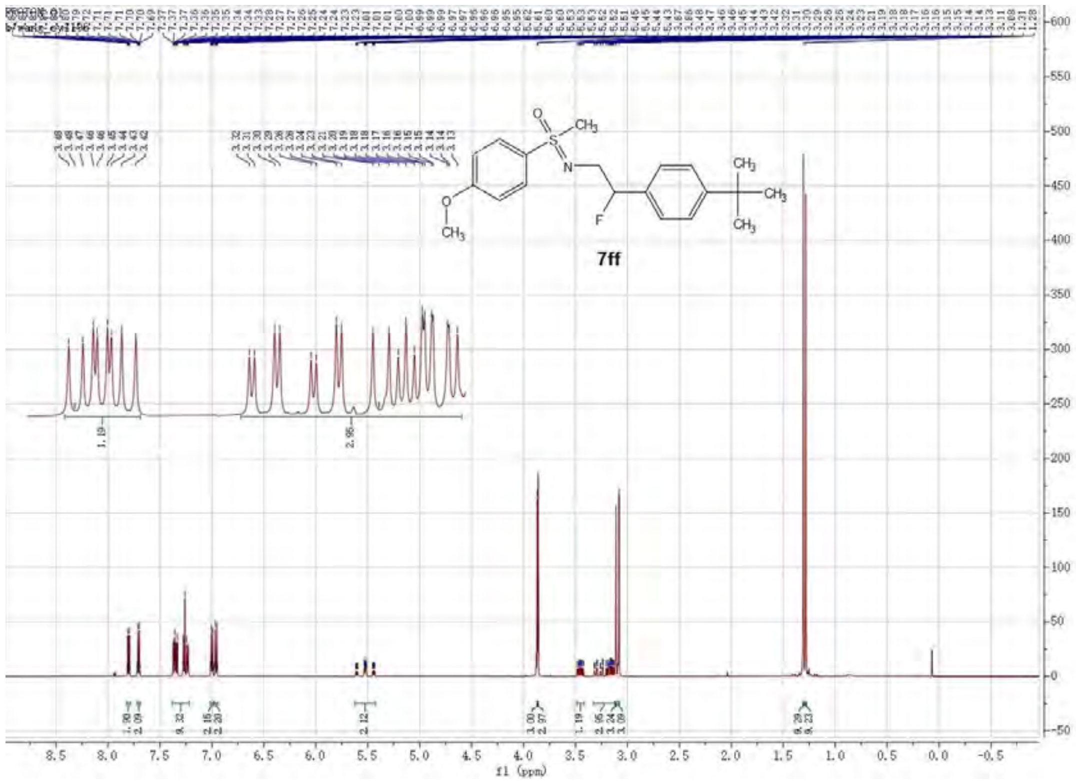

^{1}H NMR spectrum of compound **7ff** (600 MHz, $CDCl_3$)

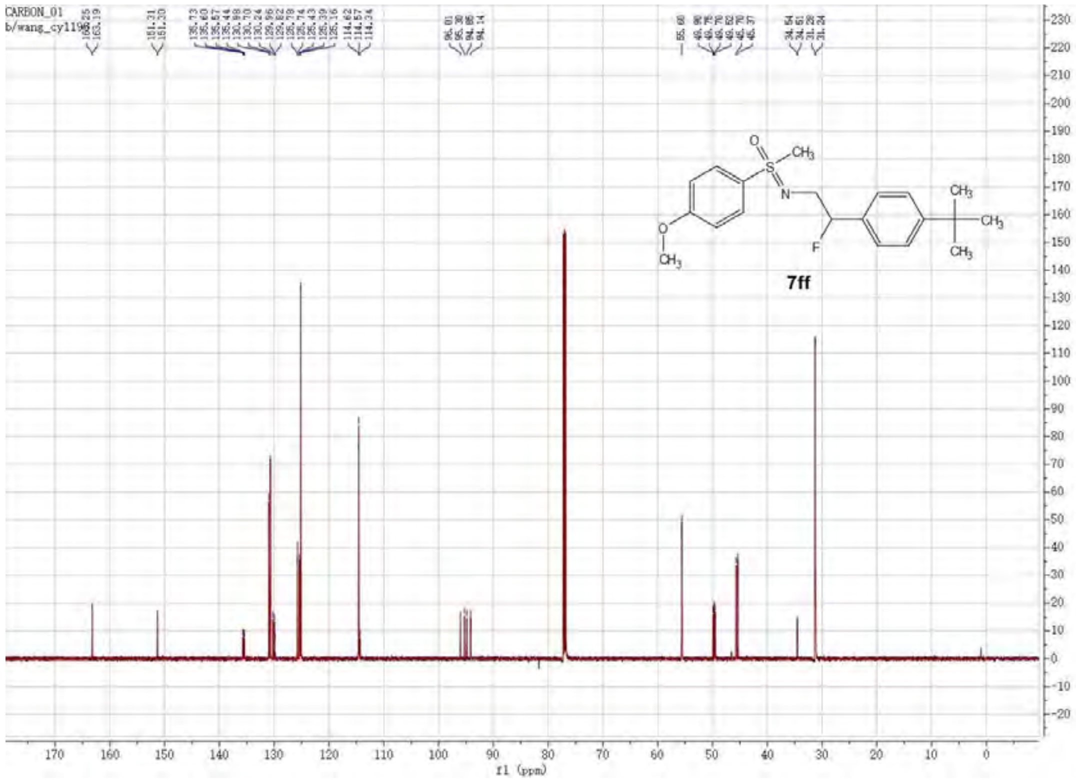

^{13}C NMR spectrum of compound **7ff** (151 MHz, $CDCl_3$)

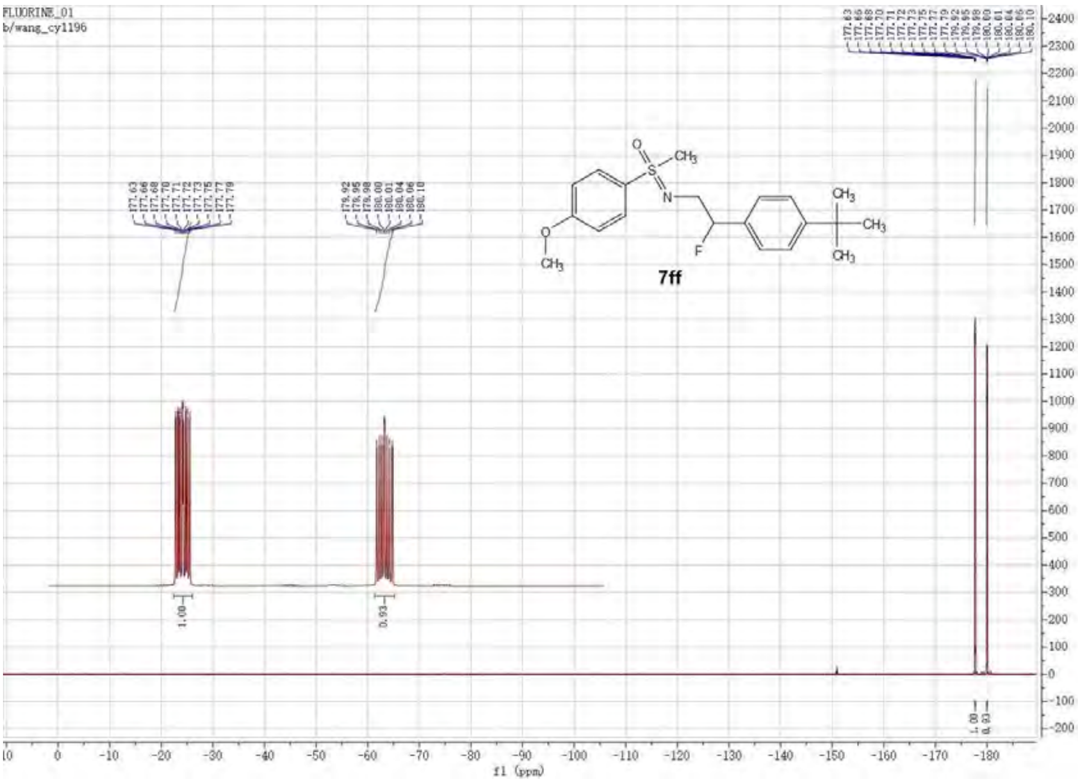

^{19}F NMR spectrum of compound **7ff** (564 MHz, $CDCl_3$)

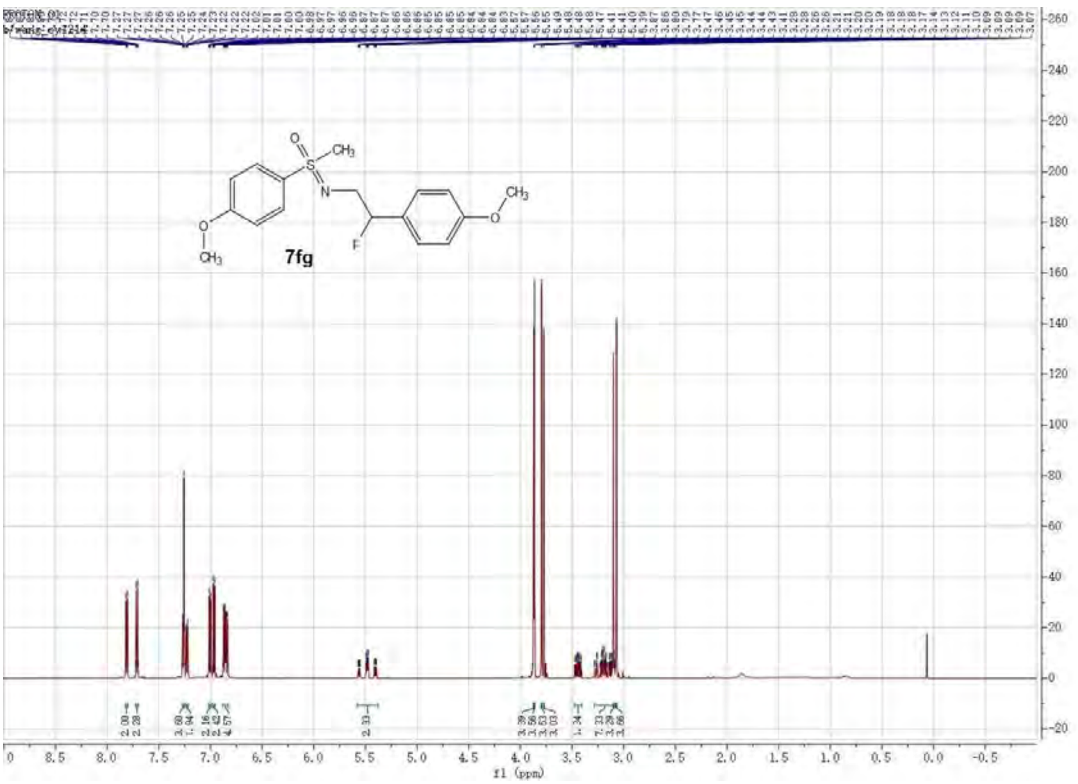

^{1}H NMR spectrum of compound **7fg** (600 MHz, $CDCl_3$)

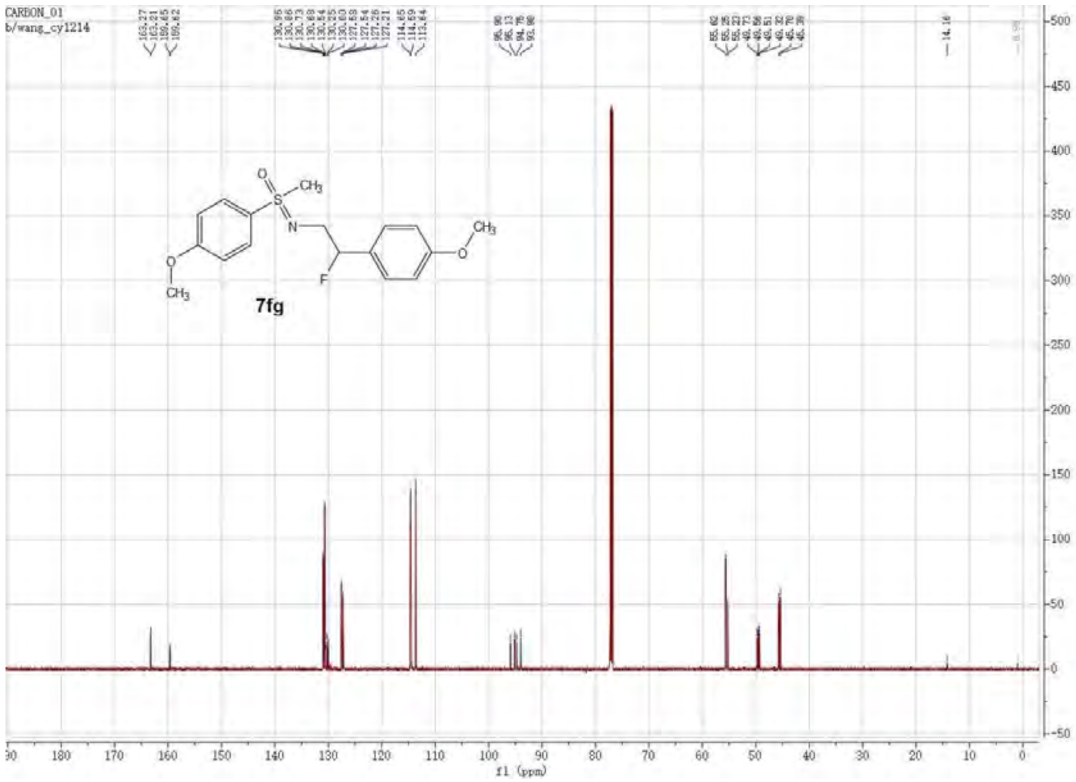

^{13}C NMR spectrum of compound **7fg** (151 MHz, $CDCl_3$)

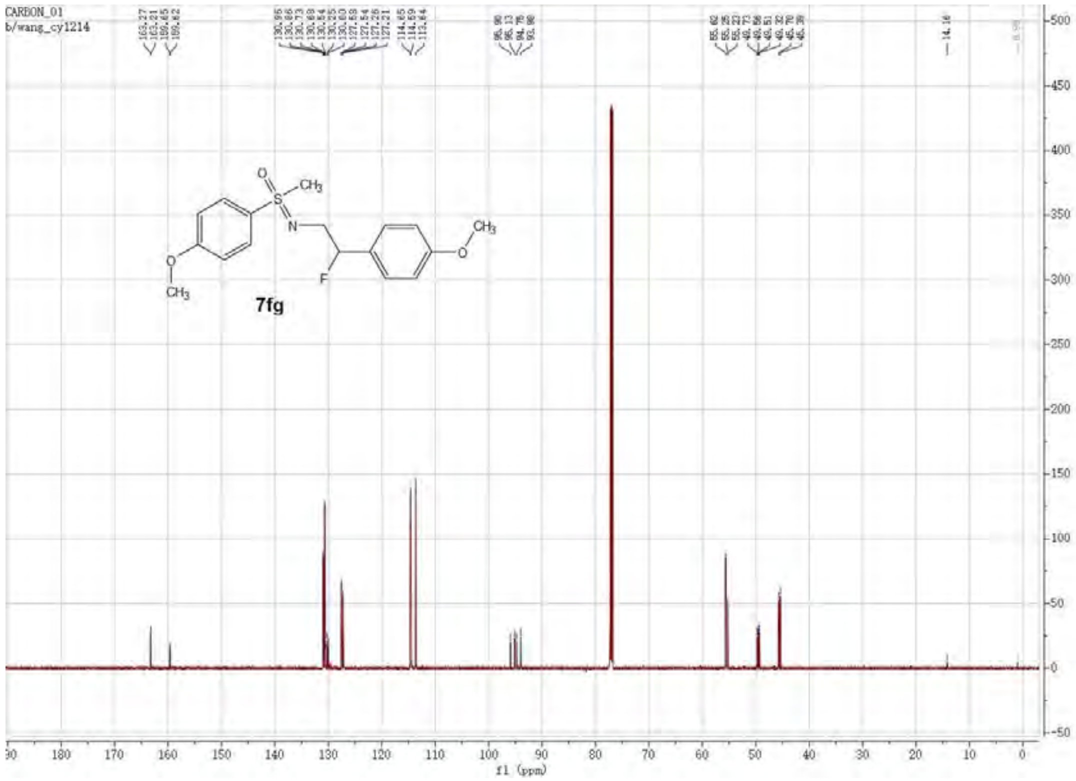

^{19}F NMR spectrum of compound **7fg** (564 MHz, $CDCl_3$)

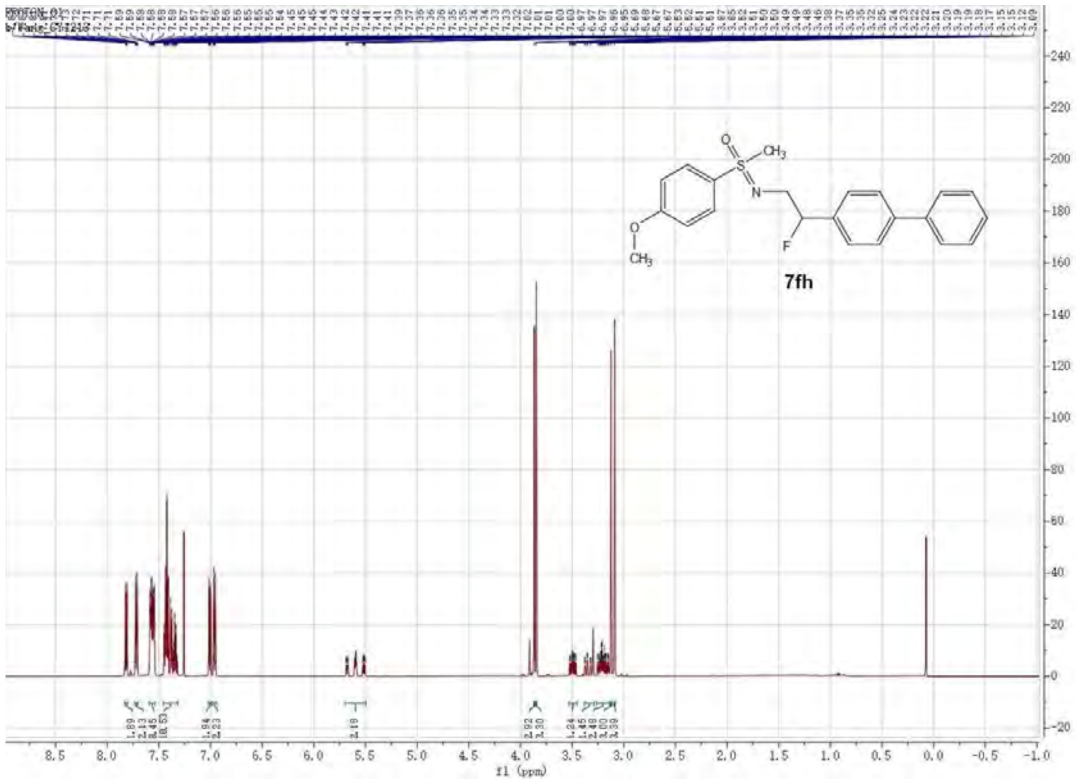

^{1}H NMR spectrum of compound **7fh** (600 MHz, $CDCl_3$)

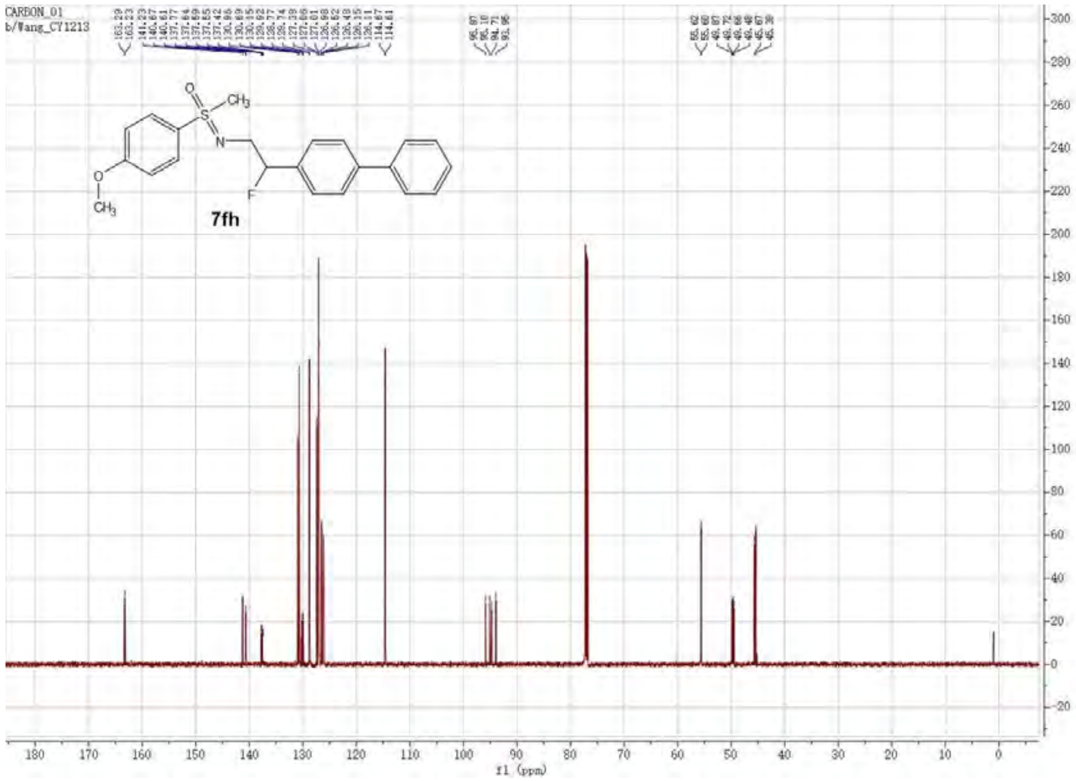

^{13}C NMR spectrum of compound **7fh** (151 MHz, $CDCl_3$)

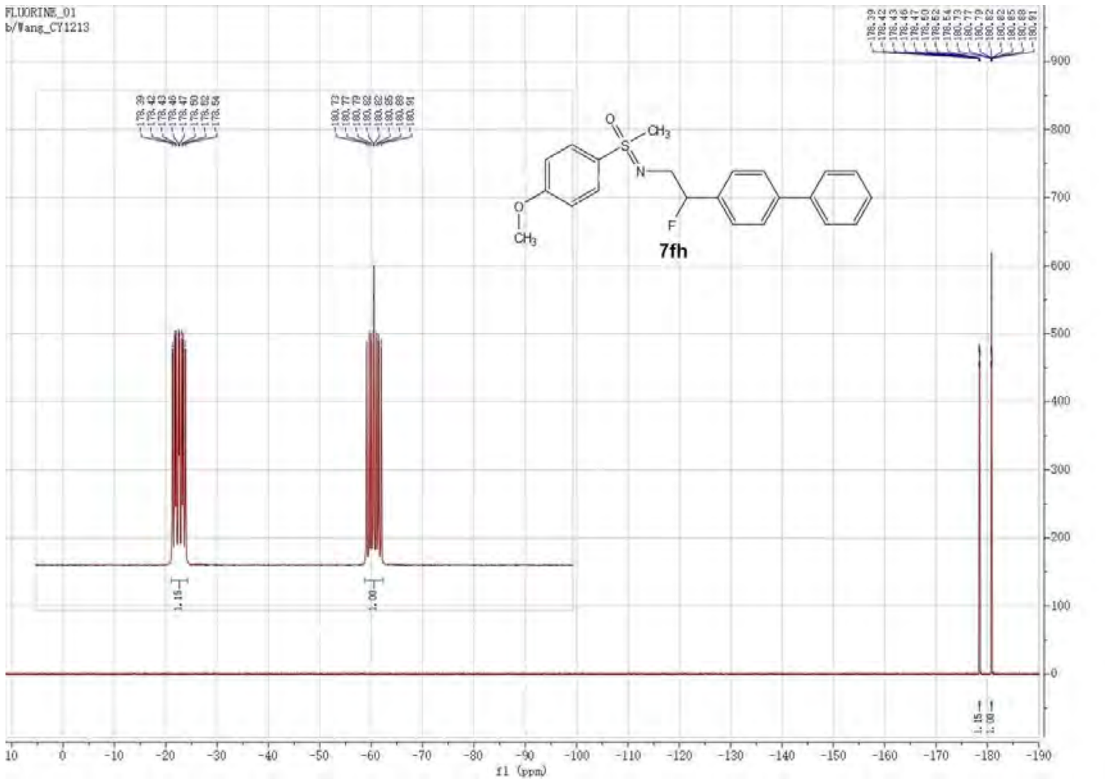

^{19}F NMR spectrum of compound **7fh** (564 MHz, $CDCl_3$)

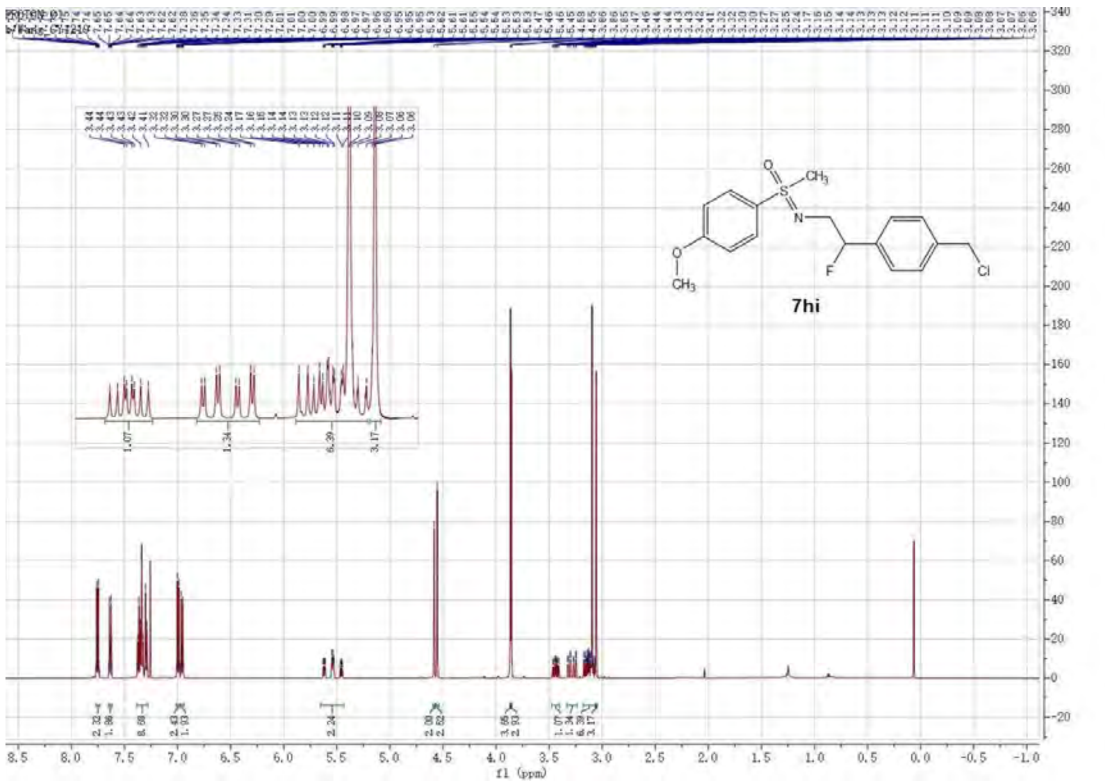

^{1}H NMR spectrum of compound **7fi** (600 MHz, $CDCl_3$)

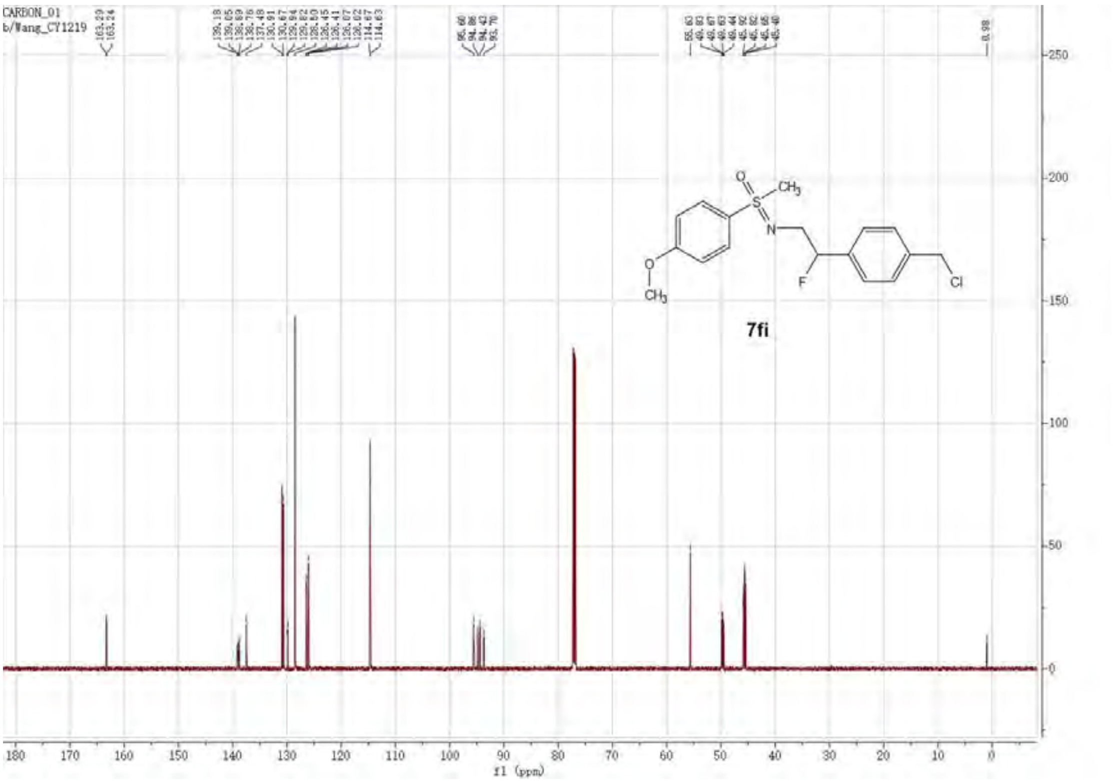

^{13}C NMR spectrum of compound **7fi** (151 MHz, CDCl$_3$)

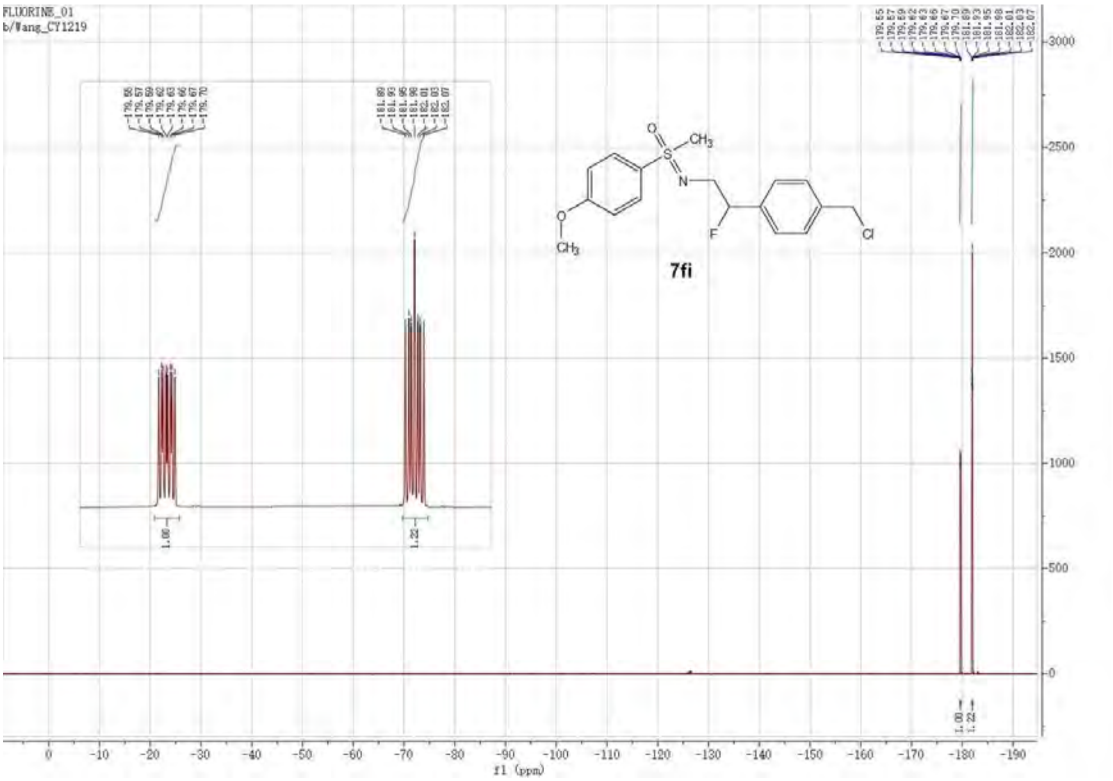

^{19}F NMR spectrum of compound **7fi** (564 MHz, CDCl$_3$)

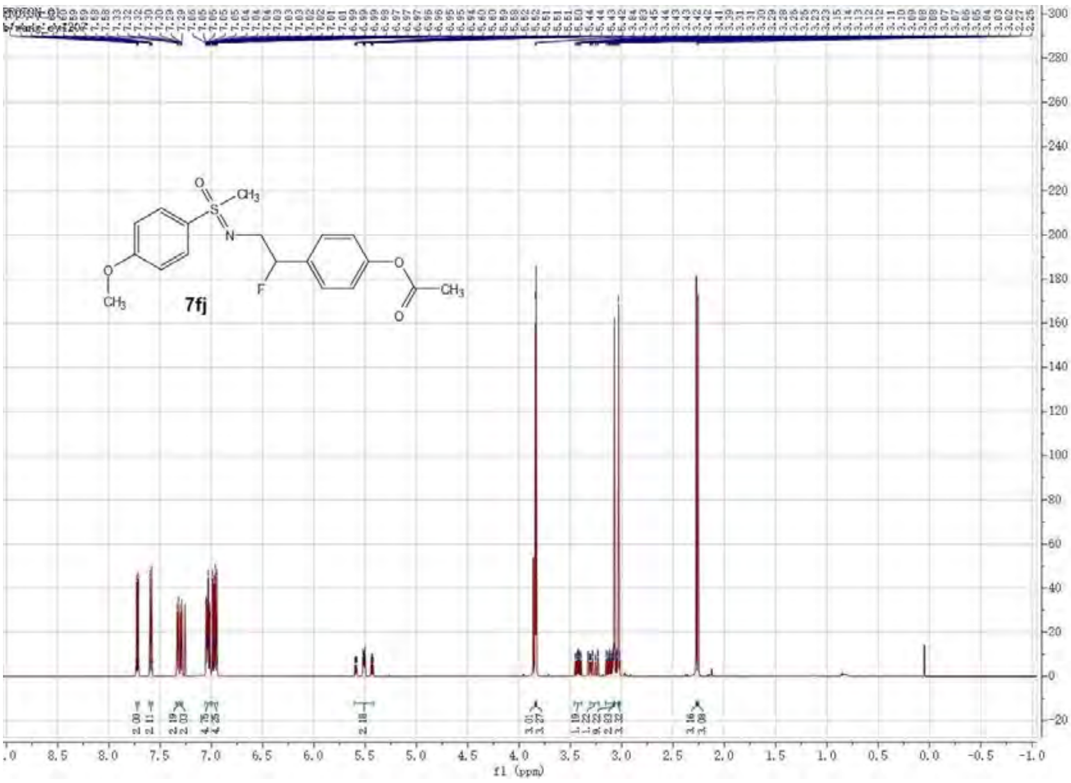

^{1}H NMR spectrum of compound **7fj** (600 MHz, $CDCl_3$)

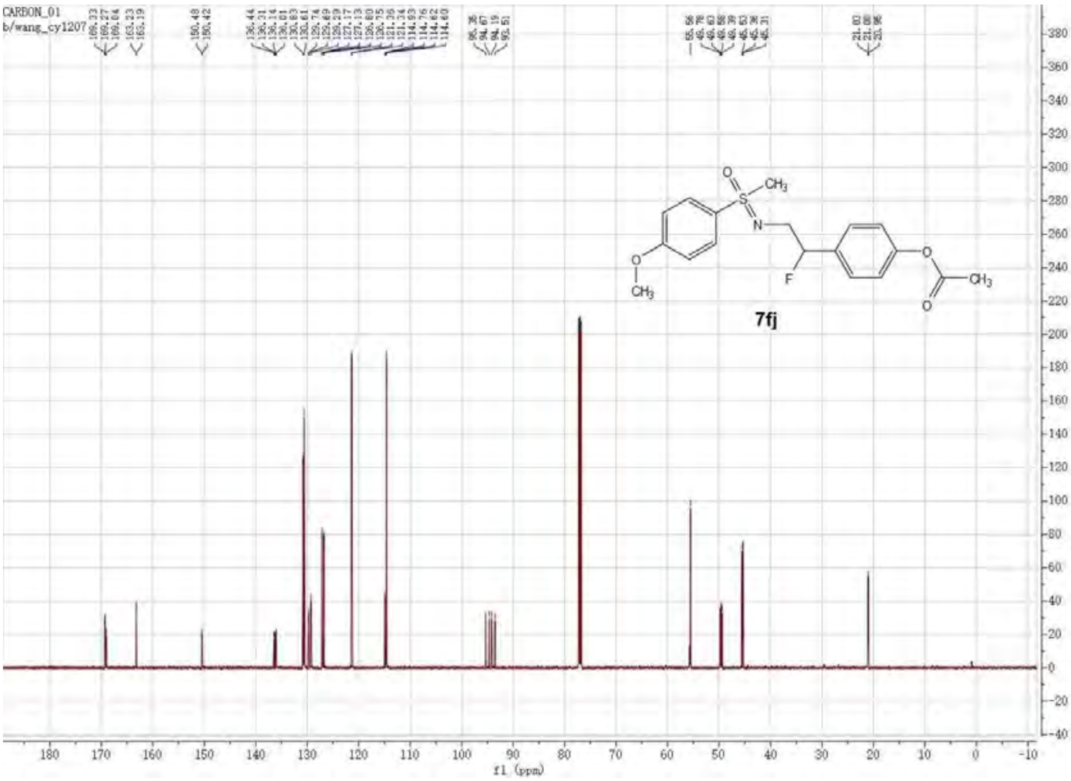

^{13}C NMR spectrum of compound **7fj** (151 MHz, $CDCl_3$)

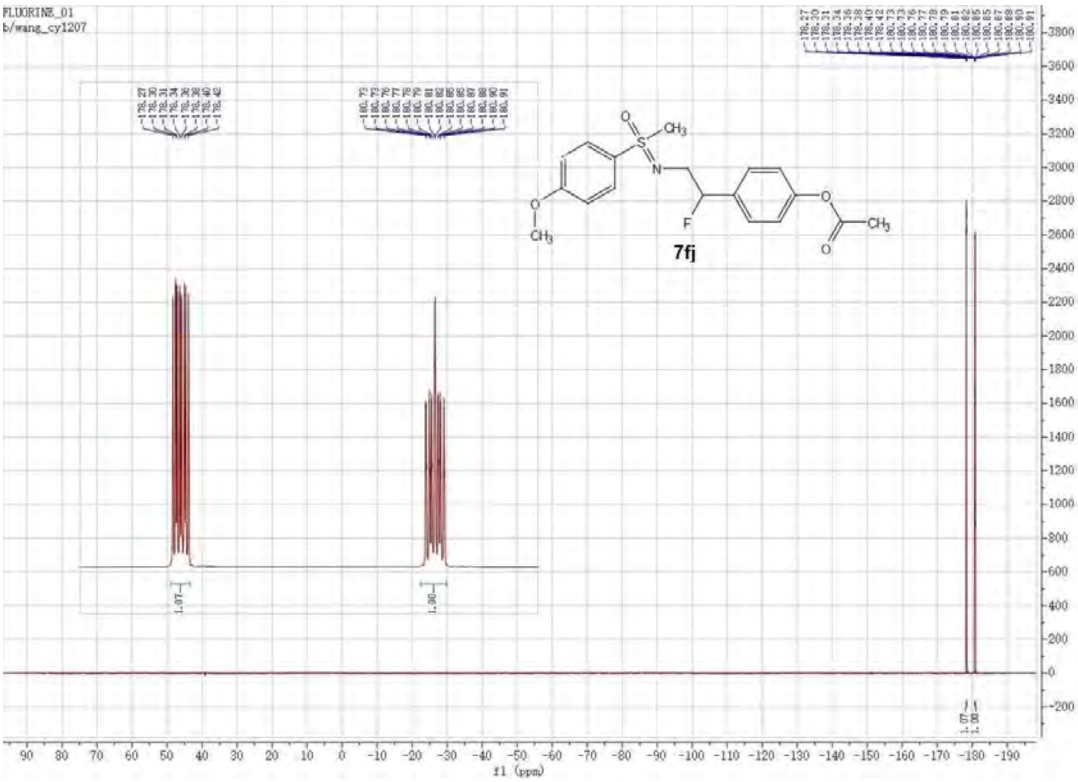

^{19}F NMR spectrum of compound **7fj** (564 MHz, $CDCl_3$)

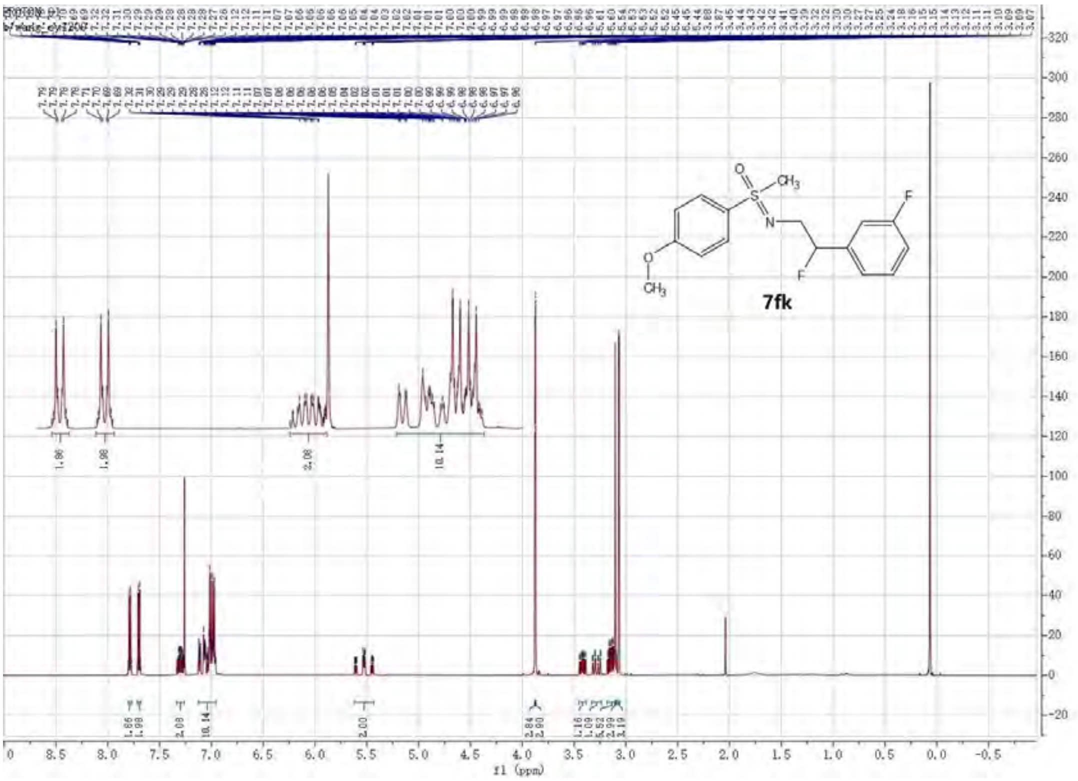

^{1}H NMR spectrum of compound **7fk** (600 MHz, $CDCl_3$)

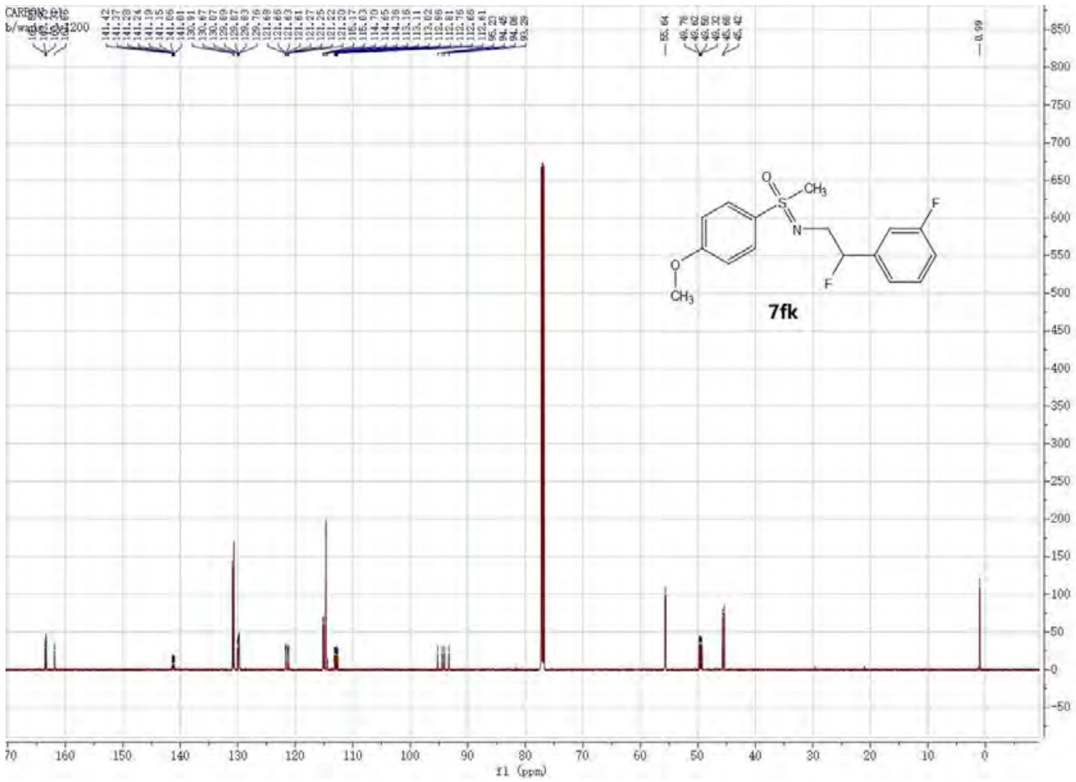

^{13}C NMR spectrum of compound **7fk** (151 MHz, CDCl$_3$)

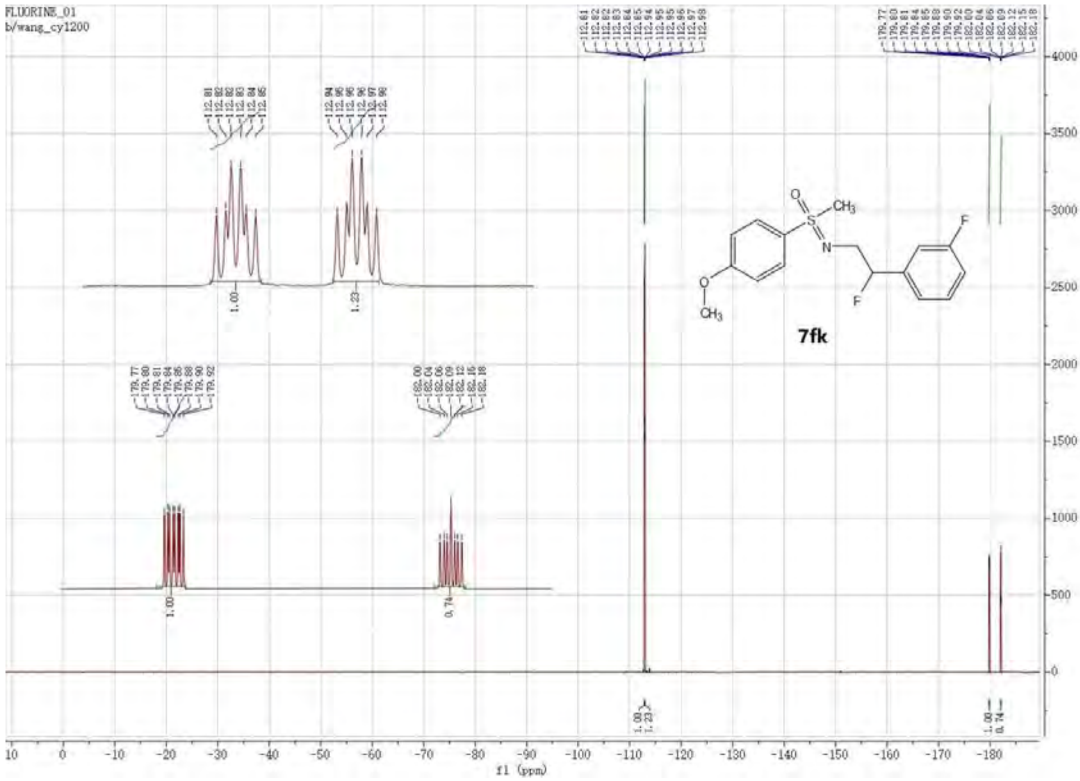

^{19}F NMR spectrum of compound **7fk** (564 MHz, CDCl$_3$)

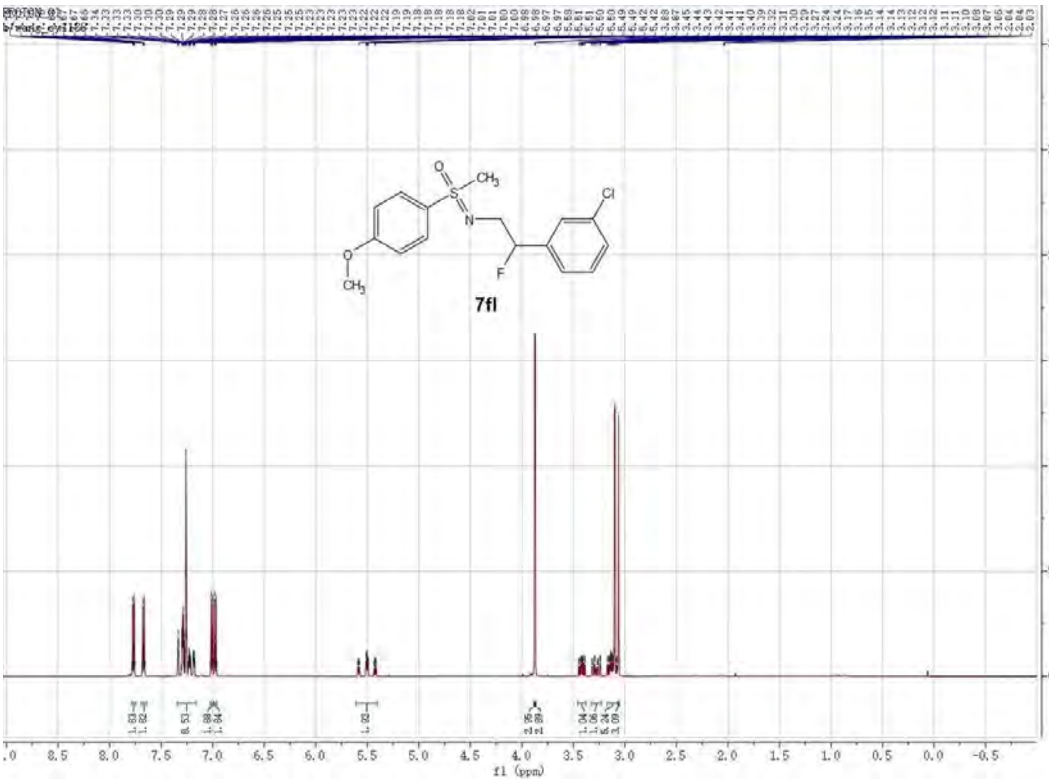

^{1}H NMR spectrum of compound **7fl** (600 MHz, $CDCl_3$)

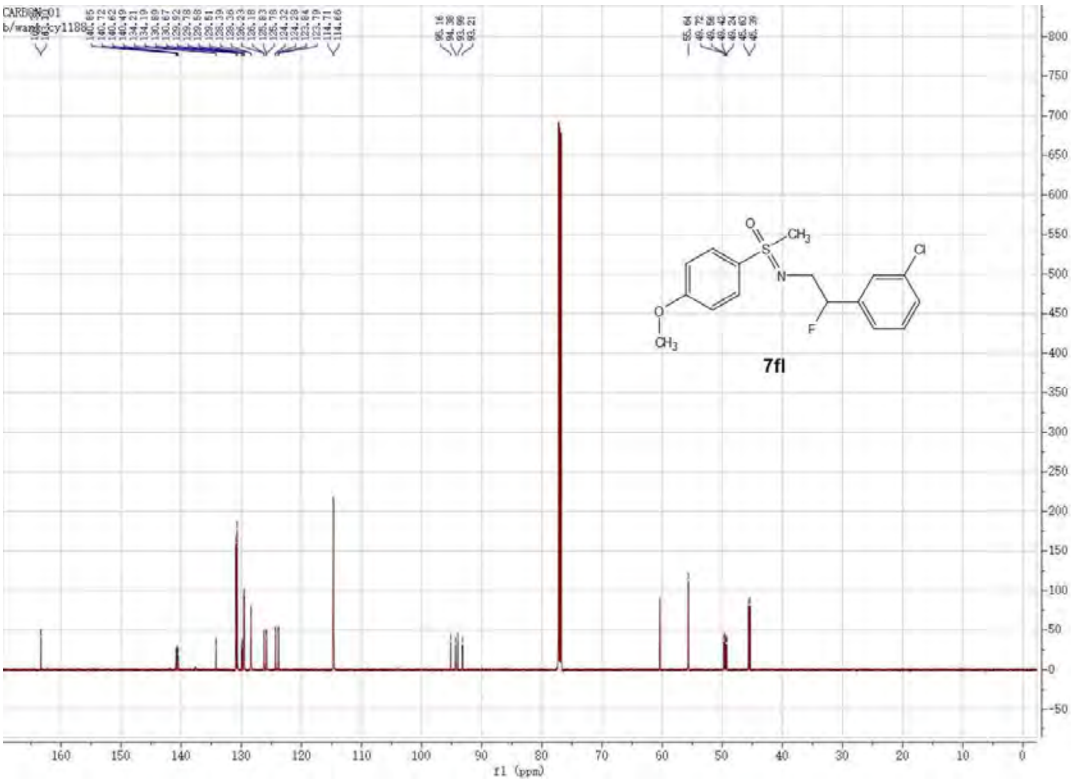

^{13}C NMR spectrum of compound **7fl** (151 MHz, $CDCl_3$)

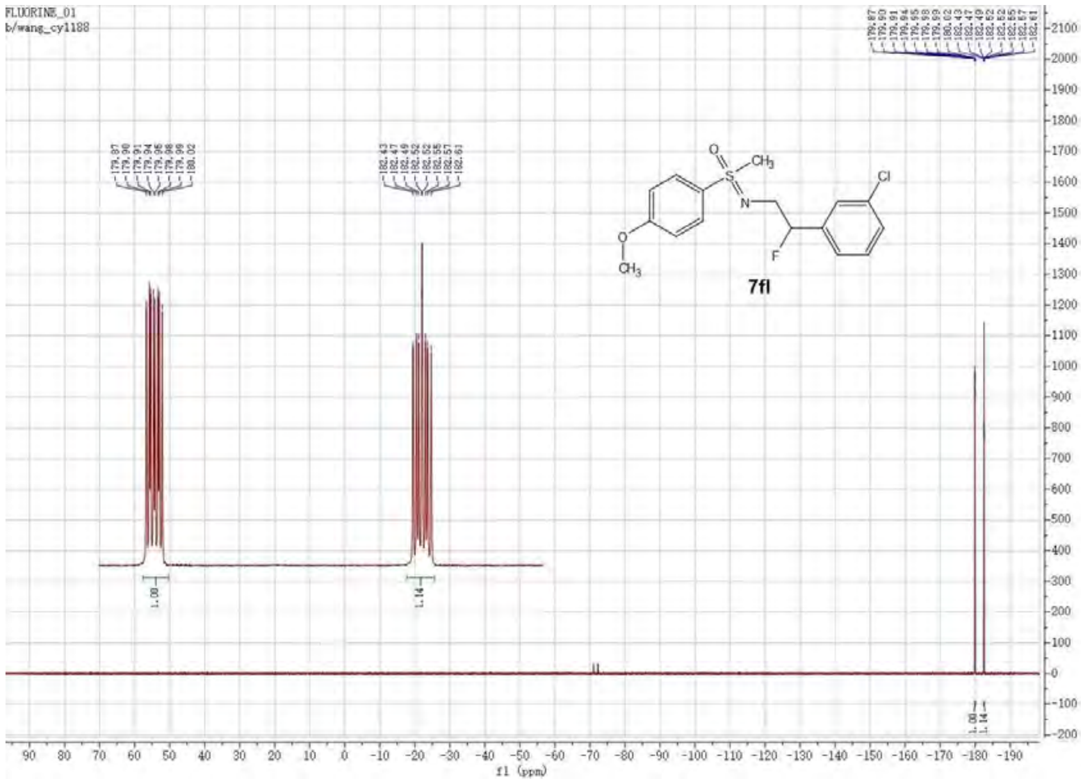

^{19}F NMR spectrum of compound **7fl** (564 MHz, $CDCl_3$)

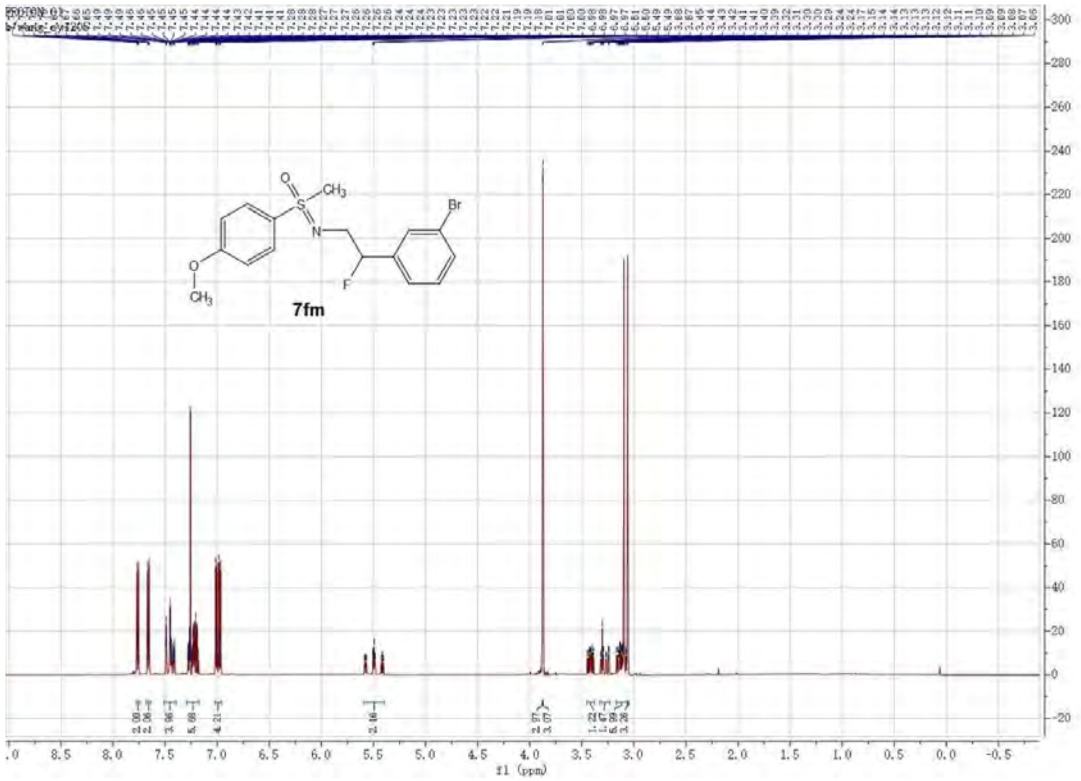

^{1}H NMR spectrum of compound **7fm** (600 MHz, $CDCl_3$)

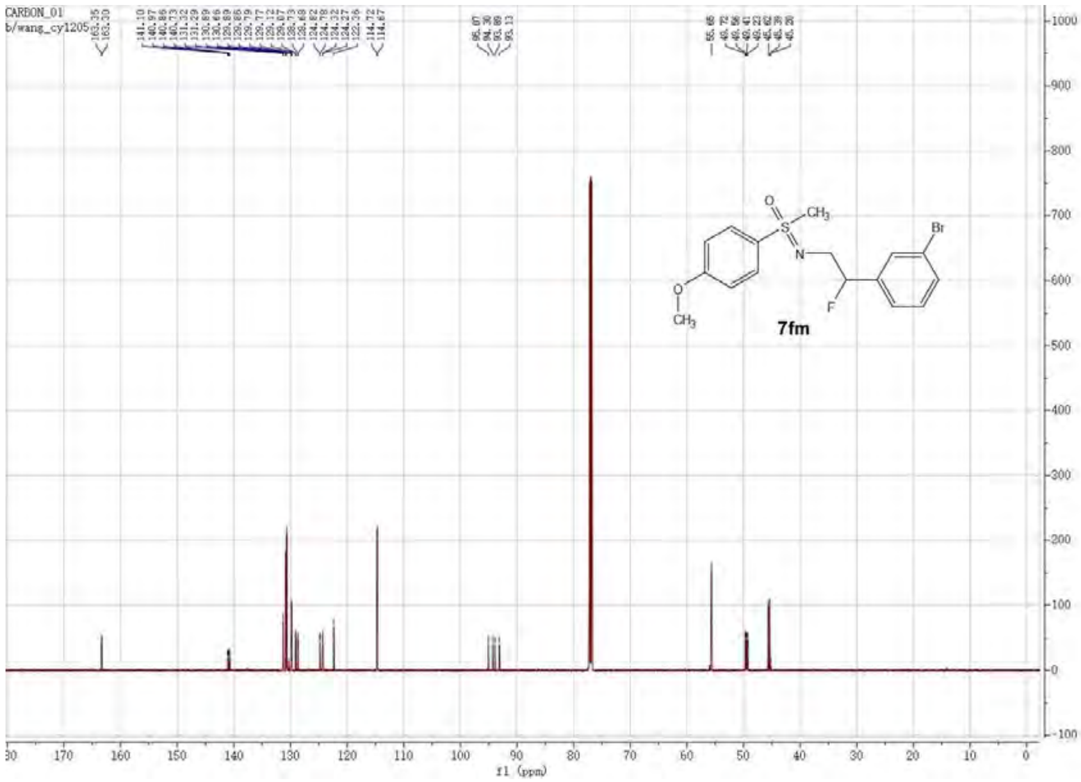

^{13}C NMR spectrum of compound **7fm** (151 MHz, $CDCl_3$)

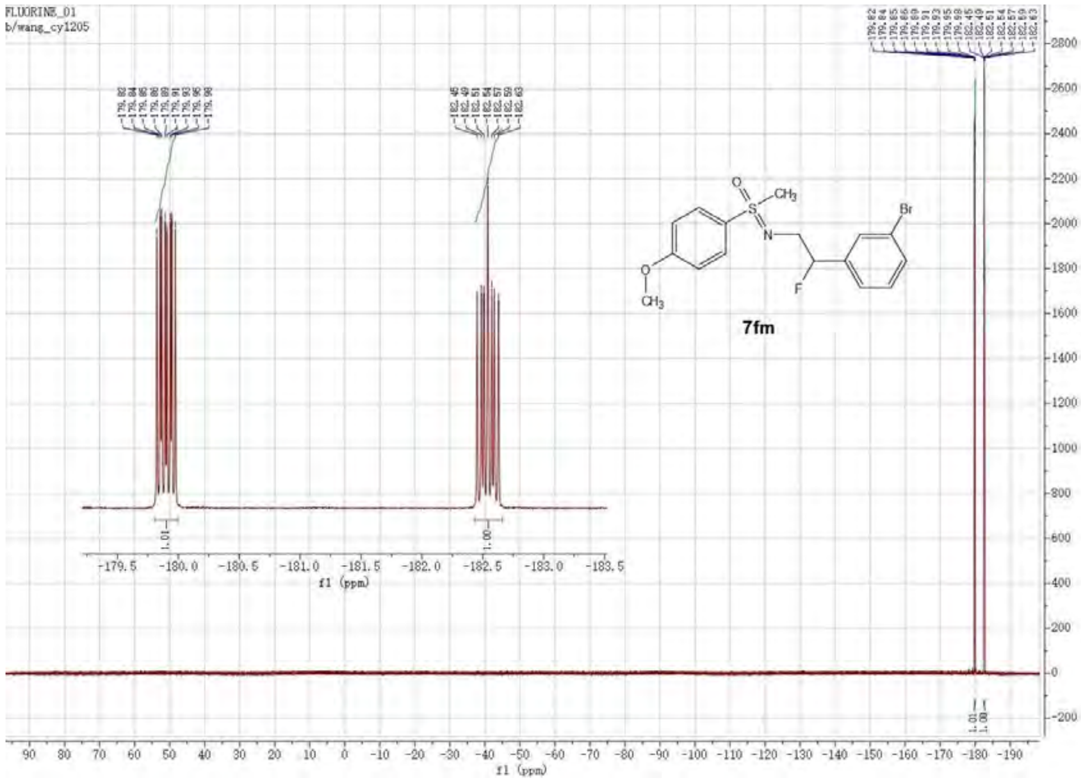

^{19}F NMR spectrum of compound **7fm** (564 MHz, $CDCl_3$)

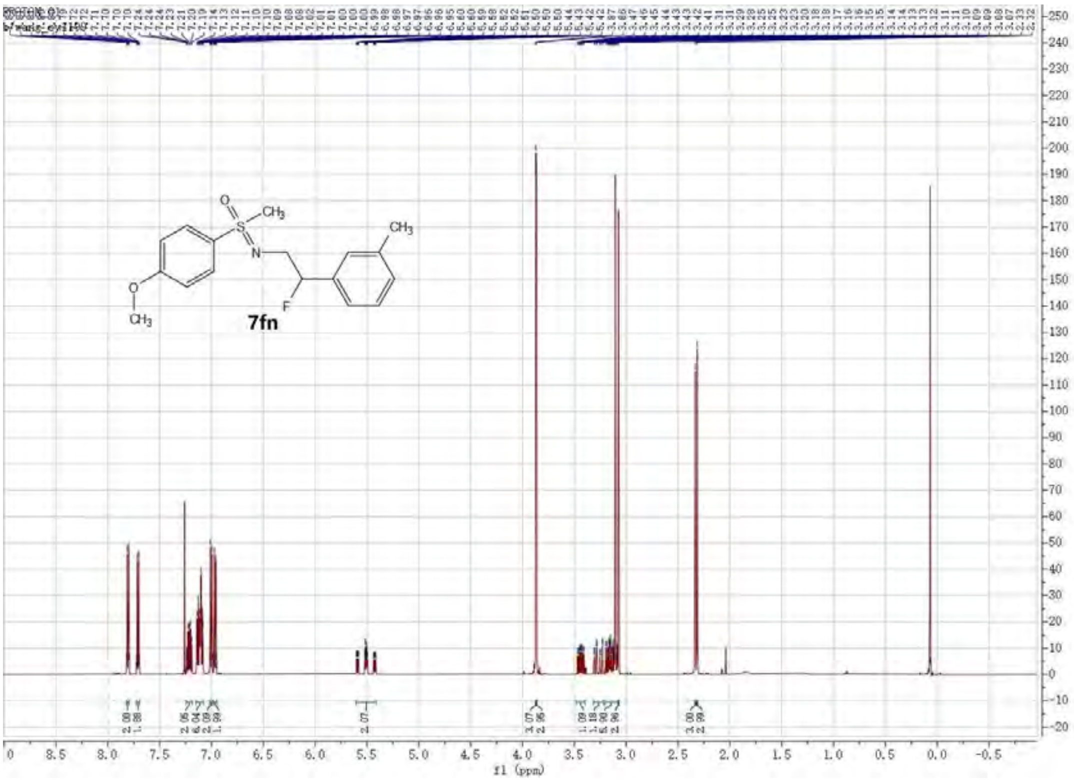

^{1}H NMR spectrum of compound **7fn** (600 MHz, $CDCl_3$)

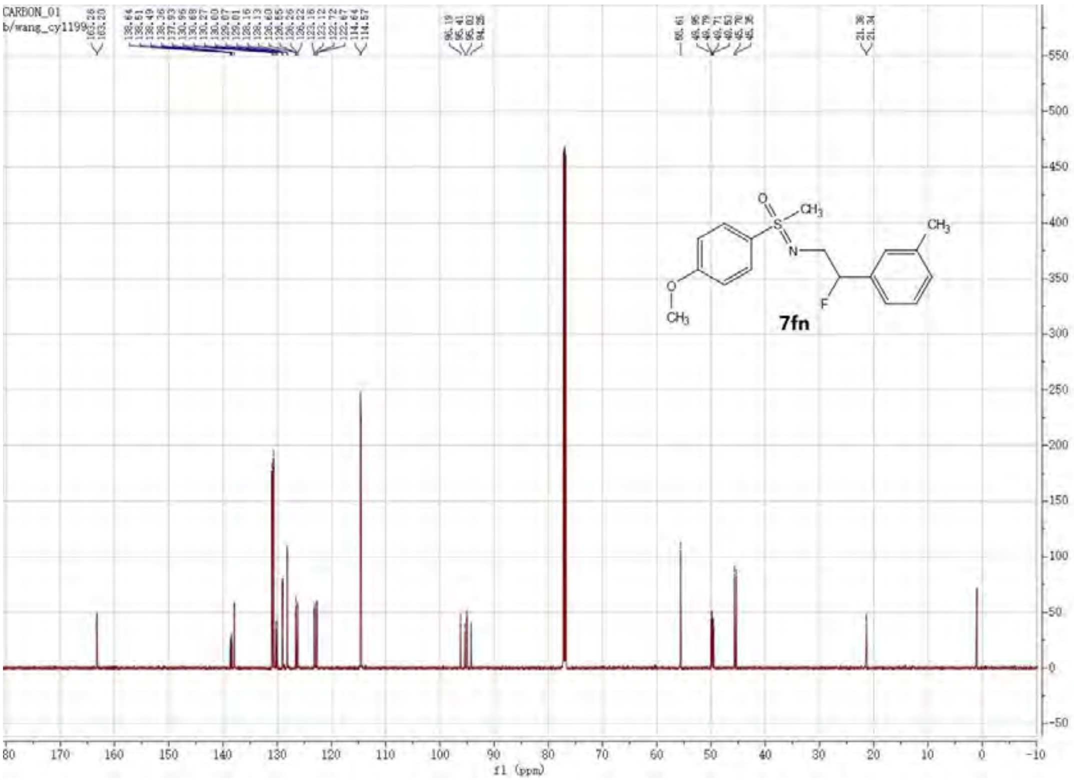

^{13}C NMR spectrum of compound **7fn** (151 MHz, $CDCl_3$)

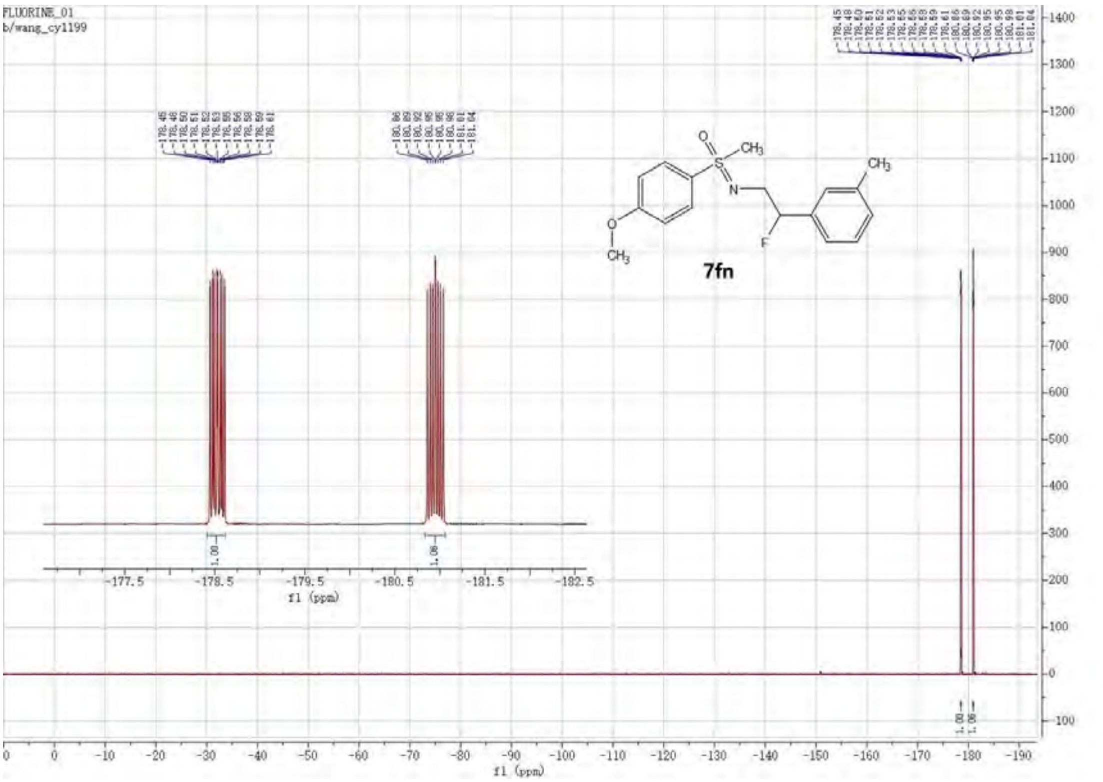

^{19}F NMR spectrum of compound **7fn** (564 MHz, $CDCl_3$)

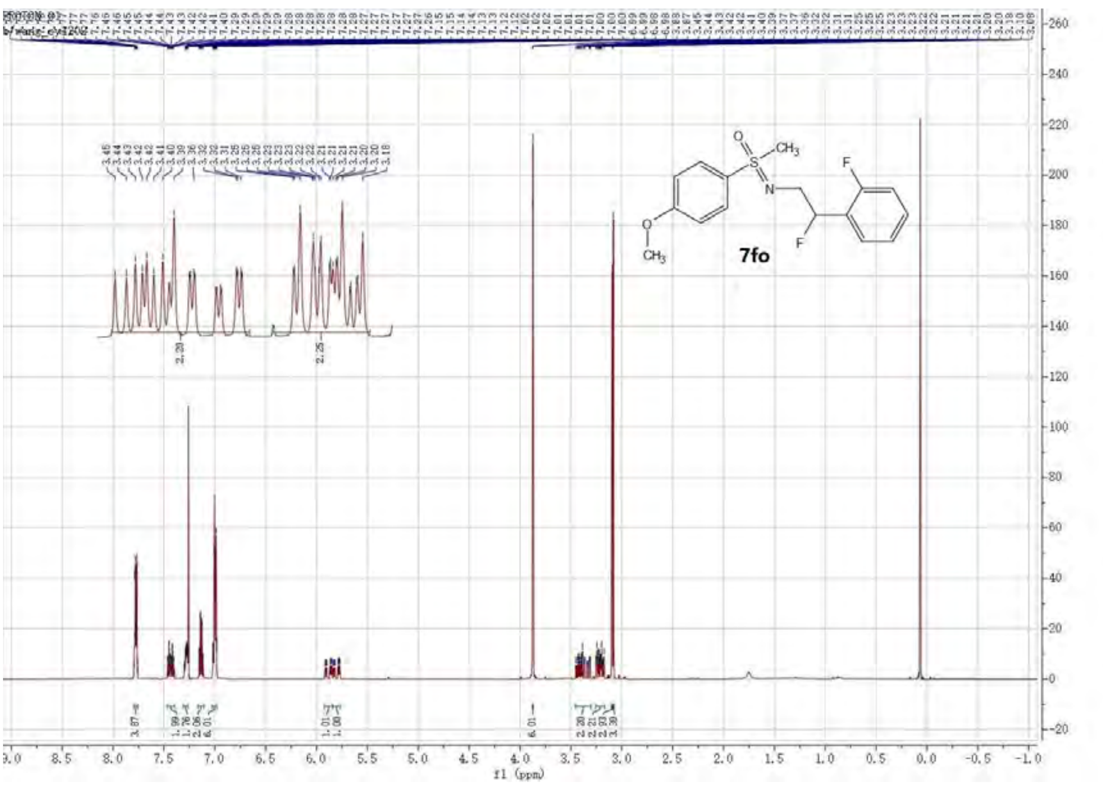

^{1}H NMR spectrum of compound **7fo** (600 MHz, $CDCl_3$)

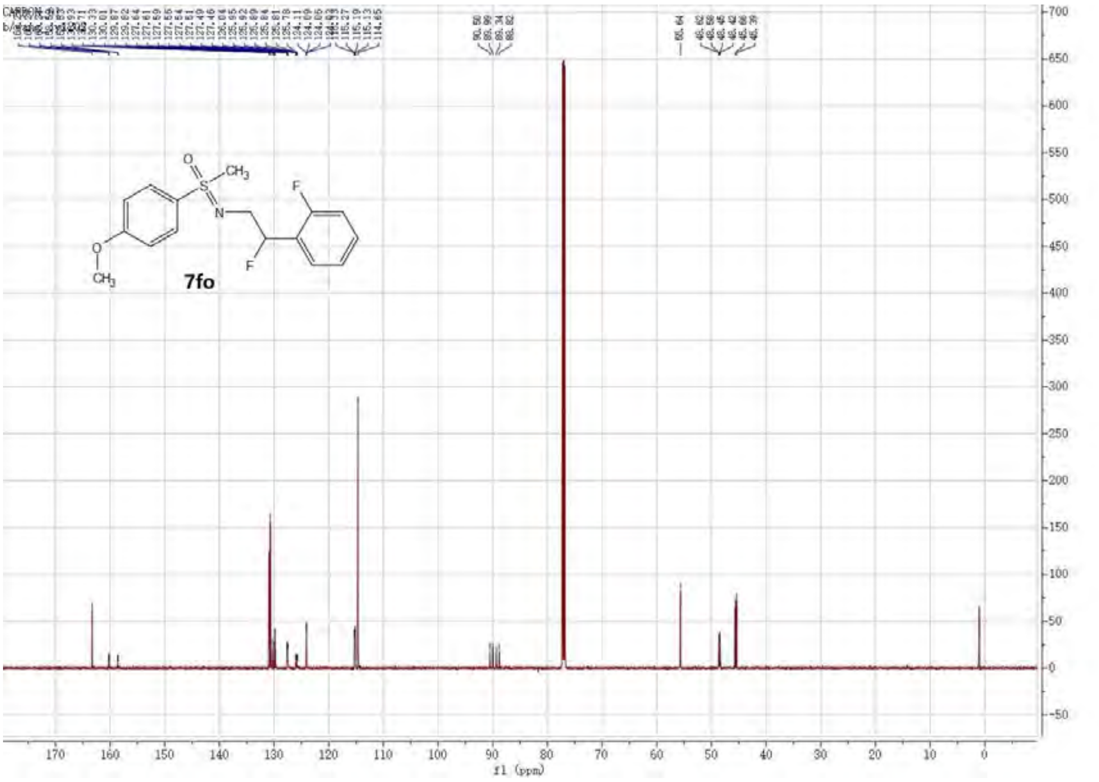

^{13}C NMR spectrum of compound **7fo** (151 MHz, $CDCl_3$)

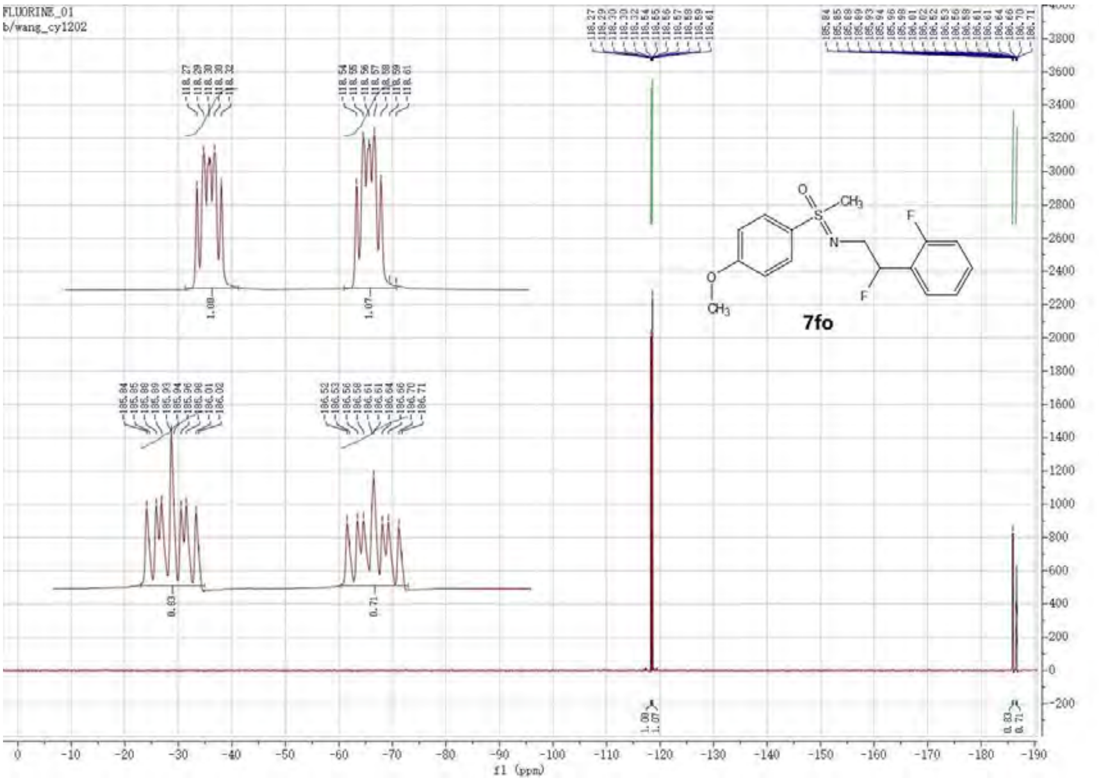

^{19}F NMR spectrum of compound **7fo** (564 MHz, $CDCl_3$)

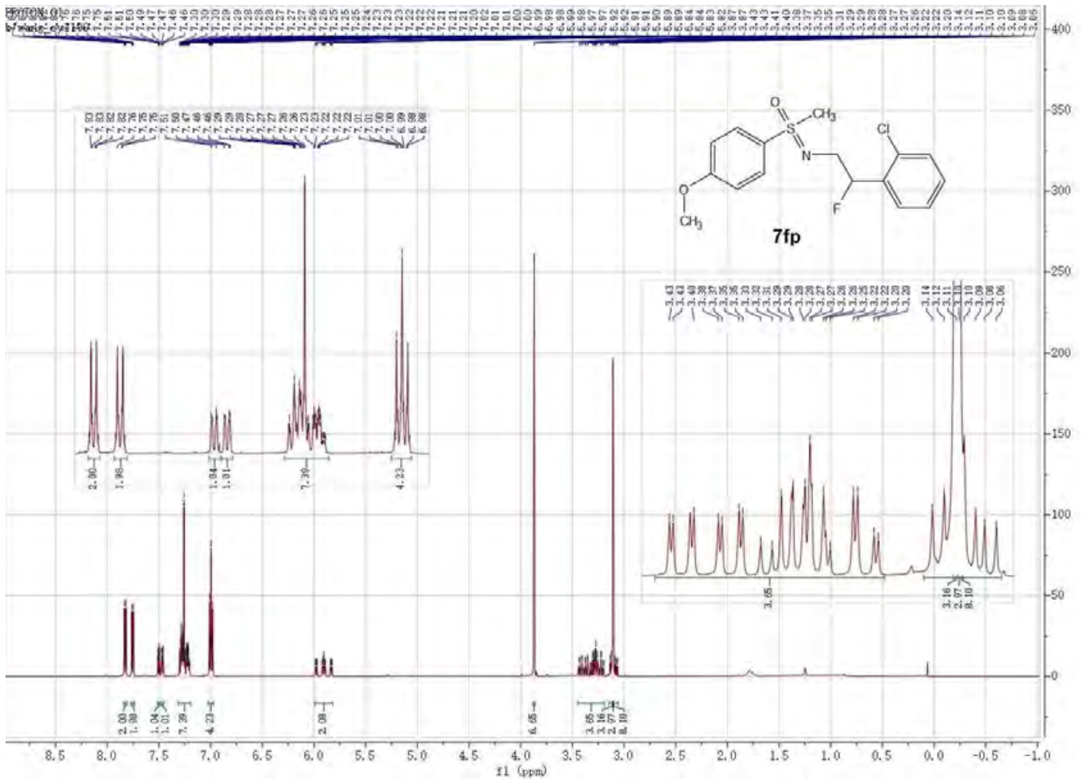

^{1}H NMR spectrum of compound **7fp** (600 MHz, $CDCl_3$)

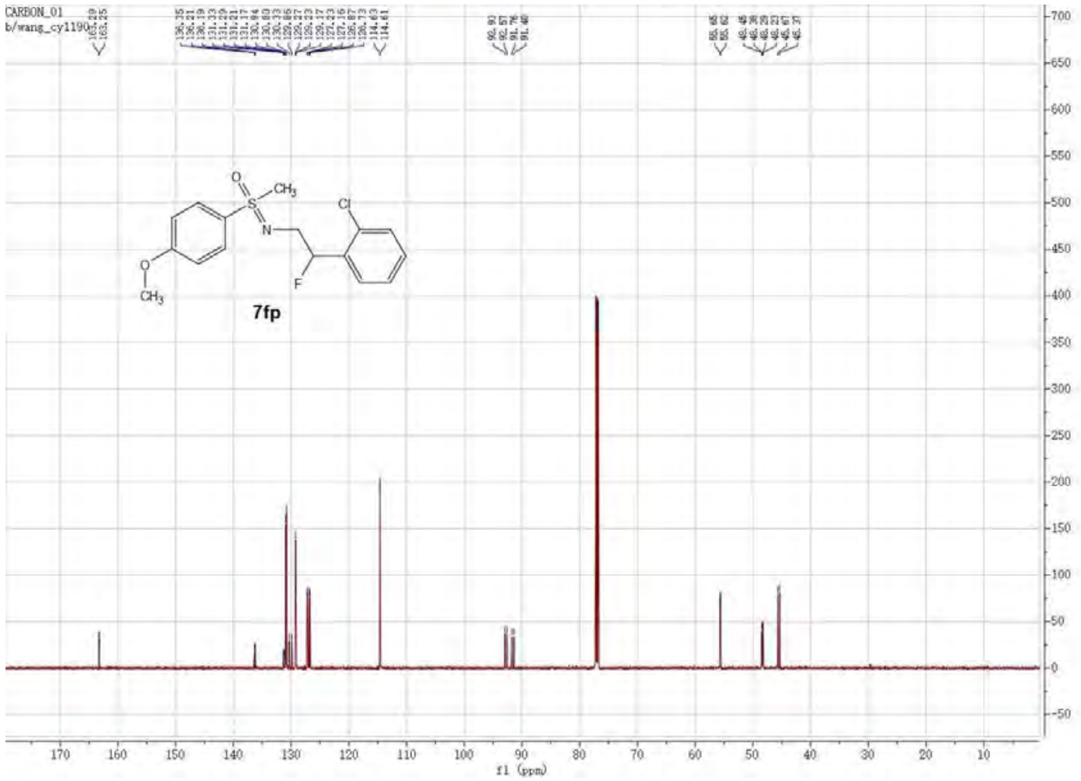

^{13}C NMR spectrum of compound **7fp** (151 MHz, $CDCl_3$)

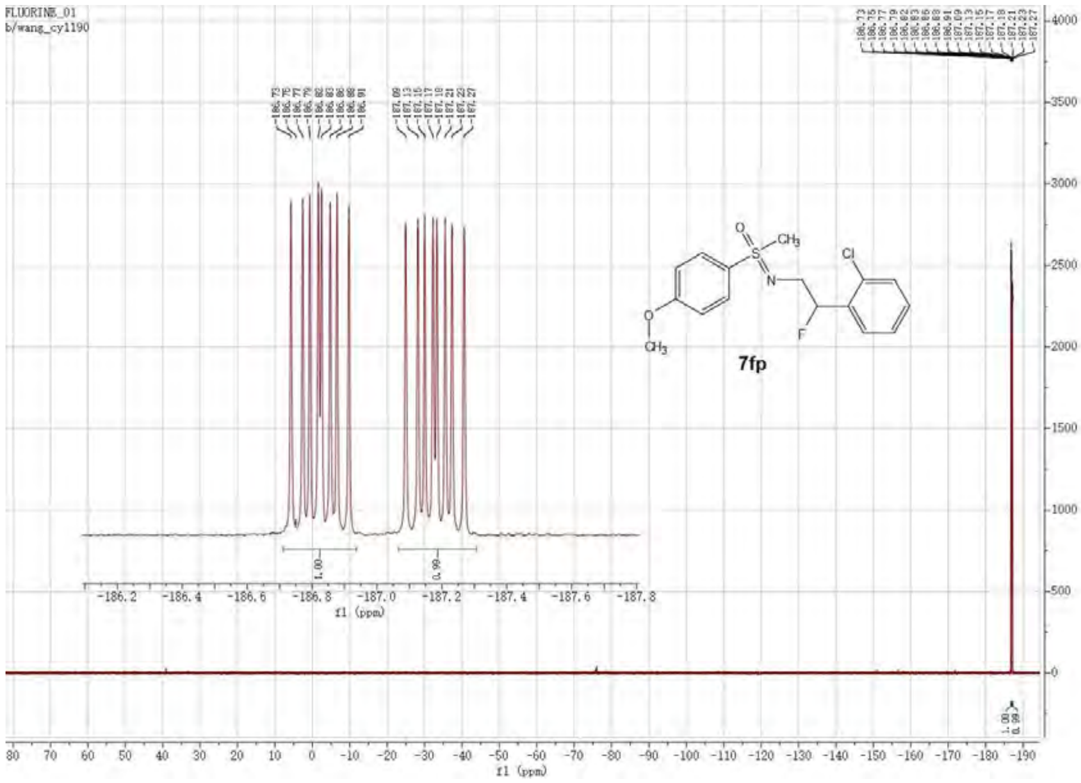

^{19}F NMR spectrum of compound **7fp** (564 MHz, $CDCl_3$)

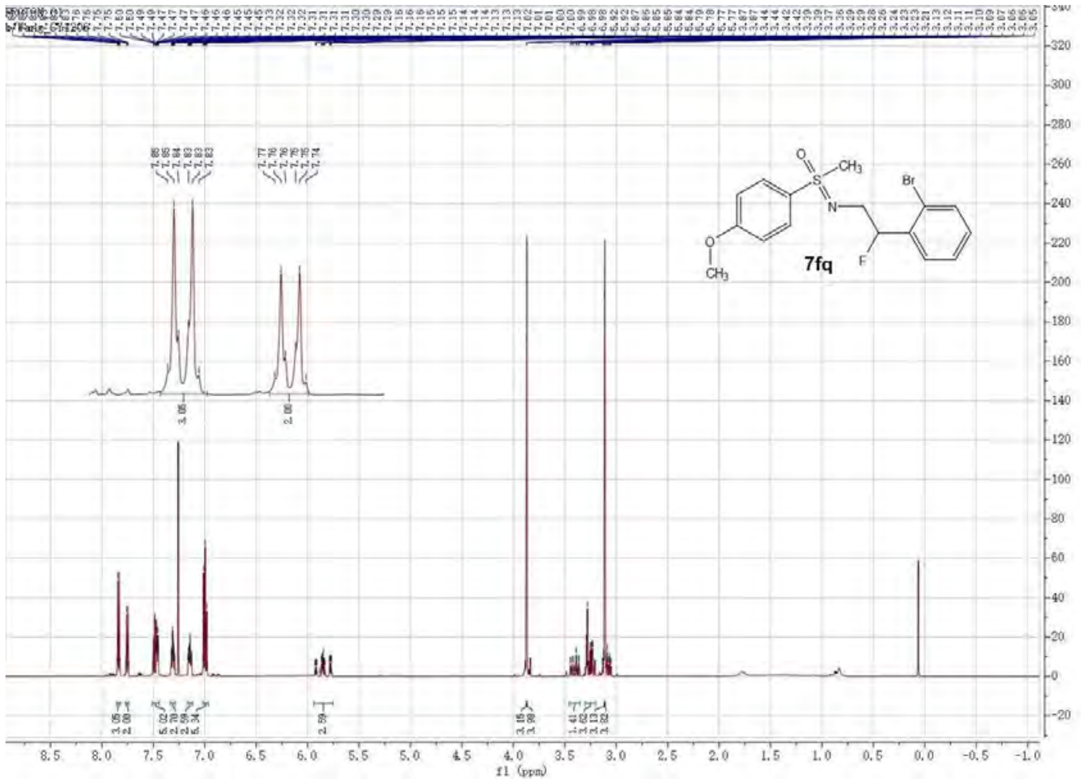

^{1}H NMR spectrum of compound **7fq** (600 MHz, $CDCl_3$)

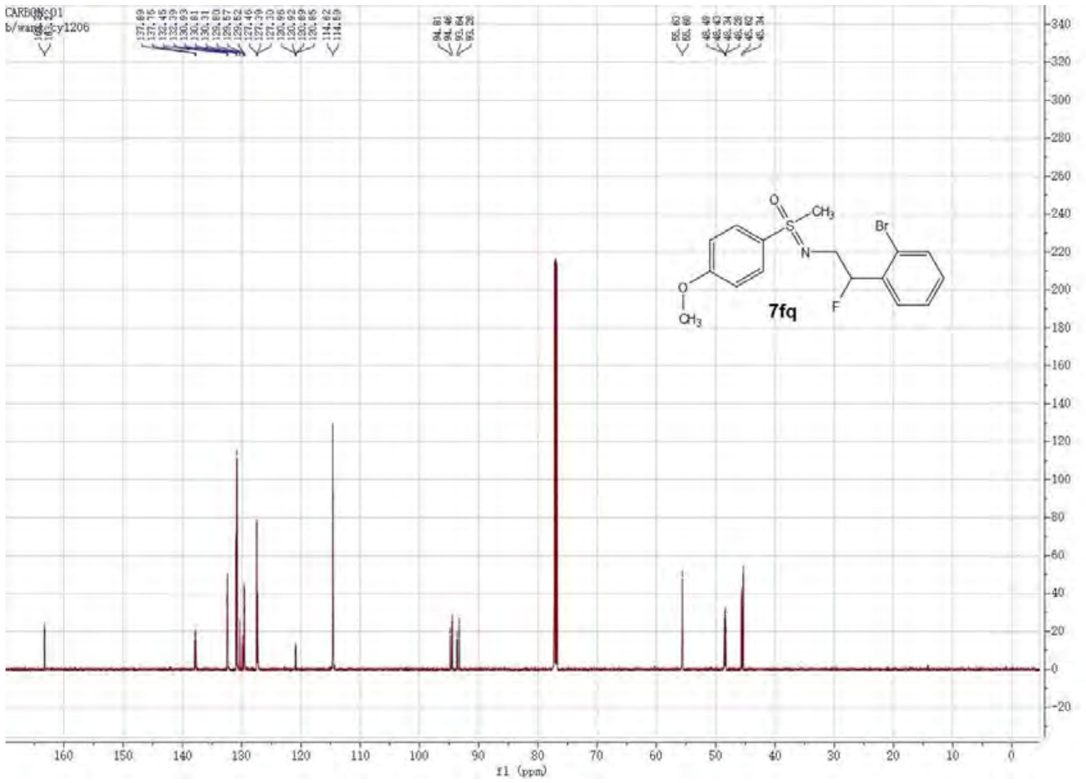

^{13}C NMR spectrum of compound **7fq** (151 MHz, $CDCl_3$)

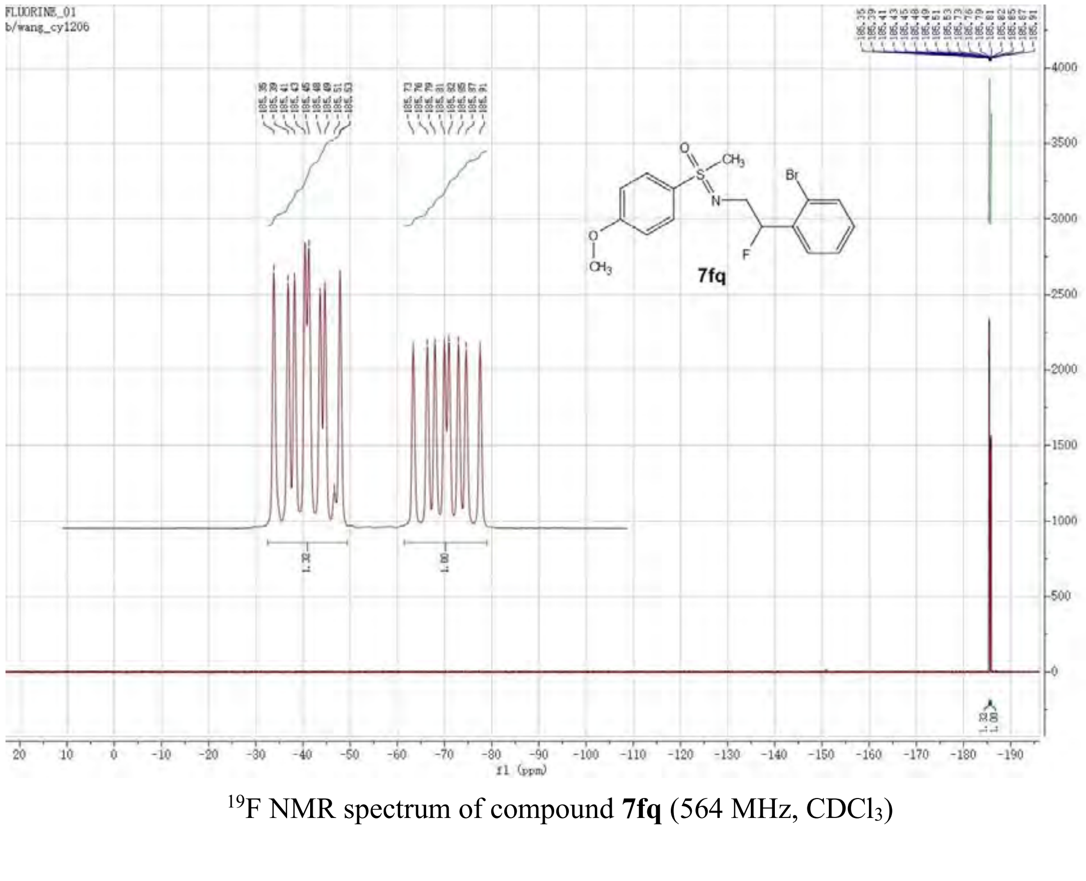

^{19}F NMR spectrum of compound **7fq** (564 MHz, $CDCl_3$)

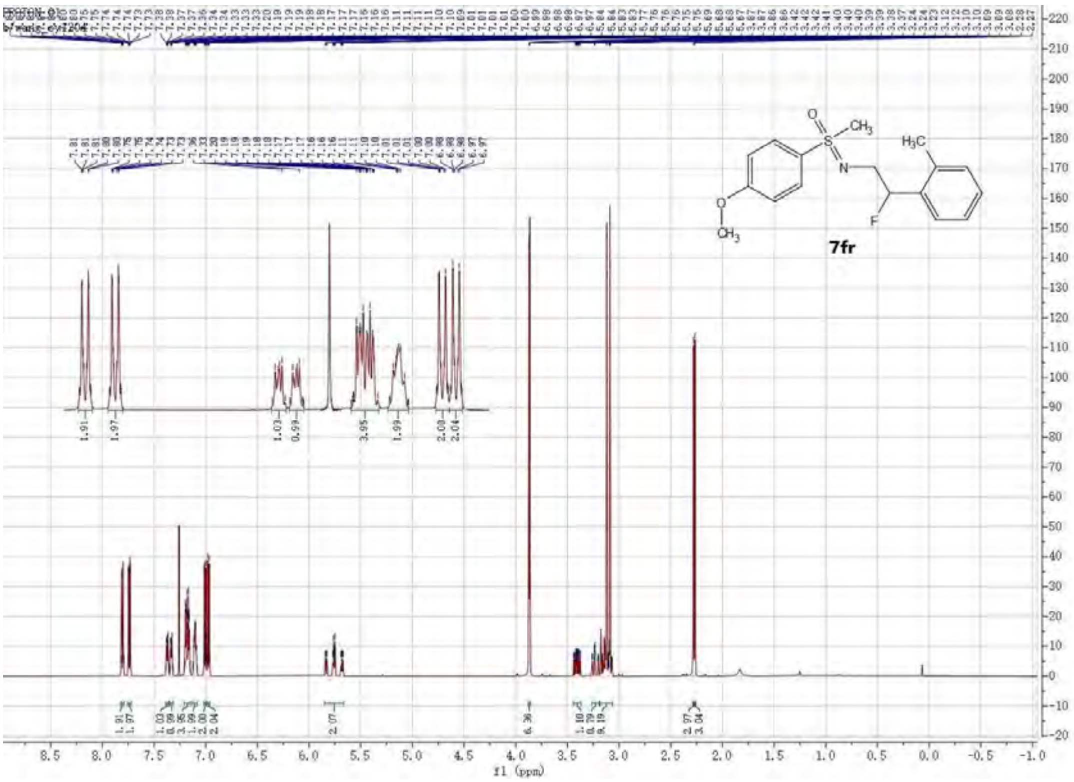

^{1}H NMR spectrum of compound **7fr** (600 MHz, $CDCl_3$)

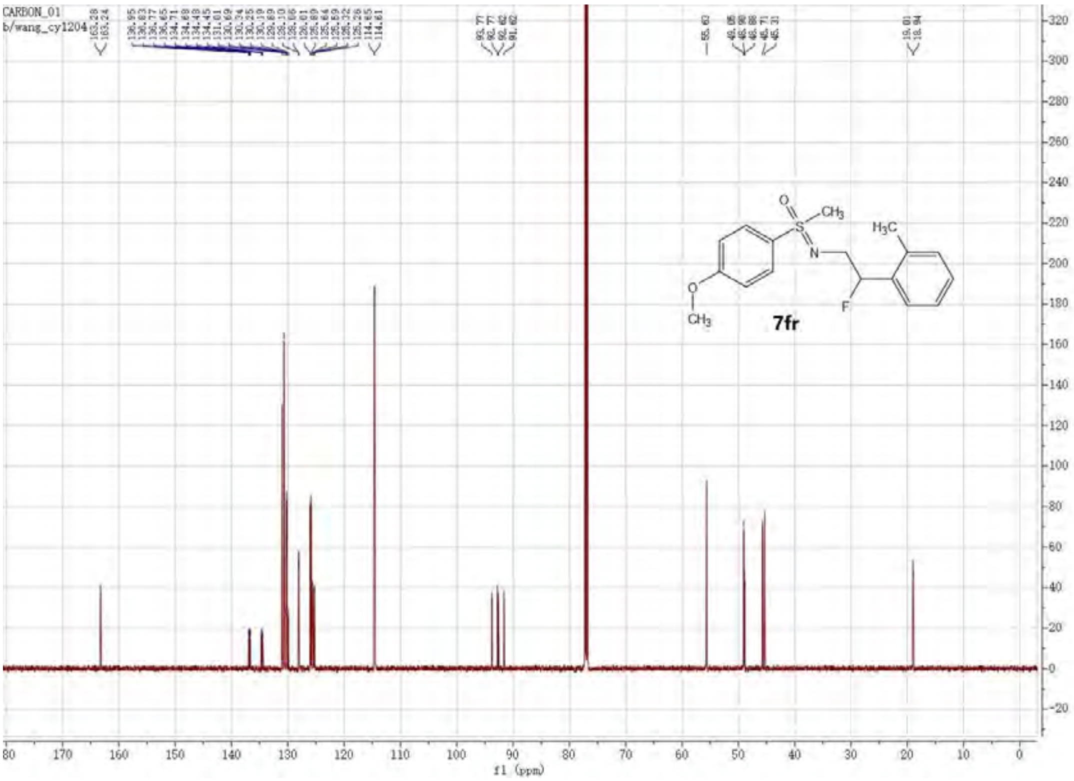

^{13}C NMR spectrum of compound **7fr** (151 MHz, $CDCl_3$)

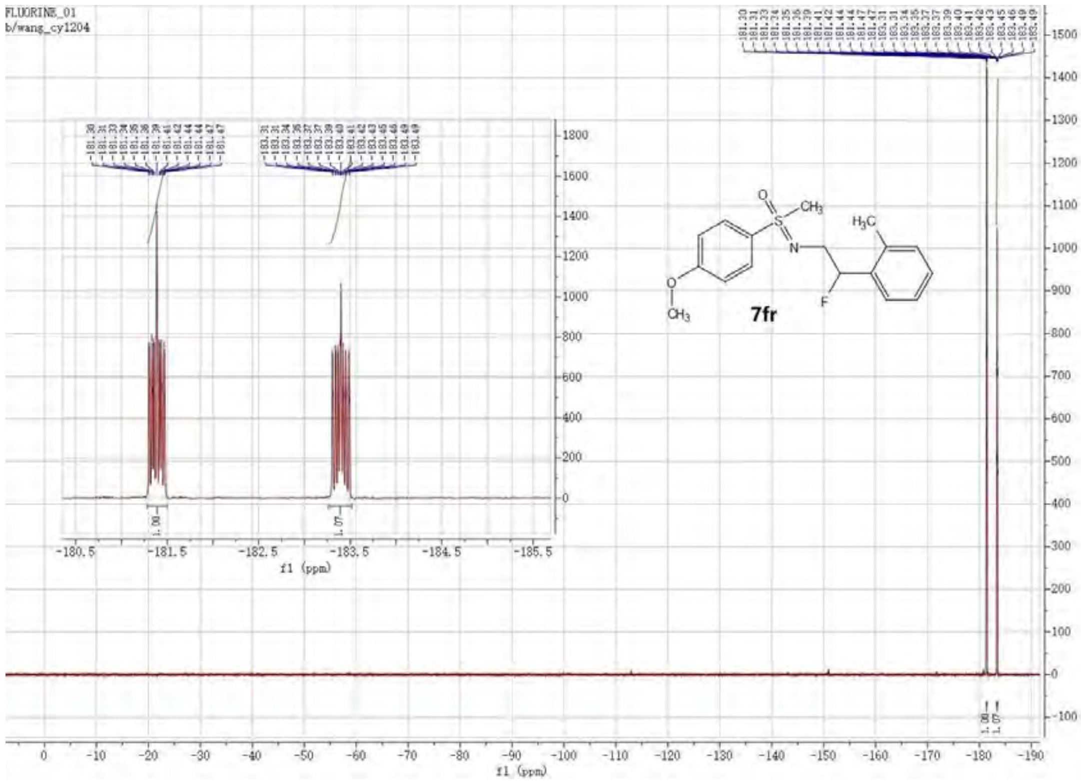

^{19}F NMR spectrum of compound **7fr** (564 MHz, $CDCl_3$)

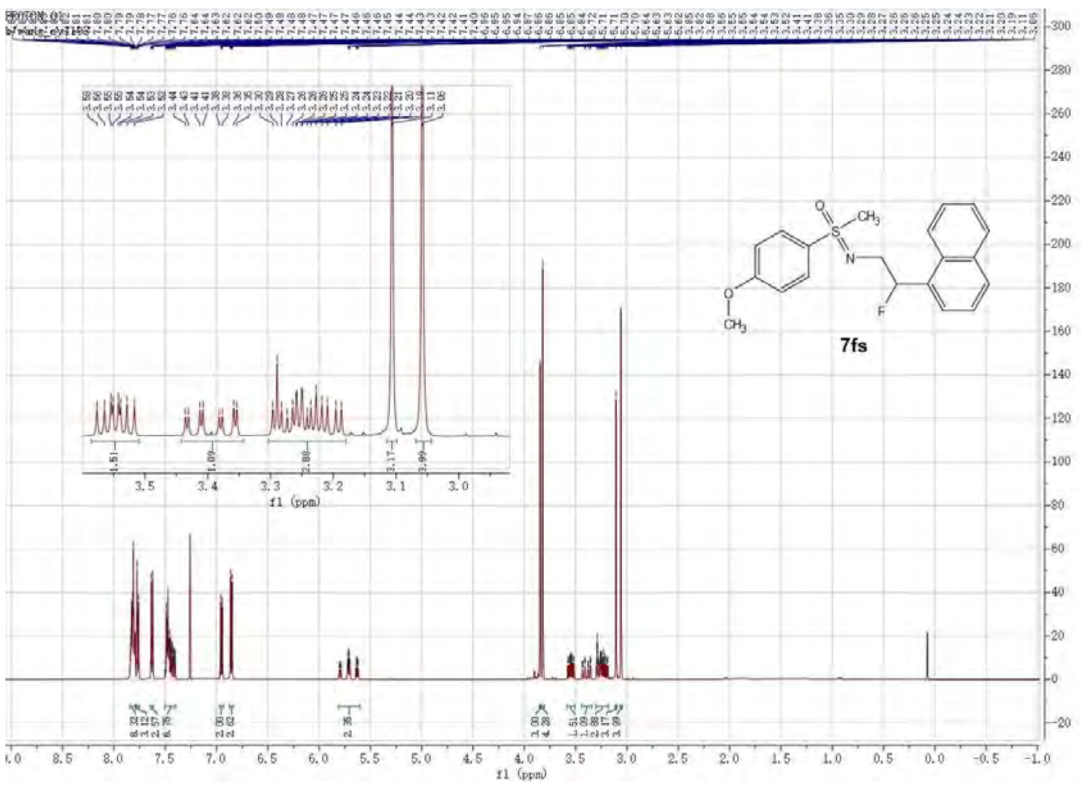

^{1}H NMR spectrum of compound **7fs** (600 MHz, $CDCl_3$)

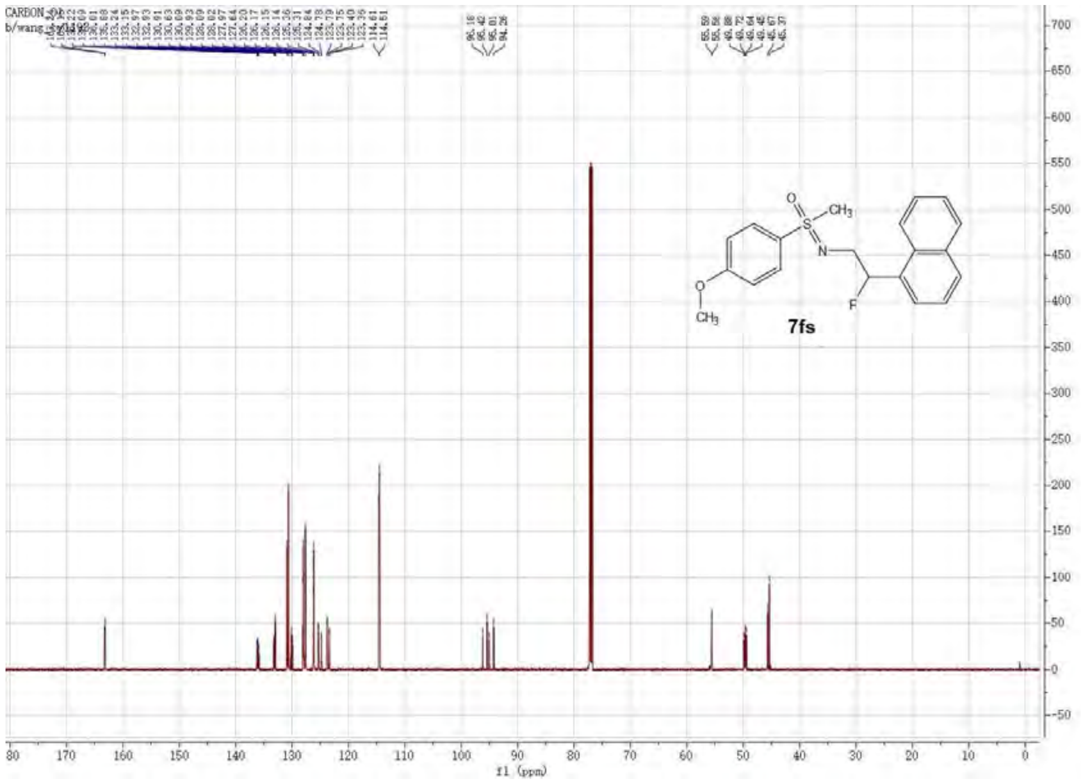

^{13}C NMR spectrum of compound **7fs** (151 MHz, $CDCl_3$)

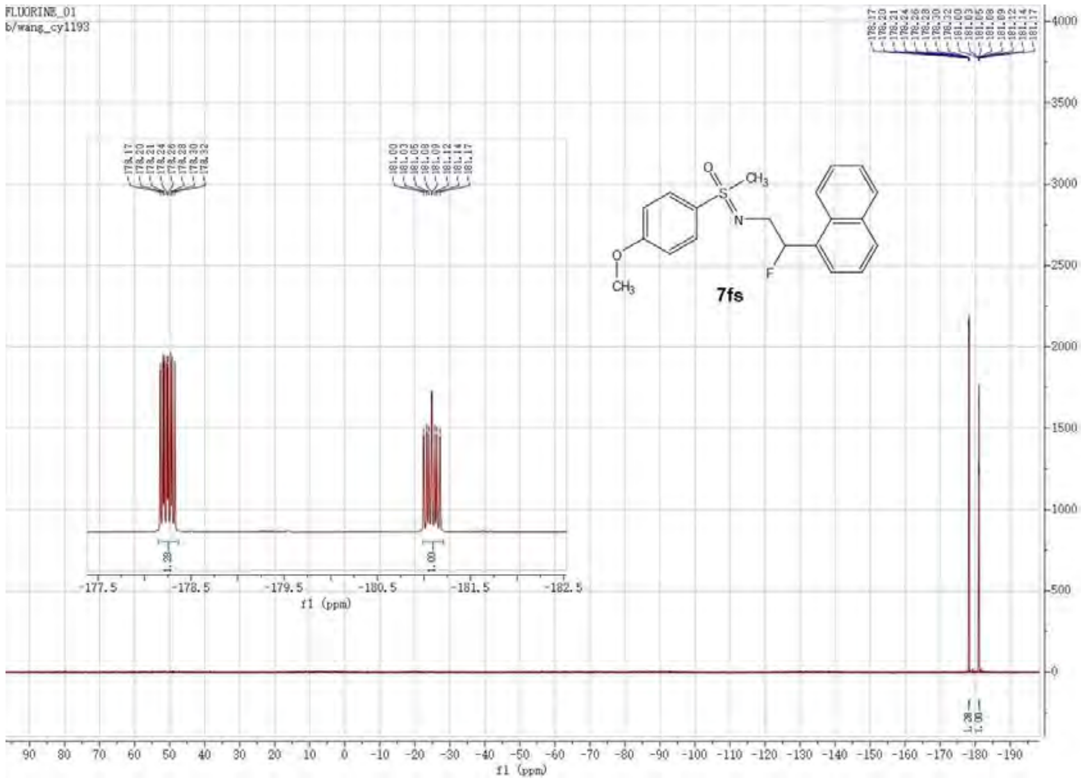

^{19}F NMR spectrum of compound **7fs** (564 MHz, $CDCl_3$)

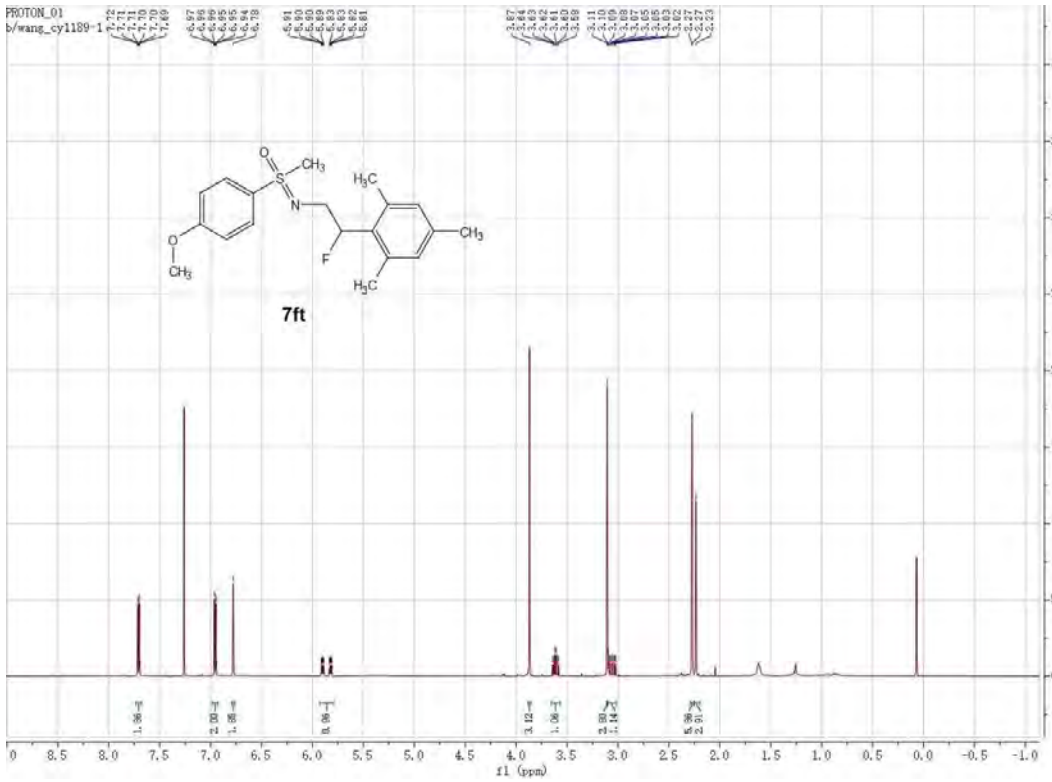

^{1}H NMR spectrum of compound **7ft** (600 MHz, $CDCl_3$, major of diastereomers)

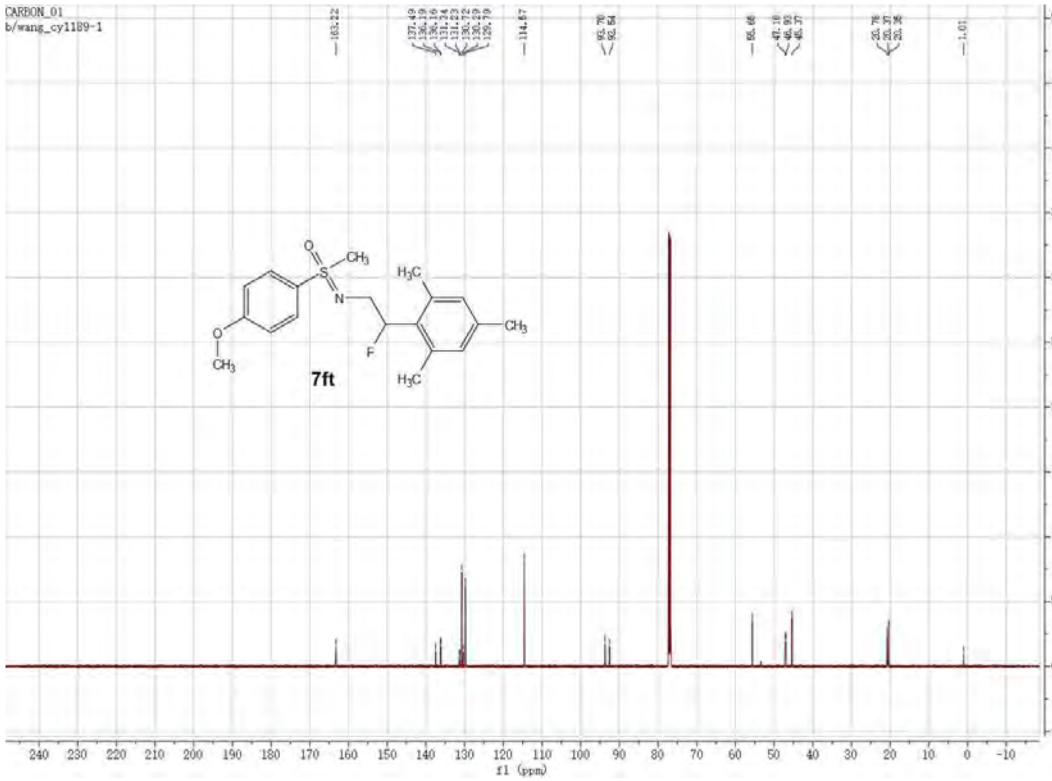

^{13}C NMR spectrum of compound **7ft** (151 MHz, $CDCl_3$, major of diastereomers)

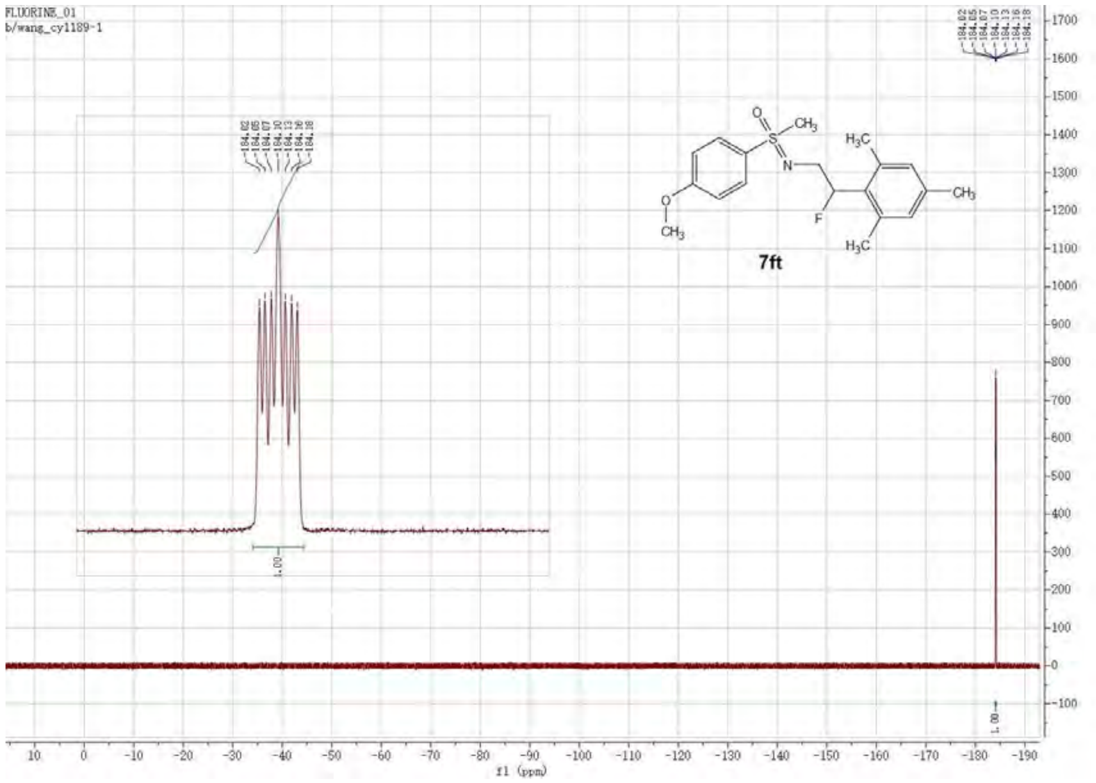

^{19}F NMR spectrum of compound **7ft** (564 MHz, $CDCl_3$, major of diastereomers)

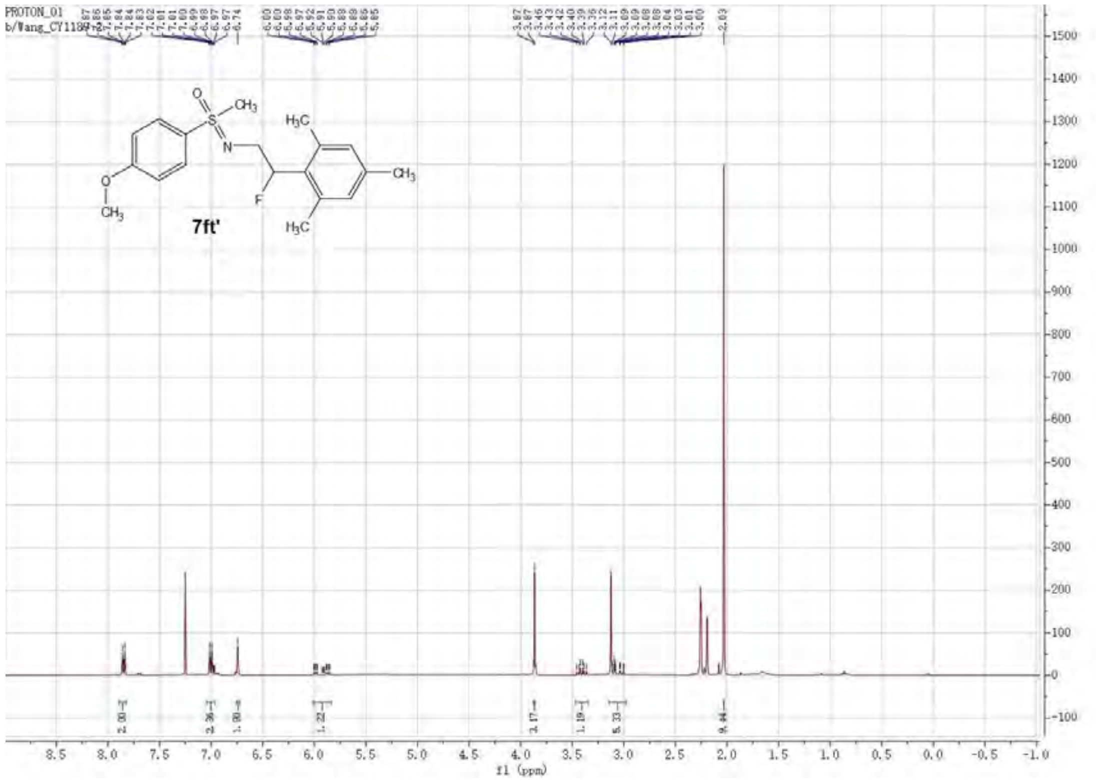

^{1}H NMR spectrum of compound **7ft'** (600 MHz, $CDCl_3$, minor of diastereomers)

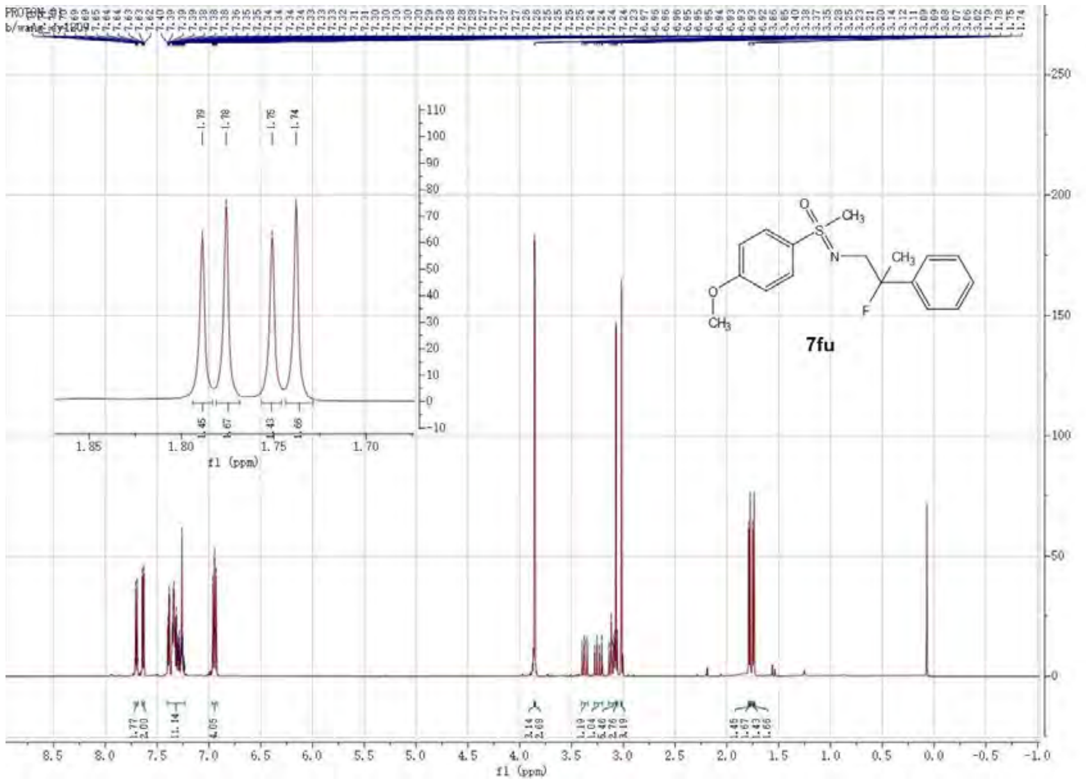

^{1}H NMR spectrum of compound **7fu** (600 MHz, $CDCl_3$)

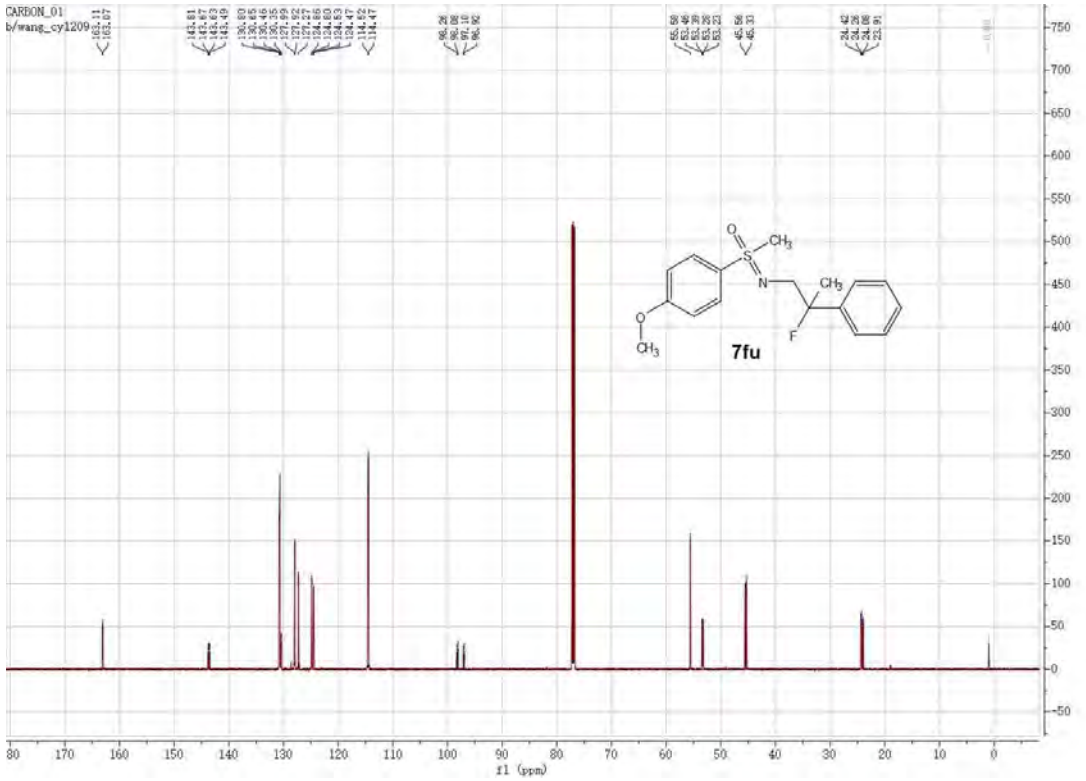

^{13}C NMR spectrum of compound **7fu** (151 MHz, $CDCl_3$)

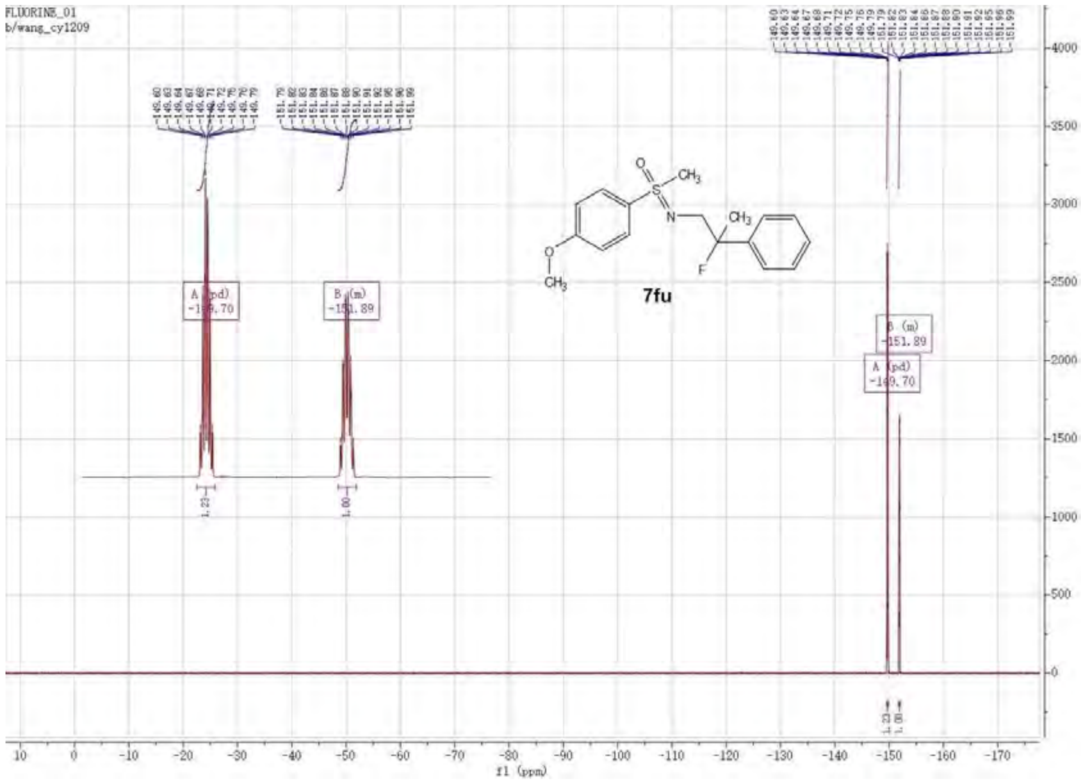

^{19}F NMR spectrum of compound **7fu** (564 MHz, CDCl$_3$)

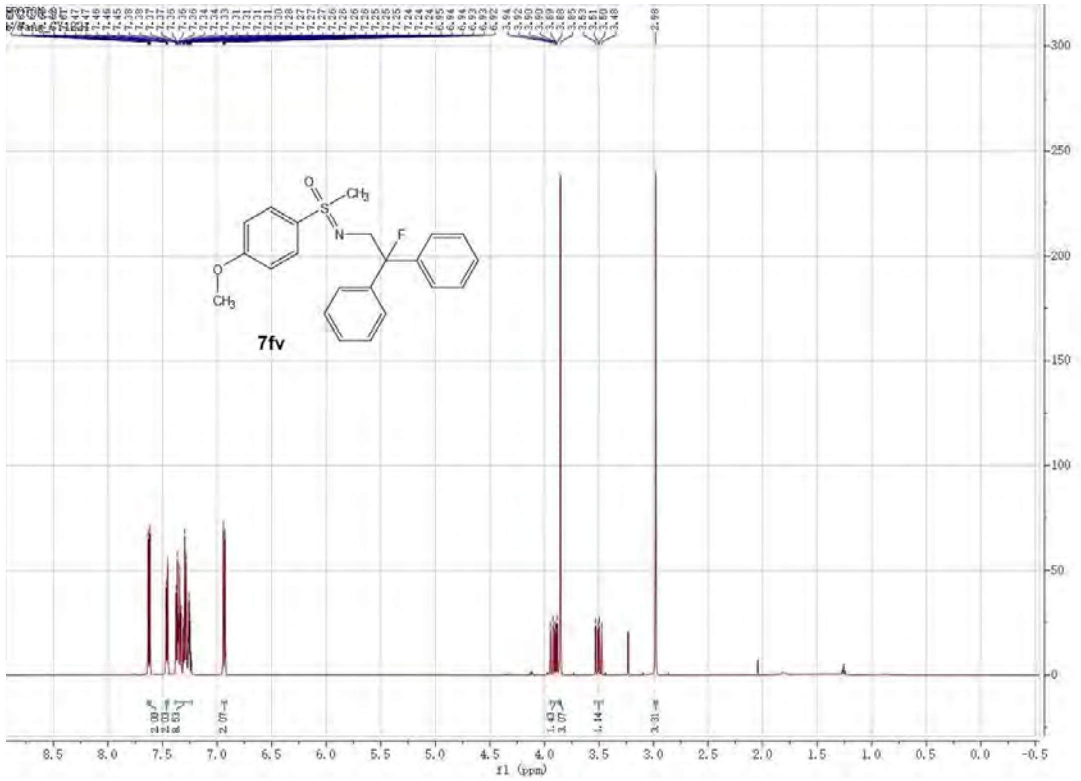

^{1}H NMR spectrum of compound **7fv** (600 MHz, CDCl$_3$)

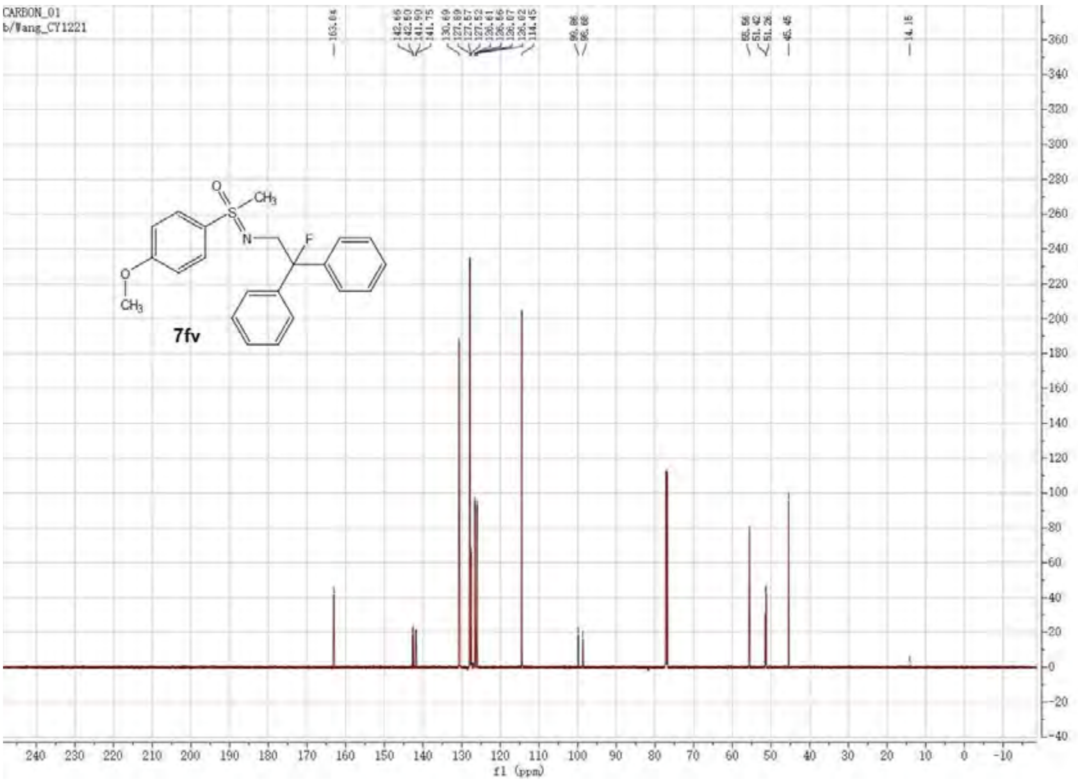

^{13}C NMR spectrum of compound **7fv** (151 MHz, $CDCl_3$)

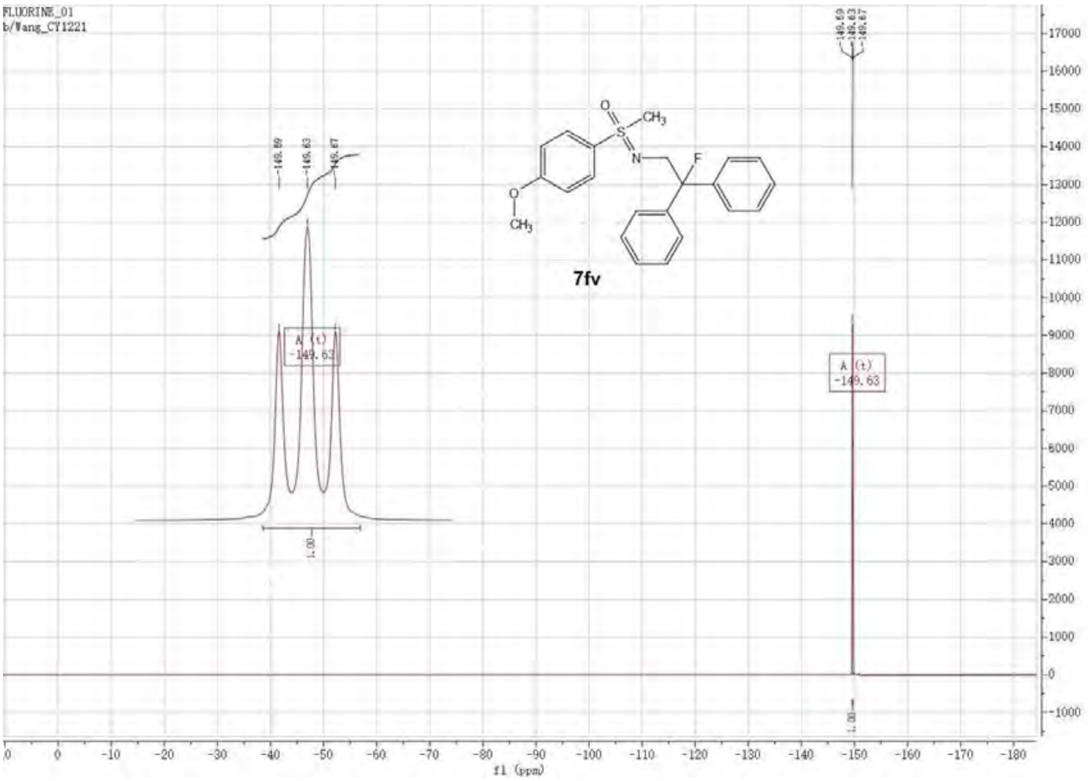

^{19}F NMR spectrum of compound **7fv** (564 MHz, $CDCl_3$)

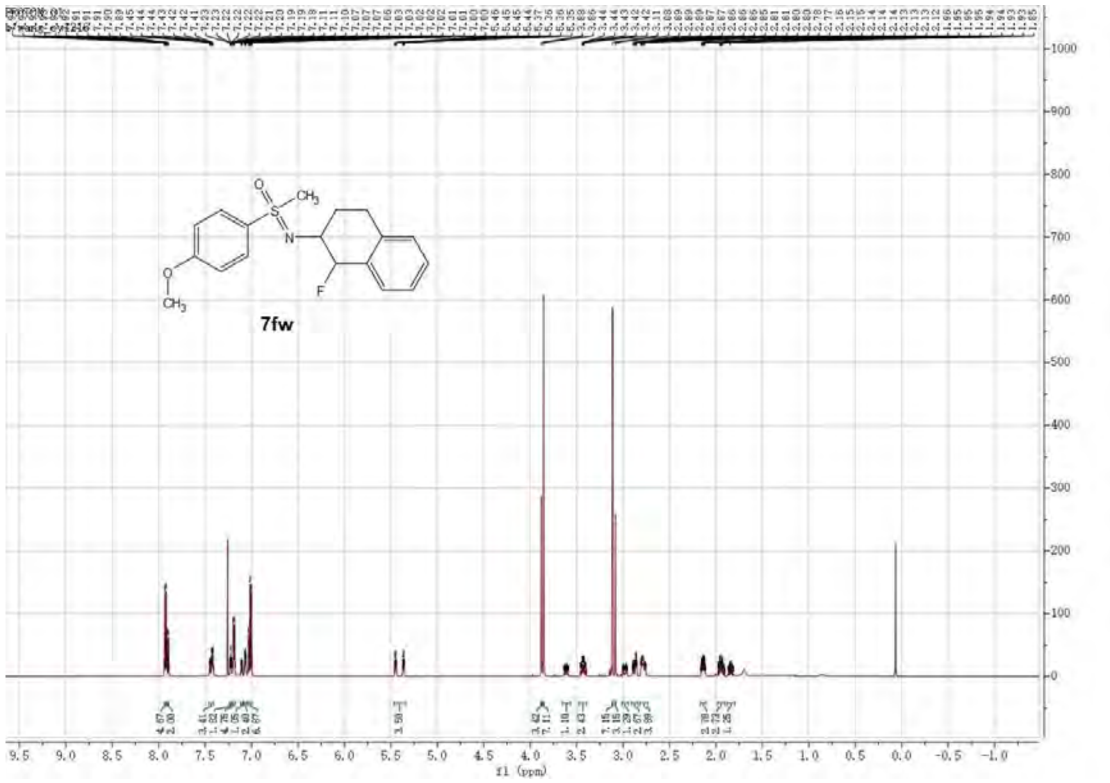

^{1}H NMR spectrum of compound **7fw** (600 MHz, $CDCl_3$)

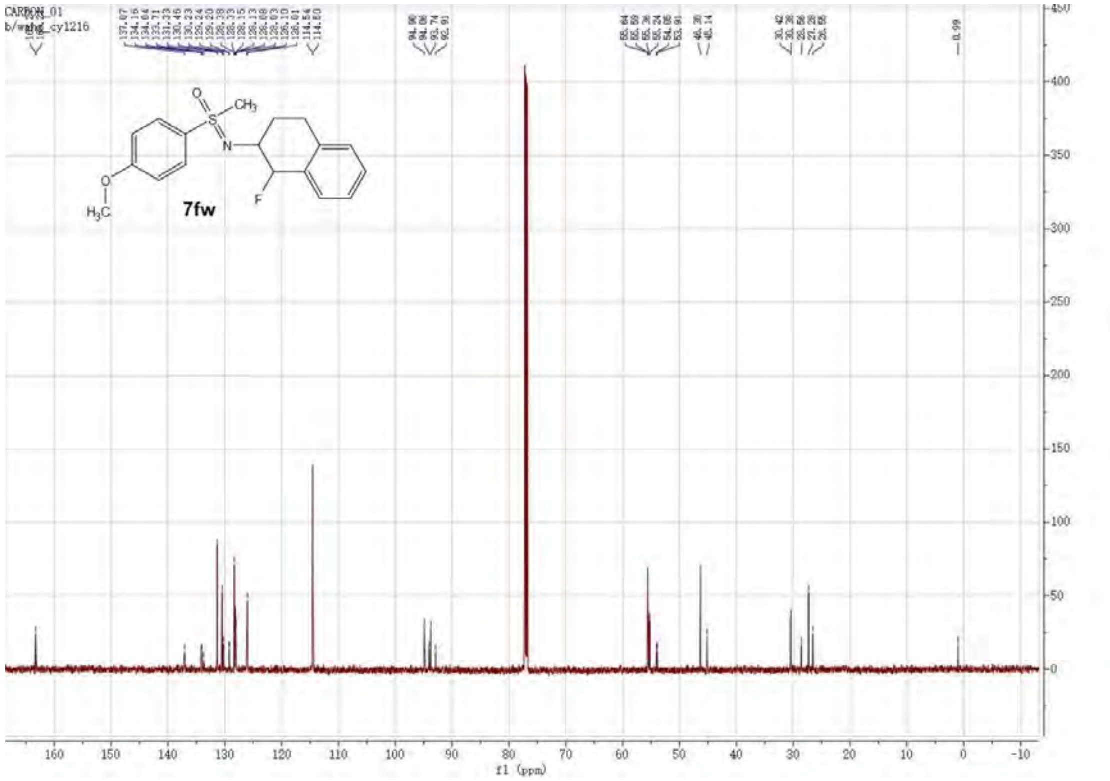

^{13}C NMR spectrum of compound **7fw** (151 MHz, $CDCl_3$)

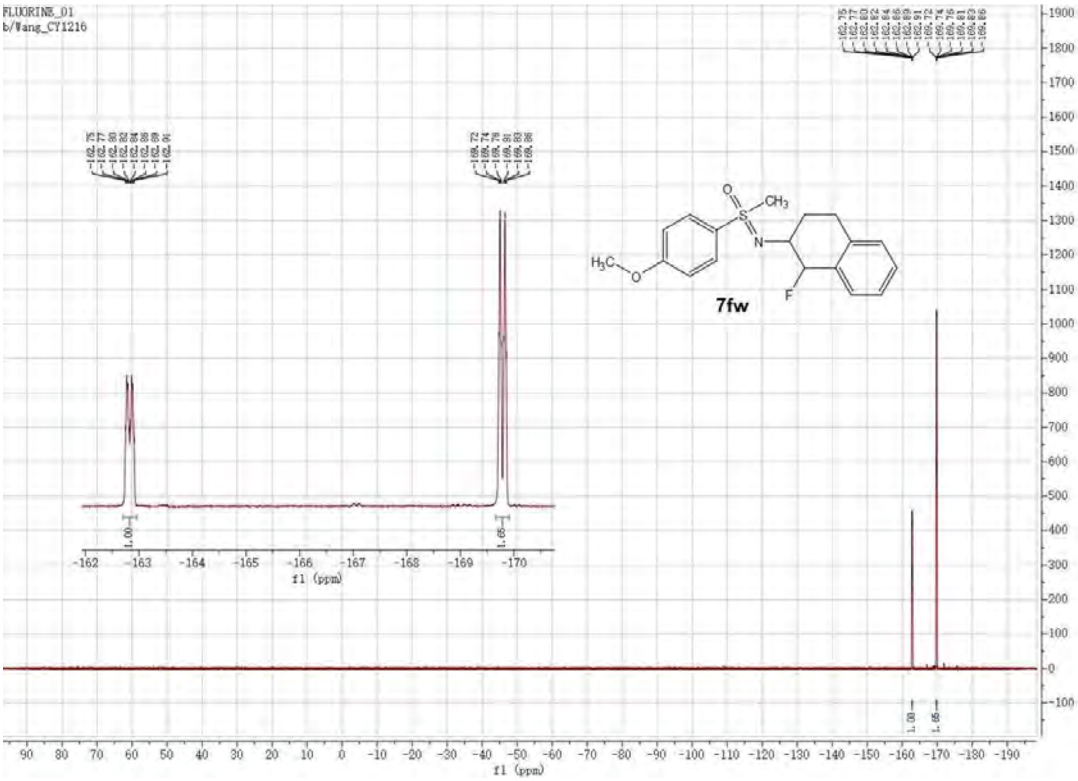

^{19}F NMR spectrum of compound **7fw** (564 MHz, $CDCl_3$)
